# Sublethal Exposure Effects of the Neonicotinoid Clothianidin Strongly Modify the Brain Transcriptome and Proteome in the Male Moth *Agrotis ipsilon*

**DOI:** 10.3390/insects12020152

**Published:** 2021-02-11

**Authors:** Camille Meslin, Françoise Bozzolan, Virginie Braman, Solenne Chardonnet, Cédric Pionneau, Marie-Christine François, Dany Severac, Christophe Gadenne, Sylvia Anton, Martine Maibèche, Emmanuelle Jacquin-Joly, David Siaussat

**Affiliations:** 1 Département Ecologie Sensorielle, Institut d’Ecologie et des Sciences de l’Environnement de Paris (iEES-Paris), Sorbonne Université, INRAE, CNRS, IRD, UPEC, Université de Paris, 75005 Paris, France; Camille.Meslin@inrae.fr (C.M.); Francoise.Bozzolan@sorbonne-universite.fr (F.B.); virginie.braman@upmc.fr (V.B.); marie-christine.francois@inrae.fr (M.-C.F.); martine.maibeche@sorbonne-universite.fr (M.M.); emmanuelle.joly@inrae.fr (E.J.-J.); 2 Département Ecologie Sensorielle, Institut d’Ecologie et des Sciences de l’Environnement de Paris (iEES-Paris), Sorbonne Université, INRAE, CNRS, IRD, UPEC, Université de Paris, 78026 Versailles, France; 3 Plateforme Post-Génomique de la Pitié-Salpêtrière (P3S), UMS 37 PASS, INSERM, Sorbonne Université, 75013 Paris, France; solenne.chardonnet@sorbonne-universite.fr (S.C.); cedric.pionneau@sorbonne-universite.fr (C.P.); 4 MGX, BioCampus Montpellier, CNRS, INSERM, Université de Montpellier, 34000 Montpellier, France; Dany.Severac@mgx.cnrs.fr; 5 Institut de Génétique Environnement et Protection des Plantes IGEPP, INRAE, Institut Agro, Université de Rennes, 49045 Angers, France; antongadenne@orange.fr (C.G.); sylvia.anton@inrae.fr (S.A.)

**Keywords:** pest insect, clothianidin, proteomics, transcriptomics, hormesis, *Agrotis ipsilon*

## Abstract

**Simple Summary:**

Insect pest management relies mainly on neurotoxic insecticides, including neonicotinoids such as clothianidin. Low doses of insecticides can stimulate various life traits in target pest insects, whereas negative effects are expected. We recently showed that treatments with different low doses of clothianidin could modify behavioral and neuronal sex pheromone responses in the male moth, *Agrotis ipsilon*. In this study, we showed that clothianidin disrupted 1229 genes and 49 proteins at the molecular level, including numerous enzymes of detoxification and neuronal actors, which could explain the acclimatization in pest insects to the insecticide-contaminated environment.

**Abstract:**

Insect pest management relies mainly on neurotoxic insecticides, including neonicotinoids such as clothianidin. The residual accumulation of low concentrations of these insecticides can have positive effects on target pest insects by enhancing various life traits. Because pest insects often rely on sex pheromones for reproduction and olfactory synaptic transmission is cholinergic, neonicotinoid residues could indeed modify chemical communication. We recently showed that treatments with low doses of clothianidin could induce hormetic effects on behavioral and neuronal sex pheromone responses in the male moth, *Agrotis ipsilon*. In this study, we used high-throughput RNAseq and proteomic analyses from brains of *A. ipsilon* males that were intoxicated with a low dose of clothianidin to investigate the molecular mechanisms leading to the observed hormetic effect. Our results showed that clothianidin induced significant changes in transcript levels and protein quantity in the brain of treated moths: 1229 genes and 49 proteins were differentially expressed upon clothianidin exposure. In particular, our analyses highlighted a regulation in numerous enzymes as a possible detoxification response to the insecticide and also numerous changes in neuronal processes, which could act as a form of acclimatization to the insecticide-contaminated environment, both leading to enhanced neuronal and behavioral responses to sex pheromone.

## 1. Introduction

Although integrated pest management strategies are increasingly being developed [1], the majority of treatments for pest insects still rely exclusively on the use of neurotoxic chemicals, such as neonicotinoid insecticides [2]. These molecules, including the widely used last-generation insecticide clothianidin, are known to disrupt synaptic transmission through their action on nicotinic acetylcholine receptors [3,4]. The widespread use of these neurotoxic insecticides raises numerous issues, such as residual accumulation in the environment [5], which is present for many years even after stopping treatments, and negative effects on physiology and behavior of non-target insects such as honeybees [6]. Indeed, there is growing evidence that sublethal or low doses of neonicotinoids impact insect physiology and thus vital behaviors such as reproduction or searching for food [7,8].

Contrary to these effects disturbing insect physiology and behavior, low doses of insecticides can also elicit hormetic effects—i.e., enhance certain physiological and behavioral traits. Hormesis is defined as a biphasic response following exposure to a given toxicant with beneficial effects at low-dose exposure and adverse effects at high-dose exposure [9]. Numerous examples of this toxicological phenomenon have been reported for many types of biological and pathological processes in microorganisms, plants, and mammals including humans [10,11]. In insects, insecticide-induced hormesis in developmental and reproductive life traits (such as growth stimulation, enhanced pupation, decrease in pupal mortality, increased fecundity and longevity, and increase in oviposition) has likewise been observed following treatments with different insecticides, including neonicotinoids, carbamates, and organophosphates [12,13,14]. In addition to the mentioned life traits, insecticides also interfere with chemical communication in insects: they can, for example, disrupt the behavioral response of pest insects to sex pheromones or food odors [15,16,17,18]. Recent results from the black cutworm, *Agrotis ipsilon* (Hufnagel) (Lepidoptera: Noctuidae), show that low doses of the neonicotinoid clothianidin induce a biphasic effect on pheromone-guided behavior with a hormetic-like effect [17]. In another moth species, the cotton leafworm *Spodoptera littoralis* (Boisduval) (Lepidoptera: Noctuidae), the same effect has been observed for deltamethrin, and detailed investigations revealed that these effects were restricted to the response of males to the sex pheromone produced by conspecific females, whereas no effect of the same doses was observed on behavioral responses to food odor [16].

Although evidence is accumulating that the concept of hormesis is valid for many insecticides used to control pest insects, very little is known about the molecular and cellular mechanisms leading to this phenomenon [14]. Most likely, the effects are due to changes in the expression of genes involved in basic cellular and physiological processes. The recent progresses in “omic” approaches have opened up possibilities to decipher such changes, especially in non-model species such as crop pest insects. It is now possible to acquire a large amount of data on gene expression (transcriptomics, e.g., RNAseq) or protein levels (proteomics) on a given species and in different experimental conditions, pinpointing any mechanisms or possible regulatory pathways involved, without any a priori study. However, most of such studies have mainly focused on gene expression alone (see, for example, [19]), and rare are those that also take into account protein levels [20].

The well described olfactory plasticity and available molecular data on neuromodulatory mechanisms in *A. ipsilon* males [21,22] make it an excellent model with which to study the molecular mechanisms responsible for the hormetic action of pesticides on behavior and on the central nervous system.

In our previous study, the lethal dose 50 (LD50: dose resulting in 50% mortality in tested insects) was found to be 69 ng/moth in male *A ipsilon* [17]. At higher doses, intoxicated insects exhibited trembling and incapacity to move before dying. In the low lethal dose range, 5 ng of clothianidin decreased the proportion of males able to fly at the exception of the LD20 dose (10 ng), which induces a hormetic-like effect. Indeed, at this dose, we observed an improved orientation behavior of males in response to female pheromone, whereas no clothianidin effect was observed on behavioral responses to plant odor [17]. We also discovered that this dose of clothianidin modifies the pheromone response thresholds of central neurons of *A. ipsilon*, but not those of peripheral olfactory receptor neurons [23]. This correlates with the changes in behavioral responses after clothianidin treatment and suggests the antennal lobe—i.e., the part of the brain that processes responses to odorants—as the neural substrate involved in clothianidin-induced behavioral changes. We therefore attempted to explore the molecular mechanisms underlying the hormetic effects within the brain, using combined transcriptomic and proteomic analyses of male *A. ipsilon* brains originating from individuals orally treated with a LD20 dose of clothianidin dissolved in dimethyl sulfoxide (DMSO), compared with untreated individuals and with individuals treated with the solvent DMSO alone in the same way as in Rabhi et al. [17,23]. Concerning oral treatments with insecticides, even though the gut epithelium is supposed to have a barrier role with the presence of a high number and quantity of detoxification enzymes, several studies have shown that the ingestion of pesticides can induce strong effects in the brain, such as the degeneration of neural tissue or the molecular alteration of neuronal actors and degradation/detoxification enzymes [24,25,26]. Because obvious physiological and behavioral effects in *A. ipsilon* with oral insecticide treatments were observed, we kept the same approach for our molecular analysis.

This approach allowed us to perform an unbiased search for genes and corresponding proteins, which could be modulated by the insecticide treatments and eventually by the solvent treatment. We expected to identify the genes and proteins involved in different physiological processes, among which would be those possibly responsible for the modified neuronal odor responses [23].

## 2. Materials and Methods

### 2.1. Insects

Adult males of *A. ipsilon* Hufnagel originated from a laboratory colony. Wild insects were introduced into the colony each spring to maintain the genetic diversity and overall health of the colony. Insects were reared on an artificial diet [27] in individual cups at 22 °C under a 16 h:8 h light:dark photoperiod until pupation. Pupae were sexed, and males and females were kept separately at 22 °C in an inversed light–dark cycle (16 h:8 h light:dark photoperiod). Newly emerged adults were removed every day from hatching containers and were given access to a 20% sucrose solution ad libitum. The day of emergence was considered as Day 0.

### 2.2. Chemicals

For the stock solution, 25 µg of clothianidin (99% purity, Sigma-Aldrich, Saint-Quentin Fallavier, France) were dissolved in 100 µL of 2% DMSO. The solution was diluted to 1 ng/µL clothianidin with a 20% sucrose solution, with the final concentration of DMSO being 0.008%. Fresh solutions were prepared weekly as needed from frozen aliquots of the stock solution. All the chemicals were purchased from Sigma-Aldrich (Saint-Quentin Fallavier, France), unless stated otherwise.

### 2.3. Clothianidin Intoxication

Oral acute clothianidin intoxications were performed as described previously [17]. Briefly, 4-day-old males were each fed with 10 µL of the freshly prepared clothianidin solution, resulting in a dose of 10 ng of insecticide per insect. We chose 4-day-old males for treatments because the effects of low doses of clothianidin were assessed 24 h after treatment (day 5), which corresponds to the tested age in wind tunnel and electrophysiological experiments and to the optimal age for behavioral responses to sex pheromone in wind tunnel experiments [27]. Males were individually treated with clothianidin before the onset of the scotophase under a ventilated fume hood, as described previously [17]. In order to control for the effect of DMSO (clothianidin solvent), animals were exposed to DMSO alone in the same way at the same final concentration (0.008%). Once the males had ingested the presented solution, they were placed in individual plastic cups and transferred to a climate chamber different from the rearing room for 24 h until the dissection of the brains for proteomic and transcriptomic analyses. Control and treated males originated from at least 3 different generations. Brains from the 3 groups of *A. ipsilon* males (not treated, DMSO-treated, 10 ng of clothianidin-treated) were independently dissected at day 5 after their emergence in Ringer solution and immediately dipped in Eppendorf vials kept in liquid nitrogen, then stored at −80 °C until further treatment. For RNAseq, 50 brains from a given condition were cumulated to make one sample, and this was replicated 3 times for each condition. In total, 9 samples of 50 brains each were prepared. For proteomics, the same procedure was performed to produce 15 samples (5 replicates for each condition), except that 30 to 50 brains were used for each sample.

### 2.4. Transcriptomic Analyses

Total RNA extractions were performed on the 9 brain samples using TRIzol (Invitrogen, Paris, France), according to the manufacturer’s protocol. Total RNA samples were stored at −80 °C until library preparation and sequencing. All the samples were processed at the MGX platform (Montpellier, France). All 9 libraries were prepared separately using the TruSeq Stranded mRNA Sample Preparation Kit (Illumina, Paris, France) according to the manufacturer’s protocol and sequenced on an Illumina HiSeq2000 to generate paired-end reads of 150 bp. After trimming off the adaptor sequences, raw reads were processed in terms of both their quality and length using Cutadapt [28]. Reads were scanned and trimmed off when a quality score <30 was encountered. Reads with a length <20 bp were discarded. Clean Illumina single-end reads from a previous round of *A. ipsilon* brain sequencing [21] were added for the de novo assembly of the transcriptome, generating 734,263,081 clean paired-end reads and 86,325,883 clean single-end reads that were used for the transcriptome reconstruction using the MIRA assembler v4.0.2 with default parameters [29]. MIRA generated 514,857 contigs, and multiple filtration steps were then applied to reduce the complexity of the de novo transcriptome. First, only contigs with a length >200 bp were kept. Second, CD-HIT [30,31] was applied with default parameters to lower the redundancy. All the Illumina reads were then mapped to the new transcriptome, and only the contigs with an expression >1 fragment per kilobase of exon per million fragments mapped (FPKM) were kept. Finally, only contigs with an open reading frame >30 amino acids were kept, resulting in a final *A. ipsilon* brain transcriptome of 17,986 contigs. The completeness of the transcriptome was assessed using BUSCO v3.0.2 [32] and the Insecta gene reference set. The functional annotation of the contigs was carried out by (1) blastp against the nr database (NR-2016-12-09) and blastx against the Uniprot-sprot database to capture BLAST homologies, (2) running HMMER to identify protein domains [33], (3) running SignalP [34] to predict signal peptides, and (4) running TMHMM v2.0 to predict the transmembrane regions [35]. Gene Ontologies (GO) were mapped to each transcript according to the annotation of their best blast hit by blastp and blastx and assigned to 12,627 contigs. GO Slim annotations were used in order to give a broad overview of the ontology content. Enrichment or depletion for GO categories was determined in comparison to the whole GO-annotated transcriptome using the Fisher exact test and was considered significant when the FDR (False Discovery Rate) was <0.1.

### 2.5. Abundance Estimation and Differential Expression Analysis

All the clean reads from the 9 samples generated in this study were mapped on the assembly using a Bowtie aligner [36]. Transcript abundance was estimated for each sample using RNA-Seq by Expectation Maximization (RSEM) [37] and was measured as the FPKM values. RNAseq counts were normalized between the different samples and replicates using the trimmed mean of M-values normalization method (TMM) [38]. After that step, a quality check was performed to determine if the biological replicates were well correlated for each condition. That quality check revealed that for each condition, one sample did not correlate with the two others. These outliers (DMSO1, clothianidin2 and Control3) were removed from further analyses and the correlations between samples were checked again (Supplementary Data 1). Differentially expressed transcripts were identified using edgeR within the Bioconductor package [39] by taking into account two biological replicates per condition. Genes were considered differentially expressed for an FDR (False Discovery Rate) <0.10.

### 2.6. Proteomics Analysis

Three biological replicates were prepared for each condition and analyzed using a proteomics approach [40]. Proteins were extracted using a polytron in 2D buffer (Urea 7 M; Thiourea 2 M; CHAPS 1%; SB3-10 0.5%; Triton-X100 0.5%; Isobutanol 10%, 25 mM Tris pH 8.8). Protein content was assessed using the Bradford quick start protein assay (BioRad Paris, France). An internal standard was prepared by pooling an equal amount of all samples. Protein labelling was performed using the 3Dye Cy2/3/5 fluor labelling (FluoProbe, Interchim, Paris, France) with 400 pmol of CyDye for 50 µg of protein, incubated 30 min on ice, then quenched with 0.35 mM of Lysine for 10 min. Cy3 and Cy5 were used to label individual samples whereas Cy2 was used to label the internal standard. Following labelling, samples were stored at −80 °C until use. 2DE was run on 24 cm gels in two complementary pH ranges, pH 5–8 and pH 6–9, using commercial strips (GE Healthcare, Paris, France). Each strip was run with 50 µg of internal standard labelled with Cy2 and 50 µg of two different samples labelled with Cy3 and Cy5, respectively. Strips were passively rehydrated overnight directly with the samples diluted in a rehydration buffer (Urea 7 M; Thiourea 2 M; CHAPS 1%; SD3-10 0.5%; Triton-X100 0.5%; Isobutanol 10%, 40 mM DTT, 0.5% ampholites) or for a basic pH range of 6–9 strips, with a rehydration buffer only (Urea 7 M, Thiourea 2 M, CHAPS 4%, Triton X-100 0.05%, glycerol 5% and Destreak 10 mg/mL). Isoelectrofocalisation (IEF) of the pH range 5–8 strips was performed on an Ettan IPGphor (GE Healthcare) as follows: 2 h at 50 V, 2 h at 200 V, 2 h gradient from 200 V to 1000 V, 2 h at 1000 V, 2 h gradient from 1000 V to 10,000 V, 7 h at 10,000 V. For basic pH range 6–9 strips, samples were incorporated by cup-loading during IEF, as described by [41]. The IEF running program was: 7 h at 50 V, 2 h at 500 V, 2 h gradient from 500 V to 1000 V, 7 h gradient from 1000 V to 10,000 V, 2.5 h at 10,000 V. Strips were incubated for 15 min in equilibration buffer (Urea 6 M, Tris pH 8.8 75 mM, Glycerol 26%, SDS 2%) supplemented with 65 mM of DTT, then for 20 min in equilibration buffer supplemented with 135 mM of iodoacetamide. The second dimension was run in 12% acrylamide gels at 30 V for the first hour, then 150 V and 12 mA per gel in a Tris-Glycine buffer. Gel images were acquired on a scanner Ettan DIGE Imager (GE Healthcare). Images were analyzed using Progenesis SameSpots 3.2.3107.24565 (Nonlinear Dynamics). Spots were automatically detected and matched, then manually validated. Relative quantification was performed in each individual gel against its own internal standard, and signal intensities were normalized between all gels based on the internal standard signal of one reference gel. Statistical analyses between each pair of sample groups were performed using ANOVA. Significant spots were selected when the fold change was >1.6 with a *p* value < 0.05 or a fold change >1.3 with a *p* value < 0.01 in at least one of the two comparisons: not treated/clothianidin-treated and DMSO-treated/clothianidin-treated (see Table S1 for details).

Spots of interest were excised, cut into 1 mm^3^ cubes and destained in potassium ferricyanure 15 mM and sodium thiosulfate 50 mM. The gel pieces were submitted to reduction by incubation in 10 mM of DTT in 50 mM of ammonium bicarbonate (AmBic, Simga aldrci-, Paris, France) for 30 min at 56 °C, then to alkylation by incubation in 50 mM of iodoacetamide in 50 mM of AmBic for 30 min at room temperature. Proteins were digested with 200 ng of trypsin per spot, overnight at 37 °C, and tryptic peptides were collected. The gel pieces were washed twice in 60% acetonitrile (ACN) and 0.1% trifluoroacetic acid (TFA) for 10 min in an ultrasonic bath. Extracted peptides were concentrated in a speed vacuum dryer and resuspended in 5% ACN with 0.1% formic acid and stored at −80 °C until MS analysis. Peptide mixtures were analyzed by LC-MS/MS on a U3000 nanoLC (Thermo, Paris, France) coupled to an HCTultra ion trap (Bruker). Peptides were concentrated and desalted for 5 min on a pre-column RP-C18 (5 mm, 300 μm i.d., 100 Å, Thermo) with mobile phase A (2% ACN/0.1% formic acid) at a flow rate of 20 μL/min, then separated on an analytical column RP-C18 (15 cm, 75 μm i.d., 100 Å, Dionex, Interchim, Paris, France) at a flow rate of 300 nL/min. Elution gradient was run from 2% to 30% of solvent B (95% ACN/0.1% formic acid) in 35 min and 30% to 40% in 5 min. The ion trap was used in the positive mode with the selection of 8 precursors from each MS spectrum for fragmentation by collision-induced dissociation (CID). The capillary voltage was set at 2 kV. Full scan spectra were acquired in the mass range 250 to 1600 *m*/*z* and MSMS spectra were acquired from 100 to 2800 *m*/*z* with singly charged ion exclusion, a dynamic exclusion of 30 s, and an isolation width of 4 Da. Raw data were processed using Data Analysis 3.4 (Bruker). Mgf files were generated with a maximum of 5000 compounds with a signal intensity threshold of 100,000 (AU) and spectra deconvolution. Protein identification was performed with X!TandemPipeline 3.4.0 (with X!Tandem search engine software 2015.04.01.1) using a database resulting from the translation of *A. ipsilon* transcriptomic data. Trypsin was selected as the enzyme with a maximum of 1 missed cleavage. Carbamidomethylation of Cys was set as a fixed modification, oxidation of Met as variable modification; MS and MS/MS tolerance at 0.5 Da. At least 2 unique peptides with a *p* value < 0.05 were required for protein validation.

## 3. Results

### 3.1. Brain Transcriptome Assembly and Annotation

We obtained 1462 complete and single-copy (83.3%), 81 complete and duplicated (4.9%), 99 fragmented (6%), and 97 missing BUSCO genes (5.8%). The high number of complete genes that were reconstructed shows the depth and the completeness of the final transcriptome. The combination of both previous and actual data (deeper sequencing, longer reads, paired-end libraries) improves the overall statistics of the de novo assembly, as described in Table 1. Compared with the previously published transcriptome [30], the median contig length is doubled, leading to more complete genes, as reflected in the BUSCO statistics. Contigs are also less fragmented, and fewer genes are missing in the new transcriptome. GO annotations were assigned to 12,627 contigs (70.2% of the transcriptome) by using the annotation of their best blastp and blastx hits.

### 3.2. DMSO Exposure Affects Both A. ipsilon Brain Transcriptome and Proteome Profiles

**Figure 1:**
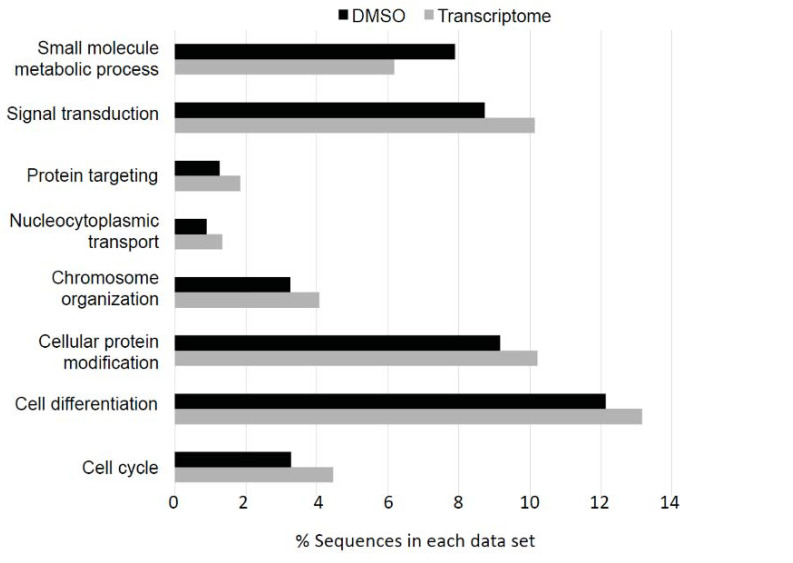
DMSO has an impact on many cellular processes. Gene Ontology Enrichment analysis showing the GO terms of transcripts deregulated after DMSO exposure compared to the control group (test set, black) and that exhibit statistically significant differences with the whole transcriptome (reference set, grey) (Fisher’s exact test, FDR < 0.1). The *X*-axis shows the percentage of sequences in each dataset. The *Y*-axis shows the GO terms. The expression of multiple genes with GO terms reflecting basic cellular processes is affected by DMSO exposure. The GO category “small molecule metabolic process” is enriched in genes deregulated by DMSO (black), while all the other categories are depleted compared to the whole transcript set (light grey).

**Figure 2:**
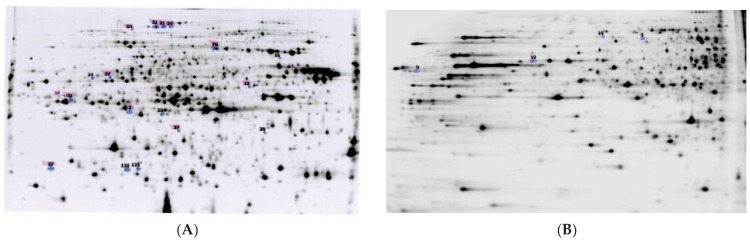
Proteomic profiles showing protein abundance between control and DMSO-treated brain groups. (**A**). Bidimensional gel electrophoresis separation of the brain of *Agrotis ipsilon* showing about 1292 protein spots after a pre-migration in the pH range 5–8 (Colloidal Coomassie Blue G250). (**B**). Bidimensional gel electrophoresis separation of the brain of *Agrotis ipsilon* showing about 468 protein spots after a pre-migration in the pH range 6–9 (Colloidal Coomassie Blue G250). Numbers and colored spots were defined by the software in order to visually identify and number the spots presenting a difference in protein quantity between two conditions.

**Figure 3:**
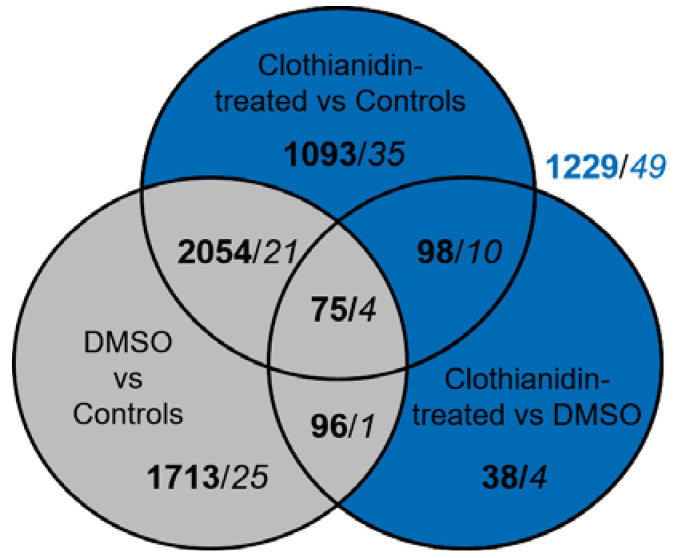
Differential expression analysis between the 3 experimental conditions for both transcriptomic and proteomic analysis. Venn diagram of transcriptomic and proteomic analyses of *A. ipsilon* brains in the 3 experimental conditions (controls, DMSO-treated, and clothianidin-treated). For transcriptomic results (values in bold), differentially expressed genes are indicated (FDR < 0.01). For proteomic results (values in italics), spots showing a significant difference in protein ratio values ≤−1.25 or ≥1.25 (Student *T*-Test) are indicated. The grey area indicates proteins/transcripts for which a DMSO effect was observed, while the blue area indicates a clothianidin effect. Blue numbers outside of the Venn diagram are the total numbers of transcripts and proteins for which the expression is modified following clothianidin exposure without the DMSO effect.

The transcriptomic analysis revealed that 3938 transcripts were deregulated after DMSO exposure compared to the control group (Table S2). Even though no significant effect of DMSO had been shown on flight activity and pheromone responses in our earlier study [17], the GO analysis of the genes showed that many biological processes were altered following DMSO exposure (Figure 1). The “small molecule metabolic process” category was enriched for the genes deregulated by DMSO while all the other categories were depleted, suggesting that DMSO was slowing down cell metabolism. DMSO also affected the brain proteome, as 51 protein spots presented a significant difference in intensity in the comparison between DMSO and control samples out of the 1760 that were detected after gel migration (Figure 2 and grey circle in Figure 3).

### 3.3. Clothianidin Exposure Affects Protein Biosynthesis and Metabolic Processes

In order to identify cellular processes related to clothianidin exposure only, we considered the transcripts and proteins that had their expression modified following the clothianidin exposure (in blue, Figure 3) and excluded transcripts and proteins showing a DMSO effect (in grey, Figure 3). By doing so, 1229 transcripts and 49 protein spots were identified and considered differentially expressed in relation with clothianidin exposure (in blue, Figure 3).

**Figure 4:**
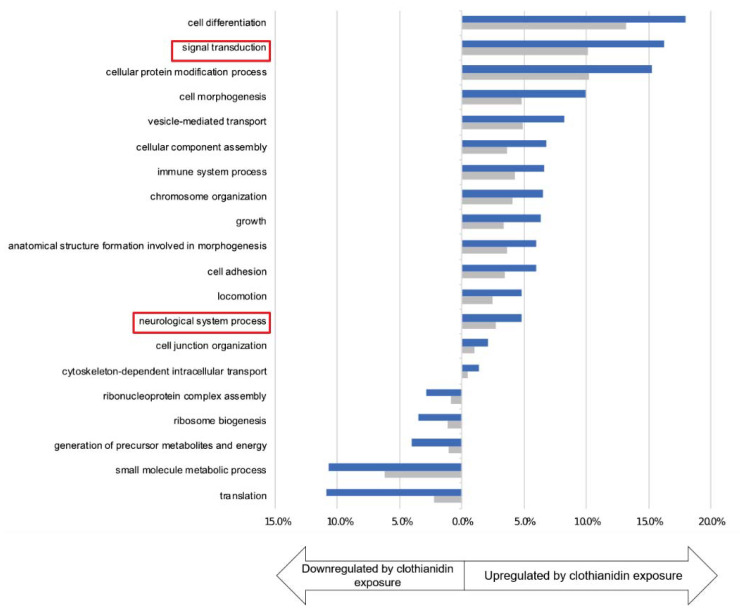
Exposure to clothianidin deregulates gene expression in the brain of *A. ipsilon*. Gene Ontology Enrichment analysis showing the GO terms of genes deregulated by clothianidin exposure compared to DMSO and controls (test set, blue) and that exhibit statistical significant differences with GO terms of to the whole transcriptome (reference set, grey) (Fisher exact test, FDR < 0.1). The *X*-axis shows the percentage of sequences in each dataset. The *Y*-axis shows the GO terms. Upregulated genes are shown on the right of the figure, downregulated genes on the left. The two categories that are relevant to our study, “signal transduction” and “neurological system process”, are highlighted with a red box.

Among these 1229 deregulated transcripts upon clothianidin exposure, 707 were upregulated while 522 were downregulated compared to both controls (control and DMSO, Table S3). Gene ontology enrichment analyses were performed on both categories of transcripts and revealed that clothianidin exposure led to (1) the downregulation of translation (GO categories “translation”, “ribosome biogenesis”, “ribonucleoprotein complex assembly” as well as “small molecule metabolic processes” and “generation of precursor metabolites and energy”) (Figure 4) and to (2) the upregulation of genes involved in particular in two categories that are relevant to our study: “signal transduction” and “neurological system process” (Figure 4).

**Figure 5:**
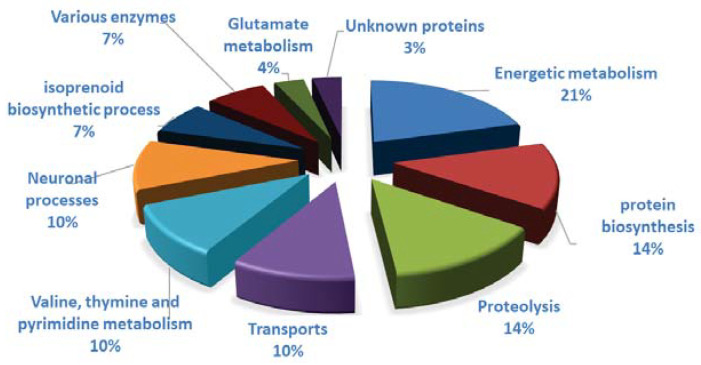
Functional categories of proteins displaying a change in their clothianidin/control level ratio. Proteins of control individuals with or without DMSO and clothianidin-treated individuals showing a significant variation in quantity were identified by LC-MS/MS procedures and classified into 10 functional categories. The name of the protein and its reference in the online database are indicated for each identified protein.

Among the 49 spots showing a difference in intensity due to clothianidin, 35 spots presented a significant difference of intensity in the comparison between clothianidin-treated and control samples and 4 in the comparison between clothianidin-treated and DMSO samples (Figure 3; *p* < 0.05, Student Test). Ten spots presented a significant difference of intensity in both comparisons. Out of these 49 spots, 27 proteins were identified by LC-MS/MS procedure and placed in groups based on their known function (Figure 5 and Table 2): 6 proteins belonged to energy metabolism; 4 proteins to protein biosynthesis; 4 proteins to proteolysis processes; 3 proteins to transport processes; 3 to valine, thymine, and pyrimidine metabolism; 2 to neuronal processes; 2 to isoprenoid biosynthetic processes; 2 to xenobiotic enzyme families; and 1 to glutamate metabolism. The peptide fragment of an unknown protein was recognized during the LC-MS/MS procedure, but no correspondence was found in online databases and none could be deduced from the brain transcriptome. Clothianidin treatment induced a significant decrease in the quantity of 21 of the identified proteins (from 17 to 63% in comparison to controls, Figure 5), mainly affecting energy metabolism, neuronal and isoprenoid biosynthetic processes, xenobiotic metabolizing enzymes, and glutamate metabolism. A significant increase in 6 proteins (from 17 to 32% in comparison to controls) was reported mainly in proteolysis and, to a lesser extent, in energy metabolism; transport; and valine, thymine, and pyrimidine metabolism.

### 3.4. Clothianidin Exposure Affects Detoxification Enzymes

Transcriptomic and proteomic analyses revealed that 10 genes and 2 proteins related to detoxification enzymes were regulated by the clothianidin treatment within *A. ipsilon* brains. Among them, one cytochrome P450 (CYPs) was upregulated, whereas five other CYPs, one Glutathione-S-transferase (GST), one aldo-keto reductase (AKR), and one carboxylesterase (CCE) were downregulated (Table 3 and Table S3). The quantity of two xenobiotic metabolizing enzymes also decreased following clothianidin exposure: one aldehyde dehydrogenase (ALDH) and a dimeric dihydrodiol dehydrogenase (Figure 5).

### 3.5. Clothianidin Exposure Affects Neuronal Processes

The transcriptomic analysis revealed significant differences in the expression of genes that are involved in synaptic function after clothianidin exposure (Table 4 and Table S3). Indeed, we observed an upregulation of the gene expression of a neurobeachin and synaptotagmin and a downregulation of the synaptic vesicle glycoprotein 2B. Other neuronal processes are also disrupted by clothianidin exposure according to the transcriptomic analysis (Table 4 and Table S3). Genes involved in the regulation of exchanges through the neuronal membrane have also their expression modified, such as the tyrosine-protein phosphatase 69D, or the flow of Ca^2+^ and/or Na^+^ through the activity of a transient receptor potential cation channel (trpm), a sodium channel protein (para type), a chloride channel, a voltage-dependent calcium channel (type A), or a sodium/calcium exchanger (type 3). For all these genes, we noted a significant upregulation of gene expression in insects exposed to clothianidin. Interestingly, we also observed the upregulation of other regulatory systems of neuronal activities (Table 4 and Table S3), such as, for example, the Gamma-aminobutyric acid (GABA) system. Proteomic analyses revealed that the quantities of three proteins involved in neuronal processes were decreased after clothianidin exposure: a N-ethylmaleimide sensitive fusion protein, also known as N-ethylmaleimide-sensitive factor (NSF); a Palmitoyl-protein Thioesterase 2 (PPT2); and a fatty alcohol acetyltransferase (FAA) (Figure 5).

## 4. Discussion

In this study, we showed that exposure to a low dose of clothianidin strongly modified gene and protein expression in the brain of *A. ipsilon* males. However, this is also the case for DMSO. DMSO is often used as a solvent or vehicle solution since this product is a good solvent for hydrophobic molecules such as clothianidin. The selection of appropriate vehicles or solvents to administer the compounds of interest is determinant for the quality of the results. There is now more and more evidence that solvents affect organisms. This also includes DMSO. Whether in vertebrates or invertebrates, DMSO effects can be deleterious, with the observation of some cases of cell toxicity [42], sterility [43], or neurotoxicity [44], but also beneficial, with some report of the neutralization or attenuation of pathologies [45] or with no observed effect [46]. Our results confirm that DMSO, a solvent used in many studies, even at very low doses, can influence gene and protein expression and that these effects need to be taken into account in differential studies.

After the exclusion of transcripts and proteins showing a DMSO effect, we thus identified 1229 transcripts and 49 protein spots considered differentially expressed in relation to clothianidin exposure. Interestingly, although we were able to identify common regulated categories such as neuronal processes using transcriptomics and proteomics, we did not identify the same genes/proteins. Additionally, genes of these categories were upregulated while protein levels were decreased following clothianidin exposure. While this may be surprising at a first glance, multiple studies showed that levels of mRNA and proteins are often not well correlated. This is due to numerous reasons, such as translational efficiency, mRNA structure, and stability [47] but also due to the difference in sensitivity between proteomics and transcriptomics methods. This work is another evidence to support the fact that these two high-throughput omics approaches are complementary for the identification of precise and complete pathways [48].

We have been able to find a wide variety of families of molecular actors and more specifically detoxification enzymes which show that clothianidin, after having crossed the digestive barrier, or its bio-transformed metabolites induce a detoxifying system response in the brain. All these enzyme super families are crucial in insects for their diverse roles on endogenous and both natural or synthetic exogenous compounds. In particular, insect CYPs, CCEs and GSTs play a prominent role in xenobiotic metabolism and many of them are involved in insecticide detoxification/resistance mechanisms [49]. Metabolic detoxification can be divided into phase I processes, consisting mainly of hydrolysis and oxidation reactions (by CYPs, AKRs or ALDHs), and phase II processes, involving the conjugation of phase I products with endogenous compounds (mainly by UGTs and GSTs), leading to the production of hydrophilic metabolites excreted outside the cells.

Neonicotinoids are complex molecules that could be metabolized by the combined action of various phase I and II enzymes [50,51]. The involvement of CYPs, CCEs, and GSTs in the neonicotinoid metabolism and/or neonicotinoid resistance in insects has been well documented [50,52]. The induction of these enzyme genes after neonicotinoid exposure and/or enhanced neonicotinoid detoxification associated with these enzymes were reported in various insect species, including *Drosophila melanogaster* [53], the honeybee *Apis mellifera* [54] and several pest insects such as *Bemisia tabaci* [55], *Bradysia odoriphaga* [56], *Leptinotarsa decemlineata* [57], *Sogatella furcifera* [58] and *Nilaparvata lugens* [58]. Our findings thus suggest that *A. ipsilon* possesses a small set of phase I and II enzymes, mainly CYPs, that could be modulated by clothianidin treatment. Only one P450 was induced by the insecticide, as a candidate gene involved in insecticide biotransformation within the *A. ipsilon* brain. All the others enzymes were down-regulated. We can suppose that the insecticide treatment may also disturb several enzymatic pathways, that could putatively interfere with the metabolism of exogenous or endogenous compounds.

Indeed, whereas less information is available on the role of AKR or ALDH in neonicotinoid metabolism, we know that AKRs are involved in the reduction in various aldehydes and ketones generated endogenously during metabolism or encountered in the environment as nutrients, drugs, or toxins (reviewed in [59]). Contrary to CYPs and CCEs, few AKRs have been functionally characterized in insects, and among them AKR2E4 has been shown to play a role in ecdysteroid metabolism (as 3-dehydroecdysone 3-beta-reductase in *S. littoralis* and *Bombyx mori* [60,61], whereas AKR2E5 is supposed to be also involved in *B. mori* pheromone metabolism [62]. Interestingly, *B. mori* AKR2E4 is induced (4.8 fold) by the organophosphate insecticide diazinon and could reduce various substrates in addition to 3-dehydroecdysone, suggesting a potential role both in steroid and xenobiotic metabolism [61]. More recently, a transcript coding for an AKR has been shown to be downregulated by chlorpyrifos exposure in *B. odoriphaga* [56]. ALDHs are involved in the oxidation of a broad range of endogenous compounds, such as biogenic amines, neurotransmitters and lipids. They also oxidize aldehyde intermediates resulting from xenobiotic and drug metabolism [63]. They are well studied for their role in ethanol metabolism in mammals and insects, converting the highly toxic intermediate acetaldehyde to acetate [64]. In the mosquito *Aedes aegypti*, ALDHs have been shown to detoxify pyrethroids, participating in insecticide resistance when up-regulated [65]. In the mammal brain, ALDH plays a crucial role by oxidizing the toxic dopamine aldehyde metabolite (DOPAL), thus protecting dopaminergic neurons [66]. It has been shown recently that brain ALDHs could be inhibited by various pesticides, leading to toxic aldehyde accumulation and dopaminergic cell death, a mechanism that could be linked to Parkinson’s disease pathogenesis [67]. Our results suggest that AKRs and ALDH may play a role in the behavioral and physiological effects of low neonicotinoid doses on *A. ipsilon*, even if the role of these enzymes has to be clarified in this species.

Significant differences in the expression of genes and proteins levels that are involved in synaptic function and neuronal processes were observed after clothianidin exposure in the brain of *A. ipsilon*. Whereas there is currently no study on the synaptic vesicle glycoprotein 2B in insects, neurobeachin was associated in *D. melanogaster* to neurodevelopmental disorders, disruption of synaptic properties and impaired behavior or associative learning [68]. Synaptotagmin appeared, again in *D. melanogaster*, to function as calcium sensor in the regulation of neurotransmitter release and hormone secretion [69]. Another gene, the neurocalcin homolog was also significantly upregulated in our study. Neurocalcin can also bind Ca^2+^ and is involved in the neuronal entry of Ca^2+^ [70]. Yet, it has never been shown in insects that the expression of these genes was modified by an insecticide treatment. In *D. melanogaster*, NSF interacts with other partners, such as the dysbindin to alter the vesicle fusion apparatus and therefore influence synaptic plasticity [71]. NSF plays also an important role in the synapse by binding neurexins, cell adhesion proteins that are involved in synaptogenesis, synaptic transmission, and synapse maintenance [72]. NSF was also demonstrated to modulate the synaptic vesicle mobility by interaction with F-actin [73]. Taken together, these results clearly show that exposure to clothianidin appears to disrupt the functioning of synapses and synaptic vesicles.

Some of the genes involved in the regulation of exchanges through the neuronal membrane were already studied in various insects exposed to insecticides belonging to the pyrethroid family [74]. The only study showing a variation of expression of a transient receptor potential cation channel (trpm) was realized in rat primary cortical neurons exposed to rotenone, an insecticide disrupting the energetic metabolism and inducing oxidative stress [75]. The authors observed an increase in the expression of the trpm2 isoform after exposure to rotenone, showing that there is a link between dysfunction of TRP channels, calcium dyshomeostasis and oxidative stress induced by insecticides.

Interestingly, we also observed the upregulation of the beta subunit of the Gamma-aminobutyric acid (GABA) receptor. The Gamma-aminobutyric acid (GABA) system is often associated to insecticide response since it appeared to be modulated from the reabsorption of the neurotransmitter (i.e., GABA) with a sodium- and chloride-dependent GABA transporter, to its reception by the GABA receptor, a receptor that may be involved in resistance to other insecticides, such as cyclodiene in *D. melanogaster* [76]. In fact, as we observed mainly an upregulation of genes involved in neuronal processes, we can hypothesize that this is the consequence of a general acclimatization of the neuronal system to a low lethal dose (LD_20_) of clothianidin. The upregulation of mushroom body large-type Kenyon cell-specific protein 1, Krueppel homolog 1, Octopamine receptor beta-3R, Glutamate-gated chloride channel or Neuropeptide CCHamide-2 receptor could also support this hypothesis. These actors are known to allow hormonal or neuropeptide modulation of neuronal activity, and they were previously described to be regulated by insecticide exposure [77,78]. Finally, some of our results also suggest that the low lethal dose exposure induced some neurogenesis or axonal growth, since we observed a significant increase in lachesin, an immunoglobulin superfamily protein whose expression correlates with neurogenesis [79] and of the SICKIE protein, which regulates F-actin mediated axonal growth in Drosophila mushroom body neurons [80].

Finally, we were able to observe effects on candidates still little studied but which potentially seem to play an important role in neuronal functioning. It is the case of PPT2 which is involved in the Palmitoylation cycle, a post-translation modification that can occur on secreted, cytoplasmic, and integral membrane proteins. PPT1 and PPT2 are the two primary thioesterases involved in removing palmitoyl groups during the lysosomal degradation process. While PPT1 is associated with neuronal disorders [81], little is known about the function of PPT2, despite its crucial role in the search for therapeutic solutions against neurodegenerative diseases in humans [82]. The difference of identity percentages between the two proteins and the inability of PPT2 to rescue the neural disruption associated with loss of PPT1 support distinct functions and substrates for these two thioesterases [83,84]. In insects, even less information is available on these proteins. In *D. melanogaster*, PPT1 plays a role in neuronal development and function [85,86]. However, the function of PPT2 remains elusive. Another interesting candidates is FAA which is often associated with sex pheromone biosynthesis, which takes place in pheromonal glands of insects. The FAA we found with our proteomic approach is very similar to the one found in a study on the role of enzymes in the pheromone biosynthesis in *Agrotis segetum* [87]. However, this FAA, as with the 33 other acetyltransferases found in the transcriptome of the pheromonal glands, appeared not to be involved in pheromone biosynthesis. Unfortunately, there is currently no information about the role of FAA in the nervous system in insects or vertebrates. Interestingly, this protein, as PPT proteins, was classified within a family of proteins having a palmitoyl-(protein) hydrolase activity (GO molecular function—UniProtKB—A0A088M9W1).

Whereas many genes/proteins involved in neuronal modulation were activated by the low dose of clothianidin, we did not find changes in the expression of the acetylcholine receptors themselves, the prime target for neonicotinoids such as clothianidin. Indeed, other ways of neuronal modulation were activated. However, we cannot conclude whether this large range of effects is due to a direct effect of clothianidin, or of its bio-transformed metabolites, or of a general response due to the stress induced by the insecticide exposure (i.e., oxidative stress, increase in metabolic cost…). Nevertheless, these neuronal changes could provide an explanatory element for the behavioral and neuronal sensitivity modifications that we observed previously in clothianidin-treated males [17,23,88].

The results of our study are consistent with the few studies showing anatomical or molecular modifications in the brain after the ingestion of pesticides in insects [24]. Studies on the capacity and limits of the digestive system to manage exposure to pesticides still need to progress since many processes remain unclear (passage of molecules, production of toxic metabolites, etc.). These effects highlight the fact that the digestive system does not represent a strict barrier to toxic compounds and that indeed insecticide effects occur in the brain after oral application. High-throughput RNAseq and proteomic analyses in our study showed a regulation of the expression of numerous enzymes as a possible detoxification response to the insecticide and also numerous changes in neuronal processes, which could act as a form of acclimatization to the insecticide-polluted environment. Functional studies will now be needed to investigate how up- or down-regulation of the differentially expressed genes and proteins might be involved in the behavioral and neuronal hormetic effects of increased sex pheromone responses in male *A. ipsilon*. An increased number of ecotoxicological studies, including the present one, now combine data from multiple omics techniques (Table 5). The complementarity of all these techniques permits more generally the identification of numerous targets that could be used for functional studies to disentangle the molecular mechanisms of pollutant effects on insects and other invertebrates.

## 5. Conclusions

Our study offers potential molecular explanations for the hormetic response that we previously observed in adult *A. ipsilon* males. Testing and understanding the effects of low doses is particularly important because these doses can have unexpected stimulating effects on pests, for which alternative management solutions are sought. In the context of integrated control measures, this information is therefore particularly crucial in order to avoid potentially counterproductive strategies (e.g., reduction in the quantities used) or to choose new alternative control methods.

## Figures and Tables

**Table 1:** *A. ipsilon* brain transcriptome assembly statistics.

	Diesner et al.	This Study
Number of contigs	50,750	17,986
Median contig length (nt)	618 bp	1233 bp
Complete BUSCOs	1062 (64.0%)	1462 (88.2%)
Complete and single-copy BUSCOs	891 (53.7%)	1381 (83.3%)
Complete and duplicated BUSCOs	171 (10.3%)	81 (4.9%)
Fragmented BUSCOs	309 (18.6%)	99 (6.0%)
Missing BUSCOs	287 (17.4%)	97 (5.8%)

**Table 2:** Characterization of differentially abundant proteins in the brain of *A. ipsilon* following clothianidin exposure.

Functional Categories	Protein Name	Uniprot Reference	Species	Peptide Number	*p* Value *	Protein Quantity in Comparison to Control (%)
Glutamate metabolism	Glutamate dehydrogenase	A0A0G3VFZ9_CHISP	*Chilo suppressalis*	4	0.009	−17.1509 ↘
Xenobiotic enzymes	Dimeric dihydrodiol dehydrogenase	S4P4A7_9NEOP	*Pararge aegeria*	8	0.026	−62.4242 ↘
	Aldehyde dehydrogenase X. mitochondrial-like	AL1B1_HUMAN	*Amyelois transitella*	6	0.019	−18.8077 ↘
Isoprenoid biosynthetic process	Geranylgeranyl diphosphate synthase	U3RD44_CHOFU	*Choristoneura fumiferana*	15	0.010	−21.7245 ↘
	Farnesyl diphosphate synthase 2	A5A7A5_BOMMO	*Bombyx mori*	7	0.026	−62.4242 ↘
Neuronal processes	Palmitoyl-protein Thioesterase 2	KGM_213554	*Danaus plexippus*	4	0.019	−18.8077 ↘
	Fatty alcohol acetyltransferase	A0A088M9K3_AGRSE	*Agrotis segetum*	13	0.006	−43.1842 ↘
	N-ethylmaleimide sensitive fusion protein	Q5G1P2_HELAM	*Helicoverpa armigera*	11	0.035	−15,9046 ↘
Valine. thymine and pyrimidine metabolism	Methylmalonate-semialdehyde dehydrogenase [acylating]. mitochondrial isoform X2	MMSA_DROME	*Papilio polytes*	2	0.019	−18.8077 ↘
	3-hydroxyisobutyryl-CoA hydrolase. mitochondrial	A0A068FL83_AGRSE	*Agrotis segetum*	7	0.026	−62.4242 ↘
	Dihydropyrimidinase isoform X2	A0A170WSE7_TRIIF	*Bombyx mori*	3	0.028	16.72897 ↗
Transport	Transferrin	A7IT76_SPOLT	*Spodoptera litura*	2	0.004	−18.3537 ↘
	Apolipophorins	APLP_DROME	*Papilio polytes*	3	0.028	16.72897 ↗
	Rabenosyn-5	A0A194QJD8_PAPXU	*Papilio xuthus*	6	0.019	−18.8077 ↘
Proteolysis	Protease m1 zinc metalloprotease	G6D6V6_DANPL	*Danaus plexippus*	23	0.017	28.09224 ↗
	Protease m1 zinc metalloprotease	G6D6V6_DANPL	*Danaus plexippus*	20	0.030	19.18782 ↗
	Mitochondrial processing peptidase beta subunit	I4DK27_PAPXU	*Papilio xuthus*	9	0.006	−43.1842 ↘
Protein biosynthesis	Elongation factor 1-a	L7WID6_SPOLT	*Spodoptera litura*	4	0.029	−23.5498 ↘
	Elongation factor 1 alpha	E0D8P8_LOCMI	*Locusta migratoria*	4	0.029	−23.5498 ↘
	UDP-N-acetylglucosamine-peptide N-acetylglucosaminyltransferase 110 kDa subunit	OGT1_HUMAN	*Bombyx mori*	2	0.013	−16.5289 ↘
	Translation initiation factor 2 gamma subunit	Q684K4_9NEOP	*Scoliopteryx libatrix*	3	0.029	−23.5498 ↘
Energy metabolism	ATP synthase subunit b. mitochondrial	A0A194PTU2_PAPXU	*Papilio xuthus*	2	0.034	−33.3046 ↘
	ATP synthase	Q2F5T4_BOMMO	*Bombyx mori*	5	0.020	−18.8077 ↘
	Aconitate hydratase. mitochondrial	Q86GF8_ANTYA	*Antheraea yamamai*	5	0.004	−18.3537 ↘
	Pyruvate kinase	H9IZ23_BOMMO	*Bombyx mori*	5	0.009	−17.1509 ↘
	NADH-ubiquinone oxidoreductase 75 kDa subunit. mitochondrial	NDUS1_DROME	*Bombyx mori*	28	0.028	16.72897 ↗
	UDP-glucose pyrophosphorylase	A0A0S1MMM3_ANTPE	*Antheraea pernyi*	5	0.009	−17.1509 ↘
Unknown	unknown			17	0.003	−28.2986 ↘

Legends: ↗ more abundant in clothianidin-treated group compared to controls; ↘ less abundant in clothianidin-treated group compared to controls; * *p* value of the Student’s *t*-test analysis.

**Table 3:** Clothianidin exposure affects the transcript abundance of detoxification enzymes.

*	Transcript ID	logFC Clothianidin vs. DMSO	logFC Clothianidin vs. Control	Annotation by Blast Research
Up	DN9632_c0_g1_i1	1.35	1.13	Cytochrome P450 4C1
	DN9385_c0_g1_i1	0.78		UDP-glucuronosyltransferase 2B7
Down	DN27963_c0_g1_i1		−1.49	Probable cytochrome P450 6a13
	DN20937_c0_g1_i1		−1.42	Cytochrome P450 4c3
	DN37703_c0_g1_i1		−1.16	Cytochrome P450 6B7
	DN34383_c0_g1_i1		−0.96	Cytochrome P450 4C1
	DN20657_c0_g1_i1		−0.69	Aldo-keto reductase AKR2E4
	DN5740_c0_g1_i1		−0.63	Glutathione S-transferase 1
	DN4625_c0_g1_i1		−0.35	Cytochrome P450 CYP12A2
	DN4767_c0_g1_i1		−0.50	Venom carboxylesterase-6

* Up- or downregulated following clothianidin exposure.

**Table 4:** Clothianidin exposure affects the transcript abundance of neuronal processes.

GO Function	*	Transcript ID	logFC Clothianidin vs. DMSO	logFC Clothianidin vs. Control	Annotation by Blast Research
Synaptic function	Up	DN6306_c0_g1_i1		0.89	Neurobeachin
		DN2580_c0_g1_i1		0.42	Synaptotagmin 1
		DN4891_c0_g1_i1		0.33	Neurocalcin homolog
	Down	DN10953_c0_g1_i1		−0.53	Synaptic vesicle glycoprotein 2B
Exchanges across the neuronal membrane	Up	DN21572_c0_g1_i1		1.14	Tyrosine-protein phosphatase 69D
		DN19704_c0_g1_i1		0.46	Transient receptor potential cation channel trpm
		DN6537_c0_g1_i1		0.56	Sodium channel protein para
		DN12028_c0_g1_i1		0.53	Chloride channel protein 2
		DN27405_c0_g1_i1		1.21	Voltage-dependent calcium channel type D subunit alpha-1
		DN1850_c0_g1_i1	1.31	1.59	Sodium/calcium exchanger 3
Neuronal activity	Up	DN6443_c0_g1_i1		0.49	Sodium- and chloride-dependent GABA transporter 1
	Down	DN392_c0_g1_i1		−0.31	Sodium- and chloride-dependent GABA transporter ine
Neuronal processes	Up	DN20357_c0_g1_i1		0.64	Mushroom body large-type Kenyon cell-specific protein 1
		DN7325_c0_g1_i1		0.96	Krueppel homolog 1
		DN25883_c0_g1_i1		1.36	Octopamine receptor beta-3R
		DN49002_c0_g1_i1	0.84		Glutamate-gated chloride channel
		DN18143_c0_g1_i1		0.48	Neuropeptide CCHamide-2 receptor
Neurogenesis/axonal growth	Up	DN33688_c0_g1_i1		0.88	Lachesin
		DN10822_c0_g1_i1		0.49	Protein sickie

* Up- or downregulated expression following clothianidin exposure.

**Table 5:** Ecotoxicological studies using transcriptomics and proteomics methods.

Proteins				Transcripts					
Number of Detected Spots or Proteins	Proteins Showing Significant Variation in Quantity	Up	Down	Number of Analyzed Unigenes	Up	Down	Molecules or Condition	Species	References
1760	49	6	23	17,986	2292	1646	Clothianidin	*Agrotis ipsilon*	Present study
700	12	nc	nc	nc	41	56	Imidacloprid	*Mytilus galloprovincialis*	[20]
				nc	43	37	Thiacloprid		
				nc	26	23	Mix		
				29,146 to 31,467	646 to 658	284 to 533	Clothianidin	*Bradysia odoriphaga*	[66]
				35,222	349	271	Thiamethoxam	*Aphis gossypii*	[89]
				11,150 to 11,426	225	384	Thiamethoxam	*Apis mellifera*	[64]
1005	52	38	14	nc	664	674	Thiamethoxam	*Bemisia tabaci*	[65]
821	143	35	108				Mesoionic pyrido[1,2-]pyrimidinone	*Aphis craccivora*	[90]
>1300	28	14	14				resistant strain to imidacloprid	*Myzus persicae*	[91]
1470	155	96	59				Nicotine	*Apis mellifera*	[92]

## Data Availability

Data available on request due to restrictions eg privacy or ethical.

## Supplementary Materials

The following are available online at https://www.mdpi.com/2075-4450/12/2/152/s1: Supplementary Data 1. Fragment counts were used to build a correlation matrix between all conditions of the experiments to perform a quality check. The biological replicates DMSO1, clothianidin2, and Control3 did not correlate with the other biological replicates of their exposure groups and were removed from subsequent analysis (A). After the removal of these three samples, the two remaining biological replicates for each exposure group are correlated to each other (B). Table S1: Proteomics results for the two pH migrations. The spots for which the ANOVA result is <0.05 and the ratio is ≥1.3 were coloured in green for the CTRL/Clothia and Clothia/DMSO comparisons and in red for the CTRL/DMSO comparison. Table S2: List of deregulated genes following DMSO exposure. Table S3: List of deregulated genes following clothianidin exposure.
