# Supplementary material for: Sublethal Exposure Effects of the Neonicotinoid Clothianidin Strongly Modify the Brain Transcriptome and Proteome in the Male Moth Agrotis ipsilon
Source: Insects. 2021 Feb 11;12(2):152. doi: 10.3390/insects12020152 (PMC7916958; doi:10.3390/insects12020152)
Supplement: Supplementary file 1 [file insects-12-00152-s001.tgz › Table S1.pdf]

		CTRL / DMSO		CTRL / Clothia		Clothia / DMSO						Average Normalised Volumes			
**Spot name**	**Rank**	**Anova (p)**	**Ratio**	**Anova (p)**	**Ratio**	**Anova (p)**	**Ratio**	**Protein Description**	**Nb Uniq Pept**	**UniProt ID**	**UniProt Description**	**CTRL**	**Clothia**	**DMSO**	**% variation Clothia/DMSO**
eID: 0001	**1**	0,89033536	0,997	0,61926	0,985	0,61082347	1,012					1,075	1,091	1,078	1,2059
eID: 0002	**2**	0,4436591	1,039	0,08824	1,081	0,43050151	0,961					1,018	0,942	0,98	-3,8776
eID: 0003	**3**	0,63375904	1,211	0,01376	3,223	0,02688683	0,376	Agrotis_qualite_rep_c519|ORF3 length 394 aa, 1185 bp, from 77..1261 of	7	A5A7A5_BOMMO	Farnesyl diphosphate synthase 2 (Bombyx mori)	1,199	0,372	0,99	-62,4242
								Agrotis_qualite_rep_c6539|ORF3 length 373 aa, 1122 bp, from complement(168..1289) of	8	S4P4A7_9NEOP	Dimeric dihydrodiol dehydrogenase Pararge aegeria (specked wood butterfly)	1,199	0,372	0,99	-62,4242
								Agrotis_qualite_c15729|ORF8 length 374 aa, 1125 bp, from 219..1343 of Agrotis_qualite_c15729	7	A0A068FL83_AGRSE	3-hydroxyisobutyryl-CoA hydrolase, mitochondrial Agrotis segetum (Turnip moth)	1,199	0,372	0,99	-62,4242
								Agrotis_qualite_rep_c14255|ORF4 length 436 aa, 1311 bp, from 140..1450 of	6			1,199	0,372	0,99	-62,4242
								Agrotis_qualite_c15383|ORF2 length 389 aa, 1170 bp, from complement(1..1170) of Agrotis_qualite_c15383	3			1,199	0,372	0,99	-62,4242
								Agrotis_qualite_rep_c15638|ORF10 length 275 aa, 828 bp, from 402..1229 of	3			1,199	0,372	0,99	-62,4242
								Agrotis_qualite_rep_c140|ORF19 length 340 aa, 1023 bp, from complement(773..1795) of	2			1,199	0,372	0,99	-62,4242
								Agrotis_qualite_rep_c317|ORF5 length 381 aa, 1146 bp, from 3..1148 of	2			1,199	0,372	0,99	-62,4242
eID: 0004	**4**	0,60003412	0,976	0,64722	0,974	0,9814176	1,002					1,077	1,106	1,104	0,1812
eID: 0005	**5**	0,35262616	1,199	0,01678	1,679	0,30450237	0,714					1,261	0,751	1,052	-28,6122
eID: 0006	**6**	0,21746936	0,884	0,27553	0,661	0,4274693	1,337					0,854	1,292	0,966	33,7474

eID: 0007	**7**	0,03462989	0,699	0,00115	0,556	0,07443111	1,257					0,716	1,287	1,024	25,6836
eID: 0008	**8**	0,23221473	1,132	0,30712	1,168	0,67088282	0,969					1,203	1,030	1,063	-3,1044
eID: 0010	**9**	0,32317202	1,165	0,09909	0,773	0,02792942	1,508					1,124	1,455	0,965	50,7772
eID: 0011	**10**	0,48670689	0,919	0,00474	0,679	0,02562287	1,353					1,000	1,472	1,088	35,2941
eID: 0015	**11**	0,5989982	1,057	0,02193	1,585	0,03780286	0,667	Agrotis_qualite_rep_c28604|ORF3 length 250 aa, 753 bp, from 71..823 of	2	NP_001299400.1	ATP synthase subunit b, mitochondrial [Papilio xuthus]	1,225	0,773	1,159	-33,3046
eID: 0016	**12**	0,03619917	0,783	0,782	1,021	0,06501924	0,767					0,922	0,903	1,178	-23,3447
eID: 0017	**13**	0,00846806	1,358	0,09171	1,150	0,12937819	1,181					0,982	0,854	0,723	18,1189
eID: 0020	**14**	0,02207264	0,776	0,01386	0,713	0,33013352	1,089					0,796	1,117	1,026	8,8694
eID: 0022	**15**	0,99089899	1,013	0,44092	1,128	0,29903545	0,898					1,085	0,962	1,071	-10,1774
eID: 0024	**16**	0,99251586	0,997	0,72074	0,969	0,74310694	1,029					1,059	1,093	1,062	2,9190
eID: 0025	**17**	2,23E-01	0,923	6,88E-05	0,791	2,83E-02	1,167	Agrotis_qualite_c17211|ORF18 length 470 aa, 1413 bp, from complement(833..2245) of	28	NDUS1_DROME	NADH-ubiquinone oxidoreductase 75 kDa subunit, mitochondrial [Bombyx mori]	0,988	1,249	1,070	16,7290
								Agrotis_qualite_c17211|ORF9 length 208 aa, 627 bp, from complement(388..1014) of Agrotis_qualite_c17211	7	NDUS1_DROME	NADH-ubiquinone oxidoreductase 75 kDa subunit, mitochondrial [Bombyx mori]	0,988	1,249	1,070	16,7290
								Agrotis_qualite_c12455|ORF29 length 600 aa, 1803 bp, from complement(2027..3829) of	3	DPYL2_HUMAN	dihydropyrimidinase isoform X2 [Bombyx mori]	0,988	1,249	1,070	16,7290
								Agrotis_qualite_rep_c4225|ORF4 length 1058 aa, 3177 bp, from 96..3272 of	3	APLP_DROME	apolipophorins [Papilio polytes]	0,988	1,249	1,070	16,7290
eID: 0027	**18**	0,09932759	1,263	0,11693	1,194	0,60641263	1,057					1,341	1,123	1,062	5,7439
eID: 0028	**19**	0,05039137	1,212	0,53784	1,055	0,05286216	1,149					0,942	0,893	0,777	14,9292
eID: 0029	**20**	0,33945971	1,072	0,85736	0,970	0,40747064	1,105					1,034	1,066	0,965	10,4663
eID: 0030	**21**	0,01714701	1,235	0,75559	0,964	0,02983468	1,281					1,126	1,168	0,912	28,0702
eID: 0031	**22**	0,01949402	1,161	0,00437	1,290	0,10345876	0,900					1,167	0,905	1,005	-9,9502
eID: 0032	**23**	0,00185864	1,157	0,11056	1,141	0,91344361	1,014					1,276	1,118	1,103	1,3599
eID: 0039	**24**	0,14008745	0,951	0,01556	0,863	0,04970252	1,102					0,972	1,126	1,022	10,1761
eID: 0040	**25**	0,16036019	0,939	0,98125	1,000	0,2144648	0,939					0,953	0,953	1,015	-6,1084
eID: 0042	**26**	0,75938199	0,985	0,15194	1,095	0,12865699	0,899					1,021	0,932	1,037	-10,1254
eID: 0043	**27**	0,21197116	0,963	0,02747	0,919	0,02349697	1,047					1,039	1,130	1,079	4,7266
eID: 0045	**28**	0,1738831	0,731	0,01235	0,653	0,47882544	1,119					0,714	1,093	0,977	11,8731
eID: 0047	**29**	0,82885811	0,941	0,18413	1,207	0,21820327	0,780					0,999	0,828	1,062	-22,0339
eID: 0048	**30**	0,01801866	0,793	0,43841	0,768	0,84395385	1,032					1,170	1,523	1,476	3,1843

eID: 0049	**31**	0,00220013	0,781	0,24105	0,825	0,54484252	0,947					1,043	1,265	1,336	-5,3144
eID: 0050	**32**	0,36532416	0,893	0,39027	0,897	0,98831644	0,996					0,854	0,952	0,956	-0,4184
eID: 0051	**33**	0,69207625	1,154	0,30042	1,718	0,55656224	0,672					0,426	0,248	0,369	-32,7913
eID: 0052	**34**	0,82404497	1,147	0,21701	3,138	0,28343448	0,366					0,273	0,087	0,238	-63,4454
eID: 0053	**35**	0,77923154	1,329	0,18114	3,802	0,21010787	0,350					0,327	0,086	0,246	-65,0407
eID: 0054	**36**	0,68678265	1,405	0,28098	2,800	0,44227826	0,502					0,406	0,145	0,289	-49,8270
eID: 0055	**37**	0,37846961	1,397	0,03575	2,624	0,24014557	0,532					0,412	0,157	0,295	-46,7797
eID: 0056	**38**	0,15204884	1,414	0,0315	1,795	0,36069435	0,787					0,632	0,352	0,447	-21,2528
eID: 0057	**39**	0,42999924	0,893	0,54996	0,382	0,63534115	2,336					1,299	3,399	1,455	133,6082
eID: 0058	**40**	0,18025057	1,529	0,05698	2,120	0,31982216	0,721					1,497	0,706	0,979	-27,8856
eID: 0059	**41**	0,23564757	1,361	0,00115	2,929	0,02473472	0,465					0,697	0,238	0,512	-53,5156
eID: 0061	**42**	0,04810485	1,686	0,00319	1,927	0,78993825	0,875					1,590	0,825	0,943	-12,5133
eID: 0062	**43**	0,4146107	1,134	0,07393	1,420	0,15068624	0,799					0,873	0,615	0,77	-20,1299
eID: 0063	**44**	0,32943259	1,207	0,05719	1,667	0,32123486	0,724					1,080	0,648	0,895	-27,5978
eID: 0064	**45**	0,34657773	1,230	0,06018	1,579	0,12017909	0,779					1,255	0,795	1,020	-22,0588
eID: 0066	**46**	0,7134144	1,051	0,44091	1,120	0,68899033	0,938					1,120	1,000	1,066	-6,1914
ID: 0046	**47**	0,60254582	0,892	0,97372	0,966	0,63965043	0,923					0,604	0,625	0,677	-7,6809
ID: 0076	**48**	0,82221529	1,032	0,45814	1,156	0,52133084	0,893					0,712	0,616	0,69	-10,7246
ID: 0080	**49**	0,19606268	0,824	0,02755	0,722	0,20372408	1,141					0,817	1,132	0,992	14,1129
ID: 0082	**50**	0,21999725	0,839	0,05929	0,752	0,27069941	1,115					0,81	1,077	0,966	11,4907
ID: 0084	**51**	0,21839722	0,842	0,03766	0,670	0,15995092	1,255					0,807	1,204	0,959	25,5474
ID: 0085	**52**	0,82157554	0,986	0,60105	1,218	0,36979223	0,809					0,993	0,815	1,007	-19,0665
ID: 0087	**53**	0,84378076	0,950	0,83598	1,063	0,67318066	0,894					0,982	0,924	1,034	-10,6383
ID: 0089	**54**	0,99695229	1,011	0,81891	1,056	0,79159708	0,958					1,060	1,004	1,048	-4,1985
ID: 0090	**55**	0,84909171	0,929	0,79701	1,080	0,65031895	0,861					1,027	0,951	1,105	-13,9367
ID: 0091	**56**	0,82576294	0,895	0,97128	0,968	0,86163303	0,925					0,933	0,964	1,042	-7,4856
ID: 0092	**57**	0,91193862	0,945	0,73737	1,115	0,65325963	0,847					0,978	0,877	1,035	-15,2657
ID: 0093	**58**	0,54050852	0,838	0,84599	0,903	0,74111953	0,928					0,967	1,071	1,154	-7,1924
ID: 0094	**59**	0,15543903	0,556	0,08993	0,455	0,61199194	1,221					0,568	1,247	1,021	22,1352
ID: 0095	**60**	0,06057105	0,653	0,07267	0,668	0,94031064	0,977					0,602	0,901	0,922	-2,2777
ID: 0097	**61**	0,6731135	1,104	0,38398	1,288	0,51912498	0,857					1,069	0,83	0,968	-14,2562
ID: 0103	**62**	0,3853972	0,839	0,4125	0,849	0,98177556	0,988					0,948	1,116	1,130	-1,2389
ID: 0104	**63**	0,34706699	1,110	0,31983	1,156	0,76207883	0,960					0,756	0,654	0,681	-3,9648
ID: 0105	**64**	0,29411215	1,130	0,12523	1,464	0,20238853	0,772					0,953	0,651	0,843	-22,7758
ID: 0110	**65**	0,85310498	0,989	0,81683	0,971	0,91965621	1,018					0,983	1,012	0,994	1,8109
ID: 0112	**66**	0,81145712	0,952	0,99326	1,027	0,77252912	0,927					0,991	0,965	1,041	-7,3007
ID: 0113	**67**	0,76735672	0,950	0,81919	0,949	0,97634352	1,001					0,988	1,041	1,040	0,0962
ID: 0114	**68**	0,45551373	0,872	0,66742	0,941	0,647126	0,926					0,917	0,974	1,052	-7,4144
ID: 0116	**69**	0,23831773	0,785	0,6877	0,970	0,28381912	0,809					0,877	0,904	1,117	-19,0689
ID: 0117	**70**	0,16267938	0,711	0,24467	0,829	0,52131932	0,858					0,863	1,041	1,213	-14,1797

ID: 0118	**71**	0,90266349	1,006	0,3587	1,082	0,35507799	0,930					0,913	0,844	0,908	-7,0485
ID: 0119	**72**	0,63497491	0,970	0,93936	1,017	0,33624695	0,955					0,855	0,841	0,881	-4,5403
ID: 0121	**73**	0,37972903	0,924	0,44017	0,934	0,89701021	0,989					0,861	0,922	0,932	-1,0730
ID: 0122	**74**	0,35485037	0,942	0,87508	1,013	0,28237036	0,930					0,87	0,859	0,924	-7,0346
ID: 0123	**75**	0,157635	0,923	0,74245	0,977	0,35947728	0,945					0,927	0,949	1,004	-5,4781
ID: 0125	**76**	0,0799507	0,822	0,38245	1,158	0,07323137	0,709					0,893	0,771	1,087	-29,0708
ID: 0126	**77**	0,73279243	0,966	0,3537	0,907	0,50178164	1,065					0,958	1,056	0,992	6,4516
ID: 0127	**78**	0,39802515	0,911	0,58435	0,947	0,75997492	0,962					0,879	0,928	0,965	-3,8342
ID: 0128	**79**	0,74113375	1,038	0,31323	1,197	0,51798272	0,867					0,968	0,809	0,933	-13,2905
ID: 0131	**80**	0,9347956	1,017	0,54093	0,945	0,29300604	1,077					0,89	0,942	0,875	7,6571
ID: 0132	**81**	0,7324213	1,047	0,73702	1,051	0,9480816	0,996					0,987	0,939	0,943	-0,4242
ID: 0133	**82**	0,98471048	1,010	0,94118	1,016	0,93327156	0,995					0,972	0,957	0,962	-0,5198
ID: 0136	**83**	0,95874889	0,949	0,18612	1,302	0,2389322	0,729					0,974	0,748	1,026	-27,0955
ID: 0137	**84**	0,68816886	0,930	0,53115	0,935	0,92811674	0,995					0,971	1,039	1,044	-0,4789
ID: 0139	**85**	0,84000239	1,012	0,62963	0,974	0,53752246	1,038					1,134	1,164	1,121	3,8359
ID: 0140	**86**	0,14922904	0,767	0,03387	0,643	0,11111091	1,193					0,795	1,236	1,036	19,3050
ID: 0143	**87**	0,52313073	1,062	0,19345	1,317	0,28520447	0,806					0,914	0,694	0,861	-19,3961
ID: 0144	**88**	0,8512961	1,009	0,80345	0,979	0,72110328	1,031					1,085	1,108	1,075	3,0698
ID: 0145	**89**	0,08744307	0,764	0,03084	0,722	0,52152451	1,058					0,814	1,128	1,066	5,8161
ID: 0146	**90**	0,64177071	0,965	0,45186	0,942	0,64363965	1,024					1,065	1,131	1,104	2,4457
ID: 0147	**91**	0,11888826	0,812	0,04403	0,696	0,12579877	1,167					0,834	1,198	1,027	16,6504
ID: 0148	**92**	0,48458174	0,907	0,39749	0,934	0,89551905	0,971					0,752	0,805	0,829	-2,8951
ID: 0149	**93**	0,85823104	1,025	0,49924	0,935	0,30840521	1,096					1,064	1,138	1,038	9,6339
ID: 0150	**94**	0,19863799	0,835	0,03036	0,712	0,10407947	1,174					0,918	1,290	1,099	17,3794
ID: 0151	**95**	0,33537066	0,917	0,1851	0,844	0,41422491	1,086					0,933	1,106	1,018	8,6444
ID: 0152	**96**	0,99725227	1,027	0,76794	0,970	0,54734701	1,059					1,030	1,062	1,003	5,8824
ID: 0153	**97**	0,42685322	1,119	0,13147	1,244	0,67341781	0,900					1,059	0,851	0,946	-10,0423
ID: 0154	**98**	0,24013409	0,868	0,06078	0,742	0,07130598	1,171					0,799	1,077	0,92	17,0652
ID: 0155	**99**	0,22095972	0,840	0,15462	0,788	0,27645561	1,066					0,819	1,039	0,975	6,5641
ID: 0157	**100**	0,25156972	0,860	0,25423	0,861	0,99022336	0,999					1,109	1,288	1,289	-0,0776
ID: 0158	**101**	0,83216779	1,002	0,71988	1,036	0,93676008	0,967					1,054	1,017	1,052	-3,3270
ID: 0159	**102**	0,12921189	0,758	0,02937	0,660	0,23773833	1,148					0,789	1,195	1,041	14,7935
ID: 0160	**103**	0,16763555	0,833	0,03862	0,679	0,11017665	1,226					0,673	0,991	0,808	22,6485
ID: 0162	**104**	0,48626021	1,114	0,39409	1,152	0,93528921	0,967					0,939	0,815	0,843	-3,3215
ID: 0163	**105**	0,22239877	0,794	0,00179	0,684	0,26381816	1,161					0,771	1,127	0,971	16,0659
ID: 0164	**106**	0,41437722	0,892	0,42314	0,888	0,9546227	1,005					0,75	0,845	0,841	0,4756
ID: 0165	**107**	0,76949313	0,935	0,37921	0,866	0,52499487	1,080					1,037	1,198	1,109	8,0252
ID: 0167	**108**	0,49169558	0,881	0,35759	0,793	0,64846566	1,111					0,761	0,96	0,864	11,1111
ID: 0168	**109**	0,38463157	1,134	0,17679	1,326	0,50265494	0,855					1,142	0,861	1,007	-14,4985
ID: 0170	**110**	0,18205971	0,847	0,07334	0,837	0,83693799	1,012					0,898	1,073	1,060	1,2264

<table>
<tr><td>ID: 0171</td><td>111</td><td>0,52628183</td><td>1,107</td><td>0,32337</td><td>1,260</td><td>0,63227902</td><td>0,879</td><td></td><td></td><td></td><td></td><td>1,115</td><td>0,885</td><td>1,007</td><td>-12,1152</td></tr>
<tr><td>ID: 0172</td><td>112</td><td>0,70595654</td><td>1,037</td><td>0,19828</td><td>0,871</td><td>0,18574503</td><td>1,190</td><td></td><td></td><td></td><td></td><td>1,046</td><td>1,201</td><td>1,009</td><td>19,0287</td></tr>
<tr><td>ID: 0173</td><td>113</td><td>0,606063</td><td>0,934</td><td>0,11658</td><td>0,864</td><td>0,46162721</td><td>1,081</td><td></td><td></td><td></td><td></td><td>0,893</td><td>1,033</td><td>0,956</td><td>8,0544</td></tr>
<tr><td>ID: 0175</td><td>114</td><td>0,3361382</td><td>0,930</td><td>0,15116</td><td>0,851</td><td>0,34766238</td><td>1,094</td><td></td><td></td><td></td><td></td><td>0,923</td><td>1,085</td><td>0,992</td><td>9,3750</td></tr>
<tr><td>ID: 0176</td><td>115</td><td>0,51419087</td><td>1,109</td><td>0,20776</td><td>1,395</td><td>0,47455144</td><td>0,795</td><td></td><td></td><td></td><td></td><td>1,152</td><td>0,826</td><td>1,039</td><td>-20,5005</td></tr>
<tr><td rowspan="3">ID: 0177</td><td rowspan="3">116</td><td rowspan="3">0,0820049</td><td rowspan="3">0,856</td><td rowspan="3">0,00465</td><td rowspan="3">0,718</td><td rowspan="3">0,03076929</td><td rowspan="3">1,192</td><td>Agrotis_qualite_rep_c514|ORF4 length 662 aa, 1989 bp, from complement(37..2025) of Agrotis_qualite_rep_c514</td><td>23</td><td>G6D6V6_DANPL</td><td>Protease m1 zinc metalloprotease Danaus plexippus (Monarch butterfly)</td><td>0,843</td><td>1,174</td><td>0,985</td><td>19,1878</td></tr>
<tr><td>Agrotis_qualite_rep_c3329|ORF3 length 247 aa, 744 bp, from 3..746 of</td><td>7</td><td>G6D6V5_DANPL</td><td>Protease m1 zinc metalloprotease Danaus plexippus (Monarch</td><td>0,843</td><td>1,174</td><td>0,985</td><td>19,1878</td></tr>
<tr><td>Agrotis_qualite_rep_c1757|ORF25 length 189 aa, 570 bp, from 1081..1650 of</td><td>4</td><td></td><td></td><td>0,843</td><td>1,174</td><td>0,985</td><td>19,1878</td></tr>
<tr><td>ID: 0178</td><td>117</td><td>0,95754863</td><td>1,001</td><td>0,93081</td><td>1,002</td><td>0,96679105</td><td>0,999</td><td></td><td></td><td></td><td></td><td>1,017</td><td>1,015</td><td>1,016</td><td>-0,0984</td></tr>
<tr><td>ID: 0179</td><td>118</td><td>0,96741056</td><td>0,997</td><td>0,66075</td><td>0,955</td><td>0,6741835</td><td>1,044</td><td></td><td></td><td></td><td></td><td>1,045</td><td>1,094</td><td>1,048</td><td>4,3893</td></tr>
<tr><td>ID: 0180</td><td>119</td><td>0,04205988</td><td>0,826</td><td>0,04375</td><td>0,718</td><td>0,20016193</td><td>1,151</td><td></td><td></td><td></td><td></td><td>0,862</td><td>1,201</td><td>1,043</td><td>15,1486</td></tr>
<tr><td>ID: 0181</td><td>120</td><td>0,33828818</td><td>0,877</td><td>0,04786</td><td>0,755</td><td>0,11089917</td><td>1,162</td><td></td><td></td><td></td><td></td><td>0,909</td><td>1,204</td><td>1,036</td><td>16,2162</td></tr>
<tr><td>ID: 0183</td><td>121</td><td>0,85625619</td><td>1,020</td><td>0,46824</td><td>1,096</td><td>0,4229656</td><td>0,931</td><td></td><td></td><td></td><td></td><td>1,047</td><td>0,955</td><td>1,026</td><td>-6,9201</td></tr>
<tr><td>ID: 0184</td><td>122</td><td>0,88616693</td><td>0,991</td><td>0,25991</td><td>0,890</td><td>0,22150019</td><td>1,113</td><td></td><td></td><td></td><td></td><td>0,902</td><td>1,013</td><td>0,91</td><td>11,3187</td></tr>
<tr><td>ID: 0185</td><td>123</td><td>0,26315739</td><td>1,200</td><td>0,15476</td><td>1,498</td><td>0,36026172</td><td>0,801</td><td></td><td></td><td></td><td></td><td>1,164</td><td>0,777</td><td>0,97</td><td>-19,8969</td></tr>
<tr><td>ID: 0186</td><td>124</td><td>0,69801202</td><td>1,037</td><td>0,25491</td><td>1,124</td><td>0,55791013</td><td>0,922</td><td></td><td></td><td></td><td></td><td>1,042</td><td>0,927</td><td>1,005</td><td>-7,7612</td></tr>
<tr><td rowspan="4">ID: 0187</td><td rowspan="4">125</td><td rowspan="4">0,01342831</td><td rowspan="4">0,785</td><td rowspan="4">0,00267</td><td rowspan="4">0,594</td><td rowspan="4">0,03594707</td><td rowspan="4">1,322</td><td>Agrotis_qualite_rep_c514|ORF4 length 662 aa, 1989 bp, from complement(37..2025) of Agrotis_qualite_rep_c514</td><td>20</td><td>G6D6V6_DANPL</td><td>Protease m1 zinc metalloprotease Danaus plexippus (Monarch butterfly)</td><td>0,741</td><td>1,248</td><td>0,944</td><td>32,2034</td></tr>
<tr><td>Agrotis_qualite_rep_c3329|ORF3 length 247 aa, 744 bp, from 3..746 of</td><td>5</td><td>G6D6V5_DANPL</td><td>Protease m1 zinc metalloprotease Danaus plexippus (Monarch</td><td>0,741</td><td>1,248</td><td>0,944</td><td>32,2034</td></tr>
<tr><td>Agrotis_qualite_rep_c1757|ORF25 length 189 aa, 570 bp, from 1081..1650 of</td><td>5</td><td></td><td></td><td>0,741</td><td>1,248</td><td>0,944</td><td>32,2034</td></tr>
<tr><td>Agrotis_qualite_rep_c659|ORF23 length 505 aa, 1518 bp, from 992..2509 of</td><td>2</td><td></td><td></td><td>0,741</td><td>1,248</td><td>0,944</td><td>32,2034</td></tr>
<tr><td>ID: 0188</td><td>126</td><td>0,21088993</td><td>1,239</td><td>0,06867</td><td>1,507</td><td>0,39300591</td><td>0,822</td><td></td><td></td><td></td><td></td><td>1,209</td><td>0,802</td><td>0,976</td><td>-17,8279</td></tr>
</table>

ID: 0189	**127**	0,10323753	0,869	0,00029	0,678	0,0171166	1,281	Agrotis_qualite_rep_c514|ORF4 length 662 aa, 1989 bp, from complement(37..2025) of Agrotis_qualite_rep_c514	9	G6D6V6_DANPL	Protease m1 zinc metalloprotease Danaus plexippus (Monarch butterfly)	0,829	1,222	0,954	28,0922
								Agrotis_qualite_rep_c3329|ORF3 length 247 aa, 744 bp, from 3..746 of	2	G6D6V5_DANPL	Protease m1 zinc metalloprotease Danaus plexippus (Monarch	0,829	1,222	0,954	28,0922
								Agrotis_qualite_rep_c1757|ORF25 length 189 aa, 570 bp, from 1081..1650 of	1			0,829	1,222	0,954	28,0922
								Agrotis_qualite_rep_c659|ORF23 length 505 aa, 1518 bp, from 992..2509 of	3			0,829	1,222	0,954	28,0922
								Agrotis_qualite_c19428|ORF1 length 480 aa, 1440 bp, from 1..1440 of	2			0,829	1,222	0,954	28,0922
ID: 0190	**128**	0,07691473	1,406	0,01126	1,879	0,27479588	0,748					1,285	0,684	0,914	-25,1641
ID: 0191	**129**	0,76237406	0,982	0,83328	0,987	0,92159309	0,995					1,055	1,069	1,074	-0,4655
ID: 0192	**130**	0,41527021	0,883	0,63037	1,128	0,08559066	0,783					0,839	0,744	0,95	-21,6842
ID: 0196	**131**	0,47282263	0,941	0,42052	0,849	0,59005122	1,108					0,969	1,141	1,030	10,7767
ID: 0198	**132**	0,15456727	1,308	0,03589	1,767	0,28007981	0,740					1,325	0,75	1,013	-25,9625
ID: 0199	**133**	0,09784995	1,325	0,01558	1,794	0,2210639	0,739					1,166	0,65	0,88	-26,1364
ID: 0200	**134**	0,09121386	1,336	0,02094	1,607	0,3819157	0,831					1,300	0,809	0,973	-16,8551
ID: 0201	**135**	0,18531292	0,899	0,03072	0,852	0,39590031	1,055					0,97	1,138	1,079	5,4680
ID: 0202	**136**	0,3856926	1,151	0,15678	1,341	0,71820496	0,858					1,187	0,885	1,031	-14,1610
ID: 0204	**137**	0,46357303	0,957	0,15488	0,882	0,28871032	1,085					0,933	1,058	0,975	8,5128
ID: 0205	**138**	0,20545625	1,298	0,06165	1,640	0,46946302	0,791					1,332	0,812	1,026	-20,8577
ID: 0207	**139**	0,03593896	0,751	0,01636	0,691	0,43489092	1,086					0,771	1,115	1,027	8,5686
ID: 0208	**140**	0,10454552	0,790	0,04449	0,818	0,85997969	0,966					0,89	1,088	1,126	-3,3748
ID: 0209	**141**	0,04124797	1,934	0,20192	1,551	0,69087219	1,247					1,607	1,036	0,831	24,6691
ID: 0210	**142**	0,82935448	1,042	0,97286	0,989	0,80300726	1,054					1,006	1,017	0,965	5,3886
ID: 0211	**143**	0,21942409	1,157	0,37137	0,905	0,09927918	1,278					1,071	1,183	0,926	27,7538
ID: 0212	**144**	0,96542893	1,003	0,01837	1,202	0,01303085	0,835	Agrotis_qualite_rep_c1513|ORF1 length 495 aa, 1488 bp, from 1..1488 of Agrotis_qualite_rep_c1513	2	OGT1_HUMAN	UDP-N-acetylglucosamine--peptide N-acetylglucosaminyltransferase 110 kDa subunit	0,971	0,808	0,968	-16,5289
ID: 0213	**145**	0,07500648	0,810	0,68895	0,967	0,01603959	0,838					0,933	0,965	1,152	-16,2326
ID: 0214	**146**	0,26870682	0,866	0,01201	0,780	0,29134278	1,111					0,895	1,148	1,033	11,1326
ID: 0215	**147**	0,85231754	1,012	0,64076	0,958	0,51510529	1,056					0,845	0,882	0,835	5,6287
ID: 0216	**148**	0,02506954	0,359	0,00075	0,315	0,4120063	1,141					0,406	1,289	1,130	14,0708

ID: 0217	**149**	0,11100978	0,761	0,00991	0,708	0,54041645	1,075					0,845	1,194	1,111	7,4707
ID: 0218	**150**	0,51148639	0,895	0,30016	0,775	0,48158355	1,154					0,877	1,131	0,98	15,4082
ID: 0219	**151**	0,08956243	1,208	0,1517	0,818	0,01831897	1,477					1,051	1,285	0,87	47,7011
ID: 0220	**152**	0,56123429	0,922	0,31834	0,790	0,42600945	1,167					0,877	1,110	0,951	16,7192
ID: 0221	**153**	0,44938663	0,896	0,32325	0,823	0,57172883	1,089					0,91	1,106	1,016	8,8583
ID: 0222	**154**	0,04523698	0,825	0,16045	0,792	0,82741307	1,041					0,78	0,985	0,946	4,1226
ID: 0223	**155**	0,1804382	0,882	0,03314	0,815	0,27362356	1,083					0,915	1,123	1,037	8,2932
ID: 0224	**156**	0,78605212	1,049	0,48763	0,934	0,09555689	1,123					0,88	0,942	0,839	12,2765
ID: 0226	**157**	0,02331796	1,272	0,31706	0,892	0,01503055	1,426					1,207	1,353	0,949	42,5711
ID: 0227	**158**	0,12311334	1,126	0,28002	1,128	0,90806987	0,998					1,135	1,006	1,008	-0,1984
ID: 0228	**159**	0,46538235	0,960	0,05392	0,863	0,02806849	1,112					0,933	1,081	0,972	11,2140
ID: 0229	**160**	0,19385296	0,855	0,33874	0,843	0,99018403	1,014					0,826	0,98	0,966	1,4493
ID: 0230	**161**	0,64239912	0,974	0,22875	0,891	0,22060998	1,093					0,938	1,053	0,963	9,3458
ID: 0231	**162**	0,48839652	0,976	0,01674	0,880	0,01554553	1,108					0,921	1,046	0,944	10,8051
ID: 0232	**163**	0,42302863	0,886	0,17815	0,782	0,33087961	1,133					0,932	1,192	1,052	13,3080
ID: 0233	**164**	0,16295663	0,920	0,06638	0,877	0,14167693	1,050					0,926	1,056	1,006	4,9702
ID: 0234	**165**	0,76329521	1,027	0,47515	0,859	0,36143362	1,195					1,099	1,279	1,070	19,5327
ID: 0235	**166**	0,8787491	1,029	0,11119	0,759	0,04382787	1,356					1,053	1,387	1,023	35,5816
ID: 0236	**167**	0,3656432	1,112	0,69279	0,946	0,25284378	1,175					1,216	1,285	1,094	17,4589
ID: 0237	**168**	0,82463741	1,026	0,19405	0,851	0,18552745	1,204					0,883	1,037	0,861	20,4413
ID: 0238	**169**	0,12964706	0,833	0,06212	0,798	0,5921688	1,044					0,967	1,212	1,161	4,3928
ID: 0239	**170**	0,46176364	0,901	0,00105	0,844	0,47176993	1,068					0,955	1,132	1,060	6,7925
ID: 0240	**171**	0,76299956	0,954	0,04597	0,849	0,23197732	1,124					0,975	1,149	1,022	12,4266
ID: 0241	**172**	0,09872869	1,227	0,51971	1,089	0,09559369	1,127					1,150	1,056	0,937	12,7001
ID: 0242	**173**	0,1831594	0,914	0,02196	0,834	0,05657441	1,096					0,916	1,098	1,002	9,5808
ID: 0243	**174**	0,03530578	1,262	0,16991	1,224	0,89757759	1,032					1,193	0,975	0,945	3,1746
ID: 0244	**175**	0,03754053	1,204	0,05148	1,382	0,22925524	0,871					1,204	0,871	1,000	-12,9000
ID: 0245	**176**	0,66618842	0,868	0,6944	0,948	0,83575076	0,916					0,94	0,992	1,083	-8,4026
ID: 0246	**177**	0,42389945	0,940	0,56824	0,961	0,62125864	0,979					0,975	1,015	1,037	-2,1215
ID: 0247	**178**	0,67151459	0,985	0,9819	0,992	0,84013213	0,994					1,062	1,071	1,078	-0,6494
ID: 0248	**179**	0,56665246	0,961	0,06778	0,899	0,25037072	1,069					0,873	0,971	0,908	6,9383
ID: 0249	**180**	0,44278987	0,936	0,63989	0,965	0,61229031	0,969					0,92	0,953	0,983	-3,0519
ID: 0250	**181**	0,73078265	0,978	0,44115	0,902	0,39370591	1,084					0,896	0,993	0,916	8,4061
ID: 0251	**182**	0,07421322	0,812	0,79537	1,036	0,04153805	0,784					0,875	0,845	1,078	-21,6141
ID: 0252	**183**	0,73168973	0,984	0,81663	0,987	0,92865057	0,997					1,077	1,091	1,094	-0,2742
ID: 0253	**184**	0,62375072	1,071	0,86248	0,955	0,55247158	1,122					1,134	1,188	1,059	12,1813
ID: 0254	**185**	0,51266775	1,046	0,26804	1,072	0,66512981	0,976					0,908	0,847	0,868	-2,4194
ID: 0255	**186**	0,15160775	1,145	0,26147	1,168	0,80643326	0,981					1,323	1,133	1,155	-1,9048
ID: 0256	**187**	0,6691635	1,022	0,09466	1,137	0,06280029	0,898					0,993	0,873	0,972	-10,1852
ID: 0257	**188**	0,34057613	0,914	0,59293	0,909	0,93495012	1,005					0,9	0,99	0,985	0,5076

ID: 0258	**189**	0,09414214	0,805	0,93517	0,996	0,05696796	0,808					0,817	0,82	1,015	-19,2118
ID: 0259	**190**	0,45737877	0,940	0,74459	0,955	0,79352707	0,984					0,871	0,912	0,927	-1,6181
ID: 0260	**191**	0,63959022	1,085	0,13	1,308	0,07817592	0,829					0,883	0,675	0,814	-17,0762
ID: 0261	**192**	0,16171569	0,901	0,11535	0,869	0,55596401	1,037					0,971	1,118	1,078	3,7106
ID: 0262	**193**	0,60173026	0,943	0,93793	1,033	0,42001819	0,913					0,882	0,854	0,935	-8,6631
ID: 0263	**194**	0,90001943	0,987	0,87668	1,025	0,74162646	0,962					0,805	0,785	0,816	-3,7990
ID: 0264	**195**	0,5401697	0,960	0,44979	1,074	0,04137774	0,894					0,897	0,835	0,934	-10,5996
ID: 0265	**196**	0,42481382	1,086	0,37393	0,816	0,14916663	1,330					0,881	1,079	0,811	33,0456
ID: 0266	**197**	0,88563415	1,014	0,03861	1,248	0,03291171	0,812					0,8	0,641	0,789	-18,7579
ID: 0267	**198**	0,65476876	0,969	0,08059	0,849	0,08111351	1,141					0,998	1,175	1,030	14,0777
ID: 0268	**199**	0,49567266	0,938	0,87296	0,987	0,57559093	0,951					0,808	0,819	0,861	-4,8780
ID: 0269	**200**	0,19315258	0,839	0,04046	0,668	0,17298043	1,257					0,752	1,126	0,896	25,6696
ID: 0270	**201**	0,71724968	0,976	0,73867	0,959	0,91312398	1,017					0,84	0,876	0,861	1,7422
ID: 0271	**202**	0,2548602	1,175	0,36699	1,183	0,84067275	0,994					1,092	0,923	0,929	-0,6459
ID: 0272	**203**	0,25735749	0,883	0,08566	0,803	0,28285384	1,101					0,894	1,114	1,012	10,0791
ID: 0273	**204**	0,95357282	1,000	0,48845	1,072	0,57299771	0,933					0,939	0,876	0,939	-6,7093
ID: 0274	**205**	0,8080515	1,017	0,1133	1,115	0,36048363	0,912					0,846	0,759	0,832	-8,7740
ID: 0275	**206**	0,75763646	0,982	0,56205	1,120	0,13703341	0,877					0,729	0,651	0,742	-12,2642
ID: 0276	**207**	0,26586855	1,211	0,96721	1,005	0,28844562	1,205					0,951	0,946	0,785	20,5096
ID: 0277	**208**	0,68855826	1,052	0,20972	0,870	0,06678811	1,209					1,012	1,163	0,962	20,8940
ID: 0279	**209**	0,19783634	0,875	0,81384	0,973	0,33947483	0,899					0,801	0,823	0,915	-10,0546
ID: 0280	**210**	0,73596442	1,032	0,78954	1,025	0,96206009	1,007					0,858	0,837	0,831	0,7220
ID: 0282	**211**	0,50595251	0,911	0,36685	0,876	0,7381371	1,040					0,816	0,932	0,896	4,0179
ID: 0283	**212**	0,14201808	0,892	0,05347	0,830	0,13573498	1,074					1,006	1,212	1,128	7,4468
ID: 0284	**213**	0,15371635	0,894	0,02438	0,798	0,08953097	1,120					1,005	1,259	1,124	12,0107
ID: 0285	**214**	0,22573077	1,269	0,12749	1,532	0,49201839	0,828					1,339	0,874	1,055	-17,1564
ID: 0286	**215**	0,3761236	0,960	0,02858	0,889	0,03856304	1,080					1,109	1,247	1,155	7,9654
ID: 0290	**216**	0,76913042	1,014	0,82301	0,974	0,66971205	1,041					1,085	1,114	1,070	4,1121
ID: 0291	**217**	0,27787252	1,198	0,69994	1,080	0,25941713	1,109					1,240	1,148	1,035	10,9179
ID: 0292	**218**	0,2809748	0,933	0,0575	0,799	0,05376141	1,168					1,003	1,256	1,075	16,8372
ID: 0293	**219**	0,92184379	1,005	0,4278	0,939	0,40111961	1,070					1,082	1,152	1,077	6,9638
ID: 0294	**220**	0,41185179	0,959	0,68121	0,973	0,69409417	0,986					1,039	1,068	1,083	-1,3850
ID: 0295	**221**	0,23937938	0,886	0,02832	0,779	0,19872264	1,138					0,872	1,120	0,984	13,8211
ID: 0297	**222**	0,02878239	0,797	0,0066	0,740	0,2364046	1,077					0,948	1,281	1,189	7,7376
ID: 0298	**223**	0,16147573	0,876	0,03366	0,791	0,13605975	1,106					0,987	1,247	1,127	10,6477
ID: 0299	**224**	0,26320505	1,083	0,3435	1,073	0,8617962	1,009					1,046	0,975	0,966	0,9317
ID: 0300	**225**	0,14774608	1,288	0,03147	1,439	0,59768721	0,895					1,396	0,97	1,084	-10,5166
ID: 0301	**226**	0,08906691	0,916	0,07978	0,867	0,48207958	1,056					0,896	1,033	0,978	5,6237
ID: 0302	**227**	0,12678073	0,894	0,03028	0,842	0,32515594	1,062					0,985	1,170	1,102	6,1706
ID: 0303	**228**	0,0004124	0,614	0,0032	0,626	0,79338947	0,980					0,785	1,253	1,278	-1,9562

ID: 0304	**229**	0,31611485	1,085	0,27907	1,106	0,79000153	0,981					1,157	1,046	1,066	-1,8762
ID: 0305	**230**	0,74990389	1,055	0,48498	0,927	0,13086334	1,139					1,110	1,198	1,052	13,8783
ID: 0306	**231**	0,53319256	0,917	0,09335	0,808	0,18919015	1,136					0,999	1,237	1,089	13,5904
ID: 0307	**232**	0,37503141	0,946	0,12469	0,904	0,32166442	1,047					1,068	1,182	1,129	4,6944
ID: 0308	**233**	0,06247031	0,879	0,05521	0,852	0,55388596	1,033					0,889	1,044	1,011	3,2641
ID: 0309	**234**	0,40897253	0,948	0,09784	0,870	0,06100606	1,090					0,972	1,117	1,025	8,9756
ID: 0310	**235**	0,54717449	0,855	0,26431	1,153	0,21765874	0,741					0,74	0,642	0,866	-25,8661
ID: 0311	**236**	0,28042025	0,899	0,04206	0,802	0,08374443	1,121					1,027	1,280	1,142	12,0841
ID: 0312	**237**	0,61941328	0,938	0,19825	0,799	0,20745468	1,173					1,152	1,441	1,228	17,3453
ID: 0313	**238**	0,06614716	0,738	0,08205	0,739	0,99905971	0,998					0,936	1,266	1,268	-0,1577
ID: 0314	**239**	0,06946691	0,877	0,05805	0,860	0,5498977	1,020					0,895	1,041	1,021	1,9589
ID: 0315	**240**	0,48241168	0,920	0,23461	0,871	0,62133277	1,056					1,017	1,168	1,106	5,6058
ID: 0316	**241**	0,01348442	0,716	0,04241	0,816	0,27871338	0,876					0,903	1,106	1,262	-12,3613
ID: 0317	**242**	0,6063169	0,958	0,0745	0,795	0,07281844	1,205					0,945	1,188	0,986	20,4868
ID: 0320	**243**	0,13843582	1,119	0,58787	1,061	0,84417777	1,054					1,074	1,012	0,96	5,4167
ID: 0321	**244**	0,59803334	1,145	0,81328	1,095	0,51825812	1,045					1,140	1,041	0,996	4,5181
ID: 0323	**245**	0,8143513	0,966	0,04802	0,773	0,08249581	1,249					0,907	1,173	0,939	24,9201
ID: 0325	**246**	0,90379425	0,974	0,16705	1,252	0,15362641	0,778					1,038	0,829	1,066	-22,2326
ID: 0326	**247**	0,5543617	0,941	0,57434	0,950	0,94506917	0,990					0,983	1,035	1,045	-0,9569
ID: 0328	**248**	0,35503521	1,031	0,6663	1,020	0,86251607	1,011					1,087	1,066	1,054	1,1385
ID: 0330	**249**	0,21167268	0,896	0,00821	0,815	0,23226126	1,100					0,96	1,178	1,071	9,9907
ID: 0331	**250**	0,65186973	1,045	0,80013	0,935	0,55640995	1,118					1,088	1,164	1,041	11,8156
ID: 0332	**251**	0,11550911	0,855	0,03156	0,825	0,63853514	1,037					0,89	1,079	1,041	3,6503
ID: 0333	**252**	0,4779112	0,958	0,42122	0,910	0,59285429	1,053					0,976	1,073	1,019	5,2993
ID: 0334	**253**	0,01696596	1,173	0,17372	1,090	0,13438239	1,077					1,286	1,180	1,096	7,6642
ID: 0335	**254**	0,74472069	0,974	0,08519	0,835	0,07244406	1,166					0,978	1,171	1,004	16,6335
ID: 0336	**255**	0,58676406	1,050	0,70757	1,031	0,76602825	1,019					1,070	1,038	1,019	1,8646
ID: 0337	**256**	0,13887195	1,100	0,09758	1,108	0,93168611	0,993					1,153	1,041	1,048	-0,6679
ID: 0338	**257**	0,12222162	1,096	0,54427	1,042	0,40745978	1,051					1,193	1,145	1,089	5,1423
ID: 0339	**258**	0,58098255	0,944	0,10499	0,822	0,06666612	1,148					1,101	1,339	1,166	14,8370
ID: 0340	**259**	0,7947791	0,986	0,13202	0,932	0,25202936	1,057					1,091	1,170	1,107	5,6911
ID: 0341	**260**	0,33903413	0,857	0,99208	1,008	0,29280212	0,851					1,052	1,044	1,227	-14,9144
ID: 0342	**261**	0,48338014	0,964	0,15502	0,904	0,38270498	1,067					0,985	1,090	1,022	6,6536
ID: 0343	**262**	0,60942858	0,951	0,92013	1,011	0,52000784	0,941					1,012	1,001	1,064	-5,9211
ID: 0345	**263**	0,26793043	0,933	0,06733	0,853	0,1862387	1,094					0,911	1,068	0,976	9,4262
ID: 0346	**264**	0,53942871	0,967	0,19126	0,850	0,26082212	1,137					0,874	1,028	0,904	13,7168
ID: 0347	**265**	0,69719381	1,112	0,04704	1,760	0,04889024	0,632					0,535	0,304	0,481	-36,7983
ID: 0349	**266**	0,10114734	0,865	0,01222	0,818	0,49284584	1,058					0,926	1,132	1,070	5,7944
ID: 0353	**267**	0,19426604	0,588	0,26246	0,734	0,65870235	0,801					0,71	0,967	1,207	-19,8840
ID: 0354	**268**	0,1243044	0,927	0,25465	0,907	0,79658266	1,022					0,974	1,074	1,051	2,1884

ID: 0355	**269**	0,79834828	1,012	0,86075	1,009	0,95826294	1,003					0,981	0,972	0,969	0,3096
ID: 0356	**270**	0,53991465	1,074	0,75286	1,049	0,54688275	1,024					1,050	1,001	0,978	2,3517
ID: 0357	**271**	0,42030223	1,071	0,01322	1,205	0,30645772	0,889					1,115	0,925	1,041	-11,1431
ID: 0358	**272**	0,93192141	1,005	0,7686	0,958	0,71321291	1,049					1,070	1,117	1,065	4,8826
ID: 0359	**273**	0,26446902	1,084	0,25301	1,116	0,75949853	0,971					1,249	1,119	1,152	-2,8646
ID: 0360	**274**	0,62980919	1,130	0,25253	1,324	0,31372957	0,853					1,041	0,786	0,921	-14,6580
ID: 0361	**275**	0,45202236	1,054	0,01575	1,273	0,09959045	0,828					1,116	0,877	1,059	-17,1860
ID: 0362	**276**	0,93417193	0,983	0,32098	1,139	0,30901141	0,863					0,997	0,875	1,014	-13,7081
ID: 0363	**277**	0,25548837	1,134	0,06342	1,256	0,33437766	0,903					1,248	0,994	1,101	-9,7184
ID: 0364	**278**	0,41331052	1,058	0,2213	0,910	0,03502336	1,163					1,143	1,256	1,080	16,2963
ID: 0365	**279**	0,80046467	0,992	0,45768	0,951	0,40194281	1,043					0,972	1,022	0,98	4,2857
ID: 0366	**280**	0,98200473	0,998	0,52522	1,051	0,58836259	0,950					0,971	0,924	0,973	-5,0360
ID: 0367	**281**	0,4039208	0,932	0,20678	1,144	0,02991613	0,815					1,032	0,902	1,107	-18,5185
ID: 0368	**282**	0,0624504	0,890	0,01333	0,856	0,43920066	1,040					1,009	1,179	1,134	3,9683
ID: 0369	**283**	0,01059838	0,908	0,03397	0,909	0,94100421	0,998					1,034	1,137	1,139	-0,1756
ID: 0371	**284**	0,10850193	1,561	0,12851	1,603	0,84972678	0,974					1,391	0,868	0,891	-2,5814
ID: 0372	**285**	0,52161774	0,951	0,82726	1,035	0,21779223	0,919					0,935	0,903	0,983	-8,1384
ID: 0374	**286**	0,55573127	1,034	0,21089	1,083	0,57968468	0,955					1,004	0,927	0,971	-4,5314
ID: 0375	**287**	0,96747309	1,006	0,68363	0,967	0,60932323	1,040					0,945	0,977	0,939	4,0469
ID: 0376	**288**	0,0293291	0,901	0,12793	0,908	0,79332729	0,993					0,974	1,073	1,081	-0,7401
ID: 0378	**289**	0,76970923	1,025	0,96965	0,989	0,77185201	1,036					1,127	1,139	1,099	3,6397
ID: 0379	**290**	0,03040604	0,845	0,09202	0,872	0,5748129	0,969					0,977	1,120	1,156	-3,1142
ID: 0380	**291**	0,89322338	1,021	0,61401	1,067	0,6268744	0,957					0,987	0,925	0,967	-4,3433
ID: 0381	**292**	0,82070235	1,012	0,69096	0,980	0,66263782	1,033					1,075	1,097	1,062	3,2957
ID: 0382	**293**	0,01673726	0,874	0,08605	0,900	0,42103635	0,972					0,961	1,068	1,099	-2,8207
ID: 0384	**294**	0,93014902	0,938	0,07509	1,130	0,66421397	0,830					1,139	1,008	1,214	-16,9687
ID: 0385	**295**	0,11946106	1,249	0,00407	1,450	0,42016315	0,861					1,285	0,886	1,029	-13,8970
ID: 0386	**296**	0,9714869	1,001	0,21529	0,906	0,10539083	1,105					1,013	1,118	1,012	10,4743
ID: 0387	**297**	0,04093541	0,894	0,06238	0,864	0,53645805	1,034					0,966	1,118	1,081	3,4228
ID: 0388	**298**	0,02927877	0,877	0,01281	0,833	0,39190512	1,052					0,89	1,068	1,015	5,2217
ID: 0390	**299**	0,70946445	1,024	0,56554	1,063	0,6538099	0,963					0,995	0,936	0,972	-3,7037
ID: 0391	**300**	0,39923842	0,776	0,58322	0,944	0,55234517	0,822					0,912	0,966	1,175	-17,7872
ID: 0392	**301**	0,66492665	0,933	0,57004	1,030	0,50843728	0,906					0,931	0,904	0,998	-9,4188
ID: 0393	**302**	0,31776462	1,096	0,64705	0,972	0,04375719	1,127					1,049	1,079	0,957	12,7482
ID: 0394	**303**	0,03608443	0,914	0,00592	0,825	0,01993828	1,108					0,929	1,126	1,016	10,8268
ID: 0395	**304**	0,33170142	0,847	0,42418	0,854	0,94150586	0,992					0,849	0,994	1,002	-0,7984
ID: 0396	**305**	0,60139279	0,452	0,83458	0,982	0,6280703	0,460					0,902	0,919	1,996	-53,9579
ID: 0397	**306**	0,94470836	0,998	0,43198	0,946	0,34095447	1,055					0,951	1,005	0,953	5,4565
ID: 0399	**307**	0,21624932	0,665	0,25068	0,804	0,63982686	0,827					0,681	0,847	1,024	-17,2852
ID: 0400	**308**	0,39911823	0,948	0,72541	0,907	0,96136763	1,045					0,869	0,958	0,917	4,4711

ID: 0402	**309**	0,72404728	0,975	0,64562	0,941	0,77596574	1,036					0,944	1,003	0,968	3,6157
ID: 0403	**310**	0,0478282	0,866	0,66216	0,962	0,12683723	0,900					0,966	1,004	1,115	-9,9552
ID: 0404	**311**	0,07592792	0,871	0,57874	1,058	0,13855707	0,824					1,023	0,967	1,174	-17,6320
ID: 0408	**312**	0,16020029	0,846	0,26643	0,889	0,66660309	0,952					0,878	0,988	1,038	-4,8170
ID: 0409	**313**	0,10177867	0,860	0,03477	0,839	0,6936277	1,025					0,929	1,107	1,080	2,5000
ID: 0410	**314**	0,13156923	0,879	0,12597	0,856	0,64113363	1,027					0,9	1,052	1,024	2,7344
ID: 0411	**315**	0,09943947	1,177	0,06489	1,237	0,51741207	0,951					0,991	0,801	0,842	-4,8694
ID: 0412	**316**	0,6018775	0,866	0,04195	0,772	0,4675807	1,121					0,856	1,109	0,989	12,1335
ID: 0413	**317**	0,1179491	0,901	0,00272	0,832	0,14226085	1,083					0,919	1,105	1,020	8,3333
ID: 0414	**318**	0,83554876	0,961	0,25489	1,134	0,27850461	0,848					1,047	0,923	1,089	-15,2433
ID: 0415	**319**	0,06414853	0,878	0,16512	0,885	0,87919327	0,992					0,932	1,053	1,061	-0,7540
ID: 0417	**320**	0,37081718	0,919	0,05439	0,821	0,27336162	1,118					1,071	1,304	1,166	11,8353
ID: 0418	**321**	0,19146287	0,959	0,25771	0,956	0,92006916	1,003					0,937	0,98	0,977	0,3071
ID: 0419	**322**	0,71483647	0,947	0,8775	0,995	0,78571907	0,952					1,085	1,091	1,146	-4,7993
ID: 0421	**323**	0,32771635	0,965	0,31574	0,958	0,84358362	1,007					0,943	0,984	0,977	0,7165
ID: 0422	**324**	0,27481737	0,951	0,01693	0,888	0,07452768	1,070					1,082	1,218	1,138	7,0299
ID: 0423	**325**	0,11361347	0,856	0,17572	0,887	0,73068101	0,966					0,877	0,989	1,024	-3,4180
ID: 0424	**326**	0,10914285	1,150	0,33057	1,158	0,7953325	0,993					1,155	0,997	1,004	-0,6972
ID: 0425	**327**	0,63409437	0,974	0,2481	0,954	0,65101068	1,021					1,037	1,087	1,065	2,0657
ID: 0426	**328**	0,31561603	0,952	0,20502	0,913	0,39583198	1,044					1,002	1,098	1,052	4,3726
ID: 0429	**329**	0,2110821	0,925	0,02578	0,863	0,11602862	1,071					0,992	1,149	1,073	7,0829
ID: 0430	**330**	0,3484046	1,058	0,99747	1,000	0,35108869	1,058					1,023	1,023	0,967	5,7911
ID: 0431	**331**	0,3512349	1,048	0,57114	0,981	0,2608812	1,068					0,98	0,999	0,935	6,8449
ID: 0432	**332**	0,73657877	0,990	0,55148	0,971	0,55373267	1,019					0,97	0,999	0,98	1,9388
ID: 0433	**333**	0,53887901	1,047	0,2522	0,899	0,13507338	1,164					1,071	1,191	1,023	16,4223
ID: 0434	**334**	0,38525291	1,053	0,00331	1,079	0,75663368	0,975					0,982	0,91	0,933	-2,4652
ID: 0435	**335**	0,85081814	1,010	0,73151	1,017	0,93129601	0,994					0,982	0,966	0,972	-0,6173
ID: 0437	**336**	0,28928152	0,955	0,36862	0,936	0,73828729	1,020					0,842	0,9	0,882	2,0408
ID: 0438	**337**	0,43988356	1,038	0,88351	0,993	0,43289864	1,045					1,018	1,025	0,981	4,4852
ID: 0439	**338**	0,30568101	0,921	0,4219	0,922	0,95298807	0,999					0,938	1,017	1,018	-0,0982
ID: 0440	**339**	0,26048943	0,923	0,03868	0,856	0,24268116	1,078					1,038	1,212	1,124	7,8292
ID: 0441	**340**	0,41001325	0,930	0,30411	0,962	0,73514814	0,967					1,027	1,068	1,104	-3,2609
ID: 0442	**341**	0,21559654	1,130	0,72503	1,039	0,58184302	1,088					0,876	0,843	0,775	8,7742
ID: 0443	**342**	0,08747671	1,131	0,11258	1,141	0,90485742	0,991					1,030	0,903	0,911	-0,8782
ID: 0444	**343**	0,34544297	0,877	0,80083	0,963	0,54156749	0,911					0,904	0,939	1,031	-8,9234
ID: 0445	**344**	0,56196604	1,041	0,52932	0,948	0,17766949	1,097					0,973	1,026	0,935	9,7326
ID: 0446	**345**	0,28783022	1,078	0,83964	1,018	0,45060521	1,059					1,023	1,005	0,949	5,9009
ID: 0447	**346**	0,04613388	1,339	0,10033	1,355	0,85898574	0,989					1,173	0,866	0,876	-1,1416
ID: 0448	**347**	0,1635168	0,838	0,0303	0,889	0,69363003	0,943					0,88	0,99	1,050	-5,7143
ID: 0449	**348**	0,90430897	1,008	0,85035	0,992	0,75410164	1,017					1,069	1,078	1,060	1,6981

ID: 0450	**349**	0,95495356	0,997	0,14706	0,849	0,10724525	1,174					0,961	1,132	0,964	17,4274
ID: 0451	**350**	0,75794726	0,983	0,72447	0,950	0,81853617	1,034					0,8	0,842	0,814	3,4398
ID: 0452	**351**	0,84307751	0,964	0,40439	1,111	0,35238566	0,868					0,969	0,872	1,005	-13,2338
ID: 0453	**352**	0,27387178	1,155	0,20254	1,230	0,63982254	0,939					1,113	0,905	0,964	-6,1203
ID: 0454	**353**	0,37556883	0,761	0,79883	0,927	0,5384293	0,822					0,87	0,939	1,143	-17,8478
ID: 0455	**354**	0,38365046	1,040	0,99833	0,995	0,55775175	1,046					1,140	1,146	1,096	4,5620
ID: 0457	**355**	0,42102215	0,885	0,30861	1,132	0,13532361	0,782					1,010	0,892	1,141	-21,8230
ID: 0459	**356**	0,30971714	0,825	0,55168	0,849	0,80295573	0,971					0,824	0,97	0,999	-2,9029
ID: 0460	**357**	0,68819703	0,911	0,85992	0,987	0,75722777	0,923					0,934	0,946	1,025	-7,7073
ID: 0461	**358**	0,87217203	0,979	0,46355	0,850	0,48596015	1,152					0,962	1,132	0,983	15,1577
ID: 0462	**359**	0,61381437	0,956	0,89972	1,023	0,44842147	0,935					0,857	0,838	0,896	-6,4732
ID: 0464	**360**	0,38693491	0,906	0,69451	0,938	0,74513914	0,967					0,93	0,992	1,026	-3,3138
ID: 0465	**361**	0,03754975	0,813	0,00346	0,674	0,02440354	1,207					0,763	1,132	0,938	20,6823
ID: 0468	**362**	0,20320743	1,098	0,48902	1,056	0,71577183	1,040					1,111	1,052	1,012	3,9526
ID: 0470	**363**	0,60873352	1,024	0,26001	1,073	0,40252394	0,954					0,912	0,85	0,891	-4,6016
ID: 0471	**364**	0,89772763	0,988	0,47307	0,938	0,53575074	1,054					0,756	0,806	0,765	5,3595
ID: 0472	**365**	0,71469291	1,017	0,3533	0,955	0,24321603	1,065					1,091	1,143	1,073	6,5238
ID: 0474	**366**	0,84463114	1,010	0,88302	1,008	0,91742411	1,002					1,036	1,028	1,026	0,1949
ID: 0476	**367**	0,87132458	1,010	0,33821	1,067	0,49792538	0,946					0,952	0,892	0,943	-5,4083
ID: 0477	**368**	0,16131951	0,866	0,07802	0,720	0,28762452	1,203					0,809	1,124	0,934	20,3426
ID: 0478	**369**	0,81794874	0,984	0,26556	0,933	0,40618436	1,055					1,094	1,173	1,112	5,4856
ID: 0479	**370**	0,77160285	1,019	0,01812	1,194	0,23719066	0,853					1,069	0,895	1,049	-14,6806
ID: 0480	**371**	0,59997571	0,954	0,11175	1,168	0,0798706	0,817					0,985	0,843	1,032	-18,3140
ID: 0481	**372**	0,05260902	0,750	0,45746	1,081	0,02207219	0,694					0,916	0,847	1,221	-30,6306
ID: 0482	**373**	0,63321346	1,022	0,02034	1,187	0,03167167	0,861					1,173	0,988	1,148	-13,9373
ID: 0483	**374**	0,09369816	0,763	0,65437	0,854	0,45681849	0,893					0,959	1,123	1,257	-10,6603
ID: 0484	**375**	0,75140314	1,020	0,22129	1,086	0,07812104	0,939					1,032	0,95	1,012	-6,1265
ID: 0485	**376**	0,07953757	0,908	0,12829	0,897	0,78109425	1,012					0,96	1,070	1,057	1,2299
ID: 0486	**377**	0,84942304	1,015	0,33865	1,262	0,302897	0,804					1,049	0,831	1,033	-19,5547
ID: 0487	**378**	0,34705793	1,154	0,55826	1,078	0,6119298	1,071					1,234	1,145	1,069	7,1094
ID: 0488	**379**	0,95550934	1,007	0,50926	1,076	0,4839817	0,936					0,862	0,801	0,856	-6,4252
ID: 0489	**380**	0,6253585	0,937	0,74753	1,040	0,43280882	0,901					1,156	1,112	1,234	-9,8865
ID: 0490	**381**	0,30386962	0,912	0,6864	0,967	0,47877649	0,943					0,847	0,876	0,929	-5,7051
ID: 0491	**382**	0,04206997	1,905	0,00625	2,967	0,19331734	0,642					1,617	0,545	0,849	-35,8068
ID: 0492	**383**	0,03162374	0,896	0,07056	0,900	0,88797075	0,995					0,962	1,069	1,074	-0,4655
ID: 0493	**384**	0,37759211	0,955	0,12159	0,912	0,23170318	1,047					1,036	1,136	1,085	4,7005
ID: 0494	**385**	0,45740005	1,032	0,08017	1,111	0,21989232	0,929					1,073	0,966	1,040	-7,1154
ID: 0495	**386**	0,84803464	0,984	0,46126	1,098	0,22817378	0,897					0,799	0,728	0,812	-10,3448
ID: 0496	**387**	0,15334196	1,074	0,11164	1,141	0,41113942	0,942					1,213	1,063	1,129	-5,8459
ID: 0497	**388**	0,1609006	0,958	0,44332	1,027	0,06746236	0,933					1,045	1,018	1,091	-6,6911

ID: 0498	**389**	0,63262399	0,970	0,77211	1,025	0,46774314	0,946					0,903	0,881	0,931	-5,3706
ID: 0499	**390**	0,7431217	0,957	0,17194	1,261	0,06081134	0,759					0,793	0,629	0,829	-24,1255
ID: 0500	**391**	0,73450163	0,980	0,70113	1,038	0,47794793	0,944					1,102	1,062	1,125	-5,6000
ID: 0501	**392**	0,90883844	1,084	0,18525	1,597	0,06507149	0,678					1,075	0,673	0,992	-32,1573
ID: 0503	**393**	0,00046717	0,837	0,05461	0,870	0,47122864	0,961					0,911	1,047	1,089	-3,8567
ID: 0504	**394**	0,70419922	0,975	0,09555	0,875	0,09283788	1,114					1,048	1,198	1,075	11,4419
ID: 0507	**395**	0,44709533	0,918	0,24163	0,867	0,64081936	1,059					0,765	0,882	0,833	5,8824
ID: 0509	**396**	0,04261237	0,862	0,164	0,876	0,703685	0,985					1,085	1,239	1,258	-1,5103
ID: 0510	**397**	0,21953374	0,926	0,01453	0,815	0,1262192	1,136					0,823	1,010	0,889	13,6108
ID: 0511	**398**	0,57211341	0,953	0,60215	0,955	0,98338074	0,998					0,918	0,961	0,963	-0,2077
ID: 0512	**399**	0,2161033	0,914	0,03416	0,827	0,05993614	1,105					1,007	1,218	1,102	10,5263
ID: 0515	**400**	0,05835929	0,859	0,12209	0,879	0,67965927	0,978					1,001	1,139	1,165	-2,2318
ID: 0516	**401**	0,59153772	0,938	0,01713	0,791	0,11610798	1,186					0,821	1,038	0,875	18,6286
ID: 0517	**402**	0,10075684	1,224	0,85141	1,019	0,12213607	1,201					1,061	1,041	0,867	20,0692
ID: 0518	**403**	0,12386225	1,227	0,67113	1,050	0,32567903	1,168					1,007	0,959	0,821	16,8088
ID: 0519	**404**	0,03498446	0,849	0,19276	0,909	0,36054944	0,934					0,966	1,063	1,138	-6,5905
ID: 0520	**405**	0,3500703	0,857	0,41002	0,918	0,71291425	0,934					0,83	0,904	0,968	-6,6116
ID: 0521	**406**	0,52602899	0,882	0,42593	1,201	0,28226301	0,735					0,682	0,568	0,773	-26,5201
ID: 0522	**407**	0,06571492	1,069	0,36217	1,039	0,50885205	1,029					1,065	1,025	0,996	2,9116
ID: 0523	**408**	0,29922569	0,881	0,43026	1,118	0,04483967	0,788					1,048	0,937	1,189	-21,1943
ID: 0524	**409**	0,47319847	1,085	0,65055	1,051	0,88590142	1,033					1,101	1,048	1,015	3,2512
ID: 0525	**410**	0,73842079	0,989	0,92433	0,986	0,9481353	1,003					0,899	0,912	0,909	0,3300
ID: 0526	**411**	0,83687698	1,004	0,66921	0,973	0,70350738	1,032					1,122	1,153	1,117	3,2229
ID: 0528	**412**	0,43689176	0,970	0,14267	0,929	0,18360192	1,043					1,026	1,104	1,058	4,3478
ID: 0529	**413**	0,1983601	0,763	0,49126	0,824	0,70006229	0,926					0,769	0,933	1,008	-7,4405
ID: 0530	**414**	0,3574597	0,772	0,17053	1,058	0,24533232	0,730					0,909	0,859	1,177	-27,0178
ID: 0531	**415**	0,13649193	1,085	0,16416	1,082	0,95308079	1,003					1,008	0,932	0,929	0,3229
ID: 0532	**416**	0,43785702	0,947	0,07988	0,937	0,82025188	1,010					0,922	0,984	0,974	1,0267
ID: 0533	**417**	0,00052687	0,602	0,01338	0,611	0,78106045	0,985					0,687	1,125	1,142	-1,4886
ID: 0534	**418**	0,22348104	1,040	0,62506	0,985	0,1314955	1,056					0,979	0,994	0,941	5,6323
ID: 0535	**419**	0,36498218	1,107	0,47842	1,051	0,63703874	1,053					1,076	1,024	0,972	5,3498
ID: 0536	**420**	0,43035447	1,055	0,19493	1,111	0,48823908	0,949					1,001	0,901	0,949	-5,0580
ID: 0537	**421**	0,83259799	0,946	0,19123	1,146	0,28439885	0,825					1,077	0,94	1,139	-17,4715
ID: 0538	**422**	0,13590265	0,856	0,32601	0,867	0,84838308	0,987					0,782	0,902	0,914	-1,3129
ID: 0540	**423**	0,18125651	1,368	0,55742	1,123	0,61828686	1,218					1,222	1,088	0,893	21,8365
ID: 0541	**424**	0,70479767	0,976	0,04182	0,915	0,28941515	1,067					1,008	1,102	1,033	6,6796
ID: 0542	**425**	0,29012162	1,154	0,04732	1,305	0,45599631	0,884					0,855	0,655	0,741	-11,6059
ID: 0543	**426**	0,40697114	0,895	0,6292	1,043	0,30452717	0,858					0,821	0,787	0,917	-14,1767
ID: 0544	**427**	0,0898203	0,668	0,68869	0,950	0,13395631	0,703					0,781	0,822	1,170	-29,7436
ID: 0545	**428**	0,73449188	1,029	0,47077	1,068	0,57355441	0,963					1,065	0,997	1,035	-3,6715

ID: 0546	**429**	0,02309073	0,794	0,27398	0,876	0,32222287	0,906					0,827	0,944	1,042	-9,4050
ID: 0547	**430**	0,77240104	0,929	0,10297	0,838	0,40340113	1,109					0,94	1,122	1,012	10,8696
ID: 0548	**431**	0,5081022	0,929	0,72558	1,037	0,26913187	0,896					0,867	0,836	0,933	-10,3966
ID: 0549	**432**	0,03476353	0,930	0,10284	0,906	0,6072613	1,026					1,042	1,150	1,121	2,5870
ID: 0550	**433**	0,87479925	1,001	0,17858	0,902	0,39157008	1,110					0,898	0,996	0,897	11,0368
ID: 0551	**434**	0,00573868	0,616	0,02696	0,645	0,64939985	0,955					0,667	1,034	1,083	-4,5245
ID: 0555	**435**	0,31535858	1,151	0,20993	1,156	0,91583834	0,996					0,861	0,745	0,748	-0,4011
ID: 0556	**436**	0,25674854	0,964	0,26865	0,947	0,62626943	1,019					1,081	1,142	1,121	1,8733
ID: 0557	**437**	0,22018824	1,148	0,08208	1,342	0,3155149	0,856					1,153	0,859	1,004	-14,4422
ID: 0558	**438**	0,76970614	1,014	0,22457	1,155	0,53764418	0,878					0,925	0,801	0,912	-12,1711
ID: 0559	**439**	0,20338415	1,074	0,37821	1,061	0,83936923	1,013					1,069	1,008	0,995	1,3065
ID: 0560	**440**	0,10127869	0,945	0,00025	0,855	0,01329671	1,106					0,965	1,129	1,021	10,5779
ID: 0561	**441**	0,92398279	0,987	0,58631	1,051	0,55463519	0,939					0,89	0,847	0,902	-6,0976
ID: 0562	**442**	0,95789694	0,999	0,46178	1,054	0,28104976	0,948					1,012	0,96	1,013	-5,2320
ID: 0563	**443**	0,51441585	1,030	0,56025	1,025	0,91289133	1,004					0,966	0,942	0,938	0,4264
ID: 0564	**444**	0,25568755	0,793	0,09281	1,408	0,00735117	0,563					0,963	0,684	1,214	-43,6573
ID: 0565	**445**	0,43705991	0,966	0,93297	0,991	0,62226011	0,975					1,056	1,066	1,093	-2,4703
ID: 0566	**446**	0,11835214	1,291	0,08442	1,262	0,79397803	1,024					1,090	0,864	0,844	2,3697
ID: 0567	**447**	0,50474962	1,072	0,35447	0,932	0,2863426	1,151					0,954	1,024	0,89	15,0562
ID: 0568	**448**	0,14243615	1,283	0,01127	1,391	0,78632884	0,923					1,132	0,814	0,882	-7,7098
ID: 0569	**449**	0,08449895	1,165	0,01856	1,195	0,78552357	0,975					1,017	0,851	0,873	-2,5200
ID: 0570	**450**	0,50095709	1,016	0,93903	1,001	0,74534372	1,015					1,128	1,127	1,110	1,5315
ID: 0571	**451**	0,93241895	0,994	0,70722	0,979	0,78366481	1,016					1,143	1,168	1,150	1,5652
ID: 0572	**452**	0,74197225	0,982	0,88988	1,005	0,71665196	0,978					0,891	0,887	0,907	-2,2051
ID: 0573	**453**	0,52918325	0,894	0,72386	0,961	0,71410674	0,930					0,921	0,958	1,030	-6,9903
ID: 0574	**454**	0,52409744	0,799	0,57578	0,924	0,75229559	0,865					0,886	0,959	1,109	-13,5257
ID: 0575	**455**	0,6948532	1,036	0,70512	1,038	0,8649323	0,998					1,003	0,966	0,968	-0,2066
ID: 0576	**456**	0,71998498	0,990	0,04328	0,899	0,02388196	1,102					1,022	1,137	1,032	10,1744
ID: 0577	**457**	0,01873459	1,139	0,13687	1,133	0,99439276	1,005					1,318	1,163	1,157	0,5186
ID: 0578	**458**	0,08095735	1,374	0,29726	1,204	0,6800559	1,142					1,076	0,894	0,783	14,1762
ID: 0579	**459**	0,17353669	1,106	0,04591	1,161	0,52694996	0,952					1,043	0,898	0,943	-4,7720
ID: 0580	**460**	0,10095296	1,064	0,99995	1,001	0,03835004	1,063					1,090	1,089	1,024	6,3477
ID: 0581	**461**	0,40119942	1,048	0,25864	1,083	0,63660875	0,967					1,054	0,973	1,006	-3,2803
ID: 0582	**462**	0,02579383	1,097	0,05159	1,084	0,66630106	1,012					1,034	0,954	0,943	1,1665
ID: 0584	**463**	0,68974013	1,035	0,85335	0,985	0,56560006	1,050					1,285	1,304	1,242	4,9919
ID: 0585	**464**	0,09276807	1,538	0,02075	2,184	0,16525912	0,704					1,492	0,683	0,97	-29,5876
ID: 0587	**465**	0,86437811	1,016	0,65055	1,045	0,72826736	0,973					0,926	0,886	0,911	-2,7442
ID: 0588	**466**	0,50807369	1,016	0,95604	0,998	0,43808643	1,018					1,098	1,100	1,081	1,7576
ID: 0589	**467**	0,8354723	0,978	0,24807	0,925	0,50972086	1,057					1,137	1,229	1,163	5,6750
ID: 0590	**468**	0,38155952	1,071	0,64764	1,037	0,68306788	1,032					1,197	1,154	1,118	3,2200

ID: 0591	**469**	0,91175895	0,998	0,36472	1,084	0,14190759	0,921					1,069	0,986	1,071	-7,9365
ID: 0592	**470**	0,2136547	0,906	0,10101	0,864	0,52511889	1,049					0,93	1,077	1,027	4,8685
ID: 0593	**471**	0,15804754	1,143	0,43871	1,070	0,36762369	1,068					0,993	0,928	0,869	6,7894
ID: 0594	**472**	0,00259265	0,920	0,0002	0,850	0,01335723	1,082					0,892	1,050	0,97	8,2474
ID: 0596	**473**	0,55096498	0,908	0,94102	1,004	0,53555512	0,905					0,971	0,967	1,069	-9,5416
ID: 0597	**474**	0,79398525	0,993	0,11682	0,917	0,12669735	1,083					1,018	1,110	1,025	8,2927
ID: 0598	**475**	0,00029512	2,531	0,01158	2,113	0,45570428	1,198					1,587	0,751	0,627	19,7767
ID: 0599	**476**	0,21774512	1,041	0,09267	1,061	0,52336115	0,981					1,068	1,007	1,026	-1,8519
ID: 0600	**477**	0,61864755	1,105	0,79311	1,072	0,69996885	1,031					0,965	0,9	0,873	3,0928
ID: 0601	**478**	0,19249073	1,619	0,0754	2,227	0,42888481	0,727					1,392	0,625	0,86	-27,3256
ID: 0603	**479**	0,29113302	1,152	0,27273	1,205	0,75816426	0,957					0,877	0,728	0,761	-4,3364
ID: 0604	**480**	0,60200096	1,059	0,19698	0,871	0,04937303	1,217					1,091	1,253	1,030	21,6505
ID: 0606	**481**	0,45975503	0,927	0,10451	0,866	0,45839727	1,071					1,074	1,240	1,158	7,0812
ID: 0608	**482**	0,05451184	0,908	0,09073	0,895	0,71831865	1,014					0,949	1,060	1,045	1,4354
ID: 0609	**483**	0,33793447	0,959	0,3793	0,965	0,90172587	0,994					1,126	1,167	1,174	-0,5963
ID: 0610	**484**	0,29362167	0,960	0,05062	0,939	0,50293488	1,022					1,096	1,167	1,142	2,1891
ID: 0611	**485**	0,21944287	1,422	0,05056	1,806	0,48549226	0,788					1,246	0,69	0,876	-21,2329
ID: 0612	**486**	0,64988058	0,981	0,37511	0,935	0,50834695	1,048					1,159	1,239	1,182	4,8223
ID: 0613	**487**	0,13929111	0,941	0,26751	0,941	0,97899638	1,000					1,045	1,111	1,111	0,0000
ID: 0614	**488**	0,86877764	0,984	0,50742	0,936	0,63501701	1,050					1,074	1,147	1,092	5,0366
ID: 0615	**489**	0,65353488	0,989	0,24036	0,940	0,27451779	1,052					1,082	1,151	1,094	5,2102
ID: 0616	**490**	0,59438411	0,963	0,18483	0,936	0,62624197	1,029					1,012	1,081	1,051	2,8544
ID: 0617	**491**	0,26278536	0,960	0,24692	0,939	0,59784165	1,023					1,100	1,172	1,146	2,2688
ID: 0618	**492**	0,51246096	0,970	0,21476	0,950	0,53131919	1,021					1,074	1,130	1,107	2,0777
ID: 0619	**493**	0,42513214	0,842	0,2812	1,254	0,09708589	0,672					0,904	0,721	1,073	-32,8052
ID: 0620	**494**	0,06376282	0,686	0,38541	0,851	0,23258371	0,806					0,721	0,847	1,051	-19,4101
ID: 0622	**495**	0,85253469	0,988	0,69957	0,965	0,79264709	1,023					1,201	1,244	1,216	2,3026
ID: 0623	**496**	0,62629623	0,960	0,20295	1,197	0,01007541	0,802					0,887	0,741	0,924	-19,8052
ID: 0625	**497**	0,78177051	1,017	0,99104	0,999	0,78338634	1,018					1,102	1,103	1,084	1,7528
ID: 0627	**498**	0,10278132	0,868	0,347	0,881	0,81904409	0,986					0,936	1,063	1,078	-1,3915
ID: 0628	**499**	0,37349343	0,974	0,24989	0,965	0,7717026	1,009					1,035	1,073	1,063	0,9407
ID: 0629	**500**	0,40271497	0,884	0,95584	1,005	0,33751449	0,880					0,742	0,738	0,839	-12,0381
ID: 0630	**501**	0,40837212	0,936	0,65082	0,954	0,70867967	0,981					1,170	1,226	1,250	-1,9200
ID: 0631	**502**	0,31025696	1,206	0,03487	1,474	0,56983557	0,818					0,837	0,568	0,694	-18,1556
ID: 0632	**503**	0,37561782	0,950	0,38749	0,960	0,88222086	0,990					1,114	1,161	1,173	-1,0230
ID: 0633	**504**	0,4581791	1,079	0,5792	1,059	0,81406507	1,019					1,130	1,067	1,047	1,9102
ID: 0635	**505**	0,29647697	1,253	0,10427	1,360	0,99470552	0,922					0,703	0,517	0,561	-7,8431
ID: 0636	**506**	0,13990333	1,254	0,0447	1,378	0,39213038	0,910					0,853	0,619	0,68	-8,9706
ID: 0637	**507**	0,15314664	0,960	0,01735	0,926	0,08785203	1,037					1,076	1,162	1,121	3,6574
ID: 0638	**508**	0,20318587	1,223	0,55128	1,108	0,41501876	1,104					1,235	1,115	1,010	10,3960

ID: 0639	**509**	0,15073651	0,949	0,04693	0,912	0,23304492	1,040					1,020	1,118	1,075	4,0000
ID: 0640	**510**	0,73670507	1,015	0,89192	1,007	0,86060538	1,009					1,074	1,067	1,058	0,8507
ID: 0641	**511**	0,36860579	0,946	0,40364	0,975	0,65844541	0,971					1,059	1,086	1,119	-2,9491
ID: 0642	**512**	0,23015726	1,095	0,13508	1,133	0,7010446	0,966					1,019	0,899	0,931	-3,4372
ID: 0643	**513**	0,80253135	0,983	0,99407	0,997	0,82694172	0,986					1,086	1,089	1,105	-1,4480
ID: 0645	**514**	0,89625736	1,007	0,95445	1,004	0,93075217	1,004					1,091	1,087	1,083	0,3693
ID: 0646	**515**	0,82617162	1,019	0,83402	1,018	0,94778077	1,001					0,742	0,729	0,728	0,1374
ID: 0647	**516**	0,74863026	1,017	0,64003	0,961	0,45941264	1,058					1,048	1,090	1,030	5,8252
ID: 0648	**517**	0,8069907	0,986	0,80947	0,977	0,94225821	1,009					0,867	0,887	0,879	0,9101
ID: 0649	**518**	0,37998924	1,087	0,37703	0,918	0,10468972	1,184					1,214	1,322	1,117	18,3527
ID: 0650	**519**	0,61285262	1,033	0,65816	1,034	0,95002422	0,998					1,140	1,102	1,104	-0,1812
ID: 0651	**520**	0,74454451	1,018	0,7428	1,018	0,97065148	1,000					1,059	1,040	1,040	0,0000
ID: 0652	**521**	0,89599665	1,007	0,75948	1,018	0,8594904	0,989					0,887	0,871	0,881	-1,1351
ID: 0653	**522**	0,4235108	1,037	0,52821	1,032	0,93238743	1,004					0,933	0,904	0,9	0,4444
ID: 0654	**523**	0,82389733	1,015	0,54055	1,038	0,70424094	0,978					1,049	1,011	1,034	-2,2244
ID: 0655	**524**	0,23997497	1,086	0,1299	1,083	0,91164236	1,002					0,951	0,878	0,876	0,2283
ID: 0656	**525**	0,49054749	0,947	0,88818	1,014	0,35457255	0,934					1,007	0,993	1,063	-6,5851
ID: 0657	**526**	0,2663128	0,887	0,3819	0,907	0,8006384	0,977					1,020	1,124	1,150	-2,2609
ID: 0658	**527**	0,47175556	1,064	0,14661	1,141	0,47102892	0,932					0,92	0,806	0,865	-6,8208
ID: 0659	**528**	0,66037339	1,025	0,67575	1,026	0,97933961	0,999					0,872	0,85	0,851	-0,1175
ID: 0660	**529**	0,1712827	0,892	0,1344	0,966	0,34342543	0,924					1,086	1,124	1,217	-7,6417
ID: 0661	**530**	0,84546783	1,071	0,40305	1,238	0,31107191	0,865					0,848	0,685	0,792	-13,5101
ID: 0662	**531**	0,54701178	0,975	0,05575	0,937	0,26431719	1,040					1,132	1,208	1,161	4,0482
ID: 0663	**532**	0,34280536	0,925	0,54149	0,925	0,94901787	1,000					0,994	1,075	1,075	0,0000
ID: 0664	**533**	0,03256689	0,913	0,05594	0,872	0,36243203	1,047					1,093	1,253	1,197	4,6784
ID: 0665	**534**	0,34072599	0,866	0,1998	1,159	0,07690998	0,747					1,012	0,873	1,168	-25,2568
ID: 0666	**535**	0,52067874	1,037	0,64987	1,039	0,89766157	0,998					1,044	1,005	1,007	-0,1986
ID: 0667	**536**	0,78533125	0,987	0,82108	0,979	0,9551227	1,009					0,685	0,7	0,694	0,8646
ID: 0668	**537**	0,41396467	1,111	0,03528	1,295	0,28685183	0,858					1,040	0,803	0,936	-14,2094
ID: 0669	**538**	0,62620785	0,945	0,98545	1,003	0,60131844	0,942					1,012	1,009	1,071	-5,7890
ID: 0670	**539**	0,20096908	0,954	0,03852	0,897	0,15415313	1,063					1,088	1,213	1,141	6,3103
ID: 0671	**540**	0,33395346	0,928	0,22596	0,899	0,6933308	1,032					1,019	1,133	1,098	3,1876
ID: 0672	**541**	0,21042853	0,856	0,25089	0,862	0,96030068	0,992					0,989	1,147	1,156	-0,7785
ID: 0673	**542**	0,83649423	1,009	0,55336	1,041	0,69047889	0,969					1,112	1,068	1,102	-3,0853
ID: 0674	**543**	0,24923086	0,857	0,82454	0,970	0,36155025	0,883					0,947	0,976	1,105	-11,6742
ID: 0675	**544**	0,89896681	0,988	0,28397	0,907	0,29974094	1,090					1,111	1,225	1,124	8,9858
ID: 0676	**545**	0,73334715	1,015	0,04899	1,104	0,16918689	0,919					1,093	0,99	1,077	-8,0780
ID: 0677	**546**	0,35504702	0,805	0,05339	0,615	0,23621064	1,308					0,89	1,447	1,106	30,8318
ID: 0678	**547**	0,81557533	1,009	0,66681	0,982	0,57358178	1,027					1,058	1,077	1,049	2,6692
ID: 0679	**548**	0,29613382	1,180	0,12937	1,307	0,45547968	0,903					1,270	0,972	1,076	-9,6654

ID: 0680	**549**	0,35690797	1,070	0,00669	1,203	0,19580848	0,890					1,330	1,106	1,243	-11,0217
ID: 0681	**550**	0,94755533	0,997	0,34883	0,915	0,29588909	1,090					1,043	1,140	1,046	8,9866
ID: 0682	**551**	0,46082077	1,076	0,8654	0,988	0,3568158	1,089					1,104	1,117	1,026	8,8694
ID: 0683	**552**	0,02015029	1,111	0,73006	1,018	0,08834042	1,091					1,232	1,210	1,109	9,1073
ID: 0685	**553**	0,30042938	1,043	0,92666	0,995	0,23211724	1,048					1,104	1,109	1,058	4,8204
ID: 0687	**554**	0,75133394	0,981	0,66134	0,968	0,83471609	1,013					1,036	1,070	1,056	1,3258
ID: 0688	**555**	0,84600405	1,015	0,42036	1,062	0,30182978	0,956					0,958	0,902	0,944	-4,4492
ID: 0689	**556**	0,94175336	0,995	0,88432	1,010	0,82327969	0,986					1,045	1,035	1,050	-1,4286
ID: 0690	**557**	0,2895187	0,905	0,59878	1,067	0,10914993	0,848					0,997	0,934	1,102	-15,2450
ID: 0692	**558**	0,71044569	1,012	0,29835	0,960	0,10357229	1,053					1,044	1,087	1,032	5,3295
ID: 0693	**559**	0,57920851	1,048	0,20643	1,114	0,52716141	0,941					1,064	0,955	1,015	-5,9113
ID: 0695	**560**	0,87830789	0,995	0,88887	1,014	0,66831348	0,981					1,058	1,043	1,063	-1,8815
ID: 0696	**561**	0,13873608	1,041	0,66227	0,973	0,20196302	1,070					1,131	1,162	1,086	6,9982
ID: 0697	**562**	0,93054758	1,014	0,55631	0,958	0,28320639	1,059					1,067	1,114	1,052	5,8935
ID: 0698	**563**	0,53980395	0,977	0,92006	0,984	0,83954467	0,993					1,015	1,032	1,039	-0,6737
ID: 0699	**564**	0,3601542	1,096	0,30471	1,132	0,49047274	0,968					1,166	1,030	1,064	-3,1955
ID: 0700	**565**	0,23393144	1,152	0,20689	1,102	0,59171346	1,045					1,101	0,999	0,956	4,4979
ID: 0702	**566**	0,95801278	0,983	0,87553	1,007	0,85808263	0,976					0,982	0,975	0,999	-2,4024
ID: 0703	**567**	0,64202028	1,015	0,30019	0,948	0,15934369	1,070					1,067	1,125	1,051	7,0409
ID: 0704	**568**	0,05360709	1,184	0,00795	1,303	0,31891785	0,908					1,134	0,87	0,958	-9,1858
ID: 0706	**569**	0,54890154	0,931	0,29559	1,220	0,05972405	0,763					0,848	0,695	0,911	-23,7102
ID: 0707	**570**	0,52933165	0,979	0,71417	0,970	0,94972131	1,009					0,911	0,939	0,931	0,8593
ID: 0708	**571**	0,5680275	1,070	0,58617	1,076	0,92071516	0,995					1,024	0,952	0,957	-0,5225
ID: 0709	**572**	0,56174268	1,037	0,7692	1,017	0,82989974	1,019					1,048	1,030	1,011	1,8793
ID: 0710	**573**	0,01313826	1,107	0,19506	1,095	0,92195324	1,011					1,087	0,993	0,982	1,1202
ID: 0711	**574**	0,36240589	1,042	0,00596	1,082	0,44816107	0,963					1,106	1,022	1,061	-3,6758
ID: 0712	**575**	0,72380626	1,033	0,9668	1,008	0,65971415	1,024					0,979	0,971	0,948	2,4262
ID: 0713	**576**	0,47112753	1,094	0,17525	1,139	0,86522405	0,960					1,023	0,898	0,935	-3,9572
ID: 0714	**577**	0,13316913	1,116	0,7946	0,979	0,12801747	1,140					0,988	1,009	0,885	14,0113
ID: 0715	**578**	0,72636884	0,993	0,06214	0,936	0,03130855	1,062					1,061	1,134	1,068	6,1798
ID: 0716	**579**	0,51917178	0,750	0,04296	1,194	0,21494682	0,628					0,875	0,733	1,167	-37,1894
ID: 0718	**580**	0,82391449	1,023	0,30531	1,162	0,44253895	0,881					1,012	0,871	0,989	-11,9312
ID: 0719	**581**	0,72093615	1,044	0,50745	1,078	0,87515625	0,968					0,992	0,92	0,95	-3,1579
ID: 0721	**582**	0,14835828	1,102	0,01137	1,243	0,02531813	0,886					1,028	0,827	0,933	-11,3612
ID: 0722	**583**	0,48334752	1,076	0,31318	1,184	0,43636719	0,909					1,029	0,869	0,956	-9,1004
ID: 0723	**584**	0,94993924	0,977	0,27165	1,279	0,23602318	0,763					0,957	0,748	0,98	-23,6735
ID: 0724	**585**	0,66729653	1,058	0,30872	1,148	0,54587675	0,922					1,015	0,884	0,959	-7,8206
ID: 0725	**586**	0,99114148	0,993	0,75496	1,022	0,75756048	0,971					0,932	0,912	0,939	-2,8754
ID: 0726	**587**	0,11992928	1,057	0,08097	1,099	0,29489523	0,962					1,022	0,93	0,967	-3,8263
ID: 0727	**588**	0,28853011	1,078	0,46143	1,086	0,83425673	0,992					0,996	0,917	0,924	-0,7576

ID: 0728	**589**	0,81233804	0,848	0,1006	1,325	0,23505016	0,640					0,942	0,711	1,111	-36,0036
ID: 0729	**590**	0,1321785	0,892	0,15669	0,917	0,66827809	0,973					0,982	1,071	1,101	-2,7248
ID: 0731	**591**	0,69216172	0,985	0,401	0,959	0,62896989	1,026					1,089	1,135	1,106	2,6221
ID: 0733	**592**	0,41381131	1,024	0,18576	1,033	0,83819049	0,992					1,008	0,976	0,984	-0,8130
ID: 0734	**593**	0,1519524	1,206	0,03196	1,377	0,14004075	0,876					1,071	0,778	0,888	-12,3874
ID: 0735	**594**	0,70401835	1,022	0,70914	1,023	0,99428223	0,999					0,898	0,878	0,879	-0,1138
ID: 0736	**595**	0,71941178	1,008	0,43804	0,976	0,31386112	1,034					0,96	0,984	0,952	3,3613
ID: 0737	**596**	0,69468438	1,046	0,08482	1,240	0,14597592	0,844					0,864	0,697	0,826	-15,6174
ID: 0738	**597**	0,34493562	1,121	0,17588	1,221	0,58356002	0,918					1,067	0,874	0,952	-8,1933
ID: 0739	**598**	0,15790612	0,928	0,05004	0,881	0,27502221	1,053					1,152	1,307	1,241	5,3183
ID: 0740	**599**	0,45482401	0,956	0,12096	0,908	0,25197203	1,053					1,150	1,267	1,203	5,3200
ID: 0741	**600**	0,77840242	1,031	0,76393	0,972	0,50648418	1,061					0,957	0,985	0,928	6,1422
ID: 0742	**601**	0,85232912	1,006	0,01249	0,866	0,11590532	1,162					1,040	1,201	1,034	16,1509
ID: 0743	**602**	0,10768036	1,216	0,40028	1,112	0,35000782	1,093					0,872	0,784	0,717	9,3445
ID: 0744	**603**	0,82151986	0,899	0,68315	0,886	0,89301965	1,015					1,017	1,148	1,131	1,5031
ID: 0745	**604**	0,25706918	1,035	0,84893	1,008	0,37289039	1,027					1,091	1,082	1,054	2,6565
ID: 0746	**605**	0,84139931	0,994	0,36235	1,059	0,25106465	0,939					1,133	1,070	1,140	-6,1404
ID: 0747	**606**	0,68088695	1,050	0,83109	1,009	0,93020138	1,040					1,038	1,029	0,989	4,0445
ID: 0748	**607**	0,3044887	1,058	0,02279	1,106	0,43681592	0,957					1,057	0,956	0,999	-4,3043
ID: 0749	**608**	0,80946705	0,989	0,59942	1,026	0,42172996	0,965					1,034	1,008	1,045	-3,5407
ID: 0750	**609**	0,31713865	0,922	0,7839	0,982	0,26615388	0,939					1,159	1,180	1,257	-6,1257
ID: 0752	**610**	0,26595326	0,814	0,20612	0,869	0,78317562	0,937					0,861	0,991	1,058	-6,3327
ID: 0753	**611**	0,99994037	0,992	0,13579	1,140	0,21939393	0,870					0,885	0,776	0,892	-13,0045
ID: 0754	**612**	0,52305043	1,063	0,61298	0,956	0,29961037	1,112					1,033	1,081	0,972	11,2140
ID: 0755	**613**	0,70169469	1,020	0,98559	0,996	0,73165845	1,024					1,047	1,051	1,026	2,4366
ID: 0756	**614**	0,52065093	1,056	0,31035	1,093	0,67327781	0,966					1,081	0,989	1,024	-3,4180
ID: 0757	**615**	0,36605017	1,081	0,09275	1,246	0,30988627	0,868					0,948	0,761	0,877	-13,2269
ID: 0759	**616**	0,83941912	0,979	0,65387	0,951	0,77458585	1,029					1,413	1,486	1,444	2,9086
ID: 0760	**617**	0,76323817	0,990	0,75295	1,013	0,61693632	0,977					0,943	0,931	0,953	-2,3085
ID: 0761	**618**	0,88337481	1,008	0,78992	1,017	0,96542093	0,992					0,975	0,959	0,967	-0,8273
ID: 0762	**619**	0,6370037	0,986	0,09609	0,943	0,11001132	1,045					1,171	1,242	1,188	4,5455
ID: 0763	**620**	0,63343663	0,986	0,08821	0,923	0,04651789	1,068					1,053	1,141	1,068	6,8352
ID: 0764	**621**	0,37309615	1,083	0,83599	0,962	0,37149032	1,125					1,202	1,249	1,110	12,5225
ID: 0765	**622**	0,03546794	1,103	0,23081	0,929	0,00864124	1,188					1,145	1,233	1,038	18,7861
ID: 0766	**623**	0,00230074	0,941	0,00903	0,914	0,19867663	1,030					1,076	1,177	1,143	2,9746
ID: 0768	**624**	0,20930362	0,960	0,11876	0,911	0,33703057	1,054					1,069	1,174	1,114	5,3860
ID: 0769	**625**	0,00643789	0,906	0,11886	0,907	0,94621846	0,999					0,981	1,082	1,083	-0,0923
ID: 0770	**626**	0,92817959	0,995	0,41339	0,965	0,51786674	1,032					1,072	1,111	1,077	3,1569
ID: 0771	**627**	0,30066445	1,099	0,74401	0,964	0,19987527	1,139					1,081	1,121	0,984	13,9228
ID: 0772	**628**	0,95699114	1,001	0,58516	1,046	0,6763895	0,957					0,992	0,948	0,991	-4,3391

ID: 0773	**629**	0,70627673	0,971	0,22377	0,900	0,34679639	1,079					1,105	1,228	1,138	7,9086
ID: 0774	**630**	0,2646729	1,065	0,72522	1,026	0,6897547	1,037					1,204	1,173	1,131	3,7135
ID: 0775	**631**	0,45241173	0,885	0,06842	0,807	0,48855351	1,097					1,167	1,446	1,318	9,7117
ID: 0776	**632**	0,67416855	0,976	0,56264	1,028	0,3432673	0,949					1,016	0,988	1,041	-5,0913
ID: 0777	**633**	0,0281544	1,177	0,79355	1,012	0,31772075	1,162					1,173	1,159	0,997	16,2487
ID: 0778	**634**	0,50956853	0,966	0,16728	0,945	0,66754242	1,022					1,091	1,154	1,129	2,2143
ID: 0779	**635**	0,41976504	0,973	0,19872	0,922	0,38197917	1,055					1,074	1,165	1,104	5,5254
ID: 0780	**636**	0,74019377	0,991	0,16283	1,060	0,09821554	0,935					1,188	1,121	1,199	-6,5054
ID: 0781	**637**	0,9706103	0,976	0,65443	0,961	0,83091661	1,015					1,139	1,185	1,167	1,5424
ID: 0782	**638**	0,85532048	0,966	0,84714	0,988	0,76857506	0,978					1,008	1,020	1,043	-2,2052
ID: 0783	**639**	0,38702222	0,942	0,1052	0,898	0,3794758	1,049					0,992	1,105	1,053	4,9383
ID: 0785	**640**	0,3067807	0,933	0,14554	0,899	0,39639953	1,038					0,983	1,094	1,054	3,7951
ID: 0786	**641**	0,26914906	0,941	0,22946	0,936	0,89669909	1,005					1,171	1,251	1,245	0,4819
ID: 0787	**642**	0,04392311	0,930	0,0069	0,915	0,63915744	1,017					1,064	1,163	1,144	1,6608
ID: 0788	**643**	0,78746703	0,980	0,57504	0,873	0,55059585	1,123					0,983	1,126	1,003	12,2632
ID: 0789	**644**	0,74494441	1,020	0,68059	0,984	0,50105159	1,037					1,173	1,192	1,150	3,6522
ID: 0790	**645**	0,29911522	1,169	0,80082	1,040	0,43482551	1,124					0,983	0,945	0,841	12,3662
ID: 0791	**646**	0,62773797	1,027	0,54576	1,019	0,82766113	1,008					1,131	1,110	1,101	0,8174
ID: 0792	**647**	0,28378995	1,055	0,27524	1,043	0,82013394	1,012					1,176	1,128	1,115	1,1659
ID: 0793	**648**	0,05523587	0,577	0,96749	1,013	0,04812098	0,570					0,777	0,767	1,346	-43,0163
ID: 0795	**649**	0,72015132	0,880	0,72145	0,932	0,92220344	0,944					0,807	0,866	0,917	-5,5616
ID: 0796	**650**	0,75497482	0,855	0,25443	0,921	0,98035291	0,928					0,949	1,030	1,110	-7,2072
ID: 0797	**651**	0,72014827	0,952	0,32835	0,890	0,52316315	1,069					1,043	1,172	1,096	6,9343
ID: 0798	**652**	0,00029419	0,580	0,00122	0,523	0,4572912	1,109					0,711	1,360	1,226	10,9299
ID: 0799	**653**	0,01021517	1,239	0,06563	1,181	0,63081455	1,049					1,163	0,985	0,939	4,8988
ID: 0800	**654**	0,90238929	1,021	0,62494	0,948	0,52173169	1,077					0,952	1,004	0,932	7,7253
ID: 0802	**655**	0,35154351	0,853	0,40281	0,880	0,88201823	0,969					0,963	1,094	1,129	-3,1001
ID: 0803	**656**	0,15684745	0,787	0,90792	0,975	0,28558832	0,808					0,728	0,747	0,925	-19,2432
ID: 0805	**657**	0,0400641	0,730	0,31966	0,793	0,5798549	0,920					0,885	1,116	1,213	-7,9967
								Agrotis_qualite_rep_c8395|ORF4 length 445 aa, 1338 bp, from 109..1446 of	6	RBNS5_HUMAN	rabenosyn-5 [Papilio xuthus]	1,319	0,967	1,191	-18,8077
								Agrotis_qualite_rep_c4879|ORF3 length 268 aa, 807 bp, from 42..848 of	6	AL1B1_HUMAN	aldehyde dehydrogenase X, mitochondrial-like [Amyelois transitella]	1,319	0,967	1,191	-18,8077
								Agrotis_qualite_rep_c275|ORF5 length 543 aa, 1632 bp, from 98..1729 of	5	Q2F5T4_BOMMO	ATP synthase [Bombyx mori]	1,319	0,967	1,191	-18,8077

ID: 0807	**658**	0,26483625	1,107	0,01002	1,364	0,01983489	0,812	Agrotis_qualite_rep_c1211|ORF2 length 210 aa, 633 bp, from 1..633 of	4	KGM_213554	Palmitoyl-protein thioesterase [Danaus plexippus plexippus]	1,319	0,967	1,191	-18,8077
								Agrotis_qualite_rep_c275|ORF30 length 59 aa, 180 bp, from 1603..1782 of	3	Q2F5T4_BOMMO	ATP synthase [Bombyx mori]	1,319	0,967	1,191	-18,8077
								Agrotis_qualite_rep_c2828|ORF3 length 282 aa, 849 bp, from 168..1016 of Agrotis_qualite_rep_c2828	2	MMSA_HUMAN	methylmalonate-semialdehyde dehydrogenase [acylating], mitochondrial isoform X2	1,319	0,967	1,191	-18,8077
								Agrotis_qualite_rep_c2828|ORF17 length 277 aa, 834 bp, from 914..1747 of Agrotis_qualite_rep_c2828	2	MMSA_HUMAN	methylmalonate-semialdehyde dehydrogenase [acylating], mitochondrial isoform X2	1,319	0,967	1,191	-18,8077
ID: 0808	**659**	0,00955716	1,145	0,16929	1,139	0,9610154	1,006					1,213	1,065	1,059	0,5666
ID: 0809	**660**	0,58155123	1,046	0,5864	1,057	0,88300294	0,989					1,193	1,129	1,141	-1,0517
ID: 0810	**661**	0,05087733	1,173	0,0484	1,204	0,72115185	0,974					1,098	0,912	0,936	-2,5641
ID: 0811	**662**	0,00214931	0,684	0,04778	0,735	0,53714971	0,930					0,85	1,156	1,243	-6,9992
ID: 0812	**663**	0,36506839	0,971	0,04289	0,923	0,19606665	1,053					1,089	1,180	1,121	5,2632
ID: 0813	**664**	0,76583198	1,024	0,14316	1,187	0,3230892	0,863					0,884	0,745	0,863	-13,6732
ID: 0814	**665**	0,60424542	1,035	0,19509	1,089	0,45334582	0,950					0,974	0,894	0,941	-4,9947
ID: 0815	**666**	0,81371656	1,016	0,46552	1,054	0,61546689	0,963					0,971	0,921	0,956	-3,6611
ID: 0816	**667**	0,45130629	0,948	0,14667	0,930	0,75607163	1,019					1,031	1,109	1,088	1,9301
ID: 0817	**668**	0,45863553	0,958	0,03823	0,889	0,16024134	1,077					1,141	1,283	1,191	7,7246
ID: 0818	**669**	0,46975657	1,054	0,29889	1,097	0,69343781	0,961					0,954	0,87	0,905	-3,8674
ID: 0819	**670**	0,63930091	1,062	0,17678	1,191	0,12511836	0,892					1,123	0,943	1,057	-10,7852
ID: 0820	**671**	0,14235952	1,066	0,24076	1,076	0,85780324	0,991					1,157	1,075	1,085	-0,9217
ID: 0821	**672**	0,4588592	0,935	0,54994	0,962	0,75797315	0,971					1,242	1,291	1,329	-2,8593
ID: 0822	**673**	0,04665322	1,226	0,95886	1,001	0,07202045	1,225					1,194	1,193	0,974	22,4846
ID: 0823	**674**	0,52102343	1,045	0,49963	1,044	0,95808167	1,001					1,153	1,104	1,103	0,0907
ID: 0824	**675**	0,09480188	0,865	0,87256	0,976	0,19892141	0,886					1,054	1,080	1,219	-11,4028
ID: 0825	**676**	0,2991491	0,976	0,19201	0,959	0,48531079	1,018					1,104	1,151	1,131	1,7683
ID: 0826	**677**	0,12692073	0,952	0,2973	0,955	0,92883359	0,997					1,114	1,167	1,170	-0,2564
ID: 0827	**678**	0,75815148	1,022	0,96242	1,000	0,82250821	1,022					1,083	1,083	1,060	2,1698
ID: 0828	**679**	0,32488341	0,967	0,81285	0,990	0,54396165	0,977					1,128	1,139	1,166	-2,3156
ID: 0829	**680**	0,58552024	1,038	0,30307	0,826	0,15105885	1,256					0,931	1,127	0,897	25,6410
ID: 0830	**681**	0,81909424	0,988	0,81703	0,981	0,941629	1,007					0,927	0,945	0,938	0,7463
ID: 0831	**682**	0,16022432	0,944	0,19689	0,858	0,38276737	1,099					0,952	1,109	1,009	9,9108
ID: 0832	**683**	0,76793935	0,976	0,25787	0,860	0,29214075	1,136					0,826	0,961	0,846	13,5934
ID: 0833	**684**	0,27742693	1,115	0,03771	1,218	0,48155534	0,915					1,146	0,941	1,028	-8,4630

ID: 0834	**685**	0,3291606	1,039	0,7407	0,993	0,19628458	1,047					1,159	1,167	1,115	4,6637
ID: 0835	**686**	0,24485677	1,040	0,28955	1,049	0,78898332	0,991					1,021	0,973	0,982	-0,9165
ID: 0836	**687**	0,07114572	1,363	0,49847	0,851	0,03260594	1,601					0,991	1,164	0,727	60,1100
ID: 0837	**688**	0,52439496	1,023	0,39052	1,043	0,67927641	0,981					1,118	1,072	1,093	-1,9213
ID: 0838	**689**	0,9142142	0,984	0,51256	0,952	0,67811213	1,034					0,809	0,85	0,822	3,4063
ID: 0839	**690**	0,27592498	1,065	0,02265	1,185	0,15366923	0,899					0,979	0,826	0,919	-10,1197
ID: 0840	**691**	0,93219951	1,005	0,75677	1,018	0,83101656	0,987					1,069	1,050	1,064	-1,3158
ID: 0841	**692**	0,5401995	1,034	0,43628	0,949	0,22873435	1,089					1,067	1,124	1,032	8,9147
ID: 0842	**693**	0,2741239	0,918	0,28131	0,925	0,93549224	0,992					1,215	1,314	1,324	-0,7553
ID: 0843	**694**	0,91738858	0,993	0,43885	1,059	0,33849479	0,938					1,004	0,948	1,011	-6,2315
ID: 0844	**695**	0,85515231	0,989	0,76951	0,985	0,86839397	1,004					1,075	1,091	1,087	0,3680
ID: 0845	**696**	0,08125219	1,190	0,31087	0,899	0,00751398	1,323					1,236	1,375	1,039	32,3388
ID: 0847	**697**	0,28769167	1,303	0,21272	1,380	0,81564583	0,944					1,283	0,93	0,985	-5,5838
ID: 0848	**698**	0,85952221	0,976	0,89004	0,989	0,94958909	0,986					0,997	1,008	1,022	-1,3699
ID: 0850	**699**	0,60330641	1,033	0,3075	1,069	0,62645655	0,966					1,072	1,003	1,038	-3,3719
ID: 0852	**700**	0,83292567	0,978	0,87832	0,977	0,97891017	1,001					0,933	0,955	0,954	0,1048
ID: 0853	**701**	0,85005274	1,013	0,89566	0,985	0,76699783	1,029					1,030	1,046	1,017	2,8515
ID: 0854	**702**	0,63762825	0,877	0,09878	0,700	0,25872877	1,254					0,608	0,869	0,693	25,3968
ID: 0855	**703**	0,79949238	1,021	0,78407	0,982	0,60955065	1,039					0,939	0,956	0,92	3,9130
ID: 0857	**704**	0,99920579	1,003	0,20071	0,920	0,08732625	1,090					1,083	1,177	1,080	8,9815
ID: 0859	**705**	0,2518341	1,211	0,17223	1,248	0,93031794	0,970					0,856	0,686	0,707	-2,9703
ID: 0860	**706**	0,0205603	1,769	0,06728	1,642	0,79686815	1,078					1,617	0,985	0,914	7,7681
ID: 0861	**707**	0,15925253	1,199	0,03874	1,343	0,36279023	0,893					0,849	0,632	0,708	-10,7345
ID: 0862	**708**	0,7164915	0,986	0,80156	0,984	0,99464436	1,002					0,995	1,011	1,009	0,1982
ID: 0863	**709**	0,00061706	0,559	0,00348	0,472	0,24719672	1,185					0,608	1,289	1,088	18,4743
ID: 0864	**710**	0,93795814	1,001	0,60709	0,941	0,59283949	1,063					1,125	1,195	1,124	6,3167
ID: 0865	**711**	0,25082073	1,131	0,02834	1,374	0,2334631	0,823					0,926	0,674	0,819	-17,7045
ID: 0866	**712**	0,4301878	1,091	0,13305	1,218	0,51580943	0,895					0,927	0,761	0,85	-10,4706
ID: 0867	**713**	0,80028446	0,972	0,72946	0,978	0,99199304	0,994					1,072	1,096	1,103	-0,6346
ID: 0868	**714**	0,2619905	0,872	0,45442	0,894	0,83575438	0,976					0,967	1,082	1,109	-2,4346
ID: 0869	**715**	0,87427594	0,964	0,09322	1,118	0,29734534	0,862					0,881	0,788	0,914	-13,7856
ID: 0871	**716**	0,62375465	0,976	0,73365	1,014	0,51734802	0,963					0,95	0,937	0,973	-3,6999
ID: 0872	**717**	0,43105295	1,110	0,04548	1,262	0,53374951	0,879					0,746	0,591	0,672	-12,0536
ID: 0873	**718**	0,4304396	1,042	0,80941	0,981	0,37228916	1,062					1,070	1,091	1,027	6,2317
ID: 0874	**719**	0,60917483	0,945	0,80574	0,960	0,85438606	0,985					0,919	0,957	0,972	-1,5432
ID: 0875	**720**	0,50566309	1,028	0,13148	1,069	0,43637862	0,962					1,054	0,986	1,025	-3,8049
ID: 0876	**721**	0,65852588	1,018	0,121	1,057	0,43053007	0,964					1,062	1,005	1,043	-3,6433
ID: 0877	**722**	0,80747076	0,977	0,10274	0,913	0,3534507	1,070					1,008	1,104	1,032	6,9767
ID: 0878	**723**	0,18199273	0,925	0,58852	0,941	0,73878563	0,984					0,856	0,91	0,925	-1,6216
ID: 0879	**724**	0,46359471	0,947	0,83202	0,973	0,71030415	0,973					0,939	0,965	0,992	-2,7218

ID: 0880	**725**	0,78544334	1,019	0,35456	1,059	0,73609141	0,962					0,965	0,911	0,947	-3,8015
ID: 0881	**726**	0,799469	1,023	0,13837	1,139	0,16906633	0,898					1,038	0,911	1,015	-10,2463
ID: 0882	**727**	0,1482014	1,326	0,01265	1,536	0,6571607	0,863					1,407	0,916	1,061	-13,6664
ID: 0883	**728**	0,08124713	0,788	0,00418	0,753	0,61349088	1,046					0,9	1,195	1,142	4,6410
ID: 0885	**729**	0,92995116	0,969	0,5664	1,281	0,48401191	0,757					0,538	0,42	0,555	-24,3243
ID: 0886	**730**	0,29072555	0,829	0,42202	0,837	0,91192022	0,991					0,626	0,748	0,755	-0,9272
ID: 0887	**731**	0,64107956	0,984	0,26411	0,947	0,35611846	1,040					1,141	1,205	1,159	3,9689
ID: 0888	**732**	0,37011822	0,892	0,16709	1,185	0,01866816	0,753					0,903	0,762	1,012	-24,7036
ID: 0889	**733**	0,3571276	1,210	0,16824	1,342	0,64105603	0,902					0,765	0,57	0,632	-9,8101
ID: 0890	**734**	0,2211015	1,088	0,24318	1,070	0,69568716	1,016					1,127	1,053	1,036	1,6409
ID: 0891	**735**	0,7117988	0,963	0,24222	0,887	0,36398385	1,086					0,837	0,944	0,869	8,6306
ID: 0892	**736**	0,80239933	1,048	0,09383	1,577	0,16772607	0,664					0,571	0,362	0,545	-33,5780
ID: 0893	**737**	0,53206539	0,953	0,62282	0,972	0,81201087	0,981					1,084	1,115	1,137	-1,9349
ID: 0894	**738**	0,8415935	1,058	0,07423	1,983	0,16023297	0,534					0,837	0,422	0,791	-46,6498
ID: 0895	**739**	0,23819553	1,154	0,01245	1,400	0,14166253	0,824					1,156	0,826	1,002	-17,5649
ID: 0896	**740**	0,4805615	0,922	0,16691	1,170	0,04944585	0,788					0,921	0,787	0,999	-21,2212
ID: 0897	**741**	0,91605216	0,982	0,16855	1,225	0,14680508	0,801					1,066	0,87	1,086	-19,8895
ID: 0898	**742**	0,23661571	1,016	0,74165	0,984	0,40621014	1,033					1,121	1,139	1,103	3,2638
ID: 0899	**743**	0,59723667	0,908	0,01888	1,599	0,00654017	0,568	Agrotis_qualite_rep_c334|ORF5 length 395 aa, 1188 bp, from 225..1412 of	13	A0A088M9K3_AGRSE	Fatty alcohol acetyltransferase Agrotis segetum (Turnip moth)	0,873	0,546	0,961	-43,1842
								Agrotis_qualite_rep_c820|ORF15 length 300 aa, 903 bp, from 620..1522 of Agrotis_qualite_rep_c820	9	I4DK27_PAPXU	Mitochondrial processing peptidase beta subunit Papilio xuthus (Asian swallowtail butterfly)	0,873	0,546	0,961	-43,1842
ID: 0900	**744**	0,521839	1,045	0,28431	1,091	0,56843966	0,958					0,936	0,858	0,896	-4,2411
ID: 0901	**745**	0,83591204	0,990	0,67218	1,016	0,59448136	0,974					0,953	0,938	0,963	-2,5961
ID: 0902	**746**	0,04507232	1,280	0,6423	0,636	0,243525	2,014					0,923	1,452	0,721	101,3870
ID: 0903	**747**	0,31028945	0,850	0,26084	0,836	0,88119052	1,016					0,752	0,899	0,885	1,5819
ID: 0905	**748**	0,4598275	0,963	0,82011	1,013	0,3694802	0,951					1,019	1,006	1,058	-4,9149
ID: 0906	**749**	0,72761732	1,031	0,42437	1,108	0,51062932	0,930					1,109	1,001	1,076	-6,9703
ID: 0907	**750**	0,59585872	0,936	0,87791	1,047	0,39926121	0,895					1,074	1,026	1,147	-10,5493
ID: 0908	**751**	0,83455516	0,990	0,78272	1,011	0,61924814	0,980					1,113	1,101	1,124	-2,0463
ID: 0909	**752**	0,21588127	0,905	0,14826	0,854	0,32329828	1,060					0,864	1,012	0,955	5,9686
ID: 0910	**753**	0,64734346	1,048	0,36849	1,088	0,83719012	0,963					0,942	0,866	0,899	-3,6707
ID: 0911	**754**	0,52502162	0,918	0,78027	0,943	0,81445303	0,973					0,997	1,057	1,086	-2,6703
ID: 0912	**755**	0,05381066	0,839	0,35094	1,104	0,01146407	0,761					1,119	1,014	1,333	-23,9310
ID: 0913	**756**	0,7962836	0,982	0,82414	0,987	0,96387742	0,995					0,919	0,931	0,936	-0,5342
ID: 0914	**757**	0,94286571	1,001	0,06727	0,971	0,29760049	1,031					0,941	0,969	0,94	3,0851
ID: 0915	**758**	0,05696558	1,378	0,01166	1,738	0,19897072	0,793					1,192	0,686	0,865	-20,6936

ID: 0916	**759**	0,81433399	0,985	0,35021	1,076	0,18590975	0,916					0,995	0,925	1,010	-8,4158
ID: 0917	**760**	0,93588238	0,972	0,83216	1,008	0,83150605	0,964					0,895	0,888	0,921	-3,5831
ID: 0918	**761**	0,95717331	1,001	0,48878	1,015	0,68087333	0,986					0,937	0,923	0,936	-1,3889
ID: 0919	**762**	0,2016343	1,162	0,18801	1,172	0,96752716	0,991					0,884	0,754	0,761	-0,9198
ID: 0920	**763**	0,72750622	0,974	0,52999	1,130	0,11175678	0,862					0,757	0,67	0,777	-13,7709
ID: 0921	**764**	0,36634103	1,059	0,34138	1,143	0,48155538	0,926					1,064	0,931	1,005	-7,3632
ID: 0922	**765**	0,00041763	0,299	0,00016	0,278	0,59447562	1,075					0,315	1,132	1,053	7,5024
ID: 0923	**766**	0,95841899	0,987	0,42295	0,843	0,4507147	1,172					1,156	1,372	1,171	17,1648
ID: 0924	**767**	0,82543475	0,935	0,55515	0,881	0,74559523	1,061					1,035	1,175	1,107	6,1427
ID: 0925	**768**	0,32677962	0,963	0,76602	0,990	0,23949738	0,973					1,133	1,145	1,177	-2,7188
ID: 0926	**769**	0,29830314	1,070	0,82613	1,018	0,4522819	1,051					1,178	1,157	1,101	5,0863
ID: 0928	**770**	0,70954993	1,013	0,21107	0,938	0,1139065	1,080					1,060	1,130	1,046	8,0306
ID: 0929	**771**	0,82158917	0,993	0,83324	0,988	0,94419722	1,005					1,071	1,084	1,079	0,4634
ID: 0933	**772**	0,91061585	0,929	0,21832	1,306	0,45439561	0,711					0,921	0,705	0,991	-28,8597
ID: 0934	**773**	0,05237962	1,188	0,80236	0,980	0,0446352	1,213					1,106	1,129	0,931	21,2675
ID: 0935	**774**	0,87433703	0,990	0,3213	1,113	0,22584857	0,890					0,988	0,888	0,998	-11,0220
ID: 0936	**775**	0,27355047	0,887	0,98867	1,014	0,12971799	0,875					0,866	0,854	0,976	-12,5000
ID: 0938	**776**	0,07248537	0,841	0,94087	0,934	0,41218782	0,901					0,844	0,904	1,003	-9,8704
ID: 0939	**777**	0,78181003	0,987	0,22831	0,892	0,23309794	1,107					1,044	1,171	1,058	10,6805
ID: 0940	**778**	0,85922413	0,984	0,10717	0,875	0,18753772	1,125					1,090	1,246	1,108	12,4549
ID: 0942	**779**	0,59741904	1,017	0,64873	0,921	0,51172725	1,104					1,138	1,235	1,119	10,3664
ID: 0943	**780**	0,08901086	1,221	0,06584	1,264	0,62039384	0,966					1,106	0,875	0,906	-3,4216
ID: 0944	**781**	0,44303792	0,945	0,03504	0,741	0,03712241	1,276					0,918	1,239	0,971	27,6004
ID: 0945	**782**	0,33149849	1,042	0,66638	1,021	0,75995543	1,021					1,063	1,041	1,020	2,0588
ID: 0946	**783**	0,41412723	1,177	0,09208	2,030	0,45782198	0,580					0,619	0,305	0,526	-42,0152
ID: 0947	**784**	0,04492895	1,260	0,08671	1,313	0,67042225	0,960					1,247	0,95	0,99	-4,0404
ID: 0948	**785**	0,59160942	1,283	0,46817	1,413	0,7080004	0,908					0,725	0,513	0,565	-9,2035
ID: 0949	**786**	0,58165184	1,688	0,20331	3,429	0,31834404	0,492					0,336	0,098	0,199	-50,7538
ID: 0952	**787**	0,54603435	1,278	0,34936	1,466	0,5785817	0,871					0,764	0,521	0,598	-12,8763
ID: 0953	**788**	0,47292053	0,890	0,17792	0,855	0,62797608	1,042					0,941	1,101	1,057	4,1627
ID: 0954	**789**	0,21134096	1,114	0,61432	0,955	0,14125213	1,167					0,986	1,033	0,885	16,7232
ID: 0955	**790**	0,12626029	1,208	0,45583	1,091	0,35180545	1,106					1,181	1,082	0,978	10,6339
ID: 0956	**791**	0,26370069	0,936	0,35436	0,938	0,95212127	0,998					1,001	1,067	1,069	-0,1871
ID: 0957	**792**	0,7689675	1,024	0,73671	0,934	0,59974622	1,096					1,072	1,148	1,047	9,6466
ID: 0958	**793**	0,32181801	1,149	0,46848	1,097	0,59307677	1,047					1,151	1,049	1,002	4,6906
ID: 0959	**794**	0,77902695	1,295	0,20948	3,862	0,26063121	0,335					0,448	0,116	0,346	-66,4740
ID: 0960	**795**	0,87283583	1,130	0,30918	2,037	0,31968801	0,555					0,495	0,243	0,438	-44,5205
ID: 0961	**796**	0,86093109	1,012	0,30957	0,883	0,23133039	1,146					0,947	1,073	0,936	14,6368
ID: 0962	**797**	0,85774094	0,984	0,82347	1,018	0,70121081	0,966					1,018	1,000	1,035	-3,3816
ID: 0964	**798**	0,33612281	0,913	0,25161	0,798	0,39046707	1,145					0,939	1,177	1,028	14,4942

ID: 0965	**799**	0,04529776	1,049	0,1772	0,973	0,01744499	1,078					1,085	1,115	1,034	7,8337
ID: 0966	**800**	0,91333912	0,892	0,27436	0,373	0,29942547	2,391					0,726	1,946	0,814	139,0663
ID: 0967	**801**	0,2783516	0,903	0,34382	0,781	0,59068734	1,156					0,978	1,252	1,083	15,6048
ID: 0968	**802**	0,55441279	0,982	0,05969	0,922	0,00781804	1,064					1,066	1,156	1,086	6,4457
ID: 0969	**803**	0,22680805	1,300	0,31755	1,260	0,93448706	1,032					0,857	0,68	0,659	3,1866
ID: 0971	**804**	0,53209347	1,035	0,70686	0,852	0,55148452	1,215					1,045	1,227	1,010	21,4851
ID: 0972	**805**	0,00180227	0,795	0,00062	0,682	0,04864136	1,166					0,892	1,308	1,122	16,5775
ID: 0973	**806**	0,15915534	0,859	0,44733	0,914	0,59719246	0,939					0,929	1,016	1,082	-6,0998
ID: 0974	**807**	0,25939114	0,880	0,30224	0,862	0,90982339	1,020					1,046	1,213	1,189	2,0185
ID: 0975	**808**	0,4650766	1,095	0,67629	1,061	0,95955842	1,032					1,203	1,134	1,099	3,1847
ID: 0976	**809**	0,80659389	1,047	0,88608	0,992	0,58024171	1,056					0,997	1,005	0,952	5,5672
ID: 0977	**810**	0,48382309	0,965	0,52876	0,963	0,98200749	1,002					1,044	1,084	1,082	0,1848
ID: 0978	**811**	0,46907404	1,122	0,16132	1,327	0,66756027	0,845					0,876	0,66	0,781	-15,4930
ID: 0979	**812**	0,51893291	0,972	0,20797	0,946	0,54371001	1,028					1,010	1,068	1,039	2,7911
ID: 0980	**813**	0,06477291	0,691	0,14929	0,723	0,66966453	0,956					0,892	1,234	1,291	-4,4152
ID: 0981	**814**	0,95841451	0,908	0,26393	0,412	0,28346876	2,202					0,719	1,744	0,792	120,2020
ID: 0983	**815**	0,0909983	0,785	0,12923	0,701	0,54484725	1,120					0,871	1,243	1,110	11,9820
ID: 0985	**816**	0,18431801	1,438	0,16066	1,653	0,604183	0,870					1,296	0,784	0,901	-12,9856
ID: 0986	**817**	0,18579024	1,324	0,12228	1,460	0,76587165	0,906					1,288	0,882	0,973	-9,3525
ID: 0987	**818**	0,97261761	0,953	0,42127	0,495	0,40228045	1,926					0,806	1,629	0,846	92,5532
ID: 0988	**819**	0,54725134	1,086	0,15362	1,178	0,76054452	0,923					0,955	0,811	0,879	-7,7361
ID: 0989	**820**	0,31679691	1,150	0,19676	1,189	0,85268944	0,967					1,014	0,853	0,882	-3,2880
ID: 0990	**821**	0,13770107	0,794	0,17215	0,750	0,82259931	1,058					0,832	1,109	1,048	5,8206
ID: 0991	**822**	0,99462451	0,988	0,30686	0,909	0,45701397	1,087					0,896	0,986	0,907	8,7100
ID: 0992	**823**	0,57793524	0,945	0,79513	0,973	0,79420058	0,972					1,003	1,031	1,061	-2,8275
ID: 0993	**824**	0,40514389	0,436	0,13925	0,381	0,6336236	1,144					0,541	1,420	1,241	14,4239
ID: 0994	**825**	0,63281796	0,904	0,39134	1,143	0,26198095	0,791					0,909	0,795	1,005	-20,8955
ID: 0995	**826**	0,99615546	1,001	0,95109	0,996	0,94161376	1,005					1,036	1,040	1,035	0,4831
ID: 0996	**827**	0,19279986	1,251	0,08465	1,278	0,96321202	0,979					0,887	0,694	0,709	-2,1157
ID: 0997	**828**	0,16247384	0,686	0,20404	0,668	0,9570215	1,027					0,774	1,159	1,129	2,6572
ID: 0998	**829**	0,26989274	1,032	0,4844	0,981	0,10316004	1,052					1,110	1,132	1,076	5,2045
ID: 0999	**830**	0,59956464	0,979	0,89197	1,006	0,47252354	0,973					1,047	1,041	1,070	-2,7103
ID: 1000	**831**	0,72213706	0,982	0,89372	0,995	0,80329473	0,987					0,958	0,963	0,976	-1,3320
ID: 1001	**832**	0,317276	0,830	0,2844	0,872	0,85318801	0,952					0,865	0,992	1,042	-4,7985
ID: 1002	**833**	0,3320138	0,944	0,07856	0,901	0,28921615	1,048					0,89	0,988	0,943	4,7720
ID: 1004	**834**	0,66232731	1,028	0,88389	1,004	0,89084338	1,024					1,021	1,017	0,993	2,4169
								Agrotis_qualite_rep_c1149| ORF2 length 393 aa, 1182 bp,<br>from 77..1258 of	17		unknown / non identified by blast research	1,126	0,826	1,152	-28,2986

ID: 1005	**835**	0,79309447	0,977	0,0068	1,363	0,00333438	0,717	Agrotis_qualite_c18204|ORF 14 length 148 aa, 447 bp, from 705..1151 of	3			1,126	0,826	1,152	-28,2986
								Agrotis_qualite_rep_c18905|ORF5 length 93 aa, 282 bp, from 50..331 of	2			1,126	0,826	1,152	-28,2986
ID: 1006	**836**	0,9843355	1,016	0,92392	1,034	0,85903044	0,983					0,894	0,865	0,88	-1,7045
ID: 1007	**837**	0,67721487	1,033	0,51317	1,072	0,9676448	0,964					1,112	1,037	1,076	-3,6245
ID: 1008	**838**	0,94180876	1,002	0,56415	1,015	0,7293521	0,987					1,051	1,035	1,049	-1,3346
ID: 1009	**839**	0,21335854	1,040	0,77141	0,993	0,03829744	1,047					1,039	1,046	0,999	4,7047
ID: 1010	**840**	0,18987507	1,116	0,02677	1,257	0,04531155	0,888					1,144	0,91	1,025	-11,2195
ID: 1011	**841**	0,17267075	0,416	0,15077	0,371	0,83129775	1,122					0,445	1,199	1,069	12,1609
ID: 1012	**842**	0,59737943	0,983	0,5976	0,982	0,95995102	1,001					1,113	1,133	1,132	0,0883
ID: 1013	**843**	0,75288992	1,020	0,94022	0,993	0,81224449	1,027					1,066	1,073	1,045	2,6794
ID: 1014	**844**	0,01494495	1,345	0,00094	1,635	0,05202464	0,823					1,419	0,868	1,055	-17,7251
ID: 1015	**845**	0,84752277	0,957	0,42608	0,943	0,79744169	1,014					0,881	0,934	0,921	1,4115
ID: 1016	**846**	0,58922491	1,063	0,22206	1,152	0,70607393	0,923					1,116	0,969	1,050	-7,7143
ID: 1017	**847**	0,55092465	0,946	0,6556	1,068	0,08519896	0,886					1,198	1,122	1,266	-11,3744
ID: 1018	**848**	0,21424423	1,079	0,78645	1,015	0,40060183	1,063					0,942	0,928	0,873	6,3001
ID: 1019	**849**	0,20645843	1,066	0,14007	1,065	0,94610361	1,001					1,141	1,071	1,070	0,0935
ID: 1020	**850**	0,65916996	0,984	0,54829	0,914	0,59348593	1,077					1,038	1,136	1,055	7,6777
ID: 1021	**851**	0,30151692	0,757	0,25865	0,750	0,95136369	1,009					0,931	1,241	1,230	0,8943
ID: 1023	**852**	0,90403835	1,012	0,80393	1,022	0,61266405	0,990					1,010	0,988	0,998	-1,0020
ID: 1024	**853**	0,51200032	1,053	0,3986	1,071	0,85463557	0,983					1,065	0,994	1,011	-1,6815
ID: 1025	**854**	0,73773008	1,013	0,87704	1,007	0,91068846	1,007					1,075	1,068	1,061	0,6598
ID: 1026	**855**	0,48065728	0,957	0,55225	0,963	0,92635808	0,994					1,067	1,108	1,115	-0,6278
ID: 1027	**856**	0,89823726	1,008	0,84364	1,012	0,95103448	0,996					1,050	1,038	1,042	-0,3839
ID: 1029	**857**	0,03108317	1,200	0,00964	1,262	0,55142641	0,951					1,121	0,888	0,934	-4,9251
ID: 1030	**858**	0,90713719	0,997	0,97832	0,999	0,93233002	0,998					1,082	1,083	1,085	-0,1843
ID: 1031	**859**	0,24812672	1,071	0,089	1,127	0,1950646	0,950					1,295	1,149	1,209	-4,9628
ID: 1032	**860**	0,15402589	1,178	0,51456	0,930	0,03142124	1,266					0,915	0,984	0,777	26,6409
ID: 1033	**861**	0,63944543	1,032	0,11969	1,115	0,22952766	0,925					1,241	1,113	1,203	-7,4813
ID: 1034	**862**	0,46659207	1,040	0,34907	1,064	0,61493072	0,977					1,392	1,308	1,339	-2,3152
ID: 1035	**863**	0,62579028	1,019	0,74881	0,987	0,45649185	1,032					1,082	1,096	1,062	3,2015
ID: 1036	**864**	0,18257934	1,045	0,9629	1,002	0,27495347	1,043					1,260	1,258	1,206	4,3118
ID: 1037	**865**	0,58201687	0,987	0,00609	0,929	0,03080048	1,063					1,010	1,087	1,023	6,2561
ID: 1038	**866**	0,47322369	1,184	0,50615	0,705	0,22129736	1,678					1,044	1,480	0,882	67,8005
ID: 1039	**867**	0,28228014	1,117	0,04829	1,275	0,3930153	0,876					1,090	0,855	0,976	-12,3975
ID: 1040	**868**	0,33138016	1,071	0,95849	0,998	0,52657777	1,073					1,087	1,089	1,015	7,2906
ID: 1041	**869**	0,13131382	1,138	0,43045	1,090	0,71302645	1,045					1,201	1,102	1,055	4,4550

ID: 1043	**870**	0,83269563	0,884	0,36165	0,510	0,42875927	1,731					0,766	1,501	0,867	73,1257
ID: 1044	**871**	0,92687617	0,874	0,36002	0,483	0,41100083	1,810					0,758	1,569	0,867	80,9689
ID: 1045	**872**	0,90281151	0,997	0,82219	0,976	0,8379053	1,022					0,961	0,985	0,964	2,1784
ID: 1046	**873**	0,71131267	1,026	0,66265	1,030	0,98509423	0,996					1,112	1,080	1,084	-0,3690
ID: 1047	**874**	0,14847828	0,918	0,07497	0,731	0,14408863	1,256					1,016	1,390	1,107	25,5646
ID: 1048	**875**	0,78992424	1,031	0,75664	0,944	0,58982372	1,093					1,259	1,334	1,221	9,2547
ID: 1051	**876**	0,34407752	1,090	0,32032	1,091	0,9752365	0,999					1,082	0,992	0,993	-0,1007
ID: 1052	**877**	0,99638467	0,999	0,16174	0,908	0,14469251	1,100					1,099	1,210	1,100	10,0000
ID: 1053	**878**	0,33608437	1,197	0,05051	1,473	0,26377608	0,813					1,360	0,923	1,136	-18,7500
ID: 1054	**879**	0,75145095	1,024	0,75945	1,024	0,9591817	1,000					1,219	1,190	1,190	0,0000
ID: 1055	**880**	0,88734352	0,979	0,30514	1,172	0,25074365	0,836					1,138	0,971	1,162	-16,4372
ID: 1056	**881**	0,44755314	0,931	0,62806	0,856	0,86109647	1,088					0,923	1,078	0,991	8,7790
ID: 1057	**882**	0,4877335	1,044	0,33602	1,100	0,55361152	0,949					1,481	1,346	1,418	-5,0776
ID: 1058	**883**	0,90228272	1,033	0,38703	0,581	0,29635565	1,780					0,928	1,598	0,898	77,9510
ID: 1059	**884**	0,30822212	0,972	0,43787	0,973	0,95551815	0,999					1,112	1,143	1,144	-0,0874
ID: 1060	**885**	0,46512964	1,045	0,37827	0,954	0,09722735	1,095					1,055	1,106	1,010	9,5050
ID: 1062	**886**	0,30574481	1,057	0,55	1,042	0,90577047	1,014					1,301	1,248	1,231	1,3810
ID: 1063	**887**	0,1579141	0,845	0,91155	1,005	0,18718378	0,841					1,086	1,081	1,285	-15,8755
ID: 1064	**888**	0,57800985	1,106	0,88562	1,005	0,43269162	1,101					1,088	1,083	0,984	10,0610
ID: 1065	**889**	0,40099337	1,052	0,49043	1,052	0,96980561	1,000					1,280	1,217	1,217	0,0000
ID: 1066	**890**	0,64797037	1,161	0,85727	1,106	0,68314195	1,050					1,094	0,989	0,942	4,9894
ID: 1067	**891**	0,4750631	0,970	0,9265	0,988	0,71553994	0,982					1,038	1,051	1,070	-1,7757
ID: 1068	**892**	0,26523646	0,876	0,23913	0,862	0,8780578	1,016					1,032	1,197	1,178	1,6129
ID: 1069	**893**	0,4586891	1,066	0,37069	1,121	0,6827969	0,952					1,301	1,161	1,220	-4,8361
ID: 1070	**894**	0,1323019	1,144	0,18164	1,186	0,72805388	0,965					1,335	1,126	1,167	-3,5133
ID: 1071	**895**	0,64035674	0,855	0,0735	0,743	0,46342925	1,151					0,828	1,114	0,968	15,0826
ID: 1072	**896**	0,42599435	1,046	0,5586	1,021	0,68760524	1,025					1,042	1,021	0,996	2,5100
ID: 1073	**897**	0,26642415	1,139	0,40413	1,117	0,96257634	1,020					1,260	1,128	1,106	1,9892
ID: 1074	**898**	5,62E-01	0,958	3,30E-02	0,690	3,17E-02	1,388					1,073	1,555	1,120	38,8393
ID: 1076	**899**	0,69922885	0,975	0,13929	0,948	0,60004062	1,028					1,001	1,056	1,027	2,8238
ID: 1077	**900**	0,52409259	1,029	0,25514	1,159	0,28043307	0,888					1,064	0,918	1,034	-11,2186
ID: 1079	**901**	0,99506537	0,992	0,38931	1,087	0,46314834	0,913					0,901	0,829	0,908	-8,7004
ID: 1080	**902**	0,18943813	1,168	0,51431	1,099	0,75213262	1,063					1,120	1,019	0,959	6,2565
ID: 1081	**903**	0,74458506	1,005	0,39294	1,032	0,40108926	0,974					1,126	1,091	1,120	-2,5893
ID: 1082	**904**	0,12224867	1,134	0,06069	1,211	0,36703195	0,937					1,040	0,859	0,917	-6,3250
ID: 1085	**905**	0,079257	1,131	0,08857	1,131	0,99791446	1,000					1,279	1,131	1,131	0,0000
ID: 1086	**906**	0,93108376	1,000	0,06556	0,909	0,20565508	1,100					1,039	1,143	1,039	10,0096
ID: 1087	**907**	0,50280636	1,058	0,27006	1,112	0,37810126	0,951					1,099	0,988	1,039	-4,9086
ID: 1089	**908**	0,46891281	1,026	0,00324	1,171	0,00419038	0,877					1,166	0,996	1,136	-12,3239
ID: 1090	**909**	0,05340354	1,199	0,86297	1,005	0,20146098	1,192					0,947	0,942	0,79	19,2405

ID: 1091	**910**	0,84221	0,979	0,51025	0,957	0,75828346	1,023					0,939	0,981	0,959	2,2941
ID: 1092	**911**	0,68769139	0,986	0,46768	1,053	0,34609491	0,936					1,163	1,104	1,180	-6,4407
ID: 1093	**912**	0,55046028	1,023	0,94655	1,002	0,63270042	1,022					1,090	1,088	1,065	2,1596
ID: 1094	**913**	0,26022344	1,186	0,42102	1,111	0,72358418	1,067					0,758	0,682	0,639	6,7293
ID: 1095	**914**	0,98681763	0,989	0,11981	1,438	0,13086426	0,688					0,726	0,505	0,734	-31,1989
ID: 1096	**915**	0,8940858	0,997	0,96334	0,966	0,89152353	1,032					1,232	1,275	1,236	3,1553
ID: 1097	**916**	0,62580195	1,028	0,0368	1,146	0,22937198	0,897					1,137	0,992	1,106	-10,3074
ID: 1098	**917**	0,34253426	1,096	0,67422	1,051	0,36265386	1,044					1,080	1,028	0,985	4,3655
ID: 1100	**918**	0,82015022	0,988	0,8549	0,988	0,99236716	1,000					1,086	1,099	1,099	0,0000
ID: 1101	**919**	0,65220074	1,111	0,43033	1,224	0,57252801	0,907					1,151	0,94	1,036	-9,2664
ID: 1102	**920**	0,21066494	1,162	0,19119	1,167	0,99905863	0,996					1,134	0,972	0,976	-0,4098
ID: 1103	**921**	0,49875124	1,110	0,05895	1,609	0,11767696	0,690					0,975	0,606	0,878	-30,9795
ID: 1104	**922**	0,54204431	1,134	0,05498	1,656	0,23952538	0,685					0,962	0,581	0,848	-31,4858
ID: 1105	**923**	0,5532177	1,070	0,92877	0,969	0,56368054	1,104					0,978	1,009	0,914	10,3939
ID: 1106	**924**	0,63894966	1,095	0,22715	0,840	0,08188238	1,303					1,041	1,239	0,951	30,2839
ID: 1108	**925**	0,81557055	1,010	0,43375	1,074	0,79153177	0,940					0,742	0,691	0,735	-5,9864
ID: 1109	**926**	0,97201047	1,003	0,5384	0,962	0,40650708	1,043					1,036	1,077	1,033	4,2594
ID: 1110	**927**	0,99567558	0,984	0,14898	1,087	0,48673052	0,906					1,177	1,083	1,196	-9,4482
ID: 1111	**928**	0,26955666	1,119	0,08304	1,266	0,38643786	0,884					0,865	0,683	0,773	-11,6429
ID: 1112	**929**	0,63476132	1,046	0,30597	1,116	0,69536863	0,937					0,931	0,834	0,89	-6,2921
ID: 1114	**930**	0,99969249	0,958	0,74593	0,900	0,78469315	1,064					0,839	0,932	0,876	6,3927
ID: 1115	**931**	0,95295391	0,913	0,7978	1,005	0,8114308	0,909					0,851	0,847	0,932	-9,1202
ID: 1116	**932**	0,4775379	1,096	0,08915	1,251	0,46753538	0,876					0,807	0,645	0,736	-12,3641
ID: 1117	**933**	0,19483173	1,373	0,04381	1,614	0,52040297	0,851					1,262	0,782	0,919	-14,9075
ID: 1118	**934**	0,18039636	1,205	0,01018	1,584	0,15853426	0,761					1,093	0,69	0,907	-23,9250
ID: 1119	**935**	0,36070163	1,047	0,68298	1,027	0,79319227	1,019					1,085	1,056	1,036	1,9305
ID: 1120	**936**	0,65164049	1,016	0,43923	1,030	0,70240653	0,986					1,090	1,058	1,073	-1,3979
ID: 1121	**937**	0,48182858	0,852	0,41514	0,850	0,92744959	1,002					0,867	1,020	1,018	0,1965
ID: 1122	**938**	0,5234098	1,058	0,0655	1,156	0,28198648	0,915					0,954	0,825	0,902	-8,5366
ID: 1123	**939**	0,75588576	1,025	0,46155	0,932	0,29252387	1,101					1,050	1,127	1,024	10,0586
ID: 1124	**940**	0,61053305	1,069	0,10732	1,248	0,12624972	0,857					1,124	0,901	1,051	-14,2721
ID: 1125	**941**	0,54793852	0,961	0,59561	0,963	0,97351145	0,998					1,008	1,047	1,049	-0,1907
ID: 1126	**942**	0,7872159	1,026	0,33797	1,105	0,32865253	0,929					1,055	0,955	1,028	-7,1012
ID: 1127	**943**	0,05317008	0,877	0,5641	0,947	0,22628769	0,926					0,963	1,017	1,098	-7,3770
ID: 1128	**944**	0,39942293	0,773	0,04334	0,683	0,4068059	1,132					0,756	1,107	0,978	13,1902
ID: 1129	**945**	0,08400883	1,223	0,20399	1,303	0,54272171	0,939					1,036	0,795	0,847	-6,1393
ID: 1130	**946**	0,1704668	1,165	0,39887	1,137	0,96468618	1,024					1,019	0,896	0,875	2,4000
ID: 1131	**947**	0,01292752	1,854	0,05952	1,959	0,6602327	0,947					1,146	0,585	0,618	-5,3398
ID: 1133	**948**	0,2925879	1,036	0,61834	1,016	0,65621905	1,019					1,128	1,110	1,089	1,9284
ID: 1134	**949**	0,0442848	1,213	0,82079	1,015	0,08366769	1,195					1,084	1,068	0,894	19,4631

ID: 1135	**950**	0,43659544	1,059	0,0581	1,292	0,1258561	0,820					0,875	0,677	0,826	-18,0387
ID: 1136	**951**	0,72939791	1,026	0,94239	1,007	0,77779471	1,019					1,038	1,031	1,012	1,8775
ID: 1137	**952**	0,93491616	0,974	0,05433	1,254	0,11214221	0,777					0,838	0,668	0,86	-22,3256
ID: 1138	**953**	0,79960141	0,974	0,93964	1,001	0,78434763	0,973					0,949	0,948	0,974	-2,6694
ID: 1139	**954**	0,95748337	1,007	0,82468	0,983	0,69461144	1,025					0,986	1,003	0,979	2,4515
ID: 1141	**955**	0,51963549	1,095	0,35733	1,143	0,90405391	0,958					0,937	0,82	0,856	-4,2056
ID: 1142	**956**	0,71158443	1,022	0,04391	1,130	0,2347177	0,905					0,828	0,733	0,81	-9,5062
ID: 1143	**957**	0,82642636	0,987	0,68828	0,988	0,97453837	0,999					1,023	1,035	1,036	-0,0965
ID: 1144	**958**	0,86572788	1,028	0,17587	1,347	0,28362214	0,763					0,838	0,622	0,815	-23,6810
ID: 1145	**959**	0,66475304	1,051	0,78953	1,029	0,93173116	1,021					0,847	0,823	0,806	2,1092
ID: 1146	**960**	0,14848133	0,862	0,24931	0,852	0,9681695	1,012					1,084	1,272	1,257	1,1933
ID: 1147	**961**	0,32722256	0,855	0,04395	0,778	0,42510665	1,100					0,78	1,003	0,912	9,9781
ID: 1148	**962**	0,21905008	0,929	0,13595	0,907	0,6193003	1,025					0,908	1,001	0,977	2,4565
ID: 1149	**963**	0,92900652	0,981	0,5541	0,952	0,6965946	1,030					0,916	0,962	0,934	2,9979
ID: 1150	**964**	0,96862891	0,977	0,38316	1,215	0,47112678	0,804					0,728	0,599	0,745	-19,5973
ID: 1151	**965**	0,74837434	0,979	0,68175	0,959	0,83645278	1,021					1,177	1,227	1,202	2,0799
ID: 1152	**966**	0,58615623	0,964	0,99424	0,987	0,73690809	0,977					0,889	0,901	0,922	-2,2777
ID: 1153	**967**	0,48076542	1,061	0,34038	1,075	0,88496128	0,987					1,204	1,120	1,135	-1,3216
ID: 1154	**968**	0,75864465	1,014	0,91653	0,990	0,70375434	1,024					1,001	1,011	0,987	2,4316
ID: 1155	**969**	0,65848619	1,033	0,96584	0,997	0,72343556	1,036					1,067	1,070	1,033	3,5818
ID: 1156	**970**	0,77003741	1,015	0,42703	1,044	0,57930562	0,972					0,923	0,884	0,909	-2,7503
ID: 1158	**971**	0,51426528	0,944	0,32164	0,875	0,56667141	1,079					1,004	1,148	1,064	7,8947
ID: 1160	**972**	0,91832589	0,992	0,6227	0,945	0,66475976	1,050					0,835	0,884	0,842	4,9881
ID: 1162	**973**	0,76824974	1,012	0,5301	0,876	0,47978662	1,155					1,098	1,253	1,085	15,4839
ID: 1163	**974**	0,01849072	0,926	0,98188	0,992	0,27278633	0,934					1,089	1,098	1,176	-6,6327
ID: 1164	**975**	0,42003258	1,018	0,14987	1,068	0,27587365	0,954					1,170	1,096	1,149	-4,6127
ID: 1165	**976**	0,4327665	1,058	0,04142	1,135	0,37703162	0,932					0,95	0,837	0,898	-6,7929
ID: 1166	**977**	0,66684508	0,976	0,57681	0,962	0,80724937	1,015					0,929	0,966	0,952	1,4706
ID: 1167	**978**	0,34351449	1,151	0,11577	1,291	0,67608056	0,891					0,932	0,722	0,81	-10,8642
ID: 1168	**979**	0,43153633	1,085	0,25988	1,126	0,74948123	0,963					0,949	0,843	0,875	-3,6571
ID: 1170	**980**	0,49082272	1,060	0,87534	1,019	0,54691466	1,040					0,973	0,955	0,918	4,0305
ID: 1171	**981**	0,08959654	0,882	0,04945	0,883	0,96741062	0,999					0,93	1,053	1,054	-0,0949
ID: 1172	**982**	0,42474668	0,924	0,41022	0,896	0,82333933	1,032					1,322	1,476	1,430	3,2168
ID: 1173	**983**	0,28292923	0,851	0,61112	0,933	0,5117328	0,913					0,983	1,054	1,155	-8,7446
ID: 1175	**984**	0,70870912	0,955	0,32905	1,155	0,9968445	0,826					0,632	0,547	0,662	-17,3716
ID: 1176	**985**	0,37974179	0,901	0,23234	1,227	0,04795095	0,734					0,963	0,785	1,069	-26,5669
ID: 1177	**986**	0,49878881	0,922	0,49949	0,889	0,85481795	1,037					0,918	1,033	0,996	3,7149
ID: 1178	**987**	0,37336503	1,110	0,76909	0,965	0,31751477	1,151					0,955	0,99	0,86	15,1163
ID: 1179	**988**	0,38018414	1,096	0,01839	1,276	0,2278742	0,859					0,99	0,776	0,903	-14,0642
ID: 1180	**989**	0,11956994	1,123	0,03311	1,218	0,28609986	0,922					0,96	0,788	0,855	-7,8363

ID: 1181	**990**	0,6427691	1,027	0,92206	1,008	0,62782631	1,019					1,055	1,047	1,027	1,9474
ID: 1182	**991**	0,99687379	1,000	0,43585	1,035	0,43395386	0,966					1,148	1,109	1,148	-3,3972
ID: 1183	**992**	0,14676576	1,078	0,63429	1,029	0,25611684	1,047					0,944	0,917	0,876	4,6804
ID: 1184	**993**	0,91466454	1,006	0,97718	1,000	0,83739399	1,006					1,016	1,016	1,010	0,5941
ID: 1185	**994**	0,59943542	1,153	0,30354	1,301	0,21135281	0,886					0,967	0,743	0,839	-11,4422
ID: 1186	**995**	0,56689321	1,074	0,22502	1,169	0,24773931	0,918					0,815	0,697	0,759	-8,1686
ID: 1187	**996**	0,93934745	0,991	0,90393	1,008	0,85006148	0,983					0,877	0,87	0,885	-1,6949
ID: 1188	**997**	0,61212252	1,035	0,01195	1,384	0,01313993	0,748					0,905	0,654	0,874	-25,1716
ID: 1190	**998**	0,33045439	0,966	0,61497	0,974	0,81760799	0,992					1,085	1,114	1,123	-0,8014
ID: 1192	**999**	0,63986111	1,047	0,14491	0,882	0,16669206	1,187					0,825	0,935	0,788	18,6548
ID: 1193	**1000**	0,6157427	1,072	0,44626	0,917	0,17671348	1,169					0,956	1,043	0,892	16,9283
ID: 1194	**1001**	0,83193252	1,008	0,12584	0,906	0,12066244	1,113					1,029	1,136	1,021	11,2635
ID: 1195	**1002**	0,47137818	1,063	0,64038	0,945	0,28743564	1,125					1,142	1,208	1,074	12,4767
ID: 1196	**1003**	0,99077493	1,001	0,75653	0,984	0,69434264	1,017					1,078	1,095	1,077	1,6713
ID: 1197	**1004**	0,41485744	1,128	0,08138	1,369	0,31920258	0,824					1,098	0,802	0,973	-17,5745
ID: 1198	**1005**	0,48327313	0,961	0,80206	1,015	0,38684186	0,947					1,010	0,995	1,051	-5,3283
ID: 1200	**1006**	0,95063095	0,996	0,17278	1,214	0,26272418	0,820					0,708	0,583	0,711	-18,0028
ID: 1202	**1007**	0,3179731	1,090	0,15978	1,133	0,55221801	0,962					1,184	1,045	1,086	-3,7753
ID: 1205	**1008**	0,61997148	1,054	0,01762	1,432	0,251243	0,736					1,060	0,74	1,006	-26,4414
ID: 1207	**1009**	0,10984803	0,875	0,73416	0,937	0,49375252	0,934					0,715	0,763	0,817	-6,6095
ID: 1208	**1010**	0,26396138	0,882	0,1157	0,764	0,12457603	1,155					0,892	1,168	1,011	15,5292
ID: 1209	**1011**	0,64964427	1,026	0,91617	0,996	0,48527556	1,030					1,040	1,044	1,014	2,9586
ID: 1210	**1012**	0,08360867	1,104	0,07016	1,123	0,49499936	0,984					0,943	0,84	0,854	-1,6393
ID: 1211	**1013**	0,54178427	0,844	0,84232	0,917	0,74664895	0,920					0,626	0,683	0,742	-7,9515
ID: 1212	**1014**	0,74754273	1,050	0,98901	1,010	0,60896417	1,040					0,897	0,888	0,854	3,9813
ID: 1213	**1015**	0,43984374	1,050	0,42144	1,053	0,96372861	0,997					1,135	1,078	1,081	-0,2775
ID: 1214	**1016**	0,0307741	1,097	0,50165	0,947	0,04322935	1,158					1,032	1,090	0,941	15,8342
ID: 1215	**1017**	0,57270686	0,952	0,36319	0,953	0,94549309	0,999					1,034	1,085	1,086	-0,0921
ID: 1218	**1018**	0,19596189	1,230	0,6001	1,105	0,41434197	1,113					1,204	1,090	0,979	11,3381
ID: 1219	**1019**	0,94641781	0,984	0,21178	1,288	0,23275302	0,764					1,030	0,8	1,047	-23,5912
ID: 1220	**1020**	0,16765898	1,149	0,00202	1,468	0,01004528	0,783	Agrotis_qualite_rep_c16812| ORF3 length 323 aa, 972 bp, from 102..1073 of	15	AGW99945.1	geranylgeranyl diphosphate synthase [Choristoneura fumiferana]	1,026	0,699	0,893	-21,7245
ID: 1221	**1021**	0,52297629	0,931	0,89699	0,966	0,67135067	0,964					0,717	0,742	0,77	-3,6364
ID: 1222	**1022**	0,86776127	0,985	0,9737	1,002	0,84424673	0,982					0,895	0,893	0,909	-1,7602
ID: 1223	**1023**	0,60989291	1,035	0,2283	1,095	0,36405202	0,946					0,972	0,888	0,939	-5,4313
ID: 1224	**1024**	0,4384096	1,106	0,29617	1,149	0,81071728	0,963					1,003	0,873	0,907	-3,7486
ID: 1226	**1025**	0,15977552	1,045	0,25453	1,033	0,65689234	1,012					1,129	1,093	1,080	1,2037
ID: 1227	**1026**	0,56539648	0,983	0,01255	0,934	0,10203489	1,052					1,039	1,112	1,057	5,2034
ID: 1228	**1027**	0,35878659	1,179	0,00983	1,721	0,06390006	0,685					1,217	0,707	1,032	-31,4922

ID: 1229	**1028**	0,754764	0,986	0,11707	0,924	0,09848069	1,066					1,097	1,187	1,113	6,6487
ID: 1230	**1029**	0,01388678	1,308	0,41042	1,087	0,04178418	1,203					1,101	1,013	0,842	20,3088
ID: 1231	**1030**	0,2317652	1,401	0,06454	1,847	0,11745776	0,758					1,136	0,615	0,811	-24,1677
ID: 1232	**1031**	0,10792101	1,198	0,82245	1,016	0,33443346	1,179					0,902	0,888	0,753	17,9283
ID: 1233	**1032**	0,86877577	0,976	0,76108	0,953	0,87135058	1,023					0,877	0,92	0,899	2,3359
ID: 1234	**1033**	0,54022218	1,138	0,73615	0,969	0,24491459	1,175					0,831	0,858	0,73	17,5342
ID: 1235	**1034**	0,91048199	0,942	0,67255	0,950	0,86936835	0,991					0,994	1,046	1,055	-0,8531
ID: 1236	**1035**	0,01798247	0,751	0,14828	1,205	0,00385357	0,623					0,869	0,721	1,157	-37,6837
ID: 1237	**1036**	0,07560037	1,088	0,00994	1,123	0,53634478	0,969					1,186	1,056	1,090	-3,1193
ID: 1238	**1037**	0,74322345	1,010	0,32403	1,036	0,43558508	0,975					1,128	1,089	1,117	-2,5067
ID: 1239	**1038**	0,83361974	1,019	0,03894	1,238	0,07755169	0,823					1,144	0,924	1,123	-17,7204
ID: 1241	**1039**	0,19248249	1,152	0,90613	0,961	0,30542628	1,199					1,159	1,206	1,006	19,8807
ID: 1242	**1040**	0,32049633	0,959	0,02722	0,882	0,03389567	1,088					1,029	1,167	1,073	8,7605
ID: 1244	**1041**	0,00209087	2,133	0,04527	1,554	0,02776456	1,373					1,557	1,002	0,73	37,2603
ID: 1245	**1042**	0,37911506	1,166	0,97333	1,032	0,16294992	1,129					1,118	1,083	0,959	12,9301
ID: 1246	**1043**	0,91270181	1,006	0,24457	0,903	0,19607399	1,115					0,935	1,036	0,929	11,5178
ID: 1247	**1044**	0,17372865	1,336	0,06672	1,450	0,73014598	0,922					1,315	0,907	0,984	-7,8252
ID: 1248	**1045**	0,52540105	1,043	0,39738	0,958	0,07319445	1,088					0,945	0,986	0,906	8,8300
ID: 1249	**1046**	0,97994064	0,998	0,45396	0,858	0,41666607	1,163					0,98	1,142	0,982	16,2933
ID: 1250	**1047**	0,4514214	0,875	0,57434	1,157	0,20841101	0,756					0,75	0,648	0,857	-24,3874
ID: 1251	**1048**	0,17624084	0,930	0,49747	0,974	0,34496993	0,955					1,065	1,093	1,145	-4,5415
ID: 1252	**1049**	0,74209381	0,986	0,60136	0,976	0,81468362	1,010					1,078	1,104	1,093	1,0064
ID: 1253	**1050**	0,65473248	1,022	0,06689	1,082	0,22454212	0,944					1,132	1,046	1,108	-5,5957
ID: 1254	**1051**	0,39290668	1,065	0,24498	0,901	0,07234495	1,182					1,063	1,180	0,998	18,2365
ID: 1255	**1052**	0,78763587	0,938	0,97483	0,981	0,79319933	0,956					0,895	0,912	0,954	-4,4025
ID: 1257	**1053**	0,17831972	0,876	0,26922	0,824	0,70431322	1,064					0,896	1,088	1,023	6,3539
ID: 1259	**1054**	0,48951941	1,057	0,17492	1,096	0,73374326	0,965					1,015	0,926	0,96	-3,5417
ID: 1260	**1055**	0,24856498	1,063	0,20341	1,060	0,9148729	1,003					1,031	0,973	0,97	0,3093
ID: 1261	**1056**	0,0266331	1,133	0,95787	0,987	0,15577331	1,148					0,964	0,977	0,851	14,8061
ID: 1262	**1057**	0,04433412	1,104	0,15467	1,065	0,14977818	1,036					1,060	0,995	0,96	3,6458
ID: 1264	**1058**	0,73014189	1,064	0,54489	1,117	0,66713983	0,952					1,000	0,895	0,94	-4,7872
ID: 1265	**1059**	0,67278281	0,981	0,6999	0,985	0,93982239	0,996					1,138	1,155	1,160	-0,4310
ID: 1267	**1060**	0,28134697	0,840	0,03257	0,799	0,58774379	1,051					0,943	1,180	1,123	5,0757
ID: 1268	**1061**	0,68985311	1,020	0,91809	1,003	0,88187742	1,017					1,024	1,021	1,004	1,6932
ID: 1270	**1062**	0,97249601	1,004	0,93166	1,006	0,94846278	0,998					1,012	1,006	1,008	-0,1984
ID: 1271	**1063**	0,12671945	1,078	0,42039	1,040	0,19154543	1,036					1,187	1,141	1,101	3,6331
ID: 1272	**1064**	0,33887086	0,914	0,54764	1,044	0,24249483	0,876					0,951	0,911	1,040	-12,4038
ID: 1273	**1065**	0,7778629	1,015	0,79397	1,017	0,85503772	0,998					1,088	1,070	1,072	-0,1866
ID: 1274	**1066**	0,83617446	1,009	0,17351	1,096	0,34891088	0,921					1,020	0,931	1,011	-7,9130
ID: 1275	**1067**	0,90826868	1,003	0,81454	1,010	0,89307813	0,993					0,997	0,987	0,994	-0,7042

ID: 1276	**1068**	0,40699784	1,232	0,26936	1,393	0,93965235	0,885					1,025	0,736	0,832	-11,5385
ID: 1277	**1069**	0,55342067	0,893	0,88058	0,990	0,60275587	0,902					0,917	0,926	1,027	-9,8345
ID: 1278	**1070**	0,07411841	0,819	0,09579	0,867	0,56637223	0,944					0,851	0,981	1,039	-5,5823
ID: 1280	**1071**	0,87493361	1,005	0,30098	0,959	0,18602568	1,047					1,042	1,086	1,037	4,7252
ID: 1281	**1072**	0,33956042	1,124	0,03221	1,318	0,26382703	0,853					0,68	0,516	0,605	-14,7107
ID: 1282	**1073**	0,38041624	1,086	0,37567	1,108	0,75378107	0,980					0,998	0,901	0,919	-1,9587
ID: 1283	**1074**	0,89184597	1,005	0,81302	0,971	0,75604214	1,035					1,069	1,101	1,064	3,4774
ID: 1284	**1075**	0,93137933	0,949	0,66426	1,047	0,67016558	0,907					1,100	1,051	1,159	-9,3184
ID: 1285	**1076**	0,94138127	1,003	0,62265	0,941	0,55321898	1,066					0,969	1,030	0,966	6,6253
ID: 1286	**1077**	0,49718278	0,976	0,09509	0,898	0,11581885	1,086					1,009	1,123	1,034	8,6074
ID: 1287	**1078**	0,34817502	1,061	0,20207	0,910	0,01294592	1,166					1,008	1,108	0,95	16,6316
ID: 1288	**1079**	0,87910823	0,993	0,66147	0,979	0,75070292	1,015					1,003	1,025	1,010	1,4851
ID: 1289	**1080**	0,36754506	1,068	0,18685	1,144	0,33322056	0,934					1,267	1,108	1,186	-6,5767
ID: 1290	**1081**	0,72283193	1,053	0,72998	0,959	0,38530954	1,099					1,207	1,259	1,146	9,8604
ID: 1291	**1082**	0,16882168	1,139	0,10786	1,247	0,48407168	0,914					0,925	0,742	0,812	-8,6207
ID: 1292	**1083**	0,30618181	1,088	0,368	1,071	0,7909616	1,016					0,953	0,89	0,876	1,5982
ID: 1293	**1084**	0,64016766	1,017	0,86218	1,007	0,6278202	1,010					1,118	1,110	1,099	1,0009
ID: 1296	**1085**	0,17609663	1,062	0,0699	1,124	0,25368128	0,945					1,245	1,108	1,172	-5,4608
ID: 1297	**1086**	0,75778624	0,989	0,89733	1,006	0,70917054	0,984					1,093	1,087	1,105	-1,6290
ID: 1298	**1087**	0,15223194	1,192	0,33845	1,118	0,64418441	1,067					1,016	0,909	0,852	6,6901
ID: 1299	**1088**	0,92657908	1,007	0,64239	0,974	0,54284992	1,034					1,133	1,163	1,125	3,3778
ID: 1300	**1089**	0,95436524	1,023	0,44423	0,905	0,21353272	1,130					1,171	1,294	1,145	13,0131
ID: 1301	**1090**	0,95831574	0,993	0,52068	0,911	0,54819466	1,091					0,888	0,975	0,894	9,0604
ID: 1302	**1091**	0,81522453	0,970	0,8627	0,982	0,94891223	0,988					0,873	0,889	0,9	-1,2222
ID: 1303	**1092**	0,15893079	1,040	0,02458	1,108	0,01963792	0,939					1,060	0,957	1,019	-6,0844
ID: 1304	**1093**	0,61165198	1,060	0,68919	0,961	0,40740727	1,102					1,068	1,111	1,008	10,2183
ID: 1305	**1094**	0,44269474	1,083	0,72694	1,044	0,64595311	1,038					1,029	0,986	0,95	3,7895
ID: 1306	**1095**	0,42721138	1,136	0,80235	0,981	0,18325661	1,158					1,173	1,196	1,033	15,7793
ID: 1307	**1096**	0,08952064	1,271	0,47981	1,077	0,19075811	1,180					1,234	1,146	0,971	18,0227
ID: 1308	**1097**	0,57335546	0,872	0,2576	0,761	0,56910723	1,145					0,884	1,161	1,014	14,4970
ID: 1309	**1098**	0,87759309	0,983	0,30677	1,189	0,20997678	0,827					0,995	0,837	1,012	-17,2925
ID: 1312	**1099**	0,18317339	1,214	0,36054	1,173	0,9322747	1,035					1,184	1,009	0,975	3,4872
ID: 1313	**1100**	0,80630832	0,987	0,80988	0,980	0,95319688	1,007					1,174	1,198	1,190	0,6723
ID: 1317	**1101**	0,37178778	1,107	0,06642	1,402	0,09462008	0,790					1,082	0,772	0,977	-20,9826
ID: 1318	**1102**	0,30838923	1,335	0,31138	1,365	0,92304157	0,978					1,100	0,806	0,824	-2,1845
ID: 1319	**1103**	0,69823397	1,034	0,78525	0,977	0,50811773	1,058					1,035	1,059	1,001	5,7942
ID: 1320	**1104**	0,65910454	1,019	0,51582	0,975	0,2725701	1,045					1,039	1,066	1,020	4,5098
ID: 1321	**1105**	0,20726252	1,059	0,25561	1,046	0,81765763	1,012					1,135	1,085	1,072	1,2127
ID: 1322	**1106**	0,24033954	1,235	0,4785	1,170	0,83950109	1,056					1,200	1,026	0,972	5,5556
ID: 1323	**1107**	0,69679392	1,013	0,62117	0,978	0,32652937	1,035					1,089	1,113	1,075	3,5349

ID: 1324	**1108**	0,39974109	0,928	0,71374	0,953	0,7531782	0,973					1,064	1,116	1,147	-2,7027
ID: 1325	**1109**	0,4291832	0,974	0,46679	0,946	0,68313122	1,030					1,146	1,212	1,177	2,9737
ID: 1326	**1110**	0,88254763	1,009	0,87633	1,009	0,982089	1,000					0,902	0,894	0,894	0,0000
ID: 1327	**1111**	0,71262332	1,036	0,97277	0,994	0,69303542	1,041					0,901	0,906	0,87	4,1379
ID: 1328	**1112**	0,22940283	0,939	0,17344	0,886	0,45736725	1,061					0,992	1,120	1,056	6,0606
ID: 1329	**1113**	0,39842153	1,168	0,17936	1,388	0,60724608	0,841					0,515	0,371	0,441	-15,8730
ID: 1330	**1114**	0,61887024	1,019	0,8314	0,993	0,48061057	1,027					0,998	1,005	0,979	2,6558
ID: 1331	**1115**	0,93516736	1,003	0,12186	1,190	0,20304503	0,843					1,008	0,847	1,005	-15,7214
ID: 1332	**1116**	0,52709263	1,095	0,93338	0,972	0,53815546	1,126					1,024	1,053	0,935	12,6203
ID: 1333	**1117**	0,1656374	1,223	0,38423	1,143	0,2768937	1,070					1,531	1,340	1,252	7,0288
ID: 1334	**1118**	0,54831159	0,897	0,59455	1,094	0,3319443	0,820					0,804	0,735	0,896	-17,9688
ID: 1335	**1119**	0,08284999	1,231	0,78834	1,040	0,02516798	1,184					0,942	0,906	0,765	18,4314
ID: 1336	**1120**	0,54438799	1,042	0,58633	1,046	0,91853046	0,996					0,952	0,91	0,914	-0,4376
ID: 1337	**1121**	0,09970104	1,237	0,40897	1,116	0,34061494	1,109					1,023	0,917	0,827	10,8827
ID: 1338	**1122**	0,13422652	1,105	0,56502	1,041	0,44997362	1,062					1,075	1,033	0,973	6,1665
ID: 1339	**1123**	0,46458266	0,817	0,5602	0,904	0,78411524	0,904					0,759	0,84	0,929	-9,5802
ID: 1341	**1124**	0,110832	1,437	0,31865	1,296	0,27540133	1,109					1,078	0,832	0,75	10,9333
ID: 1342	**1125**	0,51528417	1,129	0,1427	1,348	0,37350038	0,837					0,979	0,726	0,867	-16,2630
ID: 1343	**1126**	0,11029055	1,245	0,35015	1,150	0,64206774	1,082					1,241	1,079	0,997	8,2247
ID: 1344	**1127**	0,31654645	1,100	0,35613	1,155	0,64195959	0,952					0,892	0,772	0,811	-4,8089
ID: 1345	**1128**	0,00585721	1,098	0,22006	0,951	0,00211783	1,154					1,069	1,124	0,974	15,4004
ID: 1347	**1129**	0,08851375	1,180	0,0071	1,293	0,27547783	0,913					1,153	0,892	0,977	-8,7001
ID: 1349	**1130**	0,9717186	0,975	0,10774	0,792	0,18099347	1,232					0,82	1,036	0,841	23,1867
ID: 1351	**1131**	0,22723628	1,083	0,83498	0,991	0,06634176	1,093					1,152	1,163	1,064	9,3045
ID: 1352	**1132**	0,95519546	1,005	0,33534	0,923	0,21822001	1,089					1,098	1,190	1,093	8,8747
ID: 1353	**1133**	0,16118307	1,102	0,19754	1,106	0,92005469	0,996					1,188	1,074	1,078	-0,3711
ID: 1354	**1134**	0,62926286	1,156	0,06168	2,237	0,23333619	0,517					0,311	0,139	0,269	-48,3271
ID: 1356	**1135**	0,06654661	1,439	0,22464	1,306	0,45154952	1,102					1,072	0,821	0,745	10,2013
ID: 1357	**1136**	0,28069568	1,181	0,17321	1,253	0,42046325	0,943					1,135	0,906	0,961	-5,7232
ID: 1358	**1137**	0,93170263	1,001	0,48424	1,109	0,24951239	0,902					0,842	0,759	0,841	-9,7503
ID: 1362	**1138**	0,93770524	1,005	0,99475	1,002	0,93500549	1,003					1,097	1,095	1,092	0,2747
ID: 1363	**1139**	0,37827713	1,079	0,95017	0,984	0,45540255	1,096					0,989	1,005	0,917	9,5965
ID: 1364	**1140**	0,45858841	1,085	0,03916	1,271	0,25002265	0,854					0,737	0,58	0,679	-14,5803
ID: 1365	**1141**	0,99282898	0,995	0,26736	1,084	0,35627873	0,918					0,931	0,859	0,936	-8,2265
ID: 1367	**1142**	0,51536105	1,090	0,04187	1,385	0,34131309	0,787					0,838	0,605	0,769	-21,3264
ID: 1368	**1143**	0,98016011	0,993	0,36756	1,069	0,44951471	0,929					1,047	0,979	1,054	-7,1157
ID: 1369	**1144**	0,31896231	1,074	0,15138	1,100	0,76257957	0,977					1,145	1,041	1,066	-2,3452
ID: 1370	**1145**	0,75148567	1,043	0,83961	1,026	0,96449203	1,016					0,858	0,836	0,823	1,5796
ID: 1372	**1146**	0,55011103	0,953	0,82891	1,017	0,42023383	0,938					1,222	1,202	1,282	-6,2402
ID: 1373	**1147**	0,1705372	1,140	0,21567	1,152	0,8835763	0,989					1,127	0,978	0,989	-1,1122

ID: 1374	**1148**	0,88339551	1,016	0,51486	1,063	0,38553067	0,955					1,046	0,984	1,030	-4,4660
ID: 1375	**1149**	0,87358424	1,013	0,48066	0,901	0,37075998	1,124					1,051	1,167	1,038	12,4277
ID: 1376	**1150**	0,92802498	1,004	0,73905	1,018	0,72505077	0,986					1,088	1,069	1,084	-1,3838
ID: 1377	**1151**	0,23193856	0,842	0,33804	0,875	0,82294366	0,963					0,913	1,044	1,084	-3,6900
ID: 1378	**1152**	0,1093334	1,281	0,02136	1,304	0,94601442	0,982					1,300	0,997	1,015	-1,7734
ID: 1379	**1153**	0,35128368	1,056	0,10691	1,107	0,36781462	0,954					1,080	0,976	1,023	-4,5943
ID: 1382	**1154**	0,34424884	1,173	0,71747	1,075	0,65632205	1,091					1,069	0,994	0,911	9,1109
ID: 1383	**1155**	0,26421029	0,888	0,0764	0,820	0,42027457	1,083					1,018	1,242	1,147	8,2825
ID: 1384	**1156**	0,14373254	1,157	0,80974	1,028	0,33907401	1,126					0,967	0,941	0,836	12,5598
ID: 1385	**1157**	0,77020654	1,071	0,2092	1,342	0,1155045	0,798					1,288	0,96	1,203	-20,1995
ID: 1386	**1158**	0,74577287	0,983	0,97175	0,983	0,86288675	1,000					0,921	0,937	0,937	0,0000
ID: 1388	**1159**	0,93355953	0,997	0,91791	0,997	0,98452385	1,000					1,105	1,108	1,108	0,0000
ID: 1389	**1160**	0,03194949	1,221	0,22901	1,158	0,64639495	1,055					1,291	1,115	1,057	5,4872
ID: 1390	**1161**	0,17250459	1,135	0,16849	1,143	0,93560285	0,993					1,018	0,891	0,897	-0,6689
ID: 1391	**1162**	0,06821574	1,117	0,11851	1,124	0,8522094	0,993					1,006	0,895	0,901	-0,6659
ID: 1392	**1163**	0,76424634	1,008	0,27787	1,032	0,17340507	0,977					1,105	1,071	1,096	-2,2810
ID: 1394	**1164**	0,95375759	1,002	0,26737	1,089	0,23880346	0,920					1,230	1,129	1,227	-7,9870
ID: 1395	**1165**	0,50071333	1,049	0,37288	0,921	0,08562308	1,139					1,158	1,258	1,104	13,9493
ID: 1396	**1166**	0,89336387	1,015	0,60133	0,956	0,48269503	1,061					0,963	1,007	0,949	6,1117
ID: 1398	**1167**	0,39710501	1,046	0,1205	1,111	0,34584778	0,941					1,108	0,997	1,059	-5,8546
ID: 1399	**1168**	0,90864311	1,000	0,3893	1,116	0,15692404	0,896					0,974	0,873	0,974	-10,3696
ID: 1400	**1169**	0,02451976	0,668	0,10926	0,558	0,66742561	1,197					0,703	1,260	1,053	19,6581
ID: 1401	**1170**	0,93392609	1,012	0,46854	0,908	0,39192946	1,114					1,300	1,431	1,285	11,3619
ID: 1402	**1171**	0,00020081	0,447	0,01919	0,383	0,77444871	1,166					0,449	1,172	1,005	16,6169
ID: 1403	**1172**	0,03234217	1,564	0,14355	1,557	0,74017495	1,005					1,356	0,871	0,867	0,4614
ID: 1404	**1173**	0,54101796	1,029	0,34742	1,066	0,5638047	0,965					1,367	1,282	1,328	-3,4639
ID: 1405	**1174**	0,47948055	0,958	0,49621	0,934	0,80341109	1,026					1,084	1,160	1,131	2,5641
ID: 1407	**1175**	0,40068334	1,105	0,3537	1,128	0,8455932	0,979					0,967	0,857	0,875	-2,0571
ID: 1411	**1176**	0,39236822	1,085	0,00705	0,450	0,0608207	2,411					0,764	1,697	0,704	141,0511
ID: 1412	**1177**	0,31023076	1,152	0,088	1,310	0,36185572	0,880					1,437	1,097	1,247	-12,0289
ID: 1413	**1178**	0,33465488	1,131	0,58985	1,052	0,50171305	1,075					1,172	1,114	1,036	7,5290
ID: 1414	**1179**	0,7615807	1,031	0,19319	0,901	0,11251352	1,144					1,281	1,421	1,242	14,4122
ID: 1415	**1180**	0,94828248	1,057	0,55449	1,082	0,51509157	0,977					0,722	0,667	0,683	-2,3426
ID: 1416	**1181**	0,27590065	1,072	0,85769	1,011	0,59920772	1,060					1,076	1,064	1,004	5,9761
ID: 1417	**1182**	0,66665396	0,892	0,00286	0,433	0,07788769	2,058					0,692	1,597	0,776	105,7990
ID: 1419	**1183**	0,12887134	0,854	0,77759	0,962	0,26307098	0,887					1,017	1,057	1,191	-11,2510
ID: 1420	**1184**	0,48463562	1,041	0,66088	1,024	0,7404078	1,016					1,139	1,112	1,094	1,6453
ID: 1421	**1185**	0,64036208	1,082	0,44388	0,846	0,25396523	1,279					1,078	1,274	0,996	27,9116
ID: 1422	**1186**	0,47746364	0,724	0,78219	0,983	0,51792448	0,737					1,199	1,220	1,655	-26,2840
ID: 1423	**1187**	0,79932014	0,990	0,37278	0,954	0,42919997	1,038					1,126	1,180	1,137	3,7819

ID: 1424	**1188**	0,3177539	1,147	0,10118	1,330	0,46288919	0,862					1,165	0,876	1,016	-13,7795
ID: 1425	**1189**	0,25242193	0,686	0,04729	0,549	0,40859532	1,249					0,562	1,023	0,819	24,9084
ID: 1426	**1190**	0,21906278	1,256	0,14229	1,355	0,25618481	0,927					1,217	0,898	0,969	-7,3271
ID: 1427	**1191**	0,33642582	1,076	0,39157	1,082	0,87918765	0,994					1,120	1,035	1,041	-0,5764
ID: 1428	**1192**	0,30602835	0,964	0,324	0,963	0,97136699	1,001					1,059	1,100	1,099	0,0910
ID: 1430	**1193**	0,16451236	1,085	0,52663	1,041	0,24665469	1,043					1,146	1,101	1,056	4,2614
ID: 1432	**1194**	0,82569858	1,010	0,29201	1,054	0,35440707	0,959					1,267	1,202	1,254	-4,1467
ID: 1433	**1195**	0,23594077	1,356	0,05584	1,874	0,41307825	0,723					1,726	0,921	1,273	-27,6512
ID: 1434	**1196**	0,95422745	1,000	0,82352	0,979	0,81596085	1,021					0,992	1,013	0,992	2,1169
ID: 1435	**1197**	0,00541723	1,223	0,54981	1,061	0,24071533	1,153					1,049	0,989	0,858	15,2681
ID: 1436	**1198**	0,68531332	1,028	0,24198	1,091	0,33577119	0,942					1,223	1,121	1,190	-5,7983
ID: 1437	**1199**	0,43666866	1,192	0,26456	0,844	0,0477732	1,413					1,079	1,279	0,905	41,3260
ID: 1438	**1200**	0,91558987	0,996	0,69399	1,026	0,53314108	0,971					1,250	1,218	1,255	-2,9482
ID: 1439	**1201**	0,86141867	1,007	0,96726	1,002	0,90731226	1,005					1,118	1,116	1,110	0,5405
ID: 1440	**1202**	0,8011676	1,012	0,27791	1,082	0,21443447	0,936					1,152	1,065	1,138	-6,4148
ID: 1441	**1203**	0,52395521	1,031	0,11583	0,904	0,05363357	1,140					1,098	1,214	1,065	13,9906
ID: 1443	**1204**	0,70603057	0,962	0,35141	1,153	0,14429196	0,835					0,762	0,661	0,792	-16,5404
ID: 1444	**1205**	0,40452749	0,966	0,60396	0,986	0,63617057	0,980					1,053	1,068	1,090	-2,0183
ID: 1445	**1206**	0,31662383	1,151	0,39331	1,152	0,88567736	0,999					0,968	0,84	0,841	-0,1189
ID: 1446	**1207**	0,94656059	1,010	0,63618	1,144	0,68777653	0,883					0,914	0,799	0,905	-11,7127
ID: 1447	**1208**	0,37811376	0,876	0,52071	0,916	0,71576985	0,956					0,987	1,077	1,127	-4,4366
ID: 1448	**1209**	0,57140183	0,907	0,81264	0,971	0,70347143	0,934					0,66	0,68	0,728	-6,5934
ID: 1450	**1210**	0,5542623	0,936	0,80749	0,977	0,68591139	0,958					0,934	0,956	0,998	-4,2084
ID: 1451	**1211**	0,29994841	1,050	0,93831	0,996	0,19428099	1,054					1,079	1,083	1,028	5,3502
ID: 1452	**1212**	0,32782807	1,039	0,8147	1,011	0,28752873	1,028					1,148	1,136	1,105	2,8054
ID: 1455	**1213**	0,91622982	0,989	0,6927	0,941	0,72952531	1,051					0,801	0,851	0,81	5,0617
ID: 1456	**1214**	0,80968238	1,013	0,25637	1,063	0,19779838	0,953					1,174	1,104	1,159	-4,7455
ID: 1457	**1215**	0,06267832	0,759	0,46581	0,926	0,19082402	0,820					0,926	1,000	1,220	-18,0328
ID: 1458	**1216**	0,44571169	1,150	0,41486	0,877	0,1650369	1,312					0,781	0,891	0,679	31,2224
ID: 1459	**1217**	0,54892094	0,832	0,61951	1,053	0,4066477	0,790					0,874	0,83	1,050	-20,9524
ID: 1460	**1218**	0,34497629	1,204	0,8414	0,509	0,9717732	2,366					0,786	1,545	0,653	136,6003
ID: 1461	**1219**	0,89272787	1,047	0,3504	1,287	0,33038087	0,814					1,045	0,812	0,998	-18,6373
ID: 1462	**1220**	0,41963426	1,133	0,05856	1,357	0,38966875	0,835					0,886	0,653	0,782	-16,4962
ID: 1463	**1221**	0,62614656	1,030	0,79092	1,020	0,86216621	1,010					1,252	1,228	1,216	0,9868
ID: 1465	**1222**	0,4807722	1,213	0,1847	1,578	0,35037523	0,769					1,185	0,751	0,977	-23,1320
ID: 1468	**1223**	0,0362167	1,855	0,0385	2,066	0,73841834	0,898					1,564	0,757	0,843	-10,2017
ID: 1469	**1224**	0,76713911	0,823	0,29857	0,370	0,38148315	2,222					0,722	1,949	0,877	122,2349
ID: 1470	**1225**	0,93550341	0,934	0,41697	0,450	0,39702891	2,076					0,806	1,792	0,863	107,6477
ID: 1471	**1226**	0,01304136	1,136	0,03263	1,137	0,96561	0,999					1,150	1,011	1,012	-0,0988
ID: 1472	**1227**	0,89265182	0,985	0,28657	0,950	0,57423113	1,037					1,003	1,056	1,018	3,7328

ID: 1473	**1228**	0,21239159	1,064	0,00116	1,191	0,02642495	0,893					1,083	0,909	1,018	-10,7073
ID: 1474	**1229**	0,10957164	1,212	0,22394	1,177	0,46087284	1,029					1,116	0,948	0,921	2,9316
ID: 1476	**1230**	0,60235772	1,036	0,66171	1,036	0,96745119	1,000					1,139	1,099	1,099	0,0000
ID: 1477	**1231**	0,5020247	0,973	0,52635	0,963	0,83794866	1,010					1,104	1,146	1,135	0,9692
ID: 1479	**1232**	0,44746005	0,923	0,21323	0,891	0,55054306	1,037					0,953	1,070	1,032	3,6822
ID: 1480	**1233**	0,14145475	1,178	0,02498	1,342	0,32751024	0,878					1,000	0,745	0,849	-12,2497
ID: 1482	**1234**	0,41904872	1,042	0,25281	1,044	0,99701357	0,998					1,051	1,007	1,009	-0,1982
ID: 1483	**1235**	0,47221852	0,966	0,24243	0,943	0,61508488	1,024					1,131	1,199	1,171	2,3911
ID: 1484	**1236**	0,92521748	0,983	0,85956	1,036	0,77453151	0,949					1,243	1,200	1,265	-5,1383
ID: 1485	**1237**	0,52792298	1,093	0,86614	1,006	0,74291582	1,087					0,878	0,873	0,803	8,7173
ID: 1486	**1238**	0,58928423	1,044	0,47946	1,079	0,63441375	0,968					0,969	0,898	0,928	-3,2328
ID: 1487	**1239**	0,21478525	1,180	0,79494	1,017	0,4911704	1,161					0,713	0,701	0,604	16,0596
ID: 1488	**1240**	0,9939915	1,004	0,18194	1,105	0,06294086	0,908					1,096	0,992	1,092	-9,1575
ID: 1489	**1241**	0,28440146	0,887	0,48166	0,889	0,91199651	0,998					0,911	1,025	1,027	-0,1947
ID: 1490	**1242**	0,56775936	0,958	0,46605	0,929	0,72449795	1,031					1,007	1,084	1,051	3,1399
ID: 1491	**1243**	0,69449986	1,110	0,73018	0,918	0,39133754	1,210					1,026	1,118	0,924	20,9957
ID: 1492	**1244**	0,24948392	0,951	0,47261	0,973	0,61518053	0,977					1,224	1,258	1,287	-2,2533
ID: 1493	**1245**	0,34989319	1,087	0,22192	1,138	0,59094209	0,955					1,017	0,894	0,936	-4,4872
ID: 1494	**1246**	0,91426976	0,961	0,6348	1,098	0,5885656	0,875					0,949	0,864	0,987	-12,4620
ID: 1495	**1247**	0,470802	0,677	0,34945	1,170	0,24888901	0,578					0,86	0,735	1,271	-42,1715
ID: 1496	**1248**	0,46770095	1,087	0,72059	0,959	0,37020432	1,134					1,115	1,163	1,026	13,3528
ID: 1497	**1249**	0,79052489	0,978	0,83562	1,029	0,60484748	0,951					1,080	1,050	1,104	-4,8913
ID: 1498	**1250**	0,84905148	1,079	0,27366	0,467	0,16678475	2,309					0,768	1,644	0,712	130,8989
ID: 1499	**1251**	0,80312585	1,015	0,63562	1,033	0,74773699	0,983					0,999	0,967	0,984	-1,7276
ID: 1500	**1252**	0,62244523	1,062	0,47626	0,586	0,33148974	1,813					0,972	1,659	0,915	81,3115
ID: 1501	**1253**	0,6717356	0,968	0,6348	0,927	0,79741136	1,044					0,849	0,916	0,877	4,4470
ID: 1502	**1254**	0,54405082	1,077	0,29506	1,131	0,74181433	0,952					0,793	0,701	0,736	-4,7554
ID: 1503	**1255**	0,8856074	1,008	0,40053	1,045	0,56579298	0,965					1,195	1,144	1,185	-3,4599
ID: 1504	**1256**	0,73406473	0,986	0,88865	0,991	0,8919193	0,995					1,045	1,055	1,060	-0,4717
ID: 1505	**1257**	0,39740819	1,063	0,57785	0,969	0,09241582	1,098					1,056	1,090	0,993	9,7684
ID: 1506	**1258**	0,50935095	1,040	0,88966	1,004	0,77088621	1,035					1,231	1,226	1,184	3,5473
ID: 1509	**1259**	0,71652404	1,016	0,21451	1,100	0,86860088	0,924					1,015	0,923	0,999	-7,6076
ID: 1511	**1260**	0,42122979	1,145	0,96037	0,970	0,4584868	1,181					0,992	1,023	0,866	18,1293
ID: 1512	**1261**	0,42439699	1,087	0,22376	1,098	0,97110799	0,990					1,079	0,983	0,993	-1,0070
ID: 1515	**1262**	0,66517932	1,035	0,84758	0,969	0,61967405	1,068					0,985	1,017	0,952	6,8277
ID: 1516	**1263**	0,49782188	0,815	0,68318	1,039	0,39648455	0,784					0,979	0,942	1,201	-21,5654
ID: 1520	**1264**	0,07380359	1,241	0,02224	1,329	0,638849	0,934					1,419	1,068	1,143	-6,5617
ID: 1521	**1265**	0,84052399	1,006	0,45529	0,973	0,39806128	1,034					1,094	1,124	1,087	3,4039
ID: 1522	**1266**	0,38199132	0,678	0,99536	1,024	0,36028745	0,662					1,017	0,993	1,500	-33,8000
ID: 1523	**1267**	0,69102109	0,922	0,04243	1,284	0,06262625	0,718					0,895	0,697	0,971	-28,2183

ID: 1525	**1268**	0,89513074	0,989	0,22737	1,115	0,19106537	0,886					1,478	1,325	1,495	-11,3712
ID: 1527	**1269**	0,6376994	1,073	0,65589	1,077	0,98212925	0,997					0,939	0,872	0,875	-0,3429
ID: 1528	**1270**	0,13478563	0,778	0,26102	0,829	0,67164129	0,938					1,049	1,265	1,349	-6,2268
ID: 1529	**1271**	0,63538728	1,046	0,95618	1,003	0,32965555	1,043					1,164	1,161	1,113	4,3127
ID: 1530	**1272**	0,88600208	1,031	0,62825	0,949	0,43513053	1,086					1,146	1,208	1,112	8,6331
ID: 1531	**1273**	0,95236769	1,005	0,5975	0,978	0,32471759	1,028					1,104	1,129	1,098	2,8233
ID: 1532	**1274**	0,27987156	0,935	0,68966	0,832	0,88918663	1,123					1,115	1,340	1,193	12,3219
ID: 1533	**1275**	0,58336095	1,082	0,75564	1,027	0,95379708	1,054					0,946	0,921	0,874	5,3776
ID: 1535	**1276**	0,25697755	0,758	0,04777	0,717	0,63989915	1,057					0,849	1,184	1,120	5,7143
ID: 1536	**1277**	0,55007232	0,813	0,60542	0,937	0,75626493	0,868					0,819	0,874	1,007	-13,2075
ID: 1537	**1278**	0,99319459	1,020	0,81977	0,986	0,57788932	1,034					0,948	0,961	0,929	3,4446
ID: 1538	**1279**	0,08917835	1,114	0,34935	1,057	0,16034561	1,055					1,042	0,986	0,935	5,4545
ID: 1539	**1280**	0,37561599	0,810	0,47716	0,919	0,64074935	0,882					0,746	0,812	0,921	-11,8350
ID: 1541	**1281**	0,66179154	0,859	0,89842	0,939	0,78416678	0,915					0,929	0,989	1,081	-8,5106
ID: 1542	**1282**	0,07345153	1,118	0,21475	1,109	0,95122535	1,008					0,958	0,864	0,857	0,8168
ID: 1544	**1283**	0,55359935	0,941	0,60557	1,069	0,36803157	0,880					0,943	0,882	1,002	-11,9760
ID: 1545	**1284**	0,43898297	1,035	0,42284	0,969	0,06858823	1,067					1,014	1,046	0,98	6,7347
ID: 1546	**1285**	0,87134737	0,985	0,74938	0,933	0,79501741	1,056					1,223	1,311	1,241	5,6406
ID: 1548	**1286**	0,99045551	1,006	0,95807	0,993	0,94108349	1,013					0,831	0,837	0,826	1,3317
ID: 1552	**1287**	0,54721663	0,960	0,46151	0,940	0,72049661	1,021					1,094	1,164	1,140	2,1053
ID: 1561	**1288**	0,00658219	1,368	0,92147	0,999	9,0753E-05	1,369					1,126	1,127	0,823	36,9380
ID: 1562	**1289**	0,76089592	1,014	0,06228	1,088	0,35628701	0,933					0,993	0,913	0,979	-6,7416
ID: 1563	**1290**	0,47854161	1,246	0,18314	1,516	0,36157216	0,822					0,693	0,457	0,556	-17,8058
ID: 1564	**1291**	0,97264037	0,970	0,94884	1,008	0,94179347	0,963					0,649	0,644	0,669	-3,7369
ID: 1566	**1292**	0,74195924	1,078	0,40827	1,207	0,52554794	0,894					0,852	0,706	0,79	-10,6329
