# Supplementary material for: Sublethal Exposure Effects of the Neonicotinoid Clothianidin Strongly Modify the Brain Transcriptome and Proteome in the Male Moth Agrotis ipsilon
Source: Insects. 2021 Feb 11;12(2):152. doi: 10.3390/insects12020152 (PMC7916958; doi:10.3390/insects12020152)
Supplement: Supplementary file 1 [file insects-12-00152-s001.tgz › TableS2.pdf]

TRINITY CODE	sampleA	sampleB	logFC	logCPM	PValue	FDR
TRINITY_DN25122_c0_g1_i1	dmso	control	10,6640277	1,90508919	1,68E-48	1,28E-45
TRINITY_DN36952_c0_g1_i1	dmso	control	9,29488898	0,59679319	1,15E-23	1,88E-21
TRINITY_DN18250_c0_g1_i1	dmso	control	9,19400457	0,50333148	6,43E-23	9,77E-21
TRINITY_DN4321_c0_g1_i1	dmso	control	8,68806415	0,02634119	4,04E-20	4,78E-18
TRINITY_DN30866_c0_g1_i1	dmso	control	8,58128291	-0,0736318	6,34E-19	6,64E-17
TRINITY_DN6887_c0_g1_i1	dmso	control	5,90098246	2,82913333	1,84E-59	2,41E-56
TRINITY_DN42652_c0_g1_i1	dmso	control	5,80881397	-0,3991358	9,63E-14	5,63E-12
TRINITY_DN21355_c0_g1_i1	dmso	control	5,56224706	1,02874623	4,04E-26	7,72E-24
TRINITY_DN9441_c0_g1_i1	dmso	control	5,52397279	0,98493676	1,53E-19	1,71E-17
TRINITY_DN25183_c0_g1_i1	dmso	control	5,50066191	0,0994009	1,93E-17	1,71E-15
TRINITY_DN21744_c0_g1_i1	dmso	control	5,48146831	0,08540881	8,82E-18	8,12E-16
TRINITY_DN15414_c0_g1_i1	dmso	control	5,35339094	1,11941811	6,51E-28	1,54E-25
TRINITY_DN24048_c0_g1_i1	dmso	control	5,2921991	1,07988128	6,21E-27	1,32E-24
TRINITY_DN24800_c0_g1_i1	dmso	control	5,25616325	0,39294582	5,36E-19	5,69E-17
TRINITY_DN26211_c0_g1_i1	dmso	control	5,22062452	-0,1391093	1,62E-14	1,05E-12
TRINITY_DN12087_c0_g1_i1	dmso	control	5,20150958	2,04041247	2,00E-40	1,11E-37
TRINITY_DN7730_c0_g1_i1	dmso	control	5,11670493	1,35839763	3,91E-30	1,13E-27
TRINITY_DN7478_c0_g1_i1	dmso	control	4,94137295	2,40357482	9,84E-44	6,18E-41
TRINITY_DN19844_c0_g1_i1	dmso	control	4,90916897	2,3070805	5,25E-42	3,03E-39
TRINITY_DN43861_c0_g1_i1	dmso	control	4,87988615	0,39982216	1,49E-18	1,49E-16
TRINITY_DN13627_c0_g1_i1	dmso	control	4,76615755	1,36640545	1,03E-27	2,37E-25
TRINITY_DN13899_c0_g1_i1	dmso	control	4,70509227	3,30924624	6,06E-67	2,19E-63
TRINITY_DN15148_c0_g1_i1	dmso	control	4,6848804	3,54573907	3,04E-63	6,27E-60
TRINITY_DN33832_c0_g1_i1	dmso	control	4,60001212	-0,2108762	4,31E-13	2,35E-11
TRINITY_DN4579_c0_g1_i1	dmso	control	4,51458775	-0,2853569	1,47E-12	7,37E-11
TRINITY_DN28220_c0_g1_i1	dmso	control	4,45774	-0,3462589	4,32E-12	1,97E-10
TRINITY_DN32597_c0_g1_i1	dmso	control	4,36179729	2,89552187	5,34E-50	4,28E-47
TRINITY_DN33490_c0_g1_i1	dmso	control	4,30083428	0,17044338	2,66E-14	1,69E-12
TRINITY_DN3014_c0_g1_i1	dmso	control	4,2839123	3,2479122	1,34E-60	1,95E-57
TRINITY_DN7585_c0_g1_i1	dmso	control	4,23764156	2,48919626	1,81E-34	7,06E-32

TRINITY_DN35882_c0_g1_i1	dmso	control	4,21003425	1,80322437	4,39E-31	1,29E-28
TRINITY_DN57543_c0_g1_i1	dmso	control	4,19915324	-0,5515206	3,20E-10	1,09E-08
TRINITY_DN17038_c0_g1_i1	dmso	control	4,13624194	1,71545873	6,96E-27	1,46E-24
TRINITY_DN56314_c0_g1_i1	dmso	control	4,09867378	0,90891123	1,70E-19	1,87E-17
TRINITY_DN12532_c0_g1_i1	dmso	control	4,08262099	1,33725127	2,46E-22	3,45E-20
TRINITY_DN3942_c0_g1_i1	dmso	control	4,00472372	0,34592512	1,65E-15	1,18E-13
TRINITY_DN24435_c0_g1_i1	dmso	control	3,98021512	1,14457669	8,55E-19	8,76E-17
TRINITY_DN14610_c0_g1_i1	dmso	control	3,967074	0,64476855	4,94E-17	4,25E-15
TRINITY_DN1732_c0_g1_i1	dmso	control	3,95103075	1,9177001	5,62E-28	1,38E-25
TRINITY_DN22265_c0_g1_i1	dmso	control	3,93412935	1,89360842	2,07E-25	3,83E-23
TRINITY_DN27243_c0_g1_i1	dmso	control	3,91332138	2,20134294	4,60E-32	1,54E-29
TRINITY_DN28411_c0_g1_i1	dmso	control	3,87803475	0,04363143	1,91E-12	9,30E-11
TRINITY_DN3777_c0_g1_i1	dmso	control	3,86588719	2,21633011	4,81E-09	1,32E-07
TRINITY_DN3118_c0_g1_i1	dmso	control	3,80083774	1,51284142	2,14E-22	3,08E-20
TRINITY_DN5661_c0_g1_i1	dmso	control	3,7270885	6,64211005	6,69E-34	2,42E-31
TRINITY_DN33851_c0_g1_i1	dmso	control	3,71625535	1,29074575	3,55E-20	4,23E-18
TRINITY_DN54531_c0_g1_i1	dmso	control	3,69535875	0,66723849	7,21E-17	6,13E-15
TRINITY_DN22788_c0_g1_i1	dmso	control	3,66734223	2,58718398	4,93E-37	2,23E-34
TRINITY_DN19460_c0_g1_i1	dmso	control	3,66176989	4,23063356	2,29E-59	2,76E-56
TRINITY_DN23319_c0_g1_i1	dmso	control	3,65609928	-0,1490026	2,04E-10	7,14E-09
TRINITY_DN34107_c0_g1_i1	dmso	control	3,63117515	2,44989738	8,95E-27	1,85E-24
TRINITY_DN13418_c0_g1_i1	dmso	control	3,60178281	-0,0228025	3,43E-12	1,59E-10
TRINITY_DN22273_c0_g1_i1	dmso	control	3,59577979	3,78041297	1,35E-60	1,95E-57
TRINITY_DN16982_c0_g1_i1	dmso	control	3,59007726	2,47808455	2,67E-34	1,02E-31
TRINITY_DN33188_c0_g1_i1	dmso	control	3,56026125	1,48308407	1,02E-21	1,40E-19
TRINITY_DN152_c0_g1_i1	dmso	control	3,51534236	5,19652097	3,45E-33	1,22E-30
TRINITY_DN34410_c0_g1_i1	dmso	control	3,43692204	0,00631153	2,09E-10	7,29E-09
TRINITY_DN39107_c0_g1_i1	dmso	control	3,42803799	0,31583598	1,57E-12	7,76E-11
TRINITY_DN25236_c0_g1_i1	dmso	control	3,36290965	3,71423563	7,24E-45	5,23E-42
TRINITY_DN13988_c0_g1_i1	dmso	control	3,36132614	0,71707894	3,55E-14	2,21E-12
TRINITY_DN44422_c0_g1_i1	dmso	control	3,30816476	-0,0674004	2,71E-09	7,91E-08

TRINITY_DN3362_c0_g1_i1	dmso	control	3,30083451	1,7616712	3,99E-19	4,30E-17
TRINITY_DN21762_c0_g1_i1	dmso	control	3,26213243	0,03070443	4,66E-11	1,81E-09
TRINITY_DN19726_c0_g1_i1	dmso	control	3,21512904	1,94916362	4,54E-23	6,97E-21
TRINITY_DN12473_c0_g1_i1	dmso	control	3,16963383	5,54772136	1,26E-12	6,47E-11
TRINITY_DN6435_c0_g1_i1	dmso	control	3,1650692	4,64205417	3,00E-40	1,61E-37
TRINITY_DN45980_c0_g1_i1	dmso	control	3,14524208	-0,3758013	6,40E-08	1,47E-06
TRINITY_DN22516_c0_g1_i1	dmso	control	3,14149087	1,59222562	5,17E-20	6,02E-18
TRINITY_DN11756_c0_g1_i1	dmso	control	3,13564145	4,24721406	6,67E-53	6,88E-50
TRINITY_DN8591_c0_g1_i1	dmso	control	3,13535966	-0,3749302	1,43E-07	3,06E-06
TRINITY_DN4317_c0_g1_i1	dmso	control	3,11836406	0,65830837	1,64E-13	9,34E-12
TRINITY_DN19951_c0_g1_i1	dmso	control	3,0920087	0,64473471	2,49E-13	1,40E-11
TRINITY_DN42973_c0_g1_i1	dmso	control	3,07057488	0,54954161	9,83E-10	3,08E-08
TRINITY_DN2310_c0_g1_i1	dmso	control	3,02990314	2,41776128	5,57E-27	1,20E-24
TRINITY_DN12898_c0_g1_i1	dmso	control	3,00680544	3,5396557	2,89E-37	1,35E-34
TRINITY_DN32323_c0_g1_i1	dmso	control	2,97487617	2,03772494	3,37E-15	2,35E-13
TRINITY_DN1781_c0_g1_i1	dmso	control	2,92658667	5,30333271	7,13E-78	3,43E-74
TRINITY_DN23130_c0_g1_i1	dmso	control	2,92238341	2,48485393	8,93E-25	1,59E-22
TRINITY_DN14897_c0_g1_i1	dmso	control	2,88754618	-0,1492245	1,43E-08	3,68E-07
TRINITY_DN45753_c0_g1_i1	dmso	control	2,85904824	1,30648205	9,04E-16	6,80E-14
TRINITY_DN13280_c0_g1_i1	dmso	control	2,8402165	3,04287076	2,94E-12	1,39E-10
TRINITY_DN33480_c0_g1_i1	dmso	control	2,8090417	1,82023413	1,60E-19	1,78E-17
TRINITY_DN8633_c0_g1_i1	dmso	control	2,7995424	1,45957499	2,47E-16	1,99E-14
TRINITY_DN36970_c0_g1_i1	dmso	control	2,79641257	0,29573937	3,58E-10	1,21E-08
TRINITY_DN12869_c0_g1_i1	dmso	control	2,79491289	1,41585819	3,18E-15	2,24E-13
TRINITY_DN21333_c0_g1_i1	dmso	control	2,76665884	3,7210083	1,67E-27	3,65E-25
TRINITY_DN22745_c0_g1_i1	dmso	control	2,76116415	2,790649	1,12E-27	2,52E-25
TRINITY_DN29317_c0_g1_i1	dmso	control	2,75508265	1,6706653	1,48E-15	1,07E-13
TRINITY_DN6474_c0_g1_i1	dmso	control	2,74684064	3,04546414	1,76E-31	5,65E-29
TRINITY_DN12761_c0_g1_i1	dmso	control	2,70320486	2,18193035	4,27E-08	1,00E-06
TRINITY_DN37877_c0_g1_i1	dmso	control	2,6823359	-0,1788554	2,04E-07	4,20E-06
TRINITY_DN16776_c0_g1_i1	dmso	control	2,67487116	0,29085229	3,47E-09	9,80E-08

TRINITY_DN43259_c0_g1_i1	dmso	control	2,64446475	2,82597495	6,94E-23	1,04E-20
TRINITY_DN348_c0_g1_i1	dmso	control	2,63367394	3,38668475	1,11E-22	1,61E-20
TRINITY_DN20488_c0_g1_i1	dmso	control	2,6299605	1,3442514	6,16E-09	1,67E-07
TRINITY_DN32931_c0_g1_i1	dmso	control	2,60651961	8,02676543	3,13E-51	2,66E-48
TRINITY_DN3145_c0_g1_i1	dmso	control	2,57556874	2,66140126	1,44E-23	2,31E-21
TRINITY_DN12322_c0_g1_i1	dmso	control	2,56789494	5,50463563	8,00E-52	7,22E-49
TRINITY_DN36775_c0_g1_i1	dmso	control	2,53892848	0,00938299	1,18E-07	2,58E-06
TRINITY_DN48332_c0_g1_i1	dmso	control	2,53776509	0,44570409	5,87E-08	1,35E-06
TRINITY_DN33132_c0_g1_i1	dmso	control	2,5157502	3,24947524	1,28E-19	1,47E-17
TRINITY_DN27989_c0_g1_i1	dmso	control	2,4951253	3,33079921	3,84E-14	2,37E-12
TRINITY_DN33970_c0_g1_i1	dmso	control	2,48651159	1,15104571	3,88E-12	1,78E-10
TRINITY_DN3935_c0_g1_i1	dmso	control	2,48608792	0,12119813	1,15E-07	2,53E-06
TRINITY_DN7768_c0_g1_i1	dmso	control	2,47489413	3,81124567	2,18E-29	5,93E-27
TRINITY_DN27739_c0_g1_i1	dmso	control	2,47460318	0,61175017	1,59E-09	4,80E-08
TRINITY_DN11315_c0_g1_i1	dmso	control	2,43549278	5,69944504	8,03E-67	2,32E-63
TRINITY_DN43484_c0_g1_i1	dmso	control	2,42957491	-0,0690437	4,25E-07	8,14E-06
TRINITY_DN24088_c0_g1_i1	dmso	control	2,42488824	1,55614863	2,57E-12	1,23E-10
TRINITY_DN29453_c0_g1_i1	dmso	control	2,41577587	-0,4088009	2,51E-06	4,01E-05
TRINITY_DN9129_c0_g1_i1	dmso	control	2,41239622	1,04896673	1,40E-10	5,07E-09
TRINITY_DN12755_c0_g1_i1	dmso	control	2,40999489	3,94718964	1,00E-22	1,49E-20
TRINITY_DN22307_c0_g1_i1	dmso	control	2,40605989	1,32799968	3,15E-12	1,48E-10
TRINITY_DN3473_c0_g1_i1	dmso	control	2,40134127	1,34588262	2,80E-12	1,33E-10
TRINITY_DN26733_c0_g1_i1	dmso	control	2,39089409	1,05896212	2,02E-10	7,10E-09
TRINITY_DN60644_c0_g1_i1	dmso	control	2,38091851	-0,2041713	1,81E-06	2,99E-05
TRINITY_DN13676_c0_g1_i1	dmso	control	2,37887239	1,9381353	2,24E-09	6,62E-08
TRINITY_DN13233_c0_g1_i1	dmso	control	2,37378437	4,58514707	3,90E-31	1,17E-28
TRINITY_DN41128_c0_g1_i1	dmso	control	2,37263153	0,7042469	6,37E-10	2,07E-08
TRINITY_DN20952_c0_g1_i1	dmso	control	2,37009275	0,77980081	3,11E-09	8,86E-08
TRINITY_DN33019_c0_g1_i1	dmso	control	2,36924951	6,33837051	9,33E-65	2,25E-61
TRINITY_DN12013_c0_g1_i1	dmso	control	2,36006947	5,66322393	1,60E-37	7,72E-35
TRINITY_DN19147_c0_g1_i1	dmso	control	2,35352995	3,30292256	5,11E-07	9,55E-06

TRINITY_DN34826_c0_g1_i1	dmso	control	2,33417177	2,77646338	7,77E-21	9,75E-19
TRINITY_DN14619_c0_g1_i1	dmso	control	2,32324772	1,66314398	1,55E-12	7,66E-11
TRINITY_DN17802_c0_g1_i1	dmso	control	2,31292767	-0,376998	3,79E-06	5,76E-05
TRINITY_DN19662_c0_g1_i1	dmso	control	2,30818451	2,21873423	3,25E-16	2,60E-14
TRINITY_DN30315_c0_g1_i1	dmso	control	2,30739183	-0,2817127	2,58E-06	4,10E-05
TRINITY_DN37332_c0_g1_i1	dmso	control	2,27876169	0,57075682	5,82E-08	1,35E-06
TRINITY_DN1656_c0_g1_i1	dmso	control	2,27412438	5,38489097	2,50E-28	6,22E-26
TRINITY_DN23818_c0_g1_i1	dmso	control	2,2721407	3,04854151	2,11E-23	3,28E-21
TRINITY_DN15334_c0_g1_i1	dmso	control	2,26786021	0,71244686	4,76E-09	1,31E-07
TRINITY_DN36204_c0_g1_i1	dmso	control	2,25477171	1,62262594	4,54E-10	1,50E-08
TRINITY_DN2849_c0_g1_i1	dmso	control	2,23013533	3,30037565	4,60E-18	4,31E-16
TRINITY_DN51975_c0_g1_i1	dmso	control	2,22894488	1,36968145	3,37E-09	9,53E-08
TRINITY_DN19454_c0_g1_i1	dmso	control	2,20326866	0,16932722	2,39E-05	0,00029867
TRINITY_DN4305_c0_g1_i1	dmso	control	2,19413666	0,04108324	1,95E-06	3,18E-05
TRINITY_DN7774_c0_g1_i1	dmso	control	2,19265311	1,59629199	1,36E-11	5,80E-10
TRINITY_DN25984_c0_g1_i1	dmso	control	2,19070373	2,86431228	1,47E-19	1,66E-17
TRINITY_DN45070_c0_g1_i1	dmso	control	2,16194305	2,18508797	4,27E-15	2,95E-13
TRINITY_DN25709_c0_g1_i1	dmso	control	2,15960175	-0,1191399	7,22E-06	0,00010415
TRINITY_DN2887_c0_g1_i1	dmso	control	2,14211142	2,69130465	8,29E-19	8,61E-17
TRINITY_DN15183_c0_g1_i1	dmso	control	2,13762807	3,78320985	6,56E-22	9,02E-20
TRINITY_DN8798_c0_g1_i1	dmso	control	2,13420423	0,5663384	5,94E-08	1,37E-06
TRINITY_DN43825_c0_g1_i1	dmso	control	2,12304296	0,73332748	1,79E-08	4,49E-07
TRINITY_DN643_c0_g1_i1	dmso	control	2,12266401	6,19580736	3,94E-09	1,10E-07
TRINITY_DN17103_c0_g1_i1	dmso	control	2,10742167	4,81257535	3,40E-36	1,45E-33
TRINITY_DN25898_c0_g1_i1	dmso	control	2,10557573	1,55753273	3,37E-08	8,03E-07
TRINITY_DN45291_c0_g1_i1	dmso	control	2,10515602	1,22436785	2,89E-09	8,32E-08
TRINITY_DN20432_c0_g1_i1	dmso	control	2,10441919	6,49429971	1,85E-24	3,23E-22
TRINITY_DN20292_c0_g1_i1	dmso	control	2,09394891	2,88756482	4,03E-17	3,50E-15
TRINITY_DN15687_c0_g1_i1	dmso	control	2,06652181	2,97423084	2,26E-11	9,29E-10
TRINITY_DN15824_c0_g1_i1	dmso	control	2,05547821	0,74417957	6,20E-07	1,14E-05
TRINITY_DN20090_c0_g1_i1	dmso	control	2,05430339	1,3936269	2,19E-08	5,41E-07

TRINITY_DN6200_c0_g1_i1	dmso	control	2,04862947	4,56239209	3,13E-09	8,89E-08
TRINITY_DN21833_c0_g1_i1	dmso	control	2,04628563	0,88528987	1,80E-06	2,98E-05
TRINITY_DN20896_c0_g1_i1	dmso	control	2,04457925	1,96725427	9,18E-13	4,88E-11
TRINITY_DN7782_c0_g1_i1	dmso	control	2,04225232	3,77797389	1,16E-14	7,70E-13
TRINITY_DN32348_c0_g1_i1	dmso	control	2,04100098	3,04602984	1,05E-09	3,27E-08
TRINITY_DN6357_c0_g1_i1	dmso	control	2,03997992	6,07370996	2,16E-58	2,40E-55
TRINITY_DN42547_c0_g1_i1	dmso	control	2,03524433	1,30009062	4,92E-06	7,35E-05
TRINITY_DN35429_c0_g1_i1	dmso	control	2,02180884	0,01206838	1,02E-05	0,00014289
TRINITY_DN3648_c0_g1_i1	dmso	control	2,02060041	2,36930137	4,40E-12	2,00E-10
TRINITY_DN19691_c0_g1_i1	dmso	control	2,01898175	0,70905392	7,52E-05	0,00082007
TRINITY_DN14084_c0_g1_i1	dmso	control	2,01224931	3,82207164	1,47E-16	1,20E-14
TRINITY_DN33258_c0_g1_i1	dmso	control	2,01119621	3,66818799	1,10E-22	1,61E-20
TRINITY_DN1182_c0_g1_i1	dmso	control	2,0034369	1,77988824	3,82E-11	1,51E-09
TRINITY_DN13928_c0_g1_i1	dmso	control	1,99695338	2,17238232	3,38E-10	1,15E-08
TRINITY_DN2132_c0_g1_i1	dmso	control	1,99201193	3,9121807	1,80E-10	6,35E-09
TRINITY_DN48528_c0_g1_i1	dmso	control	1,98934011	-0,3455519	3,46E-05	0,00041463
TRINITY_DN15154_c0_g1_i1	dmso	control	1,98316479	2,82479183	3,30E-15	2,32E-13
TRINITY_DN4469_c0_g1_i1	dmso	control	1,98121548	1,45860375	0,00019062	0,00180914
TRINITY_DN37203_c0_g1_i1	dmso	control	1,97490593	-0,4234368	4,19E-05	0,00049045
TRINITY_DN34140_c0_g1_i1	dmso	control	1,97348695	0,10870849	8,67E-06	0,00012238
TRINITY_DN34978_c0_g1_i1	dmso	control	1,96889761	0,78174832	1,84E-07	3,85E-06
TRINITY_DN24207_c0_g1_i1	dmso	control	1,96379386	2,42369915	3,08E-11	1,23E-09
TRINITY_DN49208_c0_g1_i1	dmso	control	1,95199417	-0,1928089	1,93E-05	0,00024828
TRINITY_DN24866_c0_g1_i1	dmso	control	1,93548555	0,07150248	1,14E-05	0,000158
TRINITY_DN24304_c0_g1_i1	dmso	control	1,93152755	1,07622454	1,42E-06	2,42E-05
TRINITY_DN42477_c0_g1_i1	dmso	control	1,92805976	1,73565379	5,13E-11	1,97E-09
TRINITY_DN23881_c0_g1_i1	dmso	control	1,9278007	3,6765304	9,51E-21	1,18E-18
TRINITY_DN2168_c0_g1_i1	dmso	control	1,92468715	4,53143292	2,74E-09	7,98E-08
TRINITY_DN42014_c0_g1_i1	dmso	control	1,9238329	0,79181301	2,97E-07	5,87E-06
TRINITY_DN59535_c0_g1_i1	dmso	control	1,90345672	-0,2381455	5,51E-05	0,00062577
TRINITY_DN26141_c0_g1_i1	dmso	control	1,90154699	0,76506212	2,35E-07	4,76E-06

TRINITY_DN27690_c0_g1_i1	dmso	control	1,89810672	1,52577441	1,89E-07	3,94E-06
TRINITY_DN8508_c0_g1_i1	dmso	control	1,8966663	2,52615372	3,56E-10	1,20E-08
TRINITY_DN23634_c0_g1_i1	dmso	control	1,88178113	0,74784371	4,34E-07	8,28E-06
TRINITY_DN37515_c0_g1_i1	dmso	control	1,86884712	1,2622122	3,73E-06	5,69E-05
TRINITY_DN5732_c0_g1_i1	dmso	control	1,86699681	3,62608598	4,23E-12	1,93E-10
TRINITY_DN13564_c0_g1_i1	dmso	control	1,85464331	2,70426601	3,63E-12	1,68E-10
TRINITY_DN23156_c0_g1_i1	dmso	control	1,84471021	2,82176103	6,58E-14	3,96E-12
TRINITY_DN3872_c0_g1_i1	dmso	control	1,84430166	4,89136094	5,13E-21	6,56E-19
TRINITY_DN20769_c0_g1_i1	dmso	control	1,84325512	2,09339268	5,06E-11	1,95E-09
TRINITY_DN2550_c0_g1_i1	dmso	control	1,84102246	3,81518217	3,97E-22	5,52E-20
TRINITY_DN19802_c0_g1_i1	dmso	control	1,84048483	3,74105053	2,19E-19	2,40E-17
TRINITY_DN14516_c0_g1_i1	dmso	control	1,83932551	3,07764241	1,59E-14	1,04E-12
TRINITY_DN41630_c0_g1_i1	dmso	control	1,83787315	0,20338588	0,00010203	0,00106315
TRINITY_DN12964_c0_g1_i1	dmso	control	1,83148368	3,89146564	1,22E-15	8,97E-14
TRINITY_DN7136_c0_g1_i1	dmso	control	1,83030234	2,99388987	6,12E-16	4,78E-14
TRINITY_DN32174_c0_g1_i1	dmso	control	1,82576747	-0,2610069	0,00021805	0,00202681
TRINITY_DN19388_c0_g1_i1	dmso	control	1,82214104	2,54370316	1,25E-12	6,47E-11
TRINITY_DN22249_c0_g1_i1	dmso	control	1,81674726	0,35435078	5,75E-05	0,0006513
TRINITY_DN32817_c0_g1_i1	dmso	control	1,8079575	2,13797327	7,97E-11	2,98E-09
TRINITY_DN15279_c0_g1_i1	dmso	control	1,7995688	2,25588453	7,23E-12	3,21E-10
TRINITY_DN43533_c0_g1_i1	dmso	control	1,79150906	0,38855212	6,40E-06	9,34E-05
TRINITY_DN42403_c0_g1_i1	dmso	control	1,78609835	0,41554011	2,78E-05	0,00034203
TRINITY_DN41921_c0_g1_i1	dmso	control	1,78559809	0,26073606	0,00026296	0,00238734
TRINITY_DN36956_c0_g1_i1	dmso	control	1,78532307	0,1715158	3,92E-05	0,00046341
TRINITY_DN3363_c0_g1_i1	dmso	control	1,78246009	1,30152681	1,28E-06	2,18E-05
TRINITY_DN49125_c0_g1_i1	dmso	control	1,78131325	0,57284576	2,94E-05	0,00035831
TRINITY_DN32912_c0_g1_i1	dmso	control	1,77733902	3,46422705	2,60E-12	1,24E-10
TRINITY_DN63705_c0_g1_i1	dmso	control	1,77604951	0,07566521	0,00019349	0,00183352
TRINITY_DN9481_c0_g1_i1	dmso	control	1,77512851	-0,1927107	0,00012304	0,00124902
TRINITY_DN32468_c0_g1_i1	dmso	control	1,77416681	2,28337968	1,48E-07	3,16E-06
TRINITY_DN12336_c0_g1_i1	dmso	control	1,77287855	3,98787719	1,30E-18	1,30E-16

TRINITY_DN13682_c0_g1_i1	dmso	control	1,76393277	4,68398303	1,43E-27	3,17E-25
TRINITY_DN19621_c0_g1_i1	dmso	control	1,76319993	2,42814762	5,43E-05	0,00061797
TRINITY_DN17591_c0_g1_i1	dmso	control	1,75874274	0,53931287	4,61E-05	0,00053346
TRINITY_DN14987_c0_g1_i1	dmso	control	1,75153836	2,14418955	1,95E-10	6,84E-09
TRINITY_DN1313_c0_g1_i1	dmso	control	1,74669016	4,26519799	2,21E-21	2,91E-19
TRINITY_DN28963_c0_g1_i1	dmso	control	1,74042746	0,73161127	1,92E-06	3,15E-05
TRINITY_DN5094_c0_g1_i1	dmso	control	1,73975646	4,85528373	5,53E-29	1,43E-26
TRINITY_DN11781_c0_g1_i1	dmso	control	1,73903121	4,73527466	1,38E-26	2,80E-24
TRINITY_DN3043_c0_g1_i1	dmso	control	1,73689988	4,37331248	9,38E-09	2,49E-07
TRINITY_DN25687_c0_g1_i1	dmso	control	1,73388843	0,86517108	1,21E-05	0,00016696
TRINITY_DN2802_c0_g1_i1	dmso	control	1,73102909	4,27998161	2,42E-21	3,14E-19
TRINITY_DN10854_c0_g1_i1	dmso	control	1,72991233	6,4349028	6,65E-36	2,67E-33
TRINITY_DN15081_c0_g1_i1	dmso	control	1,7282076	2,02746342	2,59E-09	7,61E-08
TRINITY_DN11126_c0_g1_i1	dmso	control	1,72819267	3,90056263	8,37E-19	8,64E-17
TRINITY_DN14029_c0_g1_i1	dmso	control	1,72783766	1,84799845	2,46E-09	7,26E-08
TRINITY_DN17088_c0_g1_i1	dmso	control	1,72226543	0,91413342	0,00033051	0,00292001
TRINITY_DN12344_c0_g1_i1	dmso	control	1,72192644	3,18953841	7,28E-15	4,98E-13
TRINITY_DN17302_c0_g1_i1	dmso	control	1,71694074	0,85290703	9,78E-07	1,71E-05
TRINITY_DN22752_c0_g1_i1	dmso	control	1,71522386	1,62947126	6,89E-09	1,86E-07
TRINITY_DN16748_c0_g1_i1	dmso	control	1,71355392	2,20395261	1,48E-10	5,32E-09
TRINITY_DN12123_c0_g1_i1	dmso	control	1,71084599	3,80995665	1,94E-18	1,89E-16
TRINITY_DN13576_c0_g1_i1	dmso	control	1,71022786	1,52631408	7,61E-07	1,37E-05
TRINITY_DN1492_c0_g1_i1	dmso	control	1,70008388	7,7969178	4,17E-38	2,08E-35
TRINITY_DN6396_c0_g1_i1	dmso	control	1,69992495	3,14625557	2,73E-11	1,10E-09
TRINITY_DN349_c0_g1_i1	dmso	control	1,6968641	6,29562263	1,61E-17	1,45E-15
TRINITY_DN11835_c0_g1_i1	dmso	control	1,69600953	3,37188751	4,36E-13	2,37E-11
TRINITY_DN13043_c0_g1_i1	dmso	control	1,69412852	3,24370636	4,61E-14	2,81E-12
TRINITY_DN2953_c0_g1_i1	dmso	control	1,69344438	1,68408639	2,64E-08	6,41E-07
TRINITY_DN30327_c0_g1_i1	dmso	control	1,68731881	-0,1030556	0,000238	0,00218977
TRINITY_DN28942_c0_g1_i1	dmso	control	1,68588581	0,00793236	0,00012738	0,00128315
TRINITY_DN60799_c0_g1_i1	dmso	control	1,68381381	-0,2516115	0,000257	0,00234072

TRINITY_DN28060_c0_g1_i1	dmso	control	1,67562324	-0,0167262	0,00016666	0,00161244
TRINITY_DN40777_c0_g1_i1	dmso	control	1,6738833	0,06013969	8,21E-05	0,00088093
TRINITY_DN12428_c0_g1_i1	dmso	control	1,67039912	3,51982115	2,46E-13	1,39E-11
TRINITY_DN21813_c0_g1_i1	dmso	control	1,66937221	3,8552871	7,71E-16	5,89E-14
TRINITY_DN8410_c0_g1_i1	dmso	control	1,6645799	-0,0498768	0,00017072	0,00164736
TRINITY_DN44412_c0_g1_i1	dmso	control	1,66225604	0,39575672	9,37E-05	0,00099009
TRINITY_DN12904_c0_g1_i1	dmso	control	1,66102502	3,25579408	1,12E-13	6,53E-12
TRINITY_DN39817_c0_g1_i1	dmso	control	1,65217641	1,61495833	0,00059004	0,00476149
TRINITY_DN35358_c0_g1_i1	dmso	control	1,64991753	0,98455884	1,77E-05	0,00023072
TRINITY_DN33134_c0_g1_i1	dmso	control	1,64723202	3,4289179	7,38E-16	5,67E-14
TRINITY_DN3343_c0_g1_i1	dmso	control	1,64310842	4,17043582	3,18E-13	1,77E-11
TRINITY_DN27795_c0_g1_i1	dmso	control	1,64203393	2,8464505	4,55E-12	2,06E-10
TRINITY_DN9184_c0_g1_i1	dmso	control	1,64013721	3,77629443	2,25E-15	1,59E-13
TRINITY_DN8611_c0_g1_i1	dmso	control	1,63806345	1,48382116	1,35E-07	2,92E-06
TRINITY_DN55625_c0_g1_i1	dmso	control	1,63686152	-0,0123738	0,000181	0,00173145
TRINITY_DN40948_c0_g1_i1	dmso	control	1,63411505	0,3596979	2,97E-05	0,00036113
TRINITY_DN9565_c0_g1_i1	dmso	control	1,63289265	1,06577909	0,00602458	0,03123654
TRINITY_DN32511_c0_g1_i1	dmso	control	1,63015586	3,93600614	1,30E-10	4,72E-09
TRINITY_DN22383_c0_g1_i1	dmso	control	1,62703207	3,65514895	1,63E-08	4,13E-07
TRINITY_DN4685_c0_g1_i1	dmso	control	1,61839936	7,07563474	4,47E-39	2,31E-36
TRINITY_DN25584_c0_g1_i1	dmso	control	1,61839734	2,38368145	1,24E-09	3,80E-08
TRINITY_DN22598_c0_g1_i1	dmso	control	1,61397991	1,23176451	1,72E-05	0,00022475
TRINITY_DN24214_c0_g1_i1	dmso	control	1,60617643	2,52032193	2,91E-10	1,00E-08
TRINITY_DN11191_c0_g1_i1	dmso	control	1,6030225	5,133323	4,51E-20	5,30E-18
TRINITY_DN19830_c0_g1_i1	dmso	control	1,59849287	2,30227301	1,54E-09	4,66E-08
TRINITY_DN30519_c0_g1_i1	dmso	control	1,59822896	-0,2523006	0,00065549	0,00521968
TRINITY_DN34339_c0_g1_i1	dmso	control	1,58658634	0,70143091	3,14E-05	0,00038132
TRINITY_DN23017_c0_g1_i1	dmso	control	1,58627512	0,50612664	7,48E-05	0,00081683
TRINITY_DN6849_c0_g1_i1	dmso	control	1,58621258	4,24499203	9,11E-20	1,05E-17
TRINITY_DN7831_c0_g1_i1	dmso	control	1,58606053	2,48906235	2,91E-10	1,00E-08
TRINITY_DN5269_c0_g1_i1	dmso	control	1,58314437	5,72381585	5,15E-24	8,64E-22

TRINITY_DN10160_c0_g1_i1	dmso	control	1,58279331	-0,1895464	0,00037973	0,00327277
TRINITY_DN25087_c0_g1_i1	dmso	control	1,58063607	3,34959018	1,32E-14	8,73E-13
TRINITY_DN12195_c0_g1_i1	dmso	control	1,57927592	2,59357682	4,45E-09	1,24E-07
TRINITY_DN27022_c0_g1_i1	dmso	control	1,57903848	3,14751204	8,94E-14	5,25E-12
TRINITY_DN27626_c0_g1_i1	dmso	control	1,5759709	1,29179574	1,93E-06	3,16E-05
TRINITY_DN22943_c0_g1_i1	dmso	control	1,57069082	2,9493184	2,19E-11	9,05E-10
TRINITY_DN9308_c0_g1_i1	dmso	control	1,56451365	1,9147704	5,14E-08	1,19E-06
TRINITY_DN562_c0_g1_i1	dmso	control	1,56127343	6,27116174	4,06E-26	7,72E-24
TRINITY_DN24653_c0_g1_i1	dmso	control	1,55677124	2,33553652	1,90E-09	5,68E-08
TRINITY_DN7900_c0_g1_i1	dmso	control	1,55634803	1,2194705	4,23E-05	0,00049422
TRINITY_DN47973_c0_g1_i1	dmso	control	1,54506365	0,01301378	0,00060918	0,00489874
TRINITY_DN6643_c0_g1_i1	dmso	control	1,54451542	4,01396049	8,27E-16	6,26E-14
TRINITY_DN20401_c0_g1_i1	dmso	control	1,5419703	0,93661774	2,15E-05	0,00027245
TRINITY_DN19664_c0_g1_i1	dmso	control	1,54104903	4,63032619	2,22E-07	4,52E-06
TRINITY_DN826_c0_g1_i1	dmso	control	1,53719147	7,82886013	1,76E-23	2,79E-21
TRINITY_DN6269_c0_g1_i1	dmso	control	1,53139468	5,91656983	3,51E-31	1,08E-28
TRINITY_DN3582_c0_g1_i1	dmso	control	1,52752208	2,8149451	3,10E-11	1,23E-09
TRINITY_DN2721_c0_g1_i1	dmso	control	1,52734178	5,30726378	1,79E-18	1,76E-16
TRINITY_DN615_c0_g1_i1	dmso	control	1,52601114	5,82998922	6,39E-28	1,54E-25
TRINITY_DN27439_c0_g1_i1	dmso	control	1,52437246	1,37447334	3,06E-06	4,76E-05
TRINITY_DN7961_c0_g1_i1	dmso	control	1,5241341	2,41897465	2,64E-07	5,30E-06
TRINITY_DN35505_c0_g1_i1	dmso	control	1,52280112	0,73606418	3,30E-05	0,00039704
TRINITY_DN8889_c0_g1_i1	dmso	control	1,51894348	0,84056095	2,99E-05	0,00036421
TRINITY_DN13740_c0_g1_i1	dmso	control	1,51767845	3,86045791	7,20E-16	5,56E-14
TRINITY_DN3039_c0_g1_i1	dmso	control	1,51718279	3,52400722	3,84E-13	2,11E-11
TRINITY_DN3649_c0_g1_i1	dmso	control	1,51605279	4,7723785	2,20E-20	2,69E-18
TRINITY_DN9976_c0_g1_i1	dmso	control	1,51536995	1,04292206	0,01357277	0,05891187
TRINITY_DN33105_c0_g1_i1	dmso	control	1,51318143	1,04783199	6,00E-06	8,80E-05
TRINITY_DN6060_c0_g1_i1	dmso	control	1,51102119	5,2896518	3,21E-26	6,36E-24
TRINITY_DN9398_c0_g1_i1	dmso	control	1,50784485	2,50185166	8,54E-10	2,72E-08
TRINITY_DN23062_c0_g1_i1	dmso	control	1,50639223	3,81425462	7,21E-15	4,96E-13

TRINITY_DN5078_c0_g1_i1	dmso	control	1,50505773	7,06917029	5,60E-33	1,93E-30
TRINITY_DN13466_c0_g1_i1	dmso	control	1,50426021	2,16723612	4,32E-08	1,02E-06
TRINITY_DN3569_c0_g1_i1	dmso	control	1,50418885	2,48491289	8,81E-10	2,79E-08
TRINITY_DN49595_c0_g1_i1	dmso	control	1,50372287	0,09535876	0,00029891	0,00267684
TRINITY_DN23576_c0_g1_i1	dmso	control	1,50363731	1,65151155	5,73E-07	1,06E-05
TRINITY_DN1717_c0_g1_i1	dmso	control	1,50213824	3,67590623	4,51E-10	1,49E-08
TRINITY_DN922_c0_g1_i1	dmso	control	1,50185559	10,3499376	8,76E-45	6,03E-42
TRINITY_DN1427_c0_g1_i1	dmso	control	1,50182942	3,21250267	5,01E-09	1,37E-07
TRINITY_DN21652_c0_g1_i1	dmso	control	1,4972815	1,6236403	4,70E-07	8,86E-06
TRINITY_DN12376_c0_g1_i1	dmso	control	1,49725267	4,20578158	2,97E-17	2,60E-15
TRINITY_DN21960_c0_g1_i1	dmso	control	1,49640674	1,82529067	6,08E-07	1,12E-05
TRINITY_DN36905_c0_g1_i1	dmso	control	1,49445413	0,13272878	0,00042024	0,00356452
TRINITY_DN30586_c0_g1_i1	dmso	control	1,49284666	-0,0666684	0,00073445	0,00573153
TRINITY_DN20888_c0_g1_i1	dmso	control	1,49143373	2,78249232	1,17E-10	4,29E-09
TRINITY_DN6980_c0_g1_i1	dmso	control	1,48638612	3,25737874	4,03E-10	1,34E-08
TRINITY_DN30308_c0_g1_i1	dmso	control	1,48621818	2,27154757	1,09E-08	2,88E-07
TRINITY_DN19130_c0_g1_i1	dmso	control	1,4852049	3,36911625	5,65E-13	3,05E-11
TRINITY_DN12484_c0_g1_i1	dmso	control	1,48360759	4,72170363	8,29E-12	3,64E-10
TRINITY_DN38192_c0_g1_i1	dmso	control	1,48104157	4,24632847	1,62E-17	1,45E-15
TRINITY_DN22067_c0_g1_i1	dmso	control	1,48081733	1,60907774	1,80E-06	2,98E-05
TRINITY_DN6877_c0_g1_i1	dmso	control	1,48002626	2,82539379	4,32E-11	1,69E-09
TRINITY_DN23151_c0_g1_i1	dmso	control	1,47072029	-0,1469406	0,00064083	0,00512274
TRINITY_DN13300_c0_g1_i1	dmso	control	1,47047741	2,80758955	5,36E-09	1,46E-07
TRINITY_DN24400_c0_g1_i1	dmso	control	1,46877097	1,39293916	0,00010548	0,00109463
TRINITY_DN35071_c0_g1_i1	dmso	control	1,46790046	0,60945284	0,00013843	0,00138285
TRINITY_DN50209_c0_g1_i1	dmso	control	1,46768455	0,22714729	0,00133629	0,00944359
TRINITY_DN41736_c0_g1_i1	dmso	control	1,46668083	1,70255288	3,71E-07	7,16E-06
TRINITY_DN17930_c0_g1_i1	dmso	control	1,46560429	0,20030389	0,00060942	0,00489874
TRINITY_DN6394_c0_g1_i1	dmso	control	1,46466616	3,11125673	7,60E-11	2,85E-09
TRINITY_DN31951_c0_g1_i1	dmso	control	1,46402273	2,53716787	1,07E-09	3,33E-08
TRINITY_DN8557_c0_g1_i1	dmso	control	1,46322893	0,78120904	0,00043069	0,00364247

TRINITY_DN13187_c0_g1_i1	dmso	control	1,46297552	3,92376841	9,55E-16	7,08E-14
TRINITY_DN23093_c0_g1_i1	dmso	control	1,46094125	4,33841699	1,34E-16	1,10E-14
TRINITY_DN22968_c0_g1_i1	dmso	control	1,45536692	3,18525402	1,44E-10	5,17E-09
TRINITY_DN9091_c0_g1_i1	dmso	control	1,45525849	0,4554617	0,00080972	0,00622568
TRINITY_DN30628_c0_g1_i1	dmso	control	1,44856598	2,89955886	1,03E-10	3,85E-09
TRINITY_DN14980_c0_g1_i1	dmso	control	1,4445473	2,66505669	2,92E-09	8,38E-08
TRINITY_DN27008_c0_g1_i1	dmso	control	1,44209861	2,29782105	4,38E-07	8,34E-06
TRINITY_DN21756_c0_g1_i1	dmso	control	1,44012967	3,11246128	7,36E-08	1,67E-06
TRINITY_DN36818_c0_g1_i1	dmso	control	1,43808988	2,01814065	8,23E-05	0,0008821
TRINITY_DN11040_c0_g1_i1	dmso	control	1,43786132	4,93067099	7,26E-14	4,35E-12
TRINITY_DN11101_c0_g1_i1	dmso	control	1,43779737	5,81830283	2,44E-22	3,45E-20
TRINITY_DN24119_c0_g1_i1	dmso	control	1,42910903	0,52092931	0,00061777	0,00495276
TRINITY_DN19990_c0_g1_i1	dmso	control	1,42805591	7,35393137	1,12E-28	2,83E-26
TRINITY_DN22423_c0_g1_i1	dmso	control	1,4257083	1,43653645	1,17E-05	0,00016206
TRINITY_DN12548_c0_g1_i1	dmso	control	1,42408304	2,97883651	6,86E-11	2,60E-09
TRINITY_DN21137_c0_g1_i1	dmso	control	1,42298979	2,08514356	1,50E-07	3,19E-06
TRINITY_DN27712_c0_g1_i1	dmso	control	1,42254134	-0,3126184	0,00281329	0,01717581
TRINITY_DN33643_c0_g1_i1	dmso	control	1,42076888	1,45750282	3,24E-06	5,01E-05
TRINITY_DN2880_c0_g1_i1	dmso	control	1,41843292	5,793832	3,29E-24	5,59E-22
TRINITY_DN13879_c0_g1_i1	dmso	control	1,4171526	1,40594469	1,21E-05	0,00016659
TRINITY_DN28225_c0_g1_i1	dmso	control	1,41668991	0,17485555	0,00058762	0,0047474
TRINITY_DN34421_c0_g1_i1	dmso	control	1,41530359	1,41735047	5,11E-06	7,62E-05
TRINITY_DN9439_c0_g1_i1	dmso	control	1,40947565	1,84910032	5,38E-06	7,96E-05
TRINITY_DN22182_c0_g1_i1	dmso	control	1,40927895	1,99585307	1,54E-07	3,28E-06
TRINITY_DN24909_c0_g1_i1	dmso	control	1,40472051	0,59975009	0,00031914	0,00283434
TRINITY_DN2075_c0_g1_i1	dmso	control	1,40077849	2,10377441	8,94E-07	1,58E-05
TRINITY_DN2124_c0_g1_i1	dmso	control	1,39426553	3,80185659	4,14E-07	7,94E-06
TRINITY_DN26440_c0_g1_i1	dmso	control	1,39424167	0,98862496	3,51E-05	0,00042041
TRINITY_DN1464_c0_g1_i1	dmso	control	1,39412626	7,83109891	4,22E-34	1,56E-31
TRINITY_DN2377_c0_g1_i1	dmso	control	1,39255834	5,55210156	1,47E-11	6,24E-10
TRINITY_DN3951_c0_g1_i1	dmso	control	1,38903565	3,45749814	6,36E-12	2,83E-10

TRINITY_DN36717_c0_g1_i1	dmso	control	1,38730931	1,71845512	5,24E-06	7,79E-05
TRINITY_DN33961_c0_g1_i1	dmso	control	1,38654759	1,07085117	3,28E-05	0,00039452
TRINITY_DN17520_c0_g1_i1	dmso	control	1,38500342	1,38857177	3,60E-05	0,00042971
TRINITY_DN4732_c0_g1_i1	dmso	control	1,38435141	3,60438525	9,65E-11	3,60E-09
TRINITY_DN14845_c0_g1_i1	dmso	control	1,38096028	3,90575046	9,36E-13	4,93E-11
TRINITY_DN2708_c0_g1_i1	dmso	control	1,38059049	2,67949163	4,51E-08	1,06E-06
TRINITY_DN24832_c0_g1_i1	dmso	control	1,37966261	2,55428018	1,84E-07	3,85E-06
TRINITY_DN37404_c0_g1_i1	dmso	control	1,37953848	0,09349816	0,00079408	0,00612481
TRINITY_DN11954_c0_g1_i1	dmso	control	1,37943397	2,39919197	3,25E-06	5,03E-05
TRINITY_DN15312_c0_g1_i1	dmso	control	1,37825997	0,88550207	0,00012909	0,00129945
TRINITY_DN4632_c0_g1_i1	dmso	control	1,37809855	5,72023878	8,02E-17	6,77E-15
TRINITY_DN37431_c0_g1_i1	dmso	control	1,371032	0,68771913	0,00016875	0,00163162
TRINITY_DN3113_c0_g1_i1	dmso	control	1,36856064	5,25756184	2,63E-18	2,51E-16
TRINITY_DN3128_c0_g1_i1	dmso	control	1,36802697	4,01059329	7,95E-12	3,51E-10
TRINITY_DN7258_c0_g1_i1	dmso	control	1,36571013	2,62628776	1,10E-06	1,90E-05
TRINITY_DN9677_c0_g1_i1	dmso	control	1,36440765	0,8460415	0,0013506	0,00952612
TRINITY_DN10583_c0_g1_i1	dmso	control	1,3625894	1,11375022	0,00025285	0,00231019
TRINITY_DN16695_c0_g1_i1	dmso	control	1,36206863	1,85080239	1,07E-05	0,00014921
TRINITY_DN9977_c0_g1_i1	dmso	control	1,36193629	0,68819122	0,00012711	0,00128218
TRINITY_DN9518_c0_g1_i1	dmso	control	1,35905473	1,85219038	4,77E-06	7,15E-05
TRINITY_DN6124_c0_g1_i1	dmso	control	1,3578855	5,28990741	2,06E-20	2,55E-18
TRINITY_DN3834_c0_g1_i1	dmso	control	1,3536886	0,91369479	0,00011445	0,00117414
TRINITY_DN19407_c0_g1_i1	dmso	control	1,35206718	4,84676142	2,12E-10	7,38E-09
TRINITY_DN7953_c0_g1_i1	dmso	control	1,34877578	7,18740381	1,01E-27	2,36E-25
TRINITY_DN22523_c0_g1_i1	dmso	control	1,34754292	0,16867917	0,00144136	0,01004173
TRINITY_DN11220_c0_g1_i1	dmso	control	1,34332774	4,39347556	1,06E-12	5,49E-11
TRINITY_DN14304_c0_g1_i1	dmso	control	1,3431381	2,62880465	1,73E-06	2,88E-05
TRINITY_DN38474_c0_g1_i1	dmso	control	1,33949125	0,08760238	0,00177534	0,01190116
TRINITY_DN8965_c0_g1_i1	dmso	control	1,33805389	2,02730063	3,26E-05	0,000393
TRINITY_DN37494_c0_g1_i1	dmso	control	1,33661186	2,48100554	2,10E-07	4,30E-06
TRINITY_DN9427_c0_g1_i1	dmso	control	1,33328751	4,54435553	9,10E-17	7,60E-15

TRINITY_DN22536_c0_g1_i1	dmso	control	1,3330922	1,1607189	6,95E-05	0,00076482
TRINITY_DN38770_c0_g1_i1	dmso	control	1,33172524	-0,1560892	0,00528724	0,02819278
TRINITY_DN24868_c0_g1_i1	dmso	control	1,32770663	0,02197281	0,00243123	0,01528244
TRINITY_DN6242_c0_g1_i1	dmso	control	1,32455009	2,78402398	8,58E-09	2,28E-07
TRINITY_DN39195_c0_g1_i1	dmso	control	1,32243026	3,2084293	6,53E-10	2,11E-08
TRINITY_DN1626_c0_g1_i1	dmso	control	1,32101736	4,54407278	5,43E-11	2,08E-09
TRINITY_DN6192_c0_g1_i1	dmso	control	1,31909623	3,288749	4,69E-07	8,85E-06
TRINITY_DN24187_c0_g1_i1	dmso	control	1,3181046	1,46630288	5,00E-05	0,00057382
TRINITY_DN2145_c0_g1_i1	dmso	control	1,31264644	4,32245044	1,14E-10	4,21E-09
TRINITY_DN30926_c0_g1_i1	dmso	control	1,31149983	-0,0543705	0,00209162	0,01354257
TRINITY_DN14291_c0_g1_i1	dmso	control	1,3113291	2,70844866	3,20E-08	7,64E-07
TRINITY_DN13771_c0_g1_i1	dmso	control	1,31076754	3,32833282	0,00019872	0,00187497
TRINITY_DN19276_c0_g1_i1	dmso	control	1,30846178	4,16736622	2,66E-09	7,77E-08
TRINITY_DN11557_c0_g1_i1	dmso	control	1,30451308	4,79084916	2,06E-13	1,17E-11
TRINITY_DN22545_c0_g1_i1	dmso	control	1,30432993	2,10396326	6,68E-07	1,22E-05
TRINITY_DN20945_c0_g1_i1	dmso	control	1,30319247	6,51717137	5,33E-18	4,96E-16
TRINITY_DN41867_c0_g1_i1	dmso	control	1,3012752	-0,1059447	0,00219382	0,01408433
TRINITY_DN1222_c0_g1_i1	dmso	control	1,29780332	3,46980845	2,06E-07	4,23E-06
TRINITY_DN37285_c0_g1_i1	dmso	control	1,29713746	1,77406055	5,35E-05	0,0006089
TRINITY_DN29949_c0_g1_i1	dmso	control	1,29665065	0,97932073	0,00010429	0,00108381
TRINITY_DN13936_c0_g1_i1	dmso	control	1,29633461	2,06063219	1,97E-06	3,21E-05
TRINITY_DN43658_c0_g1_i1	dmso	control	1,2957035	0,17704414	0,00107427	0,00788908
TRINITY_DN38490_c0_g1_i1	dmso	control	1,29555967	0,28026954	0,00274481	0,01682887
TRINITY_DN43053_c0_g1_i1	dmso	control	1,294662	2,02987384	1,11E-06	1,91E-05
TRINITY_DN16703_c0_g1_i1	dmso	control	1,29211771	0,00672405	0,00493784	0,02671428
TRINITY_DN14152_c0_g1_i1	dmso	control	1,29164726	0,77125915	0,00908617	0,04299039
TRINITY_DN23822_c0_g1_i1	dmso	control	1,29115574	4,0258372	5,86E-12	2,61E-10
TRINITY_DN16834_c0_g1_i1	dmso	control	1,2908854	1,89303035	3,05E-06	4,76E-05
TRINITY_DN17643_c0_g1_i1	dmso	control	1,28979246	0,03759095	0,00192552	0,01267739
TRINITY_DN10402_c0_g1_i1	dmso	control	1,28868996	1,33946149	2,84E-05	0,00034826
TRINITY_DN22504_c0_g1_i1	dmso	control	1,28619644	1,46923203	1,86E-05	0,00024118

TRINITY_DN12657_c0_g1_i1	dmso	control	1,28490372	2,15675476	2,81E-06	4,43E-05
TRINITY_DN3332_c0_g1_i1	dmso	control	1,28486396	1,95386475	0,02560529	0,09520421
TRINITY_DN12506_c0_g1_i1	dmso	control	1,28437511	6,13548244	7,71E-26	1,45E-23
TRINITY_DN34038_c0_g1_i1	dmso	control	1,28385686	3,07714701	4,93E-09	1,35E-07
TRINITY_DN22855_c0_g1_i1	dmso	control	1,28023034	5,48800238	2,10E-18	2,03E-16
TRINITY_DN11299_c0_g1_i1	dmso	control	1,27958567	6,28173925	1,42E-21	1,89E-19
TRINITY_DN16865_c0_g1_i1	dmso	control	1,27618606	2,43922598	3,22E-06	4,99E-05
TRINITY_DN22251_c0_g1_i1	dmso	control	1,26923429	2,6583037	2,69E-07	5,37E-06
TRINITY_DN27454_c0_g1_i1	dmso	control	1,26863784	1,59182866	0,00330424	0,01956407
TRINITY_DN33083_c0_g1_i1	dmso	control	1,26560261	4,52007673	2,24E-14	1,43E-12
TRINITY_DN1561_c0_g1_i1	dmso	control	1,26290505	6,68725895	1,54E-24	2,71E-22
TRINITY_DN26235_c0_g1_i1	dmso	control	1,26053438	1,98213996	2,11E-05	0,00026863
TRINITY_DN1488_c0_g1_i1	dmso	control	1,25921757	3,4755699	4,62E-08	1,08E-06
TRINITY_DN22210_c0_g1_i1	dmso	control	1,25812067	-0,0760549	0,00370416	0,02137006
TRINITY_DN20804_c0_g1_i1	dmso	control	1,25768836	1,99594165	3,08E-06	4,79E-05
TRINITY_DN35600_c0_g1_i1	dmso	control	1,25453902	0,12628468	0,00386733	0,02208922
TRINITY_DN7974_c0_g1_i1	dmso	control	1,25227373	1,98241185	4,37E-06	6,59E-05
TRINITY_DN4445_c0_g1_i1	dmso	control	1,25198441	0,34098578	0,00403495	0,02283066
TRINITY_DN39130_c0_g1_i1	dmso	control	1,25119867	2,23637938	3,00E-06	4,68E-05
TRINITY_DN9281_c0_g1_i1	dmso	control	1,24999611	2,89002667	5,34E-06	7,93E-05
TRINITY_DN17039_c0_g1_i1	dmso	control	1,24945002	2,3839159	9,38E-06	0,00013159
TRINITY_DN6598_c0_g1_i1	dmso	control	1,24940888	5,75360791	2,31E-19	2,51E-17
TRINITY_DN24368_c0_g1_i1	dmso	control	1,24708608	0,46879299	0,01290975	0,05685408
TRINITY_DN21906_c0_g1_i1	dmso	control	1,24655846	1,47963885	2,68E-05	0,00033159
TRINITY_DN33590_c0_g1_i1	dmso	control	1,24020617	3,64097002	3,10E-10	1,07E-08
TRINITY_DN7278_c0_g1_i1	dmso	control	1,24015096	3,20607477	2,87E-08	6,92E-07
TRINITY_DN4228_c0_g1_i1	dmso	control	1,2371869	3,56033898	1,23E-06	2,11E-05
TRINITY_DN15493_c0_g1_i1	dmso	control	1,23636632	2,66782358	3,34E-06	5,14E-05
TRINITY_DN12089_c0_g1_i1	dmso	control	1,23606312	4,43093322	1,01E-12	5,27E-11
TRINITY_DN301_c0_g1_i1	dmso	control	1,23588242	6,06368685	1,17E-23	1,89E-21
TRINITY_DN37330_c0_g1_i1	dmso	control	1,23488857	0,56333504	0,00084297	0,0064325

TRINITY_DN15860_c0_g1_i1	dmso	control	1,22958372	3,87113795	1,96E-08	4,89E-07
TRINITY_DN12609_c0_g1_i1	dmso	control	1,22930583	3,22186724	1,16E-08	3,04E-07
TRINITY_DN24140_c0_g1_i1	dmso	control	1,22916476	0,57200482	0,00169971	0,01148378
TRINITY_DN3501_c0_g1_i1	dmso	control	1,22804401	3,75676723	2,36E-10	8,18E-09
TRINITY_DN13736_c0_g1_i1	dmso	control	1,22310113	4,13405237	3,17E-12	1,48E-10
TRINITY_DN6932_c0_g1_i1	dmso	control	1,22265945	3,7978064	1,24E-10	4,53E-09
TRINITY_DN13598_c0_g1_i1	dmso	control	1,22061255	3,67124066	3,94E-10	1,31E-08
TRINITY_DN15255_c0_g1_i1	dmso	control	1,21903297	0,96989252	0,01154317	0,05196279
TRINITY_DN1023_c0_g1_i1	dmso	control	1,21806911	5,16926161	2,05E-14	1,32E-12
TRINITY_DN1448_c0_g1_i1	dmso	control	1,2173007	4,91001962	5,10E-12	2,29E-10
TRINITY_DN672_c0_g1_i1	dmso	control	1,21596196	5,00981372	1,42E-12	7,18E-11
TRINITY_DN32300_c0_g1_i1	dmso	control	1,21487641	5,87437222	1,36E-21	1,81E-19
TRINITY_DN3742_c0_g1_i1	dmso	control	1,21442616	2,14692783	0,00444032	0,02464096
TRINITY_DN36321_c0_g1_i1	dmso	control	1,21390851	1,47625129	0,00019911	0,00187737
TRINITY_DN15157_c0_g1_i1	dmso	control	1,2135822	4,3110857	2,93E-11	1,18E-09
TRINITY_DN8696_c0_g1_i1	dmso	control	1,21080522	1,37426869	0,00098636	0,00729954
TRINITY_DN27622_c0_g1_i1	dmso	control	1,20846994	2,24408714	3,83E-05	0,00045432
TRINITY_DN24991_c0_g1_i1	dmso	control	1,20600572	0,52858659	0,00162219	0,01105831
TRINITY_DN16291_c0_g1_i1	dmso	control	1,20572585	0,83020623	0,00047371	0,0039527
TRINITY_DN12305_c0_g1_i1	dmso	control	1,20327665	3,1991731	1,64E-08	4,15E-07
TRINITY_DN7225_c0_g1_i1	dmso	control	1,20172875	3,22664872	5,28E-09	1,44E-07
TRINITY_DN7866_c0_g1_i1	dmso	control	1,20004127	0,70906511	0,00075925	0,0058996
TRINITY_DN6452_c0_g1_i1	dmso	control	1,19771746	2,36734998	0,00015267	0,0014982
TRINITY_DN15667_c0_g1_i1	dmso	control	1,19709448	1,58828139	7,53E-05	0,00082012
TRINITY_DN7932_c0_g1_i1	dmso	control	1,19677937	3,81263353	7,84E-08	1,76E-06
TRINITY_DN7295_c0_g1_i1	dmso	control	1,19653476	3,7425722	2,06E-09	6,16E-08
TRINITY_DN16069_c0_g1_i1	dmso	control	1,19610735	4,37936661	1,06E-10	3,93E-09
TRINITY_DN25576_c0_g1_i1	dmso	control	1,19423081	1,37597403	0,0002725	0,0024648
TRINITY_DN11458_c0_g1_i1	dmso	control	1,19397456	7,41210349	2,57E-17	2,26E-15
TRINITY_DN7426_c0_g1_i1	dmso	control	1,19310412	1,81453691	1,59E-05	0,00020933
TRINITY_DN8691_c0_g1_i1	dmso	control	1,18823559	7,53943086	2,97E-24	5,11E-22

TRINITY_DN12148_c0_g1_i1	dmso	control	1,18700226	5,20259464	8,71E-17	7,32E-15
TRINITY_DN7034_c0_g1_i1	dmso	control	1,18652436	3,99971101	6,87E-11	2,60E-09
TRINITY_DN29208_c0_g1_i1	dmso	control	1,18537705	2,01710263	1,22E-05	0,00016735
TRINITY_DN35637_c0_g1_i1	dmso	control	1,18400714	1,6481518	3,73E-05	0,00044395
TRINITY_DN13656_c0_g1_i1	dmso	control	1,18397829	1,01039773	0,00050271	0,00415427
TRINITY_DN41557_c0_g1_i1	dmso	control	1,18351947	-0,0590264	0,00917353	0,04333277
TRINITY_DN10200_c0_g1_i1	dmso	control	1,182574	0,91521808	0,00072664	0,00567979
TRINITY_DN33333_c0_g1_i1	dmso	control	1,17782856	1,88714988	9,64E-05	0,00101522
TRINITY_DN38912_c0_g1_i1	dmso	control	1,17345448	2,42691132	1,04E-05	0,00014463
TRINITY_DN20885_c0_g1_i1	dmso	control	1,17158035	0,85970509	0,0006584	0,00523714
TRINITY_DN6016_c0_g1_i1	dmso	control	1,1714354	5,28586607	3,76E-15	2,61E-13
TRINITY_DN3085_c0_g1_i1	dmso	control	1,17068704	4,0004867	1,19E-09	3,65E-08
TRINITY_DN22142_c0_g1_i1	dmso	control	1,16962781	1,28589211	0,00027201	0,00246194
TRINITY_DN23366_c0_g1_i1	dmso	control	1,16914282	3,62495381	1,40E-09	4,26E-08
TRINITY_DN45586_c0_g1_i1	dmso	control	1,16847686	0,01534533	0,00593785	0,0308533
TRINITY_DN16415_c0_g1_i1	dmso	control	1,16669463	1,72283735	0,00287757	0,01750154
TRINITY_DN25057_c0_g1_i1	dmso	control	1,16235375	0,47679837	0,00165195	0,01120301
TRINITY_DN11908_c0_g1_i1	dmso	control	1,16213199	5,08634748	5,92E-14	3,59E-12
TRINITY_DN3848_c0_g1_i1	dmso	control	1,16187647	3,18135849	3,05E-07	6,01E-06
TRINITY_DN33066_c0_g1_i1	dmso	control	1,16141933	3,03764749	4,68E-08	1,09E-06
TRINITY_DN39790_c0_g1_i1	dmso	control	1,1606411	0,94830444	0,0008642	0,00657018
TRINITY_DN31404_c0_g1_i1	dmso	control	1,16060164	0,49770026	0,01378248	0,05948254
TRINITY_DN2223_c0_g1_i1	dmso	control	1,15830414	3,8439654	7,02E-10	2,25E-08
TRINITY_DN11330_c0_g1_i1	dmso	control	1,15791848	4,38479667	0,00272342	0,01671189
TRINITY_DN22872_c0_g1_i1	dmso	control	1,15693569	4,03102876	7,54E-09	2,02E-07
TRINITY_DN14040_c0_g1_i1	dmso	control	1,15291626	2,96256247	8,70E-07	1,54E-05
TRINITY_DN42039_c0_g1_i1	dmso	control	1,15276661	1,85443057	0,00017976	0,00172216
TRINITY_DN8305_c0_g1_i1	dmso	control	1,15114711	3,63976793	2,75E-07	5,47E-06
TRINITY_DN1629_c0_g1_i1	dmso	control	1,15022752	2,90646215	5,71E-07	1,06E-05
TRINITY_DN1731_c0_g1_i1	dmso	control	1,14812959	3,87383776	9,85E-10	3,08E-08
TRINITY_DN2811_c0_g1_i1	dmso	control	1,14588311	4,94091341	1,18E-13	6,83E-12

TRINITY_DN22339_c0_g1_i1	dmso	control	1,144633	2,25331376	4,36E-05	0,00050828
TRINITY_DN6261_c0_g1_i1	dmso	control	1,14422178	6,12915758	4,66E-17	4,03E-15
TRINITY_DN43481_c0_g1_i1	dmso	control	1,14349575	0,200966	0,00434375	0,02417938
TRINITY_DN20318_c0_g1_i1	dmso	control	1,14277564	0,18916967	0,00397569	0,02258311
TRINITY_DN2380_c0_g1_i1	dmso	control	1,14183821	2,94177956	1,56E-07	3,31E-06
TRINITY_DN3691_c0_g1_i1	dmso	control	1,14037529	1,49513342	0,00033974	0,00298876
TRINITY_DN17885_c0_g1_i1	dmso	control	1,14018124	1,37143159	0,00028157	0,00253953
TRINITY_DN11797_c0_g1_i1	dmso	control	1,13918092	3,1868962	8,25E-07	1,47E-05
TRINITY_DN22533_c0_g1_i1	dmso	control	1,13767299	0,20606086	0,00770057	0,03782566
TRINITY_DN9315_c0_g1_i1	dmso	control	1,13721663	2,21284841	0,00254696	0,01585127
TRINITY_DN3369_c0_g1_i1	dmso	control	1,13628085	3,89483692	1,72E-09	5,17E-08
TRINITY_DN27699_c0_g1_i1	dmso	control	1,13613102	2,62668931	1,06E-06	1,84E-05
TRINITY_DN43623_c0_g1_i1	dmso	control	1,13604654	0,75473776	0,00119454	0,00860174
TRINITY_DN13557_c0_g1_i1	dmso	control	1,13581523	4,92117233	3,62E-06	5,53E-05
TRINITY_DN14442_c0_g1_i1	dmso	control	1,13555192	0,6131885	0,00193942	0,01273456
TRINITY_DN67742_c0_g1_i1	dmso	control	1,13458695	3,04474466	0,0133411	0,05818607
TRINITY_DN36286_c0_g1_i1	dmso	control	1,13057885	1,28134887	0,00246164	0,01543224
TRINITY_DN20431_c0_g1_i1	dmso	control	1,13017965	1,94617449	0,00011762	0,00120326
TRINITY_DN2874_c0_g1_i1	dmso	control	1,13016458	4,60112313	3,67E-12	1,70E-10
TRINITY_DN22701_c0_g1_i1	dmso	control	1,12859273	2,17224581	0,00094195	0,00701726
TRINITY_DN21988_c0_g1_i1	dmso	control	1,12817729	1,51416986	0,00160353	0,01096404
TRINITY_DN31699_c0_g1_i1	dmso	control	1,12708534	-0,0955838	0,01153012	0,05193406
TRINITY_DN19163_c0_g1_i1	dmso	control	1,12620401	4,11811061	1,48E-09	4,49E-08
TRINITY_DN13658_c0_g1_i1	dmso	control	1,12587757	1,12413227	0,00214938	0,013873
TRINITY_DN13823_c0_g1_i1	dmso	control	1,12375658	3,06408226	6,48E-07	1,18E-05
TRINITY_DN6325_c0_g1_i1	dmso	control	1,12366628	3,74955963	8,28E-09	2,21E-07
TRINITY_DN33476_c0_g1_i1	dmso	control	1,12351964	2,31756597	0,00030349	0,00271113
TRINITY_DN20913_c0_g1_i1	dmso	control	1,12196121	1,70610902	0,00014423	0,00142995
TRINITY_DN64957_c0_g1_i1	dmso	control	1,1218931	0,65313103	0,00243004	0,01528162
TRINITY_DN579_c0_g1_i1	dmso	control	1,12126336	5,48885164	1,26E-15	9,21E-14
TRINITY_DN12445_c0_g1_i1	dmso	control	1,11910872	3,13602739	0,00040311	0,0034406

TRINITY_DN30320_c0_g1_i1	dmso	control	1,11818326	0,01332028	0,0063299	0,03242393
TRINITY_DN4515_c0_g1_i1	dmso	control	1,11681032	3,15267549	1,45E-06	2,45E-05
TRINITY_DN44037_c0_g1_i1	dmso	control	1,11524812	3,59835882	6,81E-09	1,84E-07
TRINITY_DN12955_c0_g1_i1	dmso	control	1,11494948	4,81080953	5,25E-09	1,43E-07
TRINITY_DN32582_c0_g1_i1	dmso	control	1,11334678	4,25395602	3,18E-06	4,93E-05
TRINITY_DN28209_c0_g1_i1	dmso	control	1,11184186	0,55496805	0,00326966	0,01943633
TRINITY_DN42307_c0_g1_i1	dmso	control	1,11050758	0,50626171	0,00368909	0,02131557
TRINITY_DN4119_c0_g1_i1	dmso	control	1,11047257	1,77599648	0,01638233	0,06786427
TRINITY_DN25407_c0_g1_i1	dmso	control	1,10964759	1,94757628	9,93E-05	0,00103896
TRINITY_DN14260_c0_g1_i1	dmso	control	1,1090142	6,21235787	6,73E-16	5,23E-14
TRINITY_DN27001_c0_g1_i1	dmso	control	1,10866413	2,40490643	0,0004698	0,00393423
TRINITY_DN28243_c0_g1_i1	dmso	control	1,10792069	1,33233235	0,01412922	0,06050892
TRINITY_DN18512_c0_g1_i1	dmso	control	1,10685886	1,96021364	4,60E-05	0,00053346
TRINITY_DN46976_c0_g1_i1	dmso	control	1,10665106	1,63422481	0,00012646	0,0012774
TRINITY_DN39967_c0_g1_i1	dmso	control	1,10504793	-0,1322636	0,01057464	0,04855392
TRINITY_DN12458_c0_g1_i1	dmso	control	1,10490675	4,5256935	1,35E-06	2,30E-05
TRINITY_DN15328_c0_g1_i1	dmso	control	1,10489221	2,273189	0,00057548	0,00467277
TRINITY_DN33295_c0_g1_i1	dmso	control	1,10386049	3,9873287	4,51E-06	6,78E-05
TRINITY_DN3659_c0_g1_i1	dmso	control	1,1028724	3,82645925	1,35E-07	2,92E-06
TRINITY_DN75509_c0_g1_i1	dmso	control	1,10274571	0,84037453	0,00845032	0,04076984
TRINITY_DN7501_c0_g1_i1	dmso	control	1,10125571	3,57074792	2,05E-07	4,22E-06
TRINITY_DN12598_c0_g1_i1	dmso	control	1,10053048	3,87605454	1,27E-08	3,31E-07
TRINITY_DN31350_c0_g1_i1	dmso	control	1,1002473	0,66369985	0,00499189	0,02690593
TRINITY_DN23873_c0_g1_i1	dmso	control	1,09744126	0,1371429	0,01508458	0,06360583
TRINITY_DN18922_c0_g1_i1	dmso	control	1,09675676	4,94560493	1,21E-13	6,99E-12
TRINITY_DN9970_c0_g1_i1	dmso	control	1,09651583	0,86532279	0,00675153	0,0340523
TRINITY_DN32699_c0_g1_i1	dmso	control	1,09637303	2,19592557	0,00103946	0,00766069
TRINITY_DN12652_c0_g1_i1	dmso	control	1,09551637	2,6035688	0,00086352	0,0065685
TRINITY_DN8053_c0_g1_i1	dmso	control	1,09523887	2,80162497	9,30E-07	1,63E-05
TRINITY_DN1556_c0_g1_i1	dmso	control	1,09418709	4,92887852	1,64E-06	2,75E-05
TRINITY_DN35573_c0_g1_i1	dmso	control	1,09355661	1,3289317	0,00075634	0,00588198

TRINITY_DN8662_c0_g1_i1	dmso	control	1,09279768	1,00070325	0,00681712	0,03431362
TRINITY_DN14984_c0_g1_i1	dmso	control	1,09266387	1,39763653	0,00046655	0,00391137
TRINITY_DN22166_c0_g1_i1	dmso	control	1,09247022	0,40943153	0,00817948	0,0397285
TRINITY_DN27757_c0_g1_i1	dmso	control	1,09135542	1,49638253	0,00830583	0,04019356
TRINITY_DN9581_c0_g1_i1	dmso	control	1,09059052	3,15695702	4,54E-07	8,61E-06
TRINITY_DN7451_c0_g1_i1	dmso	control	1,0883016	1,60492652	0,01609349	0,06701369
TRINITY_DN30980_c0_g1_i1	dmso	control	1,08819207	0,64884485	0,00596028	0,03093647
TRINITY_DN4951_c0_g1_i1	dmso	control	1,08800327	6,02508774	1,08E-16	8,96E-15
TRINITY_DN19301_c0_g1_i1	dmso	control	1,08646999	2,8490003	3,20E-06	4,96E-05
TRINITY_DN20482_c0_g1_i1	dmso	control	1,08630809	3,17243365	4,31E-07	8,23E-06
TRINITY_DN17907_c0_g1_i1	dmso	control	1,08602046	4,51259543	6,63E-11	2,52E-09
TRINITY_DN2099_c0_g1_i1	dmso	control	1,08519349	3,28799806	2,43E-07	4,89E-06
TRINITY_DN13052_c0_g1_i1	dmso	control	1,08442554	5,28052251	1,75E-10	6,20E-09
TRINITY_DN3220_c0_g1_i1	dmso	control	1,08413771	1,56272849	0,00019618	0,00185334
TRINITY_DN12537_c0_g1_i1	dmso	control	1,08339806	2,83181599	2,11E-05	0,00026819
TRINITY_DN15431_c0_g1_i1	dmso	control	1,08331493	4,25343925	4,06E-08	9,57E-07
TRINITY_DN54254_c0_g1_i1	dmso	control	1,0823828	-0,1692319	0,02119994	0,08276571
TRINITY_DN20016_c0_g1_i1	dmso	control	1,08089234	3,9356643	4,65E-06	6,97E-05
TRINITY_DN7382_c0_g1_i1	dmso	control	1,07802949	4,60256285	1,37E-11	5,86E-10
TRINITY_DN63568_c0_g1_i1	dmso	control	1,07662594	2,57506491	1,47E-05	0,00019641
TRINITY_DN23042_c0_g1_i1	dmso	control	1,07547603	2,36009967	1,68E-05	0,00021944
TRINITY_DN2330_c0_g1_i1	dmso	control	1,07483076	2,99221056	2,61E-05	0,0003233
TRINITY_DN3276_c0_g1_i1	dmso	control	1,07325335	1,53560116	0,00551435	0,0291562
TRINITY_DN20682_c0_g1_i1	dmso	control	1,07058419	2,88115545	0,00025647	0,0023374
TRINITY_DN33920_c0_g1_i1	dmso	control	1,0704375	0,50890898	0,00886124	0,04220265
TRINITY_DN14131_c0_g1_i1	dmso	control	1,06911721	4,51053859	6,91E-11	2,61E-09
TRINITY_DN4704_c0_g1_i1	dmso	control	1,0689154	9,53510274	2,15E-22	3,08E-20
TRINITY_DN15269_c0_g1_i1	dmso	control	1,06847538	0,51899906	0,00606005	0,03139794
TRINITY_DN32944_c0_g1_i1	dmso	control	1,06820714	2,70892045	8,50E-06	0,00012055
TRINITY_DN10393_c0_g1_i1	dmso	control	1,06781725	0,49574734	0,00723605	0,03604301
TRINITY_DN1857_c0_g1_i1	dmso	control	1,06595653	4,55002531	5,02E-10	1,65E-08

TRINITY_DN33707_c0_g1_i1	dmso	control	1,06518149	0,86611831	0,0025451	0,01584652
TRINITY_DN4050_c0_g1_i1	dmso	control	1,0626783	1,24977602	0,00259982	0,01611774
TRINITY_DN4668_c0_g1_i1	dmso	control	1,06250729	3,60728444	3,66E-08	8,66E-07
TRINITY_DN21625_c0_g1_i1	dmso	control	1,05942888	1,99464066	0,00016528	0,00160238
TRINITY_DN10193_c0_g1_i1	dmso	control	1,05930777	0,52377243	0,00751477	0,03711141
TRINITY_DN975_c0_g1_i1	dmso	control	1,05923421	5,19925381	6,17E-14	3,73E-12
TRINITY_DN19986_c0_g1_i1	dmso	control	1,05689331	0,33946107	0,01448671	0,06156534
TRINITY_DN3997_c0_g1_i1	dmso	control	1,05526285	2,88745603	1,73E-06	2,89E-05
TRINITY_DN34766_c0_g1_i1	dmso	control	1,05519888	0,10350061	0,01827032	0,07392572
TRINITY_DN60488_c0_g1_i1	dmso	control	1,05467821	0,59227066	0,00875898	0,04183979
TRINITY_DN4344_c0_g1_i1	dmso	control	1,05430686	5,40628989	7,47E-14	4,46E-12
TRINITY_DN15986_c0_g1_i1	dmso	control	1,05425135	1,174937	0,00209372	0,01355009
TRINITY_DN8240_c0_g1_i1	dmso	control	1,05368039	1,63200439	0,00031965	0,0028362
TRINITY_DN14979_c0_g1_i1	dmso	control	1,05325915	2,06332693	0,00018522	0,00176721
TRINITY_DN28421_c0_g1_i1	dmso	control	1,05320477	0,26057114	0,00819017	0,03976706
TRINITY_DN13094_c0_g1_i1	dmso	control	1,05244524	4,39659207	6,35E-10	2,07E-08
TRINITY_DN597_c0_g1_i1	dmso	control	1,05236	5,13055793	2,00E-12	9,69E-11
TRINITY_DN22213_c0_g1_i1	dmso	control	1,05097132	8,26894385	4,88E-07	9,15E-06
TRINITY_DN33962_c0_g1_i1	dmso	control	1,0509397	1,16897629	0,00127865	0,0091346
TRINITY_DN26401_c0_g1_i1	dmso	control	1,04758219	0,43853779	0,01667427	0,06885643
TRINITY_DN10263_c0_g1_i1	dmso	control	1,04745476	5,37651901	4,83E-11	1,87E-09
TRINITY_DN15586_c0_g1_i1	dmso	control	1,04742071	0,64421928	0,01108719	0,05036305
TRINITY_DN6487_c0_g1_i1	dmso	control	1,04718182	2,81613541	9,81E-05	0,00102875
TRINITY_DN13593_c0_g1_i1	dmso	control	1,04649752	0,05071126	0,00939963	0,04424168
TRINITY_DN6550_c0_g1_i1	dmso	control	1,04623134	3,7356854	2,47E-08	6,04E-07
TRINITY_DN13575_c0_g1_i1	dmso	control	1,04531529	4,16683335	3,19E-08	7,62E-07
TRINITY_DN3360_c0_g1_i1	dmso	control	1,04444831	1,2653212	0,00164816	0,0111878
TRINITY_DN5107_c0_g1_i1	dmso	control	1,04361039	4,55001519	2,80E-08	6,76E-07
TRINITY_DN7619_c0_g1_i1	dmso	control	1,04314855	2,58662454	0,00547019	0,02897577
TRINITY_DN3845_c0_g1_i1	dmso	control	1,04291865	2,44285422	5,65E-05	0,00063981
TRINITY_DN14717_c0_g1_i1	dmso	control	1,04246858	2,22430514	0,00010756	0,0011122

TRINITY_DN80_c0_g1_i1	dmso	control	1,04239288	8,03159473	2,84E-20	3,45E-18
TRINITY_DN14159_c0_g1_i1	dmso	control	1,04087805	3,76635153	3,44E-07	6,68E-06
TRINITY_DN45102_c0_g1_i1	dmso	control	1,04081503	0,60227982	0,00731119	0,0363546
TRINITY_DN26979_c0_g1_i1	dmso	control	1,03957534	0,22703234	0,01587299	0,06629875
TRINITY_DN2659_c0_g1_i1	dmso	control	1,03883781	3,64677092	9,25E-07	1,63E-05
TRINITY_DN2932_c0_g1_i1	dmso	control	1,03872266	1,71527071	0,00072777	0,00568559
TRINITY_DN4878_c0_g1_i1	dmso	control	1,03818166	5,39077377	6,18E-11	2,36E-09
TRINITY_DN314_c0_g1_i1	dmso	control	1,03759908	9,15773828	6,21E-18	5,75E-16
TRINITY_DN9950_c0_g1_i1	dmso	control	1,03729255	2,22420167	0,00014609	0,00144637
TRINITY_DN278_c0_g1_i1	dmso	control	1,03566074	7,63776253	3,38E-20	4,07E-18
TRINITY_DN3163_c0_g1_i1	dmso	control	1,03414716	5,84294942	3,68E-16	2,92E-14
TRINITY_DN194_c0_g1_i1	dmso	control	1,03349552	8,35947133	1,44E-14	9,46E-13
TRINITY_DN22291_c0_g1_i1	dmso	control	1,03256019	1,38592498	0,0005553	0,00452923
TRINITY_DN23723_c0_g1_i1	dmso	control	1,0312777	0,58120095	0,00687279	0,03455531
TRINITY_DN16752_c0_g1_i1	dmso	control	1,03069802	5,14983291	4,11E-11	1,62E-09
TRINITY_DN43976_c0_g1_i1	dmso	control	1,03059712	1,07090389	0,00157777	0,01081248
TRINITY_DN38497_c0_g1_i1	dmso	control	1,03015577	1,60112479	0,00075502	0,00587937
TRINITY_DN62771_c0_g1_i1	dmso	control	1,02931898	0,04627803	0,02277545	0,08724248
TRINITY_DN7357_c0_g1_i1	dmso	control	1,02919675	4,99268132	2,01E-11	8,35E-10
TRINITY_DN6462_c0_g1_i1	dmso	control	1,02861146	3,93300151	9,54E-08	2,12E-06
TRINITY_DN26291_c0_g1_i1	dmso	control	1,02854102	1,45695087	0,0008418	0,00642697
TRINITY_DN22117_c0_g1_i1	dmso	control	1,02833941	2,4472444	0,00028326	0,00255096
TRINITY_DN14528_c0_g1_i1	dmso	control	1,02824714	1,88106035	0,00031191	0,00277602
TRINITY_DN4484_c0_g1_i1	dmso	control	1,02824543	0,42144812	0,0235995	0,08963838
TRINITY_DN26866_c0_g1_i1	dmso	control	1,02813483	0,92492525	0,00251582	0,01571843
TRINITY_DN36936_c0_g1_i1	dmso	control	1,02585964	0,60299831	0,00451171	0,02497955
TRINITY_DN11301_c0_g1_i1	dmso	control	1,02471377	5,11157815	4,80E-12	2,16E-10
TRINITY_DN9577_c0_g1_i1	dmso	control	1,0245579	3,01340613	2,12E-06	3,43E-05
TRINITY_DN25958_c0_g1_i1	dmso	control	1,02436335	2,82613155	5,23E-05	0,00059655
TRINITY_DN7906_c0_g1_i1	dmso	control	1,02385561	1,4096426	0,00058606	0,00474024
TRINITY_DN8430_c0_g1_i1	dmso	control	1,02311681	1,96836796	0,00147259	0,0102267

TRINITY_DN11385_c0_g1_i1	dmso	control	1,02290483	7,13785401	4,94E-13	2,67E-11
TRINITY_DN12564_c0_g1_i1	dmso	control	1,02247463	4,45927735	6,76E-10	2,17E-08
TRINITY_DN17236_c0_g1_i1	dmso	control	1,02201672	1,1069251	0,00150398	0,01039471
TRINITY_DN10400_c0_g1_i1	dmso	control	1,02057864	3,08987238	1,21E-06	2,09E-05
TRINITY_DN790_c0_g1_i1	dmso	control	1,01732892	5,28705931	2,74E-11	1,10E-09
TRINITY_DN33456_c0_g1_i1	dmso	control	1,01640501	1,06628774	0,002069	0,01344459
TRINITY_DN7976_c0_g1_i1	dmso	control	1,01574769	3,57351866	1,98E-07	4,10E-06
TRINITY_DN15702_c0_g1_i1	dmso	control	1,01506155	4,08619149	1,59E-05	0,00020967
TRINITY_DN13633_c0_g1_i1	dmso	control	1,01416552	0,68962991	0,00570011	0,0299411
TRINITY_DN9374_c0_g1_i1	dmso	control	1,01357051	7,19611543	9,41E-19	9,57E-17
TRINITY_DN2673_c0_g1_i1	dmso	control	1,01347106	3,05172904	6,68E-05	0,00073958
TRINITY_DN19593_c0_g1_i1	dmso	control	1,0133998	3,1485004	5,46E-06	8,06E-05
TRINITY_DN20080_c0_g1_i1	dmso	control	1,01275153	2,22687088	0,00011801	0,00120555
TRINITY_DN37826_c0_g1_i1	dmso	control	1,01141347	1,36855899	0,00244698	0,0153681
TRINITY_DN9311_c0_g1_i1	dmso	control	1,01010206	1,05377646	0,00519823	0,02775914
TRINITY_DN1769_c0_g1_i1	dmso	control	1,01002925	5,04245281	9,55E-12	4,17E-10
TRINITY_DN40642_c0_g1_i1	dmso	control	1,00967317	0,25577274	0,00937357	0,04413338
TRINITY_DN35771_c0_g1_i1	dmso	control	1,00864279	0,20945686	0,01026423	0,04739987
TRINITY_DN7849_c0_g1_i1	dmso	control	1,00751481	4,22903274	6,91E-09	1,86E-07
TRINITY_DN12329_c0_g1_i1	dmso	control	1,00671381	1,56064605	0,00047394	0,0039527
TRINITY_DN13559_c0_g1_i1	dmso	control	1,00611269	4,09959371	3,02E-07	5,96E-06
TRINITY_DN7979_c0_g1_i1	dmso	control	1,00489531	2,80560314	1,23E-05	0,0001693
TRINITY_DN20281_c0_g1_i1	dmso	control	1,00463664	2,20004389	0,00040712	0,00347154
TRINITY_DN22465_c0_g1_i1	dmso	control	1,00437491	2,64909789	0,00016131	0,00157444
TRINITY_DN295_c0_g1_i1	dmso	control	0,99936603	6,07515769	9,46E-10	2,98E-08
TRINITY_DN2898_c0_g1_i1	dmso	control	0,99885207	1,45360445	0,00114507	0,00829931
TRINITY_DN17905_c0_g1_i1	dmso	control	0,99871633	1,74662096	0,00062249	0,00498714
TRINITY_DN21570_c0_g1_i1	dmso	control	0,99756361	0,60484015	0,00819529	0,03977853
TRINITY_DN14078_c0_g1_i1	dmso	control	0,99722162	4,25190933	1,15E-08	3,04E-07
TRINITY_DN10004_c0_g1_i1	dmso	control	0,9957979	3,0012857	1,61E-05	0,00021162
TRINITY_DN27635_c0_g1_i1	dmso	control	0,99535321	2,23304078	9,97E-05	0,00104159

TRINITY_DN22165_c0_g1_i1	dmso	control	0,99481323	3,38218802	9,73E-07	1,70E-05
TRINITY_DN23041_c0_g1_i1	dmso	control	0,99432609	0,73640151	0,01271051	0,0562338
TRINITY_DN19671_c0_g1_i1	dmso	control	0,9937614	4,87949389	1,12E-10	4,12E-09
TRINITY_DN12286_c0_g1_i1	dmso	control	0,99360802	2,72833107	1,93E-05	0,00024828
TRINITY_DN14311_c0_g1_i1	dmso	control	0,99271247	2,68534789	8,20E-05	0,00088028
TRINITY_DN40665_c0_g1_i1	dmso	control	0,99157016	0,18042926	0,01440481	0,06130744
TRINITY_DN4754_c0_g1_i1	dmso	control	0,98901114	5,23817514	6,46E-10	2,09E-08
TRINITY_DN3304_c0_g1_i1	dmso	control	0,98686728	4,21364759	2,29E-08	5,64E-07
TRINITY_DN19794_c0_g1_i1	dmso	control	0,9866729	3,03107648	2,17E-05	0,00027455
TRINITY_DN12520_c0_g1_i1	dmso	control	0,986473	1,47330385	0,00303974	0,01831069
TRINITY_DN29938_c0_g1_i1	dmso	control	0,98609858	0,26597214	0,01081758	0,04948066
TRINITY_DN44160_c0_g1_i1	dmso	control	0,98582822	0,33877705	0,00989371	0,04611639
TRINITY_DN27011_c0_g1_i1	dmso	control	0,98380462	1,36462151	0,00254228	0,01584652
TRINITY_DN9826_c0_g1_i1	dmso	control	0,98317412	6,64135217	9,34E-13	4,93E-11
TRINITY_DN16871_c0_g1_i1	dmso	control	0,98261679	0,73230996	0,00980656	0,04579883
TRINITY_DN34799_c0_g1_i1	dmso	control	0,98128195	1,39644762	0,00473484	0,02587831
TRINITY_DN50056_c0_g1_i1	dmso	control	0,98079683	1,89422977	0,00072957	0,00569654
TRINITY_DN70037_c0_g1_i1	dmso	control	0,9807792	-0,11676	0,02093292	0,08194471
TRINITY_DN11634_c0_g1_i1	dmso	control	0,98052735	3,4165528	1,93E-06	3,16E-05
TRINITY_DN14262_c0_g1_i1	dmso	control	0,98040942	2,86944805	2,57E-05	0,00031975
TRINITY_DN35943_c0_g1_i1	dmso	control	0,97921739	1,228153	0,00419877	0,02352648
TRINITY_DN10826_c0_g1_i1	dmso	control	0,97917498	3,56149498	3,93E-06	5,94E-05
TRINITY_DN15819_c0_g1_i1	dmso	control	0,97660447	3,09415665	2,53E-05	0,00031561
TRINITY_DN20007_c0_g1_i1	dmso	control	0,97568692	1,5952368	0,00136335	0,00960197
TRINITY_DN15627_c0_g1_i1	dmso	control	0,97387074	2,68003721	0,00013967	0,00139234
TRINITY_DN14971_c0_g1_i1	dmso	control	0,97149938	3,71104448	4,29E-05	0,00050113
TRINITY_DN25545_c0_g1_i1	dmso	control	0,97104608	1,00503153	0,01729798	0,07084471
TRINITY_DN8807_c0_g1_i1	dmso	control	0,97082335	0,95436118	0,01409784	0,06044621
TRINITY_DN23016_c0_g1_i1	dmso	control	0,9704446	1,55967847	0,00952916	0,04473473
TRINITY_DN24922_c0_g1_i1	dmso	control	0,9693832	1,14082583	0,0022235	0,01422427
TRINITY_DN8911_c0_g1_i1	dmso	control	0,96903506	2,7153166	3,88E-05	0,00045932

TRINITY_DN36445_c0_g1_i1	dmso	control	0,96824286	2,96689118	0,0022249	0,01422694
TRINITY_DN21811_c0_g1_i1	dmso	control	0,96424925	2,71775697	9,75E-05	0,00102538
TRINITY_DN19914_c0_g1_i1	dmso	control	0,96152827	3,10303916	7,88E-06	0,0001128
TRINITY_DN40285_c0_g1_i1	dmso	control	0,95938196	1,52297206	0,00108149	0,00792986
TRINITY_DN14918_c0_g1_i1	dmso	control	0,95790551	1,44935275	0,00131148	0,00931593
TRINITY_DN3791_c0_g1_i1	dmso	control	0,95779807	2,2281721	0,00043473	0,00367021
TRINITY_DN19591_c0_g1_i1	dmso	control	0,95721447	4,29752517	1,04E-05	0,0001444
TRINITY_DN21129_c0_g1_i1	dmso	control	0,95714154	1,47484653	0,0020888	0,01353558
TRINITY_DN651_c0_g1_i1	dmso	control	0,95678478	6,04109672	2,04E-08	5,07E-07
TRINITY_DN22237_c0_g1_i1	dmso	control	0,95661417	0,84208481	0,00659097	0,03348668
TRINITY_DN1125_c0_g1_i1	dmso	control	0,95610559	4,88818876	1,72E-10	6,09E-09
TRINITY_DN41008_c0_g1_i1	dmso	control	0,95552031	0,57275912	0,0136207	0,05901349
TRINITY_DN11130_c0_g1_i1	dmso	control	0,9549767	6,32903763	1,30E-15	9,43E-14
TRINITY_DN56_c0_g1_i1	dmso	control	0,95488124	6,23946046	1,49E-06	2,52E-05
TRINITY_DN50313_c0_g1_i1	dmso	control	0,95439671	3,0636788	0,00017506	0,00168136
TRINITY_DN7848_c0_g1_i1	dmso	control	0,95414939	3,51075416	1,61E-06	2,71E-05
TRINITY_DN22156_c0_g1_i1	dmso	control	0,95335262	1,87710802	0,00038435	0,00330279
TRINITY_DN15472_c0_g1_i1	dmso	control	0,95298603	0,8453457	0,00554595	0,02926973
TRINITY_DN7367_c0_g1_i1	dmso	control	0,95253071	3,5232822	7,67E-07	1,38E-05
TRINITY_DN16904_c0_g1_i1	dmso	control	0,95240347	0,73920704	0,00716612	0,03575634
TRINITY_DN21429_c0_g1_i1	dmso	control	0,95072545	2,43702055	0,00328022	0,01947733
TRINITY_DN5968_c0_g1_i1	dmso	control	0,95025968	3,6275907	1,30E-05	0,00017711
TRINITY_DN8952_c0_g1_i1	dmso	control	0,9494109	7,11830386	5,81E-16	4,56E-14
TRINITY_DN13829_c0_g1_i1	dmso	control	0,94901216	2,78271196	0,0003605	0,00313237
TRINITY_DN17600_c0_g1_i1	dmso	control	0,94899858	4,37668133	2,71E-06	4,30E-05
TRINITY_DN18561_c0_g1_i1	dmso	control	0,94800556	3,14232411	5,08E-05	0,00058131
TRINITY_DN21521_c0_g1_i1	dmso	control	0,94709065	2,33713716	0,00012273	0,00124744
TRINITY_DN10669_c0_g1_i1	dmso	control	0,94673338	2,89956097	1,29E-05	0,00017568
TRINITY_DN33583_c0_g1_i1	dmso	control	0,9465168	2,10070062	0,00086128	0,00655493
TRINITY_DN3507_c0_g1_i1	dmso	control	0,94629771	1,69938218	0,00083414	0,00637633
TRINITY_DN9282_c0_g1_i1	dmso	control	0,94609196	5,9169197	3,76E-14	2,33E-12

TRINITY_DN37754_c0_g1_i1	dmso	control	0,94529428	0,14683545	0,02037464	0,08028142
TRINITY_DN75534_c0_g1_i1	dmso	control	0,94502858	1,18725224	0,00335103	0,0197624
TRINITY_DN19207_c0_g1_i1	dmso	control	0,94395974	4,4249349	0,0007018	0,00551251
TRINITY_DN213_c0_g1_i1	dmso	control	0,94382679	5,86690621	1,49E-12	7,43E-11
TRINITY_DN21668_c0_g1_i1	dmso	control	0,94375243	1,13947382	0,00268879	0,01653454
TRINITY_DN46_c0_g1_i1	dmso	control	0,94241071	8,40779486	1,48E-05	0,00019769
TRINITY_DN3775_c0_g1_i1	dmso	control	0,94230974	1,7132171	0,00263732	0,01629432
TRINITY_DN6343_c0_g1_i1	dmso	control	0,94230387	7,18854864	1,90E-11	7,95E-10
TRINITY_DN52677_c0_g1_i1	dmso	control	0,94223962	0,01186871	0,02080222	0,08149935
TRINITY_DN12346_c0_g1_i1	dmso	control	0,94213538	3,8399902	0,00015901	0,00155405
TRINITY_DN1167_c0_g1_i1	dmso	control	0,94168586	5,55929141	1,39E-12	7,07E-11
TRINITY_DN6379_c0_g1_i1	dmso	control	0,94000226	4,17104186	2,25E-07	4,56E-06
TRINITY_DN46543_c0_g1_i1	dmso	control	0,93935645	3,35395861	4,43E-06	6,66E-05
TRINITY_DN10091_c0_g1_i1	dmso	control	0,93928731	3,32674042	2,55E-06	4,06E-05
TRINITY_DN3703_c0_g1_i1	dmso	control	0,9387208	1,30240959	0,00664097	0,03364741
TRINITY_DN44681_c0_g1_i1	dmso	control	0,93860678	4,9715402	0,00043334	0,00366272
TRINITY_DN29984_c0_g1_i1	dmso	control	0,93845761	1,23216638	0,00306644	0,01845495
TRINITY_DN19073_c0_g1_i1	dmso	control	0,93823317	5,93010202	1,17E-11	5,05E-10
TRINITY_DN22602_c0_g1_i1	dmso	control	0,93788278	0,89655792	0,00549898	0,02908562
TRINITY_DN1921_c0_g1_i1	dmso	control	0,93787463	4,63742891	2,13E-06	3,45E-05
TRINITY_DN7008_c0_g1_i1	dmso	control	0,93638941	2,26457556	0,0003989	0,00340864
TRINITY_DN20033_c0_g1_i1	dmso	control	0,93439565	2,55860542	7,24E-05	0,00079391
TRINITY_DN20309_c0_g1_i1	dmso	control	0,93319573	3,60969572	7,01E-05	0,00077106
TRINITY_DN17608_c0_g1_i1	dmso	control	0,93319517	3,29530734	7,73E-06	0,00011093
TRINITY_DN11586_c0_g1_i1	dmso	control	0,93110622	3,82742866	4,55E-07	8,62E-06
TRINITY_DN11690_c0_g1_i1	dmso	control	0,92963993	2,1507005	0,00205171	0,01334996
TRINITY_DN21369_c0_g1_i1	dmso	control	0,9289341	1,69002704	0,00126657	0,00905725
TRINITY_DN1829_c0_g1_i1	dmso	control	0,92584742	3,73507177	5,55E-07	1,03E-05
TRINITY_DN18375_c0_g1_i1	dmso	control	0,92575005	4,52764436	5,93E-05	0,0006667
TRINITY_DN1271_c0_g1_i1	dmso	control	0,92435698	3,0703589	7,33E-05	0,00080174
TRINITY_DN6354_c0_g1_i1	dmso	control	0,92402324	0,38423253	0,01359412	0,05895137

TRINITY_DN16917_c0_g1_i1	dmso	control	0,92392276	0,13644023	0,02432426	0,0915317
TRINITY_DN27400_c0_g1_i1	dmso	control	0,92375497	0,80430069	0,00899662	0,04266452
TRINITY_DN589_c0_g1_i1	dmso	control	0,92278477	4,38846926	4,89E-07	9,16E-06
TRINITY_DN12358_c0_g1_i1	dmso	control	0,92250427	0,74991838	0,00895045	0,04254333
TRINITY_DN5957_c0_g1_i1	dmso	control	0,92225283	5,18918858	2,93E-06	4,60E-05
TRINITY_DN12949_c0_g1_i1	dmso	control	0,92129123	3,56849655	0,00021638	0,00201657
TRINITY_DN19561_c0_g1_i1	dmso	control	0,92103273	2,74192916	0,00017291	0,00166399
TRINITY_DN16620_c0_g1_i1	dmso	control	0,92096747	2,76067619	3,74E-05	0,00044395
TRINITY_DN32964_c0_g1_i1	dmso	control	0,92037182	4,31209703	0,00037622	0,0032464
TRINITY_DN11234_c0_g1_i1	dmso	control	0,91951124	5,61731246	8,67E-10	2,75E-08
TRINITY_DN10829_c0_g1_i1	dmso	control	0,9191968	3,78965307	1,43E-06	2,43E-05
TRINITY_DN25358_c0_g1_i1	dmso	control	0,91903317	1,62760182	0,00251974	0,01573615
TRINITY_DN11941_c0_g1_i1	dmso	control	0,91882036	1,42304039	0,00518023	0,02771422
TRINITY_DN7273_c0_g1_i1	dmso	control	0,91846574	2,93708713	2,79E-05	0,00034211
TRINITY_DN20389_c0_g1_i1	dmso	control	0,91842545	1,64705382	0,00486214	0,0263937
TRINITY_DN18762_c0_g1_i1	dmso	control	0,91696898	6,28254656	2,26E-13	1,28E-11
TRINITY_DN19679_c0_g1_i1	dmso	control	0,91651729	2,60267882	0,00012167	0,00123771
TRINITY_DN5820_c0_g1_i1	dmso	control	0,91624776	8,45502538	4,79E-10	1,58E-08
TRINITY_DN23459_c0_g1_i1	dmso	control	0,91602426	2,41827654	0,00151703	0,01047488
TRINITY_DN5049_c0_g1_i1	dmso	control	0,91368978	6,61653128	1,22E-13	6,99E-12
TRINITY_DN2088_c0_g1_i1	dmso	control	0,9119619	2,80077263	7,63E-05	0,00082843
TRINITY_DN15628_c0_g1_i1	dmso	control	0,91173336	1,37355239	0,00504484	0,02711036
TRINITY_DN33674_c0_g1_i1	dmso	control	0,91148158	3,92113009	3,15E-07	6,17E-06
TRINITY_DN6289_c0_g1_i1	dmso	control	0,9109476	4,65193353	3,72E-09	1,04E-07
TRINITY_DN33078_c0_g1_i1	dmso	control	0,91094615	3,29073334	8,27E-05	0,0008851
TRINITY_DN22381_c0_g1_i1	dmso	control	0,91094485	3,76676102	8,66E-06	0,00012238
TRINITY_DN10395_c0_g1_i1	dmso	control	0,91089548	2,884604	0,00053265	0,00438022
TRINITY_DN4834_c0_g1_i1	dmso	control	0,91032496	2,66804636	0,00114044	0,00826992
TRINITY_DN15082_c0_g1_i1	dmso	control	0,90731381	1,46367574	0,00365582	0,02116463
TRINITY_DN8164_c0_g1_i1	dmso	control	0,9072843	3,2795898	8,26E-06	0,0001176
TRINITY_DN12805_c0_g1_i1	dmso	control	0,90649152	2,46447287	0,00061206	0,0049145

TRINITY_DN15495_c0_g1_i1	dmso	control	0,90475531	0,94977483	0,00761204	0,03750202
TRINITY_DN5060_c0_g1_i1	dmso	control	0,90469598	1,94806131	0,00426896	0,02382734
TRINITY_DN2038_c0_g1_i1	dmso	control	0,9028432	3,71316804	1,67E-06	2,79E-05
TRINITY_DN26547_c0_g1_i1	dmso	control	0,90092137	0,10970861	0,02537833	0,09460386
TRINITY_DN18940_c0_g1_i1	dmso	control	0,9007095	5,53829662	1,07E-11	4,66E-10
TRINITY_DN8493_c0_g1_i1	dmso	control	0,899749	0,80801269	0,01152022	0,05192185
TRINITY_DN2226_c0_g1_i1	dmso	control	0,89943389	5,42066818	3,84E-11	1,52E-09
TRINITY_DN8366_c0_g1_i1	dmso	control	0,89934556	1,90612537	0,00274999	0,01685038
TRINITY_DN36418_c0_g1_i1	dmso	control	0,89838953	0,76232053	0,01358816	0,05894325
TRINITY_DN2917_c0_g1_i1	dmso	control	0,89824425	1,36123424	0,00464314	0,02549226
TRINITY_DN2240_c0_g1_i1	dmso	control	0,89689245	6,34882861	4,61E-12	2,08E-10
TRINITY_DN810_c0_g1_i1	dmso	control	0,89648639	5,87215108	2,92E-09	8,38E-08
TRINITY_DN10063_c0_g1_i1	dmso	control	0,89621355	1,06201457	0,01487845	0,0629892
TRINITY_DN2523_c0_g1_i1	dmso	control	0,89566201	2,87432144	0,00020204	0,00190007
TRINITY_DN7132_c0_g1_i1	dmso	control	0,89506869	3,41553559	1,10E-05	0,00015331
TRINITY_DN1838_c0_g1_i1	dmso	control	0,89454473	4,61745507	3,65E-08	8,66E-07
TRINITY_DN1118_c0_g1_i1	dmso	control	0,89340913	4,15279275	7,94E-05	0,00085771
TRINITY_DN13558_c0_g1_i1	dmso	control	0,89333764	1,74862189	0,0201525	0,07972521
TRINITY_DN25381_c0_g1_i1	dmso	control	0,89172016	1,56724297	0,01104918	0,05025435
TRINITY_DN27449_c0_g1_i1	dmso	control	0,8911641	0,70091939	0,01148503	0,05177942
TRINITY_DN1012_c0_g1_i1	dmso	control	0,88957485	5,19525337	6,05E-09	1,64E-07
TRINITY_DN8712_c0_g1_i1	dmso	control	0,88848424	0,43174254	0,02632431	0,09717728
TRINITY_DN23174_c0_g1_i1	dmso	control	0,8884637	2,09508285	0,00060701	0,00488482
TRINITY_DN4926_c0_g1_i1	dmso	control	0,8883671	6,38973093	0,00010863	0,00112162
TRINITY_DN19024_c0_g1_i1	dmso	control	0,88726887	5,82944712	2,89E-06	4,54E-05
TRINITY_DN33371_c0_g1_i1	dmso	control	0,88669609	3,57023465	3,18E-05	0,00038395
TRINITY_DN1211_c0_g1_i1	dmso	control	0,88664596	4,80684632	5,13E-08	1,19E-06
TRINITY_DN23381_c0_g1_i1	dmso	control	0,88660963	1,4283297	0,01555382	0,06512318
TRINITY_DN5181_c0_g1_i1	dmso	control	0,88605391	5,08561292	1,92E-07	4,00E-06
TRINITY_DN53404_c0_g1_i1	dmso	control	0,88451219	1,56318794	0,01364795	0,05909612
TRINITY_DN1942_c0_g1_i1	dmso	control	0,88441928	8,08956635	1,29E-12	6,56E-11

TRINITY_DN5822_c0_g1_i1	dmso	control	0,88299862	3,96383577	0,00114005	0,00826992
TRINITY_DN34606_c0_g1_i1	dmso	control	0,88297179	1,90180304	0,00113636	0,00825597
TRINITY_DN2469_c0_g1_i1	dmso	control	0,88210449	2,76996324	0,00011136	0,00114821
TRINITY_DN1021_c0_g1_i1	dmso	control	0,88176641	4,83147138	1,34E-08	3,47E-07
TRINITY_DN2414_c0_g1_i1	dmso	control	0,88097497	2,3212104	0,00173482	0,01168275
TRINITY_DN32716_c0_g1_i1	dmso	control	0,88083408	2,29769811	0,00086113	0,00655493
TRINITY_DN17399_c0_g1_i1	dmso	control	0,88004678	2,88555193	0,00013904	0,00138799
TRINITY_DN2617_c0_g1_i1	dmso	control	0,87998879	6,21313996	8,15E-12	3,59E-10
TRINITY_DN21121_c0_g1_i1	dmso	control	0,87958898	2,2001368	0,00132779	0,0094019
TRINITY_DN17986_c0_g1_i1	dmso	control	0,87957153	0,50918298	0,01596373	0,06662699
TRINITY_DN20664_c0_g1_i1	dmso	control	0,87867501	3,07799161	0,00013112	0,00131676
TRINITY_DN138_c0_g1_i1	dmso	control	0,87794258	4,82323963	6,90E-07	1,25E-05
TRINITY_DN5424_c0_g1_i1	dmso	control	0,87760906	4,83463836	1,21E-07	2,65E-06
TRINITY_DN17338_c0_g1_i1	dmso	control	0,87680707	0,83757445	0,01432622	0,061099
TRINITY_DN20229_c0_g1_i1	dmso	control	0,87656712	1,30866777	0,0055322	0,02922921
TRINITY_DN31288_c0_g1_i1	dmso	control	0,8764256	0,23527352	0,02604932	0,09643323
TRINITY_DN3675_c0_g1_i1	dmso	control	0,8761599	2,49432698	0,00107953	0,00791964
TRINITY_DN10687_c0_g1_i1	dmso	control	0,87613333	5,2087068	2,81E-07	5,57E-06
TRINITY_DN14059_c0_g1_i1	dmso	control	0,87583574	1,20441386	0,00460944	0,02537478
TRINITY_DN24725_c0_g1_i1	dmso	control	0,87567331	4,5314234	4,46E-07	8,49E-06
TRINITY_DN6632_c0_g1_i1	dmso	control	0,87477543	3,57993936	2,87E-05	0,00035148
TRINITY_DN33792_c0_g1_i1	dmso	control	0,87413111	2,78334122	0,00010591	0,00109747
TRINITY_DN2918_c0_g1_i1	dmso	control	0,8737077	4,34374778	6,05E-07	1,11E-05
TRINITY_DN22732_c0_g1_i1	dmso	control	0,8735184	2,43725552	0,00221759	0,01419908
TRINITY_DN8265_c0_g1_i1	dmso	control	0,87171118	2,70519134	0,00014831	0,00146538
TRINITY_DN20969_c0_g1_i1	dmso	control	0,87055457	1,20216852	0,00611032	0,03157909
TRINITY_DN2461_c0_g1_i1	dmso	control	0,86992543	3,83172227	1,55E-05	0,00020541
TRINITY_DN1871_c0_g1_i1	dmso	control	0,86975097	2,32116162	0,0049069	0,0266067
TRINITY_DN2375_c0_g1_i1	dmso	control	0,86954229	3,55258585	1,15E-05	0,00015976
TRINITY_DN16788_c0_g1_i1	dmso	control	0,86888095	2,52330534	0,00023787	0,00218977
TRINITY_DN28948_c0_g1_i1	dmso	control	0,8687346	1,79411633	0,0019169	0,01265525

TRINITY_DN22609_c0_g1_i1	dmso	control	0,86836391	2,6202298	0,00027084	0,00245438
TRINITY_DN19346_c0_g1_i1	dmso	control	0,86734543	3,45466749	3,45E-05	0,0004143
TRINITY_DN13178_c0_g1_i1	dmso	control	0,86704658	4,8133148	1,84E-06	3,03E-05
TRINITY_DN5275_c0_g1_i1	dmso	control	0,86621212	4,4380622	2,65E-07	5,31E-06
TRINITY_DN4088_c0_g1_i1	dmso	control	0,86598776	4,55881743	2,74E-05	0,00033806
TRINITY_DN9227_c0_g1_i1	dmso	control	0,86517749	2,5349355	0,00047333	0,0039527
TRINITY_DN11978_c0_g1_i1	dmso	control	0,86435964	5,65483547	2,45E-11	1,00E-09
TRINITY_DN42870_c0_g1_i1	dmso	control	0,86429313	3,70276766	1,49E-05	0,00019853
TRINITY_DN23051_c0_g1_i1	dmso	control	0,86373296	1,503239	0,00448372	0,02483409
TRINITY_DN21667_c0_g1_i1	dmso	control	0,86331439	3,06968495	5,80E-05	0,00065474
TRINITY_DN31509_c0_g1_i1	dmso	control	0,86327551	0,62095681	0,02423848	0,09134485
TRINITY_DN7756_c0_g1_i1	dmso	control	0,86297745	3,15524905	3,17E-05	0,00038389
TRINITY_DN22927_c0_g1_i1	dmso	control	0,86205756	2,61780438	0,00261433	0,0161938
TRINITY_DN14583_c0_g1_i1	dmso	control	0,86202498	0,68537944	0,01539847	0,06467895
TRINITY_DN449_c0_g1_i1	dmso	control	0,86147326	6,66585891	2,94E-11	1,18E-09
TRINITY_DN9890_c0_g1_i1	dmso	control	0,86114694	1,16604605	0,01146343	0,05172348
TRINITY_DN7691_c0_g1_i1	dmso	control	0,86084205	2,88876314	0,00073749	0,0057511
TRINITY_DN19430_c0_g1_i1	dmso	control	0,86023453	5,22788466	2,18E-09	6,49E-08
TRINITY_DN10276_c0_g1_i1	dmso	control	0,85996104	4,41497051	1,64E-07	3,46E-06
TRINITY_DN7375_c0_g1_i1	dmso	control	0,85874572	4,11066109	4,56E-06	6,85E-05
TRINITY_DN1746_c0_g1_i1	dmso	control	0,85845233	5,51722083	3,04E-07	5,98E-06
TRINITY_DN12315_c0_g1_i1	dmso	control	0,8577428	0,66497505	0,02498532	0,09344707
TRINITY_DN8702_c0_g1_i1	dmso	control	0,85712229	3,28277912	0,00047597	0,00396507
TRINITY_DN25465_c0_g1_i1	dmso	control	0,85687843	4,25049837	7,00E-07	1,27E-05
TRINITY_DN30641_c0_g1_i1	dmso	control	0,85559705	2,57551368	0,00029463	0,00264183
TRINITY_DN12275_c0_g1_i1	dmso	control	0,85415675	3,46719766	5,07E-05	0,00057985
TRINITY_DN12308_c0_g1_i1	dmso	control	0,85400982	4,41177317	8,25E-07	1,47E-05
TRINITY_DN22083_c0_g1_i1	dmso	control	0,8536384	7,86727002	1,27E-12	6,51E-11
TRINITY_DN23534_c0_g1_i1	dmso	control	0,8534853	3,15807144	0,00139536	0,00977497
TRINITY_DN10454_c0_g1_i1	dmso	control	0,85340534	1,52260111	0,00358423	0,02084308
TRINITY_DN2093_c0_g1_i1	dmso	control	0,85302057	5,77520626	1,13E-07	2,49E-06

TRINITY_DN22554_c0_g1_i1	dmso	control	0,85186648	1,50833335	0,00662873	0,03360898
TRINITY_DN26671_c0_g1_i1	dmso	control	0,85016458	1,21391415	0,00825799	0,04001563
TRINITY_DN14674_c0_g1_i1	dmso	control	0,85012776	1,96421373	0,00571624	0,02999395
TRINITY_DN7959_c0_g1_i1	dmso	control	0,85008084	3,20920824	0,0002222	0,00206412
TRINITY_DN13251_c0_g1_i1	dmso	control	0,84981373	2,14342288	0,00171729	0,01159169
TRINITY_DN3154_c0_g1_i1	dmso	control	0,84978511	3,32504519	6,08E-05	0,00068206
TRINITY_DN13873_c0_g1_i1	dmso	control	0,84903478	1,62231121	0,01983643	0,07878116
TRINITY_DN21192_c0_g1_i1	dmso	control	0,84800151	2,06943005	0,00885798	0,04220265
TRINITY_DN2061_c0_g1_i1	dmso	control	0,84795498	2,69825516	0,01790492	0,07275289
TRINITY_DN19546_c0_g1_i1	dmso	control	0,84672582	2,8857871	0,00014244	0,00141609
TRINITY_DN296_c0_g1_i1	dmso	control	0,84602221	4,46879172	4,87E-05	0,000561
TRINITY_DN2034_c0_g1_i1	dmso	control	0,84477636	6,0823093	1,77E-11	7,44E-10
TRINITY_DN13287_c0_g1_i1	dmso	control	0,84344431	2,99222205	8,99E-05	0,00095236
TRINITY_DN7257_c0_g1_i1	dmso	control	0,84320866	3,53163923	3,74E-05	0,00044395
TRINITY_DN21749_c0_g1_i1	dmso	control	0,84257357	1,6806754	0,00890282	0,04235879
TRINITY_DN13562_c0_g1_i1	dmso	control	0,84204354	1,5941657	0,00379807	0,02177106
TRINITY_DN46489_c0_g1_i1	dmso	control	0,84152537	0,66778324	0,01738912	0,07113732
TRINITY_DN13637_c0_g1_i1	dmso	control	0,84065061	2,38296014	0,00895944	0,04257209
TRINITY_DN9003_c0_g1_i1	dmso	control	0,84056837	3,55881102	1,30E-05	0,00017638
TRINITY_DN12263_c0_g1_i1	dmso	control	0,8403307	3,72648911	0,00041785	0,00355256
TRINITY_DN19317_c0_g1_i1	dmso	control	0,83980768	4,8134182	0,00030563	0,0027252
TRINITY_DN3760_c0_g1_i1	dmso	control	0,83951985	3,70814862	1,51E-05	0,00020116
TRINITY_DN1562_c0_g1_i1	dmso	control	0,83930163	4,93997948	1,29E-05	0,00017604
TRINITY_DN32745_c0_g1_i1	dmso	control	0,83929505	3,41061072	0,0002291	0,0021241
TRINITY_DN5920_c0_g1_i1	dmso	control	0,83900519	4,35388084	5,16E-07	9,61E-06
TRINITY_DN8367_c0_g1_i1	dmso	control	0,83849245	2,73194461	0,00019547	0,00184786
TRINITY_DN6601_c0_g1_i1	dmso	control	0,83817272	2,51856584	0,01823412	0,07379992
TRINITY_DN7079_c0_g1_i1	dmso	control	0,83618593	2,25148577	0,00287797	0,01750154
TRINITY_DN1545_c0_g1_i1	dmso	control	0,83588584	5,38874076	2,98E-08	7,17E-07
TRINITY_DN8311_c0_g1_i1	dmso	control	0,83554229	2,86698013	0,00365412	0,02116429
TRINITY_DN20906_c0_g1_i1	dmso	control	0,835218	3,07606975	8,92E-05	0,00094587

TRINITY_DN17092_c0_g1_i1	dmso	control	0,83496187	1,01952754	0,01993633	0,07902951
TRINITY_DN18858_c0_g1_i1	dmso	control	0,83474204	6,81881167	9,41E-13	4,94E-11
TRINITY_DN2773_c0_g1_i1	dmso	control	0,834667	3,05109633	0,00036079	0,00313237
TRINITY_DN11550_c0_g1_i1	dmso	control	0,83453849	4,63336843	1,35E-06	2,30E-05
TRINITY_DN2050_c0_g1_i1	dmso	control	0,8338299	4,34876371	7,73E-05	0,00083855
TRINITY_DN120_c0_g1_i1	dmso	control	0,83350526	7,62524859	9,44E-05	0,00099572
TRINITY_DN3503_c0_g1_i1	dmso	control	0,83334094	4,04646521	0,00057691	0,00467911
TRINITY_DN27161_c0_g1_i1	dmso	control	0,83291552	1,63960426	0,00426331	0,02380501
TRINITY_DN1445_c0_g1_i1	dmso	control	0,83272954	7,36662413	7,92E-05	0,00085693
TRINITY_DN15054_c0_g1_i1	dmso	control	0,83243409	2,43106933	0,00095165	0,0070822
TRINITY_DN34367_c0_g1_i1	dmso	control	0,83231806	4,63324785	4,29E-07	8,21E-06
TRINITY_DN33017_c0_g1_i1	dmso	control	0,83189428	4,94245143	7,26E-08	1,65E-06
TRINITY_DN8858_c0_g1_i1	dmso	control	0,83161537	1,00095125	0,0129499	0,05696143
TRINITY_DN6815_c0_g1_i1	dmso	control	0,83080455	2,63836505	0,00078428	0,00606796
TRINITY_DN23463_c0_g1_i1	dmso	control	0,83071269	2,5623916	0,00080435	0,00619013
TRINITY_DN1822_c0_g1_i1	dmso	control	0,82976002	5,47738167	7,70E-10	2,47E-08
TRINITY_DN13594_c0_g1_i1	dmso	control	0,82939544	2,3689515	0,0033047	0,01956407
TRINITY_DN6917_c0_g1_i1	dmso	control	0,82864392	4,74540368	4,39E-06	6,62E-05
TRINITY_DN6925_c0_g1_i1	dmso	control	0,82863918	3,32643861	3,18E-05	0,00038409
TRINITY_DN3603_c0_g1_i1	dmso	control	0,82855813	1,32997972	0,0086211	0,04133147
TRINITY_DN27749_c0_g1_i1	dmso	control	0,8282682	0,64721086	0,02250965	0,0865226
TRINITY_DN14598_c0_g1_i1	dmso	control	0,82734535	3,33602218	7,10E-05	0,00077996
TRINITY_DN25822_c0_g1_i1	dmso	control	0,8271599	2,04646163	0,00473674	0,02587831
TRINITY_DN20419_c0_g1_i1	dmso	control	0,82675302	2,94488649	0,00013117	0,00131676
TRINITY_DN8051_c0_g1_i1	dmso	control	0,82655626	2,20880663	0,0087586	0,04183979
TRINITY_DN15094_c0_g1_i1	dmso	control	0,82602088	1,09657692	0,01841695	0,07435238
TRINITY_DN20230_c0_g1_i1	dmso	control	0,82511512	5,0841265	2,40E-07	4,86E-06
TRINITY_DN15436_c0_g1_i1	dmso	control	0,82495593	1,00183389	0,01320969	0,05775241
TRINITY_DN22515_c0_g1_i1	dmso	control	0,8228965	0,64204762	0,02186031	0,08465744
TRINITY_DN22795_c0_g1_i1	dmso	control	0,82221005	2,91925294	0,0022155	0,01419195
TRINITY_DN854_c0_g1_i1	dmso	control	0,82217667	4,22154765	5,87E-05	0,00066106

TRINITY_DN35647_c0_g1_i1	dmso	control	0,8221464	3,42000121	0,00077324	0,00598577
TRINITY_DN2384_c0_g1_i1	dmso	control	0,82179586	3,52920013	2,06E-05	0,00026341
TRINITY_DN3688_c0_g1_i1	dmso	control	0,82022555	1,75876864	0,01494146	0,0631819
TRINITY_DN10267_c0_g1_i1	dmso	control	0,81683063	5,06339847	1,30E-08	3,36E-07
TRINITY_DN11951_c0_g1_i1	dmso	control	0,81679462	3,24891186	0,00081684	0,00626285
TRINITY_DN3069_c0_g1_i1	dmso	control	0,81609957	2,61929575	0,00259924	0,01611774
TRINITY_DN36505_c0_g1_i1	dmso	control	0,81502128	1,02015125	0,01639162	0,06788329
TRINITY_DN18340_c0_g1_i1	dmso	control	0,81373347	5,90608936	4,76E-10	1,57E-08
TRINITY_DN19570_c0_g1_i1	dmso	control	0,81284586	3,98048209	0,00329701	0,01953664
TRINITY_DN16194_c0_g1_i1	dmso	control	0,81201024	1,48513628	0,00571253	0,02999395
TRINITY_DN33634_c0_g1_i1	dmso	control	0,81173526	4,59460223	1,55E-05	0,00020541
TRINITY_DN3072_c0_g1_i1	dmso	control	0,81122785	4,33141391	2,18E-05	0,00027551
TRINITY_DN6534_c0_g1_i1	dmso	control	0,81099837	5,63993761	4,61E-07	8,72E-06
TRINITY_DN186_c0_g1_i1	dmso	control	0,81079737	4,31518946	7,12E-06	0,00010299
TRINITY_DN8419_c0_g1_i1	dmso	control	0,81004262	0,93206553	0,01877748	0,07544792
TRINITY_DN38826_c0_g1_i1	dmso	control	0,81003449	2,86801547	0,00090183	0,0067743
TRINITY_DN9060_c0_g1_i1	dmso	control	0,80928896	3,72275751	4,01E-05	0,00047215
TRINITY_DN15575_c0_g1_i1	dmso	control	0,80886	1,1592837	0,02345865	0,0891972
TRINITY_DN26613_c0_g1_i1	dmso	control	0,80713861	1,23348224	0,01686321	0,06950809
TRINITY_DN27142_c0_g1_i1	dmso	control	0,80653773	0,84540226	0,01758723	0,0718054
TRINITY_DN29499_c0_g1_i1	dmso	control	0,80550357	0,9243379	0,02007922	0,07948599
TRINITY_DN6111_c0_g1_i1	dmso	control	0,80524544	6,10019234	1,75E-06	2,90E-05
TRINITY_DN10541_c0_g1_i1	dmso	control	0,8042603	3,14075549	0,0002711	0,00245521
TRINITY_DN5417_c0_g1_i1	dmso	control	0,80321291	5,23696124	3,31E-08	7,89E-07
TRINITY_DN20537_c0_g1_i1	dmso	control	0,80278887	2,62746464	0,02469627	0,09261103
TRINITY_DN781_c0_g1_i1	dmso	control	0,8021053	7,39247837	3,49E-07	6,78E-06
TRINITY_DN20602_c0_g1_i1	dmso	control	0,8018571	1,28775423	0,01114616	0,05058883
TRINITY_DN12388_c0_g1_i1	dmso	control	0,80144429	2,41858099	0,00118076	0,00852381
TRINITY_DN33407_c0_g1_i1	dmso	control	0,80124589	1,64859653	0,00669349	0,03384232
TRINITY_DN76929_c0_g1_i1	dmso	control	0,8005746	3,88706606	1,28E-05	0,00017455
TRINITY_DN10937_c0_g1_i1	dmso	control	0,80037698	7,00479563	2,29E-12	1,10E-10

TRINITY_DN33317_c0_g1_i1	dmso	control	0,79892093	1,80143535	0,01251906	0,05559109
TRINITY_DN39813_c0_g1_i1	dmso	control	0,79854118	1,01864948	0,01447407	0,06156534
TRINITY_DN12580_c0_g1_i1	dmso	control	0,79837693	3,42625665	0,0002562	0,0023364
TRINITY_DN33172_c0_g1_i1	dmso	control	0,79820601	1,99879878	0,00352935	0,02063499
TRINITY_DN23706_c0_g1_i1	dmso	control	0,79782448	2,62372357	0,00057598	0,00467414
TRINITY_DN54100_c0_g1_i1	dmso	control	0,79778839	1,0708072	0,01813413	0,07351882
TRINITY_DN15485_c0_g1_i1	dmso	control	0,79649546	1,46676747	0,01570448	0,06567781
TRINITY_DN4977_c0_g1_i1	dmso	control	0,79632801	6,32937446	4,62E-09	1,28E-07
TRINITY_DN23059_c0_g1_i1	dmso	control	0,79483336	2,80103825	0,00092446	0,0069089
TRINITY_DN9095_c0_g1_i1	dmso	control	0,79468431	3,36696927	0,00012665	0,00127845
TRINITY_DN20435_c0_g1_i1	dmso	control	0,79449719	2,71644692	0,00333835	0,01970678
TRINITY_DN12780_c0_g1_i1	dmso	control	0,79436665	4,48212969	1,27E-06	2,17E-05
TRINITY_DN5881_c0_g1_i1	dmso	control	0,79426716	5,65428594	7,55E-08	1,70E-06
TRINITY_DN293_c0_g1_i1	dmso	control	0,79306852	6,08538846	1,34E-08	3,47E-07
TRINITY_DN34408_c0_g1_i1	dmso	control	0,79085331	1,93544878	0,00423236	0,02367792
TRINITY_DN43638_c0_g1_i1	dmso	control	0,79032495	1,2605035	0,02554423	0,09502612
TRINITY_DN1789_c0_g1_i1	dmso	control	0,79017187	1,26946938	0,01161558	0,05220506
TRINITY_DN19162_c0_g1_i1	dmso	control	0,78981419	4,22751134	2,18E-05	0,00027567
TRINITY_DN22660_c0_g1_i1	dmso	control	0,78965349	3,13563817	0,00024538	0,00224759
TRINITY_DN8039_c0_g1_i1	dmso	control	0,78895246	2,14141097	0,00295119	0,01785919
TRINITY_DN7228_c0_g1_i1	dmso	control	0,78890766	3,898734	1,17E-05	0,00016191
TRINITY_DN38596_c0_g1_i1	dmso	control	0,78859181	1,0003821	0,02206715	0,08511614
TRINITY_DN13483_c0_g1_i1	dmso	control	0,7881575	3,07383923	0,00021005	0,00196509
TRINITY_DN11208_c0_g1_i1	dmso	control	0,7861098	4,95671043	0,00066897	0,00530369
TRINITY_DN11675_c0_g1_i1	dmso	control	0,78605575	3,98924964	0,00015182	0,00149291
TRINITY_DN40799_c0_g1_i1	dmso	control	0,78575025	0,69940934	0,02394361	0,09067161
TRINITY_DN2628_c0_g1_i1	dmso	control	0,78519707	5,87346573	3,41E-07	6,64E-06
TRINITY_DN20232_c0_g1_i1	dmso	control	0,78454957	3,47042409	7,93E-05	0,00085771
TRINITY_DN33430_c0_g1_i1	dmso	control	0,78429778	3,18642022	0,00011962	0,00121937
TRINITY_DN1697_c0_g1_i1	dmso	control	0,78354596	6,11055073	2,91E-06	4,56E-05
TRINITY_DN30714_c0_g1_i1	dmso	control	0,78337565	1,49808814	0,00742421	0,03676952

TRINITY_DN6087_c0_g1_i1	dmso	control	0,78277336	5,0360993	1,75E-05	0,00022871
TRINITY_DN10045_c0_g1_i1	dmso	control	0,78249382	2,38945958	0,00134996	0,00952612
TRINITY_DN12625_c0_g1_i1	dmso	control	0,78138109	3,41124699	0,00075657	0,00588198
TRINITY_DN11656_c0_g1_i1	dmso	control	0,78121976	3,67866863	0,00035783	0,00311222
TRINITY_DN20627_c0_g1_i1	dmso	control	0,78061364	3,07937028	0,00510636	0,02740714
TRINITY_DN13941_c0_g1_i1	dmso	control	0,78036562	1,31870065	0,0161965	0,06740375
TRINITY_DN23045_c0_g1_i1	dmso	control	0,78011166	2,03892892	0,00356085	0,02075726
TRINITY_DN43423_c0_g1_i1	dmso	control	0,77996669	0,86885151	0,02218102	0,08547816
TRINITY_DN2180_c0_g1_i1	dmso	control	0,77959014	3,71688878	5,84E-05	0,00065891
TRINITY_DN3046_c0_g1_i1	dmso	control	0,77859185	4,64438261	0,00034551	0,00302418
TRINITY_DN22068_c0_g1_i1	dmso	control	0,77857154	4,58766425	1,77E-05	0,00023072
TRINITY_DN6291_c0_g1_i1	dmso	control	0,77784468	4,80243458	4,47E-07	8,50E-06
TRINITY_DN5946_c0_g1_i1	dmso	control	0,77776383	3,4658246	0,00012529	0,00126647
TRINITY_DN3193_c0_g1_i1	dmso	control	0,77769087	4,15107539	1,35E-05	0,00018274
TRINITY_DN20205_c0_g1_i1	dmso	control	0,77644416	2,49025639	0,00133163	0,00941991
TRINITY_DN29991_c0_g1_i1	dmso	control	0,77498384	1,55991763	0,00886586	0,04221071
TRINITY_DN12399_c0_g1_i1	dmso	control	0,77470254	4,30205702	0,00034565	0,00302418
TRINITY_DN11523_c0_g1_i1	dmso	control	0,77449827	2,65202539	0,00092516	0,00690999
TRINITY_DN21986_c0_g1_i1	dmso	control	0,77449688	1,56127281	0,01348296	0,05866305
TRINITY_DN32668_c0_g1_i1	dmso	control	0,77369279	2,74495064	0,00079417	0,00612481
TRINITY_DN6665_c0_g1_i1	dmso	control	0,77356481	4,34495438	8,00E-06	0,00011422
TRINITY_DN21792_c0_g1_i1	dmso	control	0,77334507	6,81766089	1,50E-11	6,35E-10
TRINITY_DN3632_c0_g1_i1	dmso	control	0,77291701	3,5128114	8,07E-05	0,00086919
TRINITY_DN276_c0_g1_i1	dmso	control	0,77206301	5,62552805	4,58E-09	1,27E-07
TRINITY_DN78375_c0_g1_i1	dmso	control	0,77198709	2,03666182	0,00801701	0,03908913
TRINITY_DN22610_c0_g1_i1	dmso	control	0,77088597	2,0571348	0,01040844	0,04793855
TRINITY_DN8149_c0_g1_i1	dmso	control	0,77069421	1,07714164	0,02027886	0,0800565
TRINITY_DN5598_c0_g1_i1	dmso	control	0,77061693	4,98626711	5,94E-05	0,00066749
TRINITY_DN180_c0_g1_i1	dmso	control	0,77041834	6,16575275	3,53E-09	9,94E-08
TRINITY_DN23682_c0_g1_i1	dmso	control	0,77010674	1,23783962	0,02228896	0,08583419
TRINITY_DN20473_c0_g1_i1	dmso	control	0,76944879	5,31476565	1,65E-07	3,47E-06

TRINITY_DN6619_c0_g1_i1	dmso	control	0,76929941	1,75462763	0,00548693	0,02904091
TRINITY_DN5197_c0_g1_i1	dmso	control	0,76766313	5,92568979	2,60E-09	7,63E-08
TRINITY_DN34973_c0_g1_i1	dmso	control	0,76722993	2,48247604	0,00373235	0,02150533
TRINITY_DN11010_c0_g1_i1	dmso	control	0,76695666	4,79960769	8,83E-07	1,56E-05
TRINITY_DN11268_c0_g1_i1	dmso	control	0,76675277	3,93090436	1,72E-05	0,00022475
TRINITY_DN12073_c0_g1_i1	dmso	control	0,76609503	3,58302872	0,00011896	0,00121352
TRINITY_DN6761_c0_g1_i1	dmso	control	0,76492046	4,17977881	0,02576179	0,09564617
TRINITY_DN7076_c0_g1_i1	dmso	control	0,7647673	4,10485588	8,16E-05	0,00087768
TRINITY_DN7642_c0_g1_i1	dmso	control	0,76446776	2,90474388	0,00540446	0,02870127
TRINITY_DN23347_c0_g1_i1	dmso	control	0,7639473	2,87633935	0,01004809	0,04659897
TRINITY_DN34034_c0_g1_i1	dmso	control	0,7636005	1,9106395	0,00649173	0,03306523
TRINITY_DN6247_c0_g1_i1	dmso	control	0,76336795	4,97515951	7,77E-05	0,00084128
TRINITY_DN5666_c0_g1_i1	dmso	control	0,76295741	2,89833458	0,00342537	0,02011519
TRINITY_DN654_c0_g1_i1	dmso	control	0,76290406	4,8037147	0,00033049	0,00292001
TRINITY_DN12972_c0_g1_i1	dmso	control	0,76267054	4,23774529	2,88E-05	0,00035171
TRINITY_DN9115_c0_g1_i1	dmso	control	0,7609522	1,97338733	0,02127537	0,08292571
TRINITY_DN32767_c0_g1_i1	dmso	control	0,7604028	1,42839867	0,01385385	0,05968354
TRINITY_DN7908_c0_g1_i1	dmso	control	0,76010673	4,18110265	1,00E-05	0,00014038
TRINITY_DN2483_c0_g1_i1	dmso	control	0,75961308	3,10001196	0,00044254	0,00372302
TRINITY_DN9930_c0_g1_i1	dmso	control	0,75938839	3,26292515	0,00013951	0,00139173
TRINITY_DN20676_c0_g1_i1	dmso	control	0,75915225	2,3609903	0,02025093	0,07997263
TRINITY_DN1845_c0_g1_i1	dmso	control	0,75827976	4,08755893	1,80E-05	0,0002335
TRINITY_DN11178_c0_g1_i1	dmso	control	0,75802804	4,72794659	9,21E-06	0,00012926
TRINITY_DN19603_c0_g1_i1	dmso	control	0,75787248	3,21861352	0,00049233	0,00408013
TRINITY_DN15207_c0_g1_i1	dmso	control	0,75785518	2,67887323	0,00510765	0,02740714
TRINITY_DN22594_c0_g1_i1	dmso	control	0,7569249	1,21433416	0,01702712	0,06996108
TRINITY_DN19377_c0_g1_i1	dmso	control	0,75636824	4,21807825	1,11E-05	0,00015436
TRINITY_DN11630_c0_g1_i1	dmso	control	0,75597946	5,35100962	2,43E-05	0,0003025
TRINITY_DN6657_c0_g1_i1	dmso	control	0,75465755	1,9685631	0,00747911	0,03696057
TRINITY_DN6246_c0_g1_i1	dmso	control	0,75432609	4,57308077	1,70E-06	2,83E-05
TRINITY_DN8451_c0_g1_i1	dmso	control	0,7540999	3,77333663	0,00194308	0,0127523

TRINITY_DN13756_c0_g1_i1	dmso	control	0,75396405	1,76780421	0,01355077	0,0588864
TRINITY_DN37220_c0_g1_i1	dmso	control	0,7537703	2,65384847	0,0068914	0,03461275
TRINITY_DN20544_c0_g1_i1	dmso	control	0,75307432	3,38005375	0,00165116	0,01120291
TRINITY_DN6408_c0_g1_i1	dmso	control	0,7514384	5,62335983	1,92E-06	3,15E-05
TRINITY_DN20677_c0_g1_i1	dmso	control	0,75060416	3,77404051	0,00088942	0,00670895
TRINITY_DN6117_c0_g1_i1	dmso	control	0,75038248	5,04207988	2,01E-07	4,15E-06
TRINITY_DN344_c0_g1_i1	dmso	control	0,75004228	6,61399762	8,39E-07	1,49E-05
TRINITY_DN19752_c0_g1_i1	dmso	control	0,74968712	2,00445942	0,01145264	0,05171411
TRINITY_DN2018_c0_g1_i1	dmso	control	0,74960075	5,04071885	3,82E-06	5,80E-05
TRINITY_DN6939_c0_g1_i1	dmso	control	0,74950227	5,49380025	2,09E-08	5,19E-07
TRINITY_DN12268_c0_g1_i1	dmso	control	0,74858816	6,67047073	3,45E-10	1,17E-08
TRINITY_DN4681_c0_g1_i1	dmso	control	0,74744303	2,94540481	0,00072279	0,00565584
TRINITY_DN5714_c0_g1_i1	dmso	control	0,74730192	3,35108362	0,00048251	0,00400833
TRINITY_DN15966_c0_g1_i1	dmso	control	0,74518398	2,83941139	0,00129605	0,00923146
TRINITY_DN15868_c0_g1_i1	dmso	control	0,74435713	1,56813139	0,00952907	0,04473473
TRINITY_DN2472_c0_g1_i1	dmso	control	0,74375031	3,52629717	0,00016186	0,00157688
TRINITY_DN3539_c0_g1_i1	dmso	control	0,74368774	2,02469532	0,01550618	0,0649802
TRINITY_DN19605_c0_g1_i1	dmso	control	0,74236861	3,12434625	0,00032839	0,00290486
TRINITY_DN7498_c0_g1_i1	dmso	control	0,74202476	3,63665687	0,00011836	0,00120831
TRINITY_DN6602_c0_g1_i1	dmso	control	0,74151679	3,27329943	0,00043991	0,00370307
TRINITY_DN497_c0_g1_i1	dmso	control	0,74073956	8,68759832	3,84E-10	1,29E-08
TRINITY_DN20715_c0_g1_i1	dmso	control	0,74045723	3,25275797	0,00617515	0,03184578
TRINITY_DN3240_c0_g1_i1	dmso	control	0,7397399	5,05187366	2,76E-07	5,48E-06
TRINITY_DN9954_c0_g1_i1	dmso	control	0,73958002	1,82515548	0,00627237	0,03222061
TRINITY_DN7484_c0_g1_i1	dmso	control	0,73853018	3,73438832	6,51E-05	0,00072486
TRINITY_DN7595_c0_g1_i1	dmso	control	0,73845225	3,19442891	0,00133938	0,00946079
TRINITY_DN7423_c0_g1_i1	dmso	control	0,73824679	3,84533787	5,25E-05	0,00059821
TRINITY_DN22576_c0_g1_i1	dmso	control	0,73690821	3,83875099	8,21E-05	0,00088093
TRINITY_DN9597_c0_g1_i1	dmso	control	0,7360494	1,82010063	0,0066812	0,03380385
TRINITY_DN17083_c0_g1_i1	dmso	control	0,73589956	3,59355282	0,00048255	0,00400833
TRINITY_DN17781_c0_g1_i1	dmso	control	0,73568376	1,80235344	0,01108017	0,05034699

TRINITY_DN18909_c0_g1_i1	dmso	control	0,73442382	4,44729918	1,25E-05	0,00017129
TRINITY_DN20794_c0_g1_i1	dmso	control	0,73427796	1,78214152	0,01367988	0,05919889
TRINITY_DN7050_c0_g1_i1	dmso	control	0,7337389	4,3139045	0,00216974	0,01396695
TRINITY_DN6076_c0_g1_i1	dmso	control	0,73311704	6,06118255	1,66E-08	4,19E-07
TRINITY_DN12170_c0_g1_i1	dmso	control	0,73261426	4,38842528	3,73E-05	0,00044395
TRINITY_DN23575_c0_g1_i1	dmso	control	0,73260254	1,51457035	0,01621012	0,06744102
TRINITY_DN6901_c0_g1_i1	dmso	control	0,73235044	4,4979473	1,40E-05	0,00018896
TRINITY_DN19362_c0_g1_i1	dmso	control	0,73233847	6,31732381	2,17E-09	6,48E-08
TRINITY_DN2585_c0_g1_i1	dmso	control	0,73210546	4,93000292	0,00392327	0,02235568
TRINITY_DN32869_c0_g1_i1	dmso	control	0,73166395	3,69533151	0,00014969	0,00147699
TRINITY_DN6596_c0_g1_i1	dmso	control	0,73099286	3,33747267	0,00498998	0,02690567
TRINITY_DN9407_c0_g1_i1	dmso	control	0,72862911	3,27484057	0,0006425	0,0051304
TRINITY_DN20112_c0_g1_i1	dmso	control	0,72748371	2,07311515	0,00512849	0,02747813
TRINITY_DN5223_c0_g1_i1	dmso	control	0,72742411	6,38843548	5,94E-10	1,94E-08
TRINITY_DN26871_c0_g1_i1	dmso	control	0,72696903	2,01116701	0,02107322	0,08236001
TRINITY_DN45881_c0_g1_i1	dmso	control	0,72574758	1,34355239	0,01849022	0,0746065
TRINITY_DN10850_c0_g1_i1	dmso	control	0,72548504	4,96837053	1,56E-05	0,00020691
TRINITY_DN12431_c0_g1_i1	dmso	control	0,72521934	4,61293083	1,66E-05	0,00021823
TRINITY_DN8877_c0_g1_i1	dmso	control	0,72498337	2,75057311	0,00110299	0,00805088
TRINITY_DN4854_c0_g1_i1	dmso	control	0,72424119	6,63050842	1,56E-08	3,95E-07
TRINITY_DN1870_c0_g1_i1	dmso	control	0,7236956	1,7474624	0,01275861	0,05636028
TRINITY_DN7086_c0_g1_i1	dmso	control	0,72306763	3,61122643	0,00079795	0,0061474
TRINITY_DN2218_c0_g1_i1	dmso	control	0,72284346	3,36330141	0,00129423	0,00922307
TRINITY_DN21098_c0_g1_i1	dmso	control	0,7223612	2,52791938	0,00435648	0,02422226
TRINITY_DN12726_c0_g1_i1	dmso	control	0,7218084	4,36574197	4,54E-05	0,00052689
TRINITY_DN13914_c0_g1_i1	dmso	control	0,7216684	2,10828944	0,01095342	0,04995964
TRINITY_DN34611_c0_g1_i1	dmso	control	0,72166155	3,6159856	0,00014627	0,00144713
TRINITY_DN5878_c0_g1_i1	dmso	control	0,72138796	4,07736937	0,00065694	0,00522835
TRINITY_DN2053_c0_g1_i1	dmso	control	0,72113572	5,84048301	1,21E-08	3,16E-07
TRINITY_DN17794_c0_g1_i1	dmso	control	0,72070166	3,67763851	0,00453803	0,02510604
TRINITY_DN12165_c0_g1_i1	dmso	control	0,72030337	2,18376274	0,00501392	0,02698437

TRINITY_DN28264_c0_g1_i1	dmso	control	0,71926564	3,41263122	0,00035369	0,00308521
TRINITY_DN6429_c0_g1_i1	dmso	control	0,71922189	4,422518	7,12E-05	0,00078168
TRINITY_DN19271_c0_g1_i1	dmso	control	0,71914679	3,20533283	0,00218765	0,0140572
TRINITY_DN31674_c0_g1_i1	dmso	control	0,71881222	2,48949889	0,00455293	0,02515954
TRINITY_DN21851_c0_g1_i1	dmso	control	0,71850242	3,9425144	0,00010811	0,0011171
TRINITY_DN990_c0_g1_i1	dmso	control	0,71789247	5,3988646	7,37E-05	0,00080608
TRINITY_DN17105_c0_g1_i1	dmso	control	0,71700588	2,54320012	0,00706769	0,03537521
TRINITY_DN11736_c0_g1_i1	dmso	control	0,71665489	3,51865386	0,00120385	0,00866019
TRINITY_DN763_c0_g1_i1	dmso	control	0,71634872	7,21287543	1,34E-10	4,86E-09
TRINITY_DN13606_c0_g1_i1	dmso	control	0,71625305	2,37442723	0,00581714	0,03040107
TRINITY_DN9473_c0_g1_i1	dmso	control	0,71622151	3,59238874	0,00018916	0,00179728
TRINITY_DN12840_c0_g1_i1	dmso	control	0,71612708	3,66685371	0,00018925	0,00179728
TRINITY_DN428_c0_g1_i1	dmso	control	0,71547292	8,87051623	1,60E-10	5,75E-09
TRINITY_DN11548_c0_g1_i1	dmso	control	0,71547217	3,09367542	0,00565053	0,02971311
TRINITY_DN11282_c0_g1_i1	dmso	control	0,71467146	4,64846307	0,0002029	0,00190685
TRINITY_DN5618_c0_g1_i1	dmso	control	0,71401163	6,65513725	3,34E-09	9,46E-08
TRINITY_DN6666_c0_g1_i1	dmso	control	0,71361815	5,64831015	2,38E-06	3,81E-05
TRINITY_DN16708_c0_g1_i1	dmso	control	0,71337173	1,91442898	0,00768921	0,03780486
TRINITY_DN1157_c0_g1_i1	dmso	control	0,71326918	4,92886922	0,00070505	0,00553201
TRINITY_DN6327_c0_g1_i1	dmso	control	0,71251475	2,3804637	0,00658491	0,03346907
TRINITY_DN6700_c0_g1_i1	dmso	control	0,71186784	2,08294126	0,01081393	0,04947963
TRINITY_DN4264_c0_g1_i1	dmso	control	0,70981196	6,45290941	1,10E-08	2,89E-07
TRINITY_DN13867_c0_g1_i1	dmso	control	0,70976275	2,69360162	0,00233646	0,01477676
TRINITY_DN18924_c0_g1_i1	dmso	control	0,70974319	5,81354862	7,41E-08	1,68E-06
TRINITY_DN6538_c0_g1_i1	dmso	control	0,7094168	4,00962948	7,54E-05	0,00082024
TRINITY_DN6287_c0_g1_i1	dmso	control	0,70925542	3,60518363	0,00024255	0,00222595
TRINITY_DN4144_c0_g1_i1	dmso	control	0,70873919	2,66052767	0,00529567	0,02822727
TRINITY_DN32840_c0_g1_i1	dmso	control	0,70830719	3,52291226	0,00049276	0,00408139
TRINITY_DN11015_c0_g1_i1	dmso	control	0,70794432	9,504789	2,63E-11	1,06E-09
TRINITY_DN18927_c0_g1_i1	dmso	control	0,70790103	4,96045665	1,62E-06	2,71E-05
TRINITY_DN12774_c0_g1_i1	dmso	control	0,70757915	4,5215183	1,59E-05	0,00020933

TRINITY_DN16418_c0_g1_i1	dmso	control	0,70739316	2,4822582	0,0051688	0,02766331
TRINITY_DN12382_c0_g1_i1	dmso	control	0,70670476	2,76182928	0,00584235	0,03047374
TRINITY_DN9288_c0_g1_i1	dmso	control	0,7062027	3,96480202	6,34E-05	0,00070805
TRINITY_DN9075_c0_g1_i1	dmso	control	0,70563421	2,32786888	0,00574684	0,03012086
TRINITY_DN1242_c0_g1_i1	dmso	control	0,70547064	8,01236785	8,36E-10	2,67E-08
TRINITY_DN15800_c0_g1_i1	dmso	control	0,70485251	2,81879871	0,00231032	0,0146307
TRINITY_DN23_c0_g1_i1	dmso	control	0,70473618	5,18644844	0,00015138	0,00148959
TRINITY_DN14535_c0_g1_i1	dmso	control	0,70465193	3,51624846	0,01633307	0,06775221
TRINITY_DN11855_c0_g1_i1	dmso	control	0,70455563	3,30688141	0,00161716	0,01104489
TRINITY_DN25495_c0_g1_i1	dmso	control	0,70437374	2,28164834	0,01146432	0,05172348
TRINITY_DN6787_c0_g1_i1	dmso	control	0,70302542	4,33198758	0,00092453	0,0069089
TRINITY_DN1162_c0_g1_i1	dmso	control	0,70085081	3,50888017	0,02067301	0,08119101
TRINITY_DN11936_c0_g1_i1	dmso	control	0,70006184	4,30580399	7,38E-05	0,00080608
TRINITY_DN8738_c0_g1_i1	dmso	control	0,69991689	1,74407032	0,01175122	0,05267793
TRINITY_DN13826_c0_g1_i1	dmso	control	0,69966419	3,21260984	0,00128465	0,00917288
TRINITY_DN15397_c0_g1_i1	dmso	control	0,69887435	3,01470513	0,00176718	0,01186746
TRINITY_DN34567_c0_g1_i1	dmso	control	0,69870743	2,06400256	0,00878182	0,04192116
TRINITY_DN11129_c0_g1_i1	dmso	control	0,69865451	3,83301486	0,00059218	0,00477612
TRINITY_DN13845_c0_g1_i1	dmso	control	0,69862023	3,34541569	0,0022428	0,01431606
TRINITY_DN2972_c0_g1_i1	dmso	control	0,69821651	4,17357627	6,22E-05	0,00069702
TRINITY_DN6460_c0_g1_i1	dmso	control	0,69749932	3,72853761	0,00017451	0,00167826
TRINITY_DN9009_c0_g1_i1	dmso	control	0,69718267	3,72447869	0,00394353	0,02244456
TRINITY_DN20302_c0_g1_i1	dmso	control	0,69701899	4,1290227	0,00057967	0,00469882
TRINITY_DN6541_c0_g1_i1	dmso	control	0,69681785	4,62009217	0,00040905	0,00348396
TRINITY_DN5563_c0_g1_i1	dmso	control	0,69660505	4,27970083	3,17E-05	0,00038395
TRINITY_DN13472_c0_g1_i1	dmso	control	0,69561758	5,39519047	9,22E-07	1,63E-05
TRINITY_DN12697_c0_g1_i1	dmso	control	0,69503405	5,8007964	6,76E-07	1,23E-05
TRINITY_DN18949_c0_g1_i1	dmso	control	0,69371366	4,89113018	5,30E-06	7,86E-05
TRINITY_DN29890_c0_g1_i1	dmso	control	0,69299241	3,81829449	0,0003723	0,00321642
TRINITY_DN5248_c0_g1_i1	dmso	control	0,69226633	6,91067569	1,43E-08	3,68E-07
TRINITY_DN22370_c0_g1_i1	dmso	control	0,6907973	1,89376947	0,01451962	0,06166888

TRINITY_DN11741_c0_g1_i1	dmso	control	0,6900833	5,91097472	2,64E-07	5,30E-06
TRINITY_DN2204_c0_g1_i1	dmso	control	0,68996144	6,11743524	2,19E-06	3,53E-05
TRINITY_DN6608_c0_g1_i1	dmso	control	0,68988357	3,55386431	0,00123574	0,00885871
TRINITY_DN3186_c0_g1_i1	dmso	control	0,68980036	3,11628061	0,01004356	0,04659897
TRINITY_DN15802_c0_g1_i1	dmso	control	0,68926135	2,34797676	0,01987013	0,07885644
TRINITY_DN6514_c0_g1_i1	dmso	control	0,68898153	2,51819255	0,0036274	0,02105173
TRINITY_DN3146_c0_g1_i1	dmso	control	0,688327	2,88867321	0,00158152	0,0108322
TRINITY_DN7502_c0_g1_i1	dmso	control	0,68765553	4,48940331	0,00052396	0,00432
TRINITY_DN7151_c0_g1_i1	dmso	control	0,68703624	7,16783174	8,42E-06	0,00011959
TRINITY_DN6126_c0_g1_i1	dmso	control	0,68652926	6,29748656	1,31E-07	2,85E-06
TRINITY_DN2993_c0_g1_i1	dmso	control	0,68649683	3,28656469	0,00063411	0,00507464
TRINITY_DN684_c0_g1_i1	dmso	control	0,68628314	9,54675072	3,24E-10	1,10E-08
TRINITY_DN6036_c0_g1_i1	dmso	control	0,68543741	3,91185764	0,00011283	0,00116005
TRINITY_DN15839_c0_g1_i1	dmso	control	0,68465441	4,36824083	0,00068511	0,0054075
TRINITY_DN11521_c0_g1_i1	dmso	control	0,68455006	3,32478532	0,00052685	0,00433638
TRINITY_DN37138_c0_g1_i1	dmso	control	0,68312283	1,86226417	0,01634543	0,06775221
TRINITY_DN46182_c0_g1_i1	dmso	control	0,68203271	1,38782053	0,02410314	0,09100101
TRINITY_DN13632_c0_g1_i1	dmso	control	0,68193226	2,34958681	0,01432314	0,061099
TRINITY_DN11491_c0_g1_i1	dmso	control	0,68185538	6,97217046	2,31E-06	3,71E-05
TRINITY_DN5313_c0_g1_i1	dmso	control	0,68120527	7,05476282	4,13E-09	1,15E-07
TRINITY_DN19126_c0_g1_i1	dmso	control	0,68050059	4,45165491	1,95E-05	0,00024976
TRINITY_DN7237_c0_g1_i1	dmso	control	0,68046275	2,38563971	0,00543605	0,02883533
TRINITY_DN5824_c0_g1_i1	dmso	control	0,67988805	7,74051289	2,65E-07	5,31E-06
TRINITY_DN23013_c0_g1_i1	dmso	control	0,67953223	3,08983434	0,0013472	0,00951139
TRINITY_DN2641_c0_g1_i1	dmso	control	0,67789984	1,68022682	0,02424554	0,09134763
TRINITY_DN7245_c0_g1_i1	dmso	control	0,67770285	3,38658072	0,00281313	0,01717581
TRINITY_DN308_c0_g1_i1	dmso	control	0,67766489	5,05674005	3,16E-05	0,00038348
TRINITY_DN19620_c0_g1_i1	dmso	control	0,67747515	2,87339146	0,00468204	0,02567654
TRINITY_DN13537_c0_g1_i1	dmso	control	0,67707298	3,88690484	0,00021715	0,00202107
TRINITY_DN2631_c0_g1_i1	dmso	control	0,67684292	3,85319165	0,00055646	0,00453361
TRINITY_DN14631_c0_g1_i1	dmso	control	0,67602464	2,58169238	0,0065729	0,03341975

TRINITY_DN23735_c0_g1_i1	dmso	control	0,67596129	1,87914433	0,01316073	0,05759063
TRINITY_DN8654_c0_g1_i1	dmso	control	0,6757765	6,04077098	2,19E-07	4,46E-06
TRINITY_DN1652_c0_g1_i1	dmso	control	0,67542483	7,75495248	1,18E-09	3,65E-08
TRINITY_DN5736_c0_g1_i1	dmso	control	0,6752002	5,89712951	4,56E-07	8,64E-06
TRINITY_DN5136_c0_g1_i1	dmso	control	0,67344922	5,92243306	1,08E-07	2,39E-06
TRINITY_DN20388_c0_g1_i1	dmso	control	0,67286066	2,4833042	0,01512139	0,06373751
TRINITY_DN17028_c0_g1_i1	dmso	control	0,67276452	2,14130599	0,01795209	0,07290356
TRINITY_DN1029_c0_g1_i1	dmso	control	0,67229746	3,57474887	0,00050312	0,00415527
TRINITY_DN2219_c0_g1_i1	dmso	control	0,67198734	2,10637233	0,00915712	0,04328355
TRINITY_DN5882_c0_g1_i1	dmso	control	0,67158159	6,97681337	1,85E-05	0,00023881
TRINITY_DN11059_c0_g1_i1	dmso	control	0,67055499	5,07908226	2,67E-05	0,00033032
TRINITY_DN6471_c0_g1_i1	dmso	control	0,6705291	4,60709537	3,93E-05	0,00046397
TRINITY_DN7210_c0_g1_i1	dmso	control	0,67019332	3,07078758	0,02167962	0,08411554
TRINITY_DN5014_c0_g1_i1	dmso	control	0,66905443	4,98969134	1,82E-05	0,00023628
TRINITY_DN15222_c0_g1_i1	dmso	control	0,66830302	2,54736889	0,00926719	0,04373232
TRINITY_DN2781_c0_g1_i1	dmso	control	0,66788406	4,77497561	0,00034071	0,00299547
TRINITY_DN720_c0_g1_i1	dmso	control	0,66755566	8,54996353	9,53E-10	2,99E-08
TRINITY_DN9149_c0_g1_i1	dmso	control	0,66701945	3,9036644	0,00043986	0,00370307
TRINITY_DN11739_c0_g1_i1	dmso	control	0,66652903	3,2131286	0,00161889	0,01104624
TRINITY_DN11701_c0_g1_i1	dmso	control	0,66566974	5,64185895	7,73E-06	0,00011093
TRINITY_DN19979_c0_g1_i1	dmso	control	0,66480868	4,17582458	0,00018824	0,00179046
TRINITY_DN3067_c0_g1_i1	dmso	control	0,66319968	5,01017469	0,00469098	0,02570605
TRINITY_DN12707_c0_g1_i1	dmso	control	0,66218906	3,53165135	0,01551365	0,06499266
TRINITY_DN20870_c0_g1_i1	dmso	control	0,66214965	2,63679738	0,00414048	0,02327207
TRINITY_DN6035_c0_g1_i1	dmso	control	0,66197489	6,9981789	7,01E-08	1,60E-06
TRINITY_DN19484_c0_g1_i1	dmso	control	0,66124736	2,89923639	0,00244675	0,0153681
TRINITY_DN7938_c0_g1_i1	dmso	control	0,66086283	2,27849693	0,01371774	0,05927394
TRINITY_DN13216_c0_g1_i1	dmso	control	0,66045937	4,47100313	6,85E-05	0,00075571
TRINITY_DN3428_c0_g1_i1	dmso	control	0,65997679	4,52870104	0,00023259	0,00214957
TRINITY_DN10959_c0_g1_i1	dmso	control	0,65988136	6,09510699	2,36E-06	3,79E-05
TRINITY_DN12405_c0_g1_i1	dmso	control	0,65964071	3,98426526	0,00310545	0,01862885

TRINITY_DN19856_c0_g1_i1	dmso	control	0,6594433	4,03392488	0,00081536	0,00625487
TRINITY_DN32726_c0_g1_i1	dmso	control	0,65905303	6,27975625	3,80E-08	8,97E-07
TRINITY_DN2441_c0_g1_i1	dmso	control	0,65870841	2,64526105	0,01767691	0,07213079
TRINITY_DN6519_c0_g1_i1	dmso	control	0,6572566	3,08670729	0,0019799	0,01295859
TRINITY_DN11116_c0_g1_i1	dmso	control	0,6570016	8,46218244	3,92E-09	1,10E-07
TRINITY_DN12797_c0_g1_i1	dmso	control	0,65681794	2,13230362	0,02115475	0,08263396
TRINITY_DN20303_c0_g1_i1	dmso	control	0,65542311	1,76415531	0,02413657	0,0910796
TRINITY_DN5611_c0_g1_i1	dmso	control	0,65479811	6,75168362	1,58E-07	3,34E-06
TRINITY_DN6743_c0_g1_i1	dmso	control	0,65479232	6,4995076	1,33E-07	2,88E-06
TRINITY_DN21922_c0_g1_i1	dmso	control	0,65381476	2,11680906	0,01026115	0,04739987
TRINITY_DN12194_c0_g1_i1	dmso	control	0,65330243	4,60751168	3,15E-05	0,00038194
TRINITY_DN2260_c0_g1_i1	dmso	control	0,65250925	3,22704002	0,00338625	0,01990819
TRINITY_DN10997_c0_g1_i1	dmso	control	0,6521663	6,0407218	0,00017979	0,00172216
TRINITY_DN20364_c0_g1_i1	dmso	control	0,6520968	1,7670711	0,02284659	0,08741697
TRINITY_DN1823_c0_g1_i1	dmso	control	0,65192239	3,95137199	0,00066363	0,00527584
TRINITY_DN5058_c0_g1_i1	dmso	control	0,65155833	6,24624222	8,36E-06	0,00011888
TRINITY_DN2760_c0_g1_i1	dmso	control	0,65147074	3,71656809	0,00061781	0,00495276
TRINITY_DN1791_c0_g1_i1	dmso	control	0,64910589	4,16499217	0,00583453	0,0304589
TRINITY_DN20202_c0_g1_i1	dmso	control	0,64855195	3,72746646	0,0010034	0,00741007
TRINITY_DN26019_c0_g1_i1	dmso	control	0,64822813	2,96270069	0,02185266	0,08465048
TRINITY_DN23769_c0_g1_i1	dmso	control	0,64785791	2,77232399	0,00505128	0,02713491
TRINITY_DN12377_c0_g1_i1	dmso	control	0,64768503	2,11489817	0,01492333	0,06312618
TRINITY_DN75603_c0_g1_i1	dmso	control	0,64674234	2,10674326	0,01503212	0,06349094
TRINITY_DN23518_c0_g1_i1	dmso	control	0,64665582	1,75809476	0,01949138	0,0776698
TRINITY_DN5927_c0_g1_i1	dmso	control	0,6460739	4,29752149	0,00108366	0,00792986
TRINITY_DN6347_c0_g1_i1	dmso	control	0,64518372	4,43569158	0,0002939	0,00263687
TRINITY_DN4673_c0_g1_i1	dmso	control	0,64517709	5,64079171	0,00099415	0,0073493
TRINITY_DN77083_c0_g1_i1	dmso	control	0,64444482	2,65725991	0,00528076	0,0281686
TRINITY_DN7403_c0_g1_i1	dmso	control	0,64419655	4,97983938	6,01E-05	0,00067501
TRINITY_DN5011_c0_g1_i1	dmso	control	0,64414103	6,61220609	7,23E-08	1,64E-06
TRINITY_DN10949_c0_g1_i1	dmso	control	0,64407176	6,26672811	1,44E-05	0,00019344

TRINITY_DN20412_c0_g1_i1	dmso	control	0,64365848	8,54615421	7,05E-08	1,61E-06
TRINITY_DN8315_c0_g1_i1	dmso	control	0,64356604	6,23124109	7,21E-06	0,00010415
TRINITY_DN3034_c0_g1_i1	dmso	control	0,64243173	7,35007792	1,45E-08	3,71E-07
TRINITY_DN23467_c0_g1_i1	dmso	control	0,64242528	4,37019269	0,00017014	0,00164282
TRINITY_DN19394_c0_g1_i1	dmso	control	0,64233302	7,35001815	8,18E-07	1,46E-05
TRINITY_DN2465_c0_g1_i1	dmso	control	0,64208034	3,66218929	0,00149765	0,01036087
TRINITY_DN4774_c0_g1_i1	dmso	control	0,64187948	9,40436785	2,92E-09	8,38E-08
TRINITY_DN19824_c0_g1_i1	dmso	control	0,6413845	8,07013338	8,42E-08	1,89E-06
TRINITY_DN936_c0_g1_i1	dmso	control	0,64122361	6,26230076	3,59E-06	5,49E-05
TRINITY_DN2340_c0_g1_i1	dmso	control	0,63935822	4,81055803	3,77E-05	0,00044738
TRINITY_DN7319_c0_g1_i1	dmso	control	0,63870749	4,6354174	8,67E-05	0,00092235
TRINITY_DN5594_c0_g1_i1	dmso	control	0,63857614	6,62964831	1,10E-07	2,43E-06
TRINITY_DN13535_c0_g1_i1	dmso	control	0,63810621	5,05149511	1,80E-05	0,0002337
TRINITY_DN10799_c0_g1_i1	dmso	control	0,63783226	7,98970249	1,53E-08	3,90E-07
TRINITY_DN14974_c0_g1_i1	dmso	control	0,63688639	2,9007284	0,00530794	0,02826141
TRINITY_DN3233_c0_g1_i1	dmso	control	0,63614936	3,37043206	0,00355134	0,0207102
TRINITY_DN19589_c0_g1_i1	dmso	control	0,63568708	4,39415011	0,00018019	0,00172487
TRINITY_DN30406_c0_g1_i1	dmso	control	0,63557503	2,63789153	0,00611934	0,03159178
TRINITY_DN29790_c0_g1_i1	dmso	control	0,63543013	3,22321874	0,00199738	0,0130553
TRINITY_DN2928_c0_g1_i1	dmso	control	0,63541076	3,69129587	0,00090242	0,00677521
TRINITY_DN14672_c0_g1_i1	dmso	control	0,63495091	7,35623846	6,32E-07	1,16E-05
TRINITY_DN5742_c0_g1_i1	dmso	control	0,63442877	5,42184083	1,46E-05	0,00019612
TRINITY_DN13211_c0_g1_i1	dmso	control	0,63413846	3,95446633	0,00036084	0,00313237
TRINITY_DN7722_c0_g1_i1	dmso	control	0,63393302	2,30788044	0,0109412	0,04991964
TRINITY_DN6524_c0_g1_i1	dmso	control	0,633925	3,02368766	0,02121868	0,08279411
TRINITY_DN6053_c0_g1_i1	dmso	control	0,63352012	6,01804443	3,10E-07	6,08E-06
TRINITY_DN789_c0_g1_i1	dmso	control	0,63268103	3,47607902	0,01958962	0,07799672
TRINITY_DN2847_c0_g1_i1	dmso	control	0,63259234	2,40474877	0,01447541	0,06156534
TRINITY_DN26357_c0_g1_i1	dmso	control	0,63241851	2,25155342	0,0194035	0,07738364
TRINITY_DN26009_c0_g1_i1	dmso	control	0,63239266	3,74500223	0,0025506	0,01586029
TRINITY_DN2534_c0_g1_i1	dmso	control	0,63219943	3,24264978	0,00628083	0,03224939

TRINITY_DN22329_c0_g1_i1	dmso	control	0,63208518	5,36737983	6,90E-05	0,00076015
TRINITY_DN5571_c0_g1_i1	dmso	control	0,63152604	4,9052682	0,0001228	0,00124744
TRINITY_DN20294_c0_g1_i1	dmso	control	0,63083148	4,90939118	0,00484445	0,02631744
TRINITY_DN11580_c0_g1_i1	dmso	control	0,63078474	6,13388867	5,79E-07	1,07E-05
TRINITY_DN2024_c0_g1_i1	dmso	control	0,63045135	4,30661379	0,00038573	0,00331263
TRINITY_DN27388_c0_g1_i1	dmso	control	0,63008299	2,77966728	0,02133289	0,08308265
TRINITY_DN8939_c0_g1_i1	dmso	control	0,62955078	1,80513245	0,02564207	0,09527535
TRINITY_DN20999_c0_g1_i1	dmso	control	0,62953734	2,45489659	0,01001671	0,04654815
TRINITY_DN12090_c0_g1_i1	dmso	control	0,62947552	5,64558396	1,41E-06	2,41E-05
TRINITY_DN8138_c0_g1_i1	dmso	control	0,62909438	4,07927417	0,00075971	0,00589998
TRINITY_DN11213_c0_g1_i1	dmso	control	0,62897686	7,85624381	1,07E-07	2,37E-06
TRINITY_DN10294_c0_g1_i1	dmso	control	0,62802454	2,67144014	0,00634985	0,03251456
TRINITY_DN1219_c0_g1_i1	dmso	control	0,62746181	7,06697756	1,56E-07	3,31E-06
TRINITY_DN10217_c0_g1_i1	dmso	control	0,62674708	3,51721053	0,01502344	0,06347283
TRINITY_DN7084_c0_g1_i1	dmso	control	0,62642134	4,35037966	0,00016312	0,00158246
TRINITY_DN8382_c0_g1_i1	dmso	control	0,62601144	3,56774406	0,00167433	0,01134412
TRINITY_DN2209_c0_g1_i1	dmso	control	0,62358028	6,00272055	7,73E-07	1,39E-05
TRINITY_DN7649_c0_g1_i1	dmso	control	0,62358011	5,16048439	0,00047449	0,00395501
TRINITY_DN896_c0_g1_i1	dmso	control	0,62347745	8,01004404	1,18E-08	3,11E-07
TRINITY_DN29213_c0_g1_i1	dmso	control	0,62326377	5,83018753	1,94E-06	3,17E-05
TRINITY_DN14914_c0_g1_i1	dmso	control	0,62306738	3,81205798	0,00200533	0,0131013
TRINITY_DN26851_c0_g1_i1	dmso	control	0,62277217	4,18016345	0,00024602	0,00225208
TRINITY_DN66001_c0_g1_i1	dmso	control	0,62195911	2,12626726	0,01714924	0,07037521
TRINITY_DN2557_c0_g1_i1	dmso	control	0,62189084	3,04981861	0,00460654	0,02536848
TRINITY_DN13755_c0_g1_i1	dmso	control	0,62187486	8,52422468	9,48E-07	1,66E-05
TRINITY_DN3295_c0_g1_i1	dmso	control	0,62184792	4,22009308	0,00031457	0,0027963
TRINITY_DN30677_c0_g1_i1	dmso	control	0,62142861	2,1684706	0,02301057	0,08797403
TRINITY_DN11332_c0_g1_i1	dmso	control	0,62107334	4,62324197	0,00838006	0,04049846
TRINITY_DN14638_c0_g1_i1	dmso	control	0,62072565	2,73269673	0,0057725	0,03020055
TRINITY_DN14216_c0_g1_i1	dmso	control	0,6199981	2,7903561	0,00477573	0,02603514
TRINITY_DN3525_c0_g1_i1	dmso	control	0,61974189	3,21989989	0,00225819	0,0143825

TRINITY_DN2565_c0_g1_i1	dmso	control	0,61961672	2,37377446	0,01190998	0,05329606
TRINITY_DN19421_c0_g1_i1	dmso	control	0,61956488	6,10224281	3,59E-07	6,95E-06
TRINITY_DN20031_c0_g1_i1	dmso	control	0,61918862	2,64389529	0,01396912	0,06000607
TRINITY_DN13246_c0_g1_i1	dmso	control	0,6191442	4,21133523	0,00972882	0,04547986
TRINITY_DN35609_c0_g1_i1	dmso	control	0,61830817	2,30418891	0,01331212	0,05811228
TRINITY_DN12893_c0_g1_i1	dmso	control	0,61808567	3,73900864	0,00100881	0,00744625
TRINITY_DN4672_c0_g1_i1	dmso	control	0,61768883	3,99753851	0,00172538	0,01163544
TRINITY_DN6655_c0_g1_i1	dmso	control	0,61679164	4,43171943	0,00011585	0,00118769
TRINITY_DN21524_c0_g1_i1	dmso	control	0,61647851	2,56312011	0,0177019	0,07218042
TRINITY_DN13253_c0_g1_i1	dmso	control	0,6162133	3,86546703	0,00144178	0,01004173
TRINITY_DN435_c0_g1_i1	dmso	control	0,61605546	6,30462971	4,09E-05	0,00047958
TRINITY_DN1968_c0_g1_i1	dmso	control	0,61571435	3,10235869	0,00353919	0,02065599
TRINITY_DN4230_c0_g1_i1	dmso	control	0,61459708	5,06962724	1,83E-05	0,00023706
TRINITY_DN1883_c0_g1_i1	dmso	control	0,61431135	3,6425649	0,00459904	0,02533684
TRINITY_DN1450_c0_g1_i1	dmso	control	0,61393683	4,47424026	0,00060999	0,00490064
TRINITY_DN366_c0_g1_i1	dmso	control	0,61283307	7,58614694	2,72E-07	5,42E-06
TRINITY_DN33613_c0_g1_i1	dmso	control	0,61224455	2,81862873	0,00777754	0,03810944
TRINITY_DN2386_c0_g1_i1	dmso	control	0,61203173	5,87501866	2,68E-06	4,27E-05
TRINITY_DN6949_c0_g1_i1	dmso	control	0,61201289	5,16392413	7,59E-05	0,00082533
TRINITY_DN6459_c0_g1_i1	dmso	control	0,61187426	2,97491039	0,00642851	0,03283587
TRINITY_DN992_c0_g1_i1	dmso	control	0,61149453	6,23809236	3,13E-05	0,00038055
TRINITY_DN6224_c0_g1_i1	dmso	control	0,61136821	3,00830645	0,00672933	0,0339878
TRINITY_DN1698_c0_g1_i1	dmso	control	0,61119569	8,12050279	1,11E-07	2,45E-06
TRINITY_DN98_c0_g1_i1	dmso	control	0,61084293	7,51524081	9,79E-08	2,18E-06
TRINITY_DN7201_c0_g1_i1	dmso	control	0,61043492	4,68446103	9,49E-05	0,00100014
TRINITY_DN11633_c0_g1_i1	dmso	control	0,60983711	3,68448952	0,00138927	0,00974172
TRINITY_DN21352_c0_g1_i1	dmso	control	0,60934186	3,16059136	0,00456143	0,02519689
TRINITY_DN3090_c0_g1_i1	dmso	control	0,60927198	5,94192051	0,00010454	0,00108563
TRINITY_DN2247_c0_g1_i1	dmso	control	0,60718276	5,26769934	6,16E-05	0,00068991
TRINITY_DN3279_c0_g1_i1	dmso	control	0,60713311	3,7534089	0,00289793	0,01758112
TRINITY_DN2481_c0_g1_i1	dmso	control	0,60705846	5,10510381	0,00373542	0,02151442

TRINITY_DN6386_c0_g1_i1	dmso	control	0,60669442	3,48830389	0,00329417	0,01953378
TRINITY_DN8928_c0_g1_i1	dmso	control	0,60629534	6,40225592	2,12E-07	4,33E-06
TRINITY_DN25023_c0_g1_i1	dmso	control	0,60563915	2,13190411	0,02125853	0,08290484
TRINITY_DN21022_c0_g1_i1	dmso	control	0,60533054	3,60891689	0,00239947	0,01512229
TRINITY_DN6823_c0_g1_i1	dmso	control	0,605185	5,47899324	6,45E-06	9,40E-05
TRINITY_DN35382_c0_g1_i1	dmso	control	0,60512127	3,0540113	0,00976925	0,04563933
TRINITY_DN10962_c0_g1_i1	dmso	control	0,60498789	3,58342819	0,00628244	0,03224939
TRINITY_DN1884_c0_g1_i1	dmso	control	0,60495673	3,30551994	0,00263481	0,01628576
TRINITY_DN5154_c0_g1_i1	dmso	control	0,60427396	8,46411438	2,00E-08	4,99E-07
TRINITY_DN2871_c0_g1_i1	dmso	control	0,60413864	5,59855197	8,15E-05	0,00087744
TRINITY_DN7287_c0_g1_i1	dmso	control	0,60379457	3,34702944	0,00376319	0,02163985
TRINITY_DN6437_c0_g1_i1	dmso	control	0,60265312	4,42159702	0,00019357	0,00183352
TRINITY_DN36723_c0_g1_i1	dmso	control	0,60257851	3,38275535	0,01544856	0,06479616
TRINITY_DN6779_c0_g1_i1	dmso	control	0,60252055	4,61461998	0,00196929	0,01290087
TRINITY_DN13969_c0_g1_i1	dmso	control	0,60249807	2,70035131	0,01413695	0,06052408
TRINITY_DN9330_c0_g1_i1	dmso	control	0,60217185	3,12311307	0,00595635	0,03093647
TRINITY_DN2274_c0_g1_i1	dmso	control	0,60205788	5,81628544	5,92E-05	0,00066601
TRINITY_DN23639_c0_g1_i1	dmso	control	0,60155198	4,33861564	0,0166921	0,06891038
TRINITY_DN1609_c0_g1_i1	dmso	control	0,60047813	4,57009708	0,00055464	0,00452765
TRINITY_DN9672_c0_g1_i1	dmso	control	0,60027395	4,22768447	0,00091029	0,00682366
TRINITY_DN11128_c0_g1_i1	dmso	control	0,60023125	3,96367777	0,00237386	0,01499363
TRINITY_DN15446_c0_g1_i1	dmso	control	0,59946063	4,8712245	0,00012426	0,00125939
TRINITY_DN1621_c0_g1_i1	dmso	control	0,59935594	3,70693048	0,00167751	0,01136035
TRINITY_DN1740_c0_g1_i1	dmso	control	0,59751766	5,35716603	2,16E-05	0,00027357
TRINITY_DN11325_c0_g1_i1	dmso	control	0,59725255	3,38522366	0,00476395	0,02600101
TRINITY_DN14780_c0_g1_i1	dmso	control	0,59708247	3,30983663	0,01241602	0,05518444
TRINITY_DN18698_c0_g1_i1	dmso	control	0,59702452	5,08936997	4,97E-05	0,00057139
TRINITY_DN4542_c0_g1_i1	dmso	control	0,59651668	4,46875098	0,00019943	0,00187918
TRINITY_DN5558_c0_g1_i1	dmso	control	0,59651092	4,70797341	0,00058609	0,00474024
TRINITY_DN4765_c0_g1_i1	dmso	control	0,59614815	7,38158616	2,04E-07	4,20E-06
TRINITY_DN20286_c0_g1_i1	dmso	control	0,59613898	3,43026056	0,01253218	0,05559808

TRINITY_DN20808_c0_g1_i1	dmso	control	0,59542944	2,12599867	0,02692033	0,09889731
TRINITY_DN1161_c0_g1_i1	dmso	control	0,59506738	6,21801284	1,61E-06	2,71E-05
TRINITY_DN6232_c0_g1_i1	dmso	control	0,59426679	3,95788352	0,01192142	0,0533181
TRINITY_DN3616_c0_g1_i1	dmso	control	0,59323542	3,84410184	0,00144841	0,01007814
TRINITY_DN10803_c0_g1_i1	dmso	control	0,59319874	6,34660213	2,33E-05	0,00029273
TRINITY_DN8386_c0_g1_i1	dmso	control	0,59303536	2,22906283	0,02611423	0,09662398
TRINITY_DN27658_c0_g1_i1	dmso	control	0,5926689	8,56326863	7,06E-08	1,61E-06
TRINITY_DN11938_c0_g1_i1	dmso	control	0,59260888	4,89556204	0,00033444	0,00294751
TRINITY_DN9575_c0_g1_i1	dmso	control	0,59079467	4,27439817	0,00148209	0,01028281
TRINITY_DN1333_c0_g1_i1	dmso	control	0,59071003	4,8315477	0,00010731	0,00111115
TRINITY_DN1264_c0_g1_i1	dmso	control	0,58934549	5,82911492	5,57E-06	8,22E-05
TRINITY_DN3218_c0_g1_i1	dmso	control	0,58851241	3,73123639	0,00640884	0,03274698
TRINITY_DN1719_c0_g1_i1	dmso	control	0,58846883	5,01643959	0,0001252	0,00126647
TRINITY_DN2722_c0_g1_i1	dmso	control	0,58817236	2,732685	0,01301384	0,05712089
TRINITY_DN11606_c0_g1_i1	dmso	control	0,58754156	4,21317034	0,00227685	0,0144695
TRINITY_DN40052_c0_g1_i1	dmso	control	0,58751102	2,55993824	0,01411374	0,06046055
TRINITY_DN858_c0_g1_i1	dmso	control	0,58721846	6,90013905	8,31E-06	0,00011823
TRINITY_DN19179_c0_g1_i1	dmso	control	0,58667944	5,77436931	3,33E-06	5,14E-05
TRINITY_DN417_c0_g1_i1	dmso	control	0,58640543	6,06209484	0,00541411	0,02874196
TRINITY_DN26937_c0_g1_i1	dmso	control	0,58613362	5,64136893	0,00011658	0,00119431
TRINITY_DN74361_c0_g1_i1	dmso	control	0,58526059	3,43302866	0,01460272	0,06196719
TRINITY_DN1038_c0_g1_i1	dmso	control	0,58427917	5,91553775	2,37E-05	0,00029686
TRINITY_DN20916_c0_g1_i1	dmso	control	0,58414144	3,28771793	0,00991528	0,04617222
TRINITY_DN2519_c0_g1_i1	dmso	control	0,58393731	5,58475078	0,0011799	0,00852182
TRINITY_DN6562_c0_g1_i1	dmso	control	0,58385813	3,40843177	0,00749355	0,03701926
TRINITY_DN24302_c0_g1_i1	dmso	control	0,58175471	2,33240946	0,02586233	0,09591305
TRINITY_DN1646_c0_g1_i1	dmso	control	0,5813168	6,96231524	0,00250731	0,01567882
TRINITY_DN12858_c0_g1_i1	dmso	control	0,58052988	2,82173831	0,00863326	0,04137606
TRINITY_DN19938_c0_g1_i1	dmso	control	0,5797471	2,327626	0,02028431	0,0800565
TRINITY_DN4814_c0_g1_i1	dmso	control	0,5797105	6,5821598	6,40E-06	9,34E-05
TRINITY_DN19248_c0_g1_i1	dmso	control	0,57914952	5,07836318	0,00064201	0,00512935

TRINITY_DN9883_c0_g1_i1	dmso	control	0,57880495	2,94026388	0,01123236	0,05092636
TRINITY_DN19288_c0_g1_i1	dmso	control	0,57853812	4,17408869	0,00800795	0,039076
TRINITY_DN14429_c0_g1_i1	dmso	control	0,57657063	3,66480202	0,01001473	0,04654815
TRINITY_DN23409_c0_g1_i1	dmso	control	0,57619165	2,73850678	0,01875073	0,07537265
TRINITY_DN23572_c0_g1_i1	dmso	control	0,57614748	5,2306966	0,00053612	0,00440265
TRINITY_DN8709_c0_g1_i1	dmso	control	0,57565571	3,90584307	0,01182641	0,05293847
TRINITY_DN23915_c0_g1_i1	dmso	control	0,57528145	3,24771526	0,00406486	0,02293628
TRINITY_DN9138_c0_g1_i1	dmso	control	0,57463018	2,29470433	0,01983059	0,07878116
TRINITY_DN3516_c0_g1_i1	dmso	control	0,57422892	2,39701	0,02564421	0,09527535
TRINITY_DN7418_c0_g1_i1	dmso	control	0,57398354	2,5204952	0,02079644	0,08149881
TRINITY_DN13032_c0_g1_i1	dmso	control	0,57379407	3,46859118	0,00453451	0,02509615
TRINITY_DN8239_c0_g1_i1	dmso	control	0,57358564	3,61822013	0,00221994	0,01420779
TRINITY_DN7098_c0_g1_i1	dmso	control	0,57349452	3,2043502	0,0045765	0,02526083
TRINITY_DN41421_c0_g1_i1	dmso	control	0,57328256	3,47230961	0,00322991	0,01925552
TRINITY_DN624_c0_g1_i1	dmso	control	0,57292087	5,81519647	3,22E-05	0,00038842
TRINITY_DN1960_c0_g1_i1	dmso	control	0,57235226	3,67095527	0,00220034	0,01410811
TRINITY_DN5477_c0_g1_i1	dmso	control	0,57232276	4,9160845	0,00014341	0,00142377
TRINITY_DN13422_c0_g1_i1	dmso	control	0,5721925	4,8816158	0,00016189	0,00157688
TRINITY_DN33764_c0_g1_i1	dmso	control	0,57122668	3,27181509	0,00504103	0,02709999
TRINITY_DN15862_c0_g1_i1	dmso	control	0,57097414	6,49606592	7,97E-05	0,00086057
TRINITY_DN1387_c0_g1_i1	dmso	control	0,56985774	4,66789802	0,00025207	0,00230599
TRINITY_DN1479_c0_g1_i1	dmso	control	0,56975073	5,2259476	0,00163399	0,01110726
TRINITY_DN4909_c0_g1_i1	dmso	control	0,56955891	5,61898794	0,00185706	0,0123278
TRINITY_DN19772_c0_g1_i1	dmso	control	0,56950456	2,71924841	0,01268038	0,05613489
TRINITY_DN19843_c0_g1_i1	dmso	control	0,56936777	2,15043202	0,0252925	0,09433261
TRINITY_DN19519_c0_g1_i1	dmso	control	0,56934226	3,86259387	0,00296971	0,01794362
TRINITY_DN14315_c0_g1_i1	dmso	control	0,56928839	3,17577154	0,00982243	0,04585813
TRINITY_DN2031_c0_g1_i1	dmso	control	0,56908293	5,85906611	6,50E-06	9,45E-05
TRINITY_DN8400_c0_g1_i1	dmso	control	0,56778131	3,80732609	0,00193748	0,01273291
TRINITY_DN1394_c0_g1_i1	dmso	control	0,56749806	4,3668682	0,00882548	0,04210174
TRINITY_DN1720_c0_g1_i1	dmso	control	0,56717577	3,84802676	0,00238341	0,01504736

TRINITY_DN41_c0_g1_i1	dmso	control	0,56685612	5,84255992	1,28E-05	0,00017455
TRINITY_DN18720_c0_g1_i1	dmso	control	0,56679869	6,39483871	4,78E-05	0,00055117
TRINITY_DN11809_c0_g1_i1	dmso	control	0,56661187	4,69649567	0,00020486	0,00192157
TRINITY_DN14129_c0_g1_i1	dmso	control	0,56639692	4,12028786	0,01538882	0,06465723
TRINITY_DN3094_c0_g1_i1	dmso	control	0,56611547	4,83918644	0,00017178	0,00165535
TRINITY_DN11717_c0_g1_i1	dmso	control	0,56589052	2,74168611	0,02033882	0,08018403
TRINITY_DN22715_c0_g1_i1	dmso	control	0,56537565	2,75148775	0,01875315	0,07537265
TRINITY_DN17140_c0_g1_i1	dmso	control	0,56535827	2,17750999	0,02432608	0,0915317
TRINITY_DN6529_c0_g1_i1	dmso	control	0,56509108	4,9232672	0,00266682	0,01645545
TRINITY_DN2078_c0_g1_i1	dmso	control	0,56485646	3,55187281	0,00384481	0,02199533
TRINITY_DN5784_c0_g1_i1	dmso	control	0,56475581	4,6948478	0,00053991	0,00442124
TRINITY_DN8092_c0_g1_i1	dmso	control	0,56443746	5,36731727	6,39E-05	0,00071283
TRINITY_DN20864_c0_g1_i1	dmso	control	0,56389722	2,96057705	0,0148534	0,06290159
TRINITY_DN22921_c0_g1_i1	dmso	control	0,56349872	3,65594529	0,01256473	0,05569116
TRINITY_DN12825_c0_g1_i1	dmso	control	0,56346113	3,3720976	0,00418507	0,02345883
TRINITY_DN1066_c0_g1_i1	dmso	control	0,5633914	6,19623501	6,48E-06	9,42E-05
TRINITY_DN3983_c0_g1_i1	dmso	control	0,56305622	3,97437861	0,00374064	0,02153591
TRINITY_DN14820_c0_g1_i1	dmso	control	0,562934	2,93843877	0,0177398	0,07224456
TRINITY_DN12608_c0_g1_i1	dmso	control	0,56233835	3,03183644	0,01334896	0,05820275
TRINITY_DN11681_c0_g1_i1	dmso	control	0,56199341	5,01595038	0,00028164	0,00253953
TRINITY_DN9513_c0_g1_i1	dmso	control	0,56127777	4,84645084	0,00149025	0,01031465
TRINITY_DN760_c0_g1_i1	dmso	control	0,56087924	7,11548932	2,99E-06	4,66E-05
TRINITY_DN8113_c0_g1_i1	dmso	control	0,56045868	2,22770259	0,02365261	0,08981651
TRINITY_DN7626_c0_g1_i1	dmso	control	0,55934691	5,57510572	6,24E-05	0,00069805
TRINITY_DN5085_c0_g1_i1	dmso	control	0,55924178	5,56550415	2,82E-05	0,00034619
TRINITY_DN23765_c0_g1_i1	dmso	control	0,55900648	3,26963219	0,0077013	0,03782566
TRINITY_DN6857_c0_g1_i1	dmso	control	0,55876543	6,49232301	1,26E-05	0,00017228
TRINITY_DN21043_c0_g1_i1	dmso	control	0,55706094	3,53615088	0,00372352	0,021463
TRINITY_DN37572_c0_g1_i1	dmso	control	0,55661685	3,16631412	0,00660546	0,03350411
TRINITY_DN8896_c0_g1_i1	dmso	control	0,55564904	3,46539449	0,00401356	0,02274454
TRINITY_DN34259_c0_g1_i1	dmso	control	0,55499294	3,90365485	0,00493774	0,02671428

TRINITY_DN1511_c0_g1_i1	dmso	control	0,55422271	6,31729248	2,78E-06	4,39E-05
TRINITY_DN19180_c0_g1_i1	dmso	control	0,55392023	4,6012487	0,00060201	0,00484725
TRINITY_DN2718_c0_g1_i1	dmso	control	0,55364754	3,79180637	0,00337368	0,01985728
TRINITY_DN36227_c0_g1_i1	dmso	control	0,5532125	2,9210692	0,02189948	0,08474095
TRINITY_DN2667_c0_g1_i1	dmso	control	0,55318542	4,26369537	0,002253	0,01436215
TRINITY_DN1340_c0_g1_i1	dmso	control	0,55310224	5,94362272	2,24E-05	0,00028181
TRINITY_DN8224_c0_g1_i1	dmso	control	0,55306794	5,32710677	5,60E-05	0,00063497
TRINITY_DN9026_c0_g1_i1	dmso	control	0,55279621	4,35287657	0,00192355	0,01267593
TRINITY_DN11976_c0_g1_i1	dmso	control	0,55096505	4,02048802	0,0017993	0,01203279
TRINITY_DN17213_c0_g1_i1	dmso	control	0,55091152	5,25027373	0,0001012	0,00105543
TRINITY_DN19881_c0_g1_i1	dmso	control	0,55078424	3,26399218	0,01294213	0,05695908
TRINITY_DN1009_c0_g1_i1	dmso	control	0,55070054	7,10219671	5,99E-06	8,78E-05
TRINITY_DN534_c0_g1_i1	dmso	control	0,55067911	6,45445995	0,00020113	0,00189394
TRINITY_DN34173_c0_g1_i1	dmso	control	0,55025851	4,81133449	0,00119159	0,00858478
TRINITY_DN34613_c0_g1_i1	dmso	control	0,55022104	4,12358169	0,01992413	0,07902363
TRINITY_DN20643_c0_g1_i1	dmso	control	0,54989651	2,52778907	0,02075545	0,08136025
TRINITY_DN6209_c0_g1_i1	dmso	control	0,54982216	5,64442506	2,55E-05	0,00031698
TRINITY_DN2528_c0_g1_i1	dmso	control	0,54961633	3,51340808	0,00447509	0,02479583
TRINITY_DN6309_c0_g1_i1	dmso	control	0,54917163	5,31385668	0,00023396	0,00216086
TRINITY_DN7452_c0_g1_i1	dmso	control	0,5485939	3,19737535	0,0068872	0,03460368
TRINITY_DN20212_c0_g1_i1	dmso	control	0,54855875	2,87455626	0,01987901	0,07886633
TRINITY_DN4413_c0_g1_i1	dmso	control	0,54845301	3,15929266	0,00747367	0,03694631
TRINITY_DN1654_c0_g1_i1	dmso	control	0,54773052	6,61953115	2,92E-05	0,00035658
TRINITY_DN10542_c0_g1_i1	dmso	control	0,54765626	3,88275487	0,01333046	0,05815721
TRINITY_DN21059_c0_g1_i1	dmso	control	0,54729976	3,14007549	0,0069622	0,03489557
TRINITY_DN5835_c0_g1_i1	dmso	control	0,54728457	5,09927044	0,00162444	0,01106329
TRINITY_DN78572_c0_g1_i1	dmso	control	0,54719809	4,30910045	0,00151029	0,01043335
TRINITY_DN16493_c0_g1_i1	dmso	control	0,54690269	3,41367951	0,00482696	0,02625633
TRINITY_DN5463_c0_g1_i1	dmso	control	0,54644277	4,82194807	0,0019814	0,01296255
TRINITY_DN407_c0_g1_i1	dmso	control	0,54643904	5,02491081	0,02279995	0,08726689
TRINITY_DN7865_c0_g1_i1	dmso	control	0,5457918	5,09243475	0,00014005	0,00139522

TRINITY_DN4441_c0_g1_i1	dmso	control	0,54573065	3,7378315	0,00940631	0,04425866
TRINITY_DN1693_c0_g1_i1	dmso	control	0,54553507	4,00842578	0,00673401	0,03399955
TRINITY_DN2798_c0_g1_i1	dmso	control	0,54540034	5,65825723	0,0001622	0,00157778
TRINITY_DN5051_c0_g1_i1	dmso	control	0,54512151	5,703795	0,00909917	0,04303328
TRINITY_DN6684_c0_g1_i1	dmso	control	0,54500309	5,67290548	0,00047392	0,0039527
TRINITY_DN16013_c0_g1_i1	dmso	control	0,54435422	4,53277583	0,00065108	0,00518742
TRINITY_DN7167_c0_g1_i1	dmso	control	0,54429533	4,2095257	0,00533409	0,02837971
TRINITY_DN21239_c0_g1_i1	dmso	control	0,54418176	4,7061509	0,00414527	0,02328995
TRINITY_DN2813_c0_g1_i1	dmso	control	0,54388196	8,59915831	5,37E-07	9,98E-06
TRINITY_DN3098_c0_g1_i1	dmso	control	0,54362863	3,28383934	0,02413321	0,0910796
TRINITY_DN2014_c0_g1_i1	dmso	control	0,54291969	3,48787266	0,01417603	0,06065544
TRINITY_DN406_c0_g1_i1	dmso	control	0,54287303	7,28807654	4,14E-05	0,00048502
TRINITY_DN3275_c0_g1_i1	dmso	control	0,54262875	6,93990595	2,05E-06	3,33E-05
TRINITY_DN20485_c0_g1_i1	dmso	control	0,54249162	3,64179205	0,00406141	0,02292579
TRINITY_DN6031_c0_g1_i1	dmso	control	0,54227507	4,83115699	0,00036517	0,00316054
TRINITY_DN12708_c0_g1_i1	dmso	control	0,54216576	3,14530709	0,01044355	0,04805896
TRINITY_DN338_c0_g1_i1	dmso	control	0,54179622	8,7236038	2,05E-06	3,32E-05
TRINITY_DN6517_c0_g1_i1	dmso	control	0,54148973	7,87363539	7,90E-07	1,42E-05
TRINITY_DN5655_c0_g1_i1	dmso	control	0,54118376	4,80416619	0,00101788	0,00750934
TRINITY_DN18868_c0_g1_i1	dmso	control	0,54071661	3,7614006	0,00357747	0,02081946
TRINITY_DN6671_c0_g1_i1	dmso	control	0,54008081	8,31847528	4,72E-07	8,90E-06
TRINITY_DN1106_c0_g1_i1	dmso	control	0,53930723	7,16111405	2,11E-06	3,42E-05
TRINITY_DN19682_c0_g1_i1	dmso	control	0,53872365	4,76780676	0,00113443	0,008247
TRINITY_DN11601_c0_g1_i1	dmso	control	0,53835939	5,36302888	0,01371363	0,0592739
TRINITY_DN6204_c0_g1_i1	dmso	control	0,53820509	4,26104641	0,00304881	0,01835765
TRINITY_DN11154_c0_g1_i1	dmso	control	0,53800392	4,95286144	0,00035362	0,00308521
TRINITY_DN4657_c0_g1_i1	dmso	control	0,53784254	7,87362023	6,25E-06	9,13E-05
TRINITY_DN2373_c0_g1_i1	dmso	control	0,53763355	5,26737362	0,00045825	0,00384847
TRINITY_DN20498_c0_g1_i1	dmso	control	0,53710099	3,26950718	0,0071894	0,03583537
TRINITY_DN3308_c0_g1_i1	dmso	control	0,5370848	4,64688559	0,0013193	0,00935098
TRINITY_DN8783_c0_g1_i1	dmso	control	0,53704273	3,36026494	0,00636908	0,03258992

TRINITY_DN577_c0_g1_i1	dmso	control	0,53604908	6,98839994	1,78E-06	2,96E-05
TRINITY_DN8941_c0_g1_i1	dmso	control	0,5355148	2,94032708	0,02193552	0,0847951
TRINITY_DN153_c0_g1_i1	dmso	control	0,53540093	6,1079395	0,00016631	0,00161018
TRINITY_DN5491_c0_g1_i1	dmso	control	0,53532836	4,98744497	0,0013186	0,00935062
TRINITY_DN21091_c0_g1_i1	dmso	control	0,53471663	3,78714197	0,00456636	0,02521449
TRINITY_DN2687_c0_g1_i1	dmso	control	0,53459055	4,73776973	0,00071089	0,00557176
TRINITY_DN6116_c0_g1_i1	dmso	control	0,53457781	6,19974958	0,00131178	0,00931593
TRINITY_DN2192_c0_g1_i1	dmso	control	0,53406673	5,01267002	0,00029981	0,00268323
TRINITY_DN6553_c0_g1_i1	dmso	control	0,53369863	3,62067348	0,00459708	0,02533568
TRINITY_DN5230_c0_g1_i1	dmso	control	0,53328518	5,72469044	7,63E-05	0,00082843
TRINITY_DN10836_c0_g1_i1	dmso	control	0,53291801	6,98081499	0,00047144	0,00394323
TRINITY_DN11515_c0_g1_i1	dmso	control	0,53275574	5,51886975	0,00015558	0,00152264
TRINITY_DN9284_c0_g1_i1	dmso	control	0,53250343	6,26207295	1,37E-05	0,00018445
TRINITY_DN2110_c0_g1_i1	dmso	control	0,53196588	5,22786368	0,00235308	0,01487537
TRINITY_DN13028_c0_g1_i1	dmso	control	0,5319374	3,76364288	0,00742518	0,03676952
TRINITY_DN20321_c0_g1_i1	dmso	control	0,53151865	3,69775159	0,00482997	0,02625852
TRINITY_DN4952_c0_g1_i1	dmso	control	0,53105937	4,8866067	0,0003617	0,00313425
TRINITY_DN3532_c0_g1_i1	dmso	control	0,53029405	3,20797687	0,01175362	0,05267793
TRINITY_DN39368_c0_g1_i1	dmso	control	0,53020467	3,02388997	0,01301358	0,05712089
TRINITY_DN13357_c0_g1_i1	dmso	control	0,52990027	3,0267706	0,01506582	0,06359608
TRINITY_DN1735_c0_g1_i1	dmso	control	0,52973282	4,4505156	0,020457	0,08054004
TRINITY_DN20917_c0_g1_i1	dmso	control	0,52967497	3,7674081	0,00552158	0,02918378
TRINITY_DN19434_c0_g1_i1	dmso	control	0,52942486	4,60515686	0,00464269	0,02549226
TRINITY_DN6307_c0_g1_i1	dmso	control	0,52942437	3,82180528	0,00446562	0,02477186
TRINITY_DN30588_c0_g1_i1	dmso	control	0,52939143	3,72398715	0,01041406	0,04793855
TRINITY_DN3136_c0_g1_i1	dmso	control	0,52914844	4,19989454	0,00198707	0,01299374
TRINITY_DN9175_c0_g1_i1	dmso	control	0,52895226	2,84141708	0,01689173	0,06953642
TRINITY_DN19379_c0_g1_i1	dmso	control	0,52823831	3,7281451	0,00609261	0,03151012
TRINITY_DN1039_c0_g1_i1	dmso	control	0,5281598	5,49372932	0,00226689	0,01442524
TRINITY_DN685_c0_g1_i1	dmso	control	0,52811676	6,13587693	0,00494263	0,02673016
TRINITY_DN3508_c0_g1_i1	dmso	control	0,52773138	4,85233083	0,0026868	0,01653454

TRINITY_DN20084_c0_g1_i1	dmso	control	0,52746975	3,54358299	0,00545609	0,0289223
TRINITY_DN6670_c0_g1_i1	dmso	control	0,52626863	4,7612323	0,00556268	0,02933657
TRINITY_DN4431_c0_g1_i1	dmso	control	0,5251847	5,14239177	0,00021341	0,00199145
TRINITY_DN5837_c0_g1_i1	dmso	control	0,52511631	4,69117267	0,00226276	0,0144053
TRINITY_DN5865_c0_g1_i1	dmso	control	0,52498107	5,61956243	4,18E-05	0,00049001
TRINITY_DN12291_c0_g1_i1	dmso	control	0,52369636	4,4732005	0,00530753	0,02826141
TRINITY_DN5446_c0_g1_i1	dmso	control	0,52368997	6,11317893	1,54E-05	0,00020484
TRINITY_DN6890_c0_g1_i1	dmso	control	0,52350615	5,26364598	0,00261595	0,01619694
TRINITY_DN7872_c0_g1_i1	dmso	control	0,52242806	4,80263177	0,00266275	0,01643738
TRINITY_DN3597_c0_g1_i1	dmso	control	0,52241964	4,27018891	0,00275065	0,01685038
TRINITY_DN3381_c0_g1_i1	dmso	control	0,5212603	5,19708769	0,00035578	0,00310153
TRINITY_DN10861_c0_g1_i1	dmso	control	0,52110191	7,10144926	6,20E-06	9,07E-05
TRINITY_DN2212_c0_g1_i1	dmso	control	0,51976686	4,72911639	0,00095363	0,007086
TRINITY_DN5535_c0_g1_i1	dmso	control	0,51935024	6,03836325	0,00121315	0,00871838
TRINITY_DN686_c0_g1_i1	dmso	control	0,51926412	5,42555528	0,00013736	0,00137315
TRINITY_DN9382_c0_g1_i1	dmso	control	0,51836628	3,5151847	0,01878233	0,07544792
TRINITY_DN6371_c0_g1_i1	dmso	control	0,51810312	4,48296398	0,00369365	0,02133339
TRINITY_DN5510_c0_g1_i1	dmso	control	0,51806444	5,03616233	0,0007669	0,0059399
TRINITY_DN5773_c0_g1_i1	dmso	control	0,51766195	5,03511729	0,00041904	0,00355639
TRINITY_DN2754_c0_g1_i1	dmso	control	0,51742243	4,96423272	0,00112935	0,00821423
TRINITY_DN6615_c0_g1_i1	dmso	control	0,51660751	3,64133742	0,01915746	0,07661392
TRINITY_DN15164_c0_g1_i1	dmso	control	0,5165485	3,52421812	0,00740673	0,03672851
TRINITY_DN8575_c0_g1_i1	dmso	control	0,5163073	3,65372867	0,00698892	0,03499303
TRINITY_DN6240_c0_g1_i1	dmso	control	0,51592508	4,38512073	0,01200603	0,05362619
TRINITY_DN13714_c0_g1_i1	dmso	control	0,51553459	2,89340737	0,01897151	0,07603869
TRINITY_DN2318_c0_g1_i1	dmso	control	0,51525153	2,97625622	0,02417393	0,09115284
TRINITY_DN5703_c0_g1_i1	dmso	control	0,51507825	5,75176236	0,00011681	0,00119585
TRINITY_DN21584_c0_g1_i1	dmso	control	0,51504491	4,54163219	0,00116481	0,00841701
TRINITY_DN2265_c0_g1_i1	dmso	control	0,51467593	4,70922352	0,00481656	0,02621525
TRINITY_DN2164_c0_g1_i1	dmso	control	0,51391415	4,98280914	0,00042884	0,00362891
TRINITY_DN1785_c0_g1_i1	dmso	control	0,51338951	3,97397572	0,01181412	0,05289986

TRINITY_DN4566_c0_g1_i1	dmso	control	0,51325453	5,57030642	0,00010208	0,00106315
TRINITY_DN19681_c0_g1_i1	dmso	control	0,51290855	5,49158809	0,00026209	0,0023826
TRINITY_DN34224_c0_g1_i1	dmso	control	0,51273595	5,1375833	0,00666999	0,03375891
TRINITY_DN1338_c0_g1_i1	dmso	control	0,51257166	6,37380759	1,12E-05	0,00015489
TRINITY_DN1743_c0_g1_i1	dmso	control	0,51214377	3,46366724	0,01296428	0,05700729
TRINITY_DN10611_c0_g1_i1	dmso	control	0,51211718	3,07399417	0,01353931	0,05887277
TRINITY_DN4683_c0_g1_i1	dmso	control	0,51209444	9,02833112	0,00038203	0,0032867
TRINITY_DN18094_c0_g1_i1	dmso	control	0,51200698	3,82079626	0,01701286	0,06993477
TRINITY_DN1098_c0_g1_i1	dmso	control	0,51177635	8,14574099	8,60E-05	0,00091654
TRINITY_DN2347_c0_g1_i1	dmso	control	0,51143171	3,05770028	0,01368906	0,05920548
TRINITY_DN5722_c0_g1_i1	dmso	control	0,51090632	6,30461327	9,73E-05	0,0010235
TRINITY_DN8054_c0_g1_i1	dmso	control	0,50950373	4,35130275	0,00470657	0,02577192
TRINITY_DN2427_c0_g1_i1	dmso	control	0,50905816	7,46553125	1,93E-05	0,00024889
TRINITY_DN15980_c0_g1_i1	dmso	control	0,50890602	3,58856569	0,00770393	0,03782571
TRINITY_DN6723_c0_g1_i1	dmso	control	0,50846311	5,01551647	0,00127767	0,00913211
TRINITY_DN11393_c0_g1_i1	dmso	control	0,5084534	4,33111524	0,00229826	0,01456067
TRINITY_DN1277_c0_g1_i1	dmso	control	0,5079948	3,1616306	0,02325564	0,08857235
TRINITY_DN19823_c0_g1_i1	dmso	control	0,50749017	3,88608146	0,00492456	0,02666242
TRINITY_DN13155_c0_g1_i1	dmso	control	0,50744506	3,66313423	0,01205962	0,05378242
TRINITY_DN4674_c0_g1_i1	dmso	control	0,50668586	6,06951885	0,00894317	0,04252276
TRINITY_DN12108_c0_g1_i1	dmso	control	0,50665102	3,96305785	0,00655368	0,03333375
TRINITY_DN21270_c0_g1_i1	dmso	control	0,50639485	3,75334799	0,01129954	0,05118276
TRINITY_DN19259_c0_g1_i1	dmso	control	0,50608447	4,63944749	0,00114861	0,00831665
TRINITY_DN21277_c0_g1_i1	dmso	control	0,5054313	2,76167673	0,02223348	0,08564338
TRINITY_DN5376_c0_g1_i1	dmso	control	0,50504916	5,65266505	8,24E-05	0,0008821
TRINITY_DN1491_c0_g1_i1	dmso	control	0,50391918	6,24739061	9,80E-05	0,00102831
TRINITY_DN2060_c0_g1_i1	dmso	control	0,50388885	4,26421741	0,00822065	0,03986148
TRINITY_DN1537_c0_g1_i1	dmso	control	0,5034482	5,31295269	0,00576635	0,03019874
TRINITY_DN10946_c0_g1_i1	dmso	control	0,50305072	6,29185292	0,00183142	0,0121968
TRINITY_DN12823_c0_g1_i1	dmso	control	0,50266689	5,05087172	0,00063963	0,005116
TRINITY_DN6631_c0_g1_i1	dmso	control	0,5026244	5,03242074	0,00473661	0,02587831

TRINITY_DN7459_c0_g1_i1	dmso	control	0,50257911	6,46934109	2,69E-05	0,00033287
TRINITY_DN7009_c0_g1_i1	dmso	control	0,50217345	5,35460241	0,00192112	0,01266574
TRINITY_DN13369_c0_g1_i1	dmso	control	0,50193274	6,86227887	1,20E-05	0,000166
TRINITY_DN2089_c0_g1_i1	dmso	control	0,50105601	4,62012781	0,00313976	0,01882682
TRINITY_DN9249_c0_g1_i1	dmso	control	0,50081908	4,32358023	0,00386652	0,02208922
TRINITY_DN6358_c0_g1_i1	dmso	control	0,50023988	3,74040245	0,00646116	0,03295601
TRINITY_DN1929_c0_g1_i1	dmso	control	0,500177	3,94109754	0,00554242	0,02926181
TRINITY_DN5583_c0_g1_i1	dmso	control	0,49981092	4,6429155	0,00562668	0,02960923
TRINITY_DN22912_c0_g1_i1	dmso	control	0,49955207	7,94649532	0,0041218	0,02318512
TRINITY_DN635_c0_g1_i1	dmso	control	0,49942131	3,16527778	0,02680993	0,09857086
TRINITY_DN566_c0_g1_i1	dmso	control	0,49935843	6,72401593	2,11E-05	0,00026886
TRINITY_DN1371_c0_g1_i1	dmso	control	0,49922666	6,53210108	0,00012984	0,00130604
TRINITY_DN1249_c0_g1_i1	dmso	control	0,49919285	5,51869705	0,00037849	0,00326402
TRINITY_DN11343_c0_g1_i1	dmso	control	0,49906223	4,88880319	0,00163043	0,01109353
TRINITY_DN7444_c0_g1_i1	dmso	control	0,49871457	4,05639894	0,00868185	0,04154911
TRINITY_DN11692_c0_g1_i1	dmso	control	0,49855475	5,34530074	0,00056861	0,00462477
TRINITY_DN12975_c0_g1_i1	dmso	control	0,49851741	3,92287209	0,00496282	0,02680924
TRINITY_DN12884_c0_g1_i1	dmso	control	0,49810532	3,27986288	0,0125532	0,05565716
TRINITY_DN13326_c0_g1_i1	dmso	control	0,49804483	5,66165801	0,00024028	0,00220793
TRINITY_DN11261_c0_g1_i1	dmso	control	0,49784114	5,08781419	0,00179391	0,01200236
TRINITY_DN47808_c0_g1_i1	dmso	control	0,49769465	3,96490895	0,00495118	0,02676636
TRINITY_DN6330_c0_g1_i1	dmso	control	0,49636069	4,47562088	0,01573764	0,06579745
TRINITY_DN962_c0_g1_i1	dmso	control	0,49603096	5,11027352	0,00958187	0,04492378
TRINITY_DN3159_c0_g1_i1	dmso	control	0,49599274	3,21445156	0,01424247	0,06084961
TRINITY_DN205_c0_g1_i1	dmso	control	0,49573082	5,6598125	0,00018139	0,00173296
TRINITY_DN6751_c0_g1_i1	dmso	control	0,49551103	3,58440348	0,00959095	0,04495176
TRINITY_DN2899_c0_g1_i1	dmso	control	0,4945907	5,71908945	0,00144323	0,010047
TRINITY_DN10780_c0_g1_i1	dmso	control	0,49434151	8,55052187	0,0003434	0,00301057
TRINITY_DN22106_c0_g1_i1	dmso	control	0,49404347	5,02041415	0,00533686	0,028384
TRINITY_DN12395_c0_g1_i1	dmso	control	0,4928418	4,40811054	0,01306032	0,05727272
TRINITY_DN14382_c0_g1_i1	dmso	control	0,49251211	3,30045538	0,02074479	0,08134051

TRINITY_DN7744_c0_g1_i1	dmso	control	0,49229937	3,96171902	0,00805693	0,03922561
TRINITY_DN1956_c0_g1_i1	dmso	control	0,49116631	4,56463471	0,00153896	0,01060098
TRINITY_DN12218_c0_g1_i1	dmso	control	0,49106664	3,40937453	0,01107507	0,05034699
TRINITY_DN11099_c0_g1_i1	dmso	control	0,49083417	5,61441554	0,00015246	0,00149715
TRINITY_DN5763_c0_g1_i1	dmso	control	0,48943114	4,83349951	0,003101	0,01862533
TRINITY_DN14753_c0_g1_i1	dmso	control	0,4892872	6,15460333	0,00019499	0,00184573
TRINITY_DN3594_c0_g1_i1	dmso	control	0,48832649	2,99829387	0,02426613	0,09135372
TRINITY_DN1707_c0_g1_i1	dmso	control	0,48830799	6,37492973	0,00647646	0,03300652
TRINITY_DN1578_c0_g1_i1	dmso	control	0,48808517	6,92942591	0,00014926	0,00147372
TRINITY_DN12987_c0_g1_i1	dmso	control	0,48769821	4,05568633	0,01046021	0,04808967
TRINITY_DN20380_c0_g1_i1	dmso	control	0,4865866	5,55201024	0,00025236	0,00230722
TRINITY_DN2741_c0_g1_i1	dmso	control	0,48650649	3,5554958	0,01950353	0,07769676
TRINITY_DN6603_c0_g1_i1	dmso	control	0,48572079	4,96279025	0,00647791	0,03300652
TRINITY_DN14128_c0_g1_i1	dmso	control	0,48559876	3,6932453	0,00948347	0,04457816
TRINITY_DN13563_c0_g1_i1	dmso	control	0,48534381	3,8138332	0,01521203	0,06406351
TRINITY_DN2062_c0_g1_i1	dmso	control	0,48479846	6,07401746	0,0002268	0,00210415
TRINITY_DN18638_c0_g1_i1	dmso	control	0,48462526	3,32069224	0,02487463	0,09318308
TRINITY_DN9179_c0_g1_i1	dmso	control	0,48452891	3,68182211	0,01318117	0,05766264
TRINITY_DN12867_c0_g1_i1	dmso	control	0,48395167	3,80851216	0,00877876	0,04192037
TRINITY_DN3549_c0_g1_i1	dmso	control	0,4833551	4,87267419	0,00136057	0,00958702
TRINITY_DN43130_c0_g1_i1	dmso	control	0,48284904	4,69643189	0,01543804	0,06478865
TRINITY_DN11789_c0_g1_i1	dmso	control	0,48233128	5,33515846	0,001288	0,00918774
TRINITY_DN1406_c0_g1_i1	dmso	control	0,48185453	4,03072013	0,0058437	0,03047374
TRINITY_DN7583_c0_g1_i1	dmso	control	0,4812482	4,89947753	0,00203379	0,01325127
TRINITY_DN1481_c0_g1_i1	dmso	control	0,48079036	5,38709234	0,00078684	0,0060845
TRINITY_DN426_c0_g1_i1	dmso	control	0,48075175	8,06318796	1,68E-05	0,00021944
TRINITY_DN1256_c0_g1_i1	dmso	control	0,48004215	6,64016887	3,71E-05	0,00044168
TRINITY_DN3494_c0_g1_i1	dmso	control	0,47985186	4,05184831	0,00586725	0,03058193
TRINITY_DN4851_c0_g1_i1	dmso	control	0,47981244	4,86993157	0,00131171	0,00931593
TRINITY_DN12394_c0_g1_i1	dmso	control	0,47927729	5,66787823	0,00092645	0,00691608
TRINITY_DN54638_c0_g1_i1	dmso	control	0,47861579	4,18519717	0,00563578	0,02964633

TRINITY_DN7494_c0_g1_i1	dmso	control	0,47855407	5,76940471	0,01440184	0,06130744
TRINITY_DN35063_c0_g1_i1	dmso	control	0,47839897	3,49433815	0,02600912	0,09633377
TRINITY_DN5942_c0_g1_i1	dmso	control	0,47779883	5,4474914	0,00047009	0,00393423
TRINITY_DN13120_c0_g1_i1	dmso	control	0,4772983	3,5994243	0,01177173	0,05272638
TRINITY_DN5437_c0_g1_i1	dmso	control	0,47720019	8,97473777	0,00046986	0,00393423
TRINITY_DN5918_c0_g1_i1	dmso	control	0,47691936	5,57205941	0,00038267	0,00329025
TRINITY_DN19596_c0_g1_i1	dmso	control	0,4767351	3,36257598	0,02305682	0,08808669
TRINITY_DN20418_c0_g1_i1	dmso	control	0,47645996	5,1494466	0,01177092	0,05272638
TRINITY_DN679_c0_g1_i1	dmso	control	0,47644789	6,58476072	5,25E-05	0,00059805
TRINITY_DN21083_c0_g1_i1	dmso	control	0,4763671	3,75900267	0,01137738	0,05143858
TRINITY_DN5972_c0_g1_i1	dmso	control	0,47541788	4,80455462	0,00190198	0,01257799
TRINITY_DN11502_c0_g1_i1	dmso	control	0,47520314	5,09907201	0,00083295	0,00637623
TRINITY_DN2148_c0_g1_i1	dmso	control	0,4748765	5,36371922	0,00048884	0,00405592
TRINITY_DN12321_c0_g1_i1	dmso	control	0,47466165	4,2550513	0,0133658	0,0582586
TRINITY_DN818_c0_g1_i1	dmso	control	0,47464179	4,34332986	0,00405136	0,02287799
TRINITY_DN37533_c0_g1_i1	dmso	control	0,47392459	4,82720682	0,00174611	0,01174234
TRINITY_DN12834_c0_g1_i1	dmso	control	0,47383161	3,10005119	0,02610344	0,0966088
TRINITY_DN20641_c0_g1_i1	dmso	control	0,4734274	3,00982168	0,02417491	0,09115284
TRINITY_DN300_c0_g1_i1	dmso	control	0,47321261	4,70247652	0,00317958	0,01901038
TRINITY_DN6627_c0_g1_i1	dmso	control	0,47282426	4,26390189	0,00497806	0,02688155
TRINITY_DN894_c0_g1_i1	dmso	control	0,4728237	5,85323046	0,00037129	0,00321151
TRINITY_DN8399_c0_g1_i1	dmso	control	0,4727875	4,92974548	0,00239001	0,01508243
TRINITY_DN13877_c0_g1_i1	dmso	control	0,47270758	4,34030459	0,00365711	0,02116463
TRINITY_DN2304_c0_g1_i1	dmso	control	0,47250416	4,2878295	0,00502787	0,02704937
TRINITY_DN1770_c0_g1_i1	dmso	control	0,47173215	9,18741293	1,06E-05	0,00014689
TRINITY_DN10323_c0_g1_i1	dmso	control	0,4716344	3,9058526	0,01097394	0,05000985
TRINITY_DN13599_c0_g1_i1	dmso	control	0,47079955	4,69994996	0,00336748	0,01983012
TRINITY_DN11030_c0_g1_i1	dmso	control	0,47065842	8,30751636	2,01E-05	0,00025767
TRINITY_DN12114_c0_g1_i1	dmso	control	0,46935666	3,78894262	0,02641018	0,09741957
TRINITY_DN26645_c0_g1_i1	dmso	control	0,46925975	4,01750241	0,01421194	0,06075508
TRINITY_DN5848_c0_g1_i1	dmso	control	0,46919102	5,28313812	0,00185212	0,01231196

TRINITY_DN5472_c0_g1_i1	dmso	control	0,46885874	6,9425923	0,00010258	0,00106679
TRINITY_DN540_c0_g1_i1	dmso	control	0,46882734	4,16647968	0,00604863	0,03135001
TRINITY_DN3765_c0_g1_i1	dmso	control	0,46871627	5,06681179	0,00107037	0,00786848
TRINITY_DN20638_c0_g1_i1	dmso	control	0,46871106	4,62043077	0,00267152	0,01647744
TRINITY_DN372_c0_g1_i1	dmso	control	0,46801517	5,19945269	0,00325027	0,01934493
TRINITY_DN21155_c0_g1_i1	dmso	control	0,46794493	5,03573181	0,00669323	0,03384232
TRINITY_DN19598_c0_g1_i1	dmso	control	0,46771896	5,75825245	0,00336422	0,01981901
TRINITY_DN6139_c0_g1_i1	dmso	control	0,46758557	5,31458793	0,0022934	0,0145554
TRINITY_DN11885_c0_g1_i1	dmso	control	0,46750425	4,72523534	0,00224234	0,01431606
TRINITY_DN1525_c0_g1_i1	dmso	control	0,46705907	3,7861453	0,01312619	0,05750918
TRINITY_DN430_c0_g1_i1	dmso	control	0,46702537	5,15855599	0,00209695	0,01356187
TRINITY_DN594_c0_g1_i1	dmso	control	0,46607284	8,08083054	0,00083137	0,00636749
TRINITY_DN471_c0_g1_i1	dmso	control	0,46600234	6,6632659	0,00089333	0,00673495
TRINITY_DN5018_c0_g1_i1	dmso	control	0,46521133	5,77837222	0,00023782	0,00218977
TRINITY_DN9109_c0_g1_i1	dmso	control	0,46397766	4,05628823	0,01283705	0,05658566
TRINITY_DN2358_c0_g1_i1	dmso	control	0,46357263	3,39070934	0,02406124	0,09094024
TRINITY_DN1074_c0_g1_i1	dmso	control	0,46312574	7,32482693	5,02E-05	0,00057642
TRINITY_DN7681_c0_g1_i1	dmso	control	0,46198451	5,45745711	0,00103048	0,0075984
TRINITY_DN13073_c0_g1_i1	dmso	control	0,46157698	4,89211854	0,00208691	0,01353025
TRINITY_DN13881_c0_g1_i1	dmso	control	0,46070482	4,05990982	0,02266654	0,08693285
TRINITY_DN2242_c0_g1_i1	dmso	control	0,46009942	5,27680364	0,00105061	0,007735
TRINITY_DN4817_c0_g1_i1	dmso	control	0,45995535	7,19866311	0,00095068	0,00707862
TRINITY_DN1168_c0_g1_i1	dmso	control	0,45983182	4,52414534	0,00842932	0,04069572
TRINITY_DN1635_c0_g1_i1	dmso	control	0,45904983	4,49533632	0,01896971	0,07603869
TRINITY_DN23253_c0_g1_i1	dmso	control	0,45869208	3,93429513	0,01658158	0,06851286
TRINITY_DN5175_c0_g1_i1	dmso	control	0,45852446	6,68526255	0,00478635	0,02607045
TRINITY_DN20304_c0_g1_i1	dmso	control	0,4583517	3,89681147	0,01083832	0,04954416
TRINITY_DN2777_c0_g1_i1	dmso	control	0,458325	4,30425902	0,00729547	0,03628893
TRINITY_DN5270_c0_g1_i1	dmso	control	0,45823825	6,3876103	8,69E-05	0,00092314
TRINITY_DN2975_c0_g1_i1	dmso	control	0,45793942	3,67944501	0,01508554	0,06360583
TRINITY_DN1443_c0_g1_i1	dmso	control	0,45789244	6,75418062	7,99E-05	0,00086244

TRINITY_DN15691_c0_g1_i1	dmso	control	0,45779046	3,60989399	0,01687538	0,06950809
TRINITY_DN1655_c0_g1_i1	dmso	control	0,45727017	5,9513083	0,00229231	0,0145549
TRINITY_DN2743_c0_g1_i1	dmso	control	0,45714333	3,83495013	0,01161128	0,05220506
TRINITY_DN13344_c0_g1_i1	dmso	control	0,45701137	4,86022252	0,00681759	0,03431362
TRINITY_DN12314_c0_g1_i1	dmso	control	0,45670491	3,90259295	0,01728488	0,07081112
TRINITY_DN11474_c0_g1_i1	dmso	control	0,45660486	5,1188237	0,00148631	0,01030218
TRINITY_DN2504_c0_g1_i1	dmso	control	0,45576067	7,46855716	0,01101888	0,05016316
TRINITY_DN6644_c0_g1_i1	dmso	control	0,45534087	4,09327902	0,02061125	0,08101482
TRINITY_DN12734_c0_g1_i1	dmso	control	0,45478274	6,81952736	0,00287545	0,01750154
TRINITY_DN1988_c0_g1_i1	dmso	control	0,45459597	7,95216533	4,36E-05	0,00050828
TRINITY_DN6904_c0_g1_i1	dmso	control	0,45382626	3,93773741	0,01403436	0,06024211
TRINITY_DN1043_c0_g1_i1	dmso	control	0,45374407	7,84471191	0,00067625	0,00535255
TRINITY_DN7669_c0_g1_i1	dmso	control	0,45349292	4,97943544	0,00185936	0,01233736
TRINITY_DN892_c0_g1_i1	dmso	control	0,45338533	7,68071772	3,31E-05	0,0003982
TRINITY_DN1272_c0_g1_i1	dmso	control	0,4533038	4,57283451	0,00609193	0,03151012
TRINITY_DN33117_c0_g1_i1	dmso	control	0,45326342	3,65492428	0,0168386	0,06945561
TRINITY_DN33741_c0_g1_i1	dmso	control	0,45257359	7,70204459	0,00011145	0,00114828
TRINITY_DN19002_c0_g1_i1	dmso	control	0,45243294	4,75043927	0,01626132	0,06757452
TRINITY_DN13215_c0_g1_i1	dmso	control	0,45049676	4,30353383	0,01233471	0,05485679
TRINITY_DN11199_c0_g1_i1	dmso	control	0,45024567	5,66929556	0,0126646	0,0560822
TRINITY_DN12109_c0_g1_i1	dmso	control	0,44995738	3,74025472	0,01410338	0,06045204
TRINITY_DN12773_c0_g1_i1	dmso	control	0,44992132	4,16199189	0,00872816	0,04173396
TRINITY_DN20201_c0_g1_i1	dmso	control	0,44964116	4,02738971	0,01161196	0,05220506
TRINITY_DN2276_c0_g1_i1	dmso	control	0,44957689	5,08165049	0,0044715	0,0247854
TRINITY_DN12863_c0_g1_i1	dmso	control	0,44952158	4,25160439	0,01116795	0,05066614
TRINITY_DN6179_c0_g1_i1	dmso	control	0,44932558	5,14402396	0,00341565	0,02007287
TRINITY_DN5494_c0_g1_i1	dmso	control	0,44932084	6,02328868	0,00180309	0,01204627
TRINITY_DN1094_c0_g1_i1	dmso	control	0,44909807	3,89316661	0,01298449	0,05703633
TRINITY_DN955_c0_g1_i1	dmso	control	0,44869932	5,10873631	0,00368058	0,0212749
TRINITY_DN24498_c0_g1_i1	dmso	control	0,44858141	3,93759298	0,02187393	0,08466478
TRINITY_DN20814_c0_g1_i1	dmso	control	0,44678902	4,29198361	0,01410822	0,06045482

TRINITY_DN14645_c0_g1_i1	dmso	control	0,4466107	3,52234421	0,02415867	0,09113919
TRINITY_DN4810_c0_g1_i1	dmso	control	0,44635747	5,75454204	0,00263258	0,01627896
TRINITY_DN12025_c0_g1_i1	dmso	control	0,44630716	4,67133397	0,00420423	0,02354793
TRINITY_DN1510_c0_g1_i1	dmso	control	0,44567432	6,34202054	0,00180465	0,01204627
TRINITY_DN20395_c0_g1_i1	dmso	control	0,4455169	9,24017398	2,38E-05	0,00029843
TRINITY_DN2843_c0_g1_i1	dmso	control	0,44549493	4,44322902	0,00511523	0,02742797
TRINITY_DN20003_c0_g1_i1	dmso	control	0,44473036	4,73511662	0,00639167	0,03268238
TRINITY_DN987_c0_g1_i1	dmso	control	0,44468903	5,39620436	0,01039496	0,04789806
TRINITY_DN14198_c0_g1_i1	dmso	control	0,44424217	5,64701988	0,00259769	0,01611774
TRINITY_DN1582_c0_g1_i1	dmso	control	0,44353582	7,53650927	0,00283403	0,01727323
TRINITY_DN12334_c0_g1_i1	dmso	control	0,44329847	4,42453777	0,0088602	0,04220265
TRINITY_DN11619_c0_g1_i1	dmso	control	0,44312694	5,49123812	0,00097939	0,00725504
TRINITY_DN19429_c0_g1_i1	dmso	control	0,44204594	5,52091665	0,00523258	0,02793222
TRINITY_DN5123_c0_g1_i1	dmso	control	0,44181456	6,00779606	0,00043527	0,00367259
TRINITY_DN727_c0_g1_i1	dmso	control	0,44133743	6,48074184	0,00180413	0,01204627
TRINITY_DN6112_c0_g1_i1	dmso	control	0,44099519	4,69521048	0,00439782	0,02443328
TRINITY_DN6012_c0_g1_i1	dmso	control	0,44065917	4,97821059	0,00464197	0,02549226
TRINITY_DN2071_c0_g1_i1	dmso	control	0,43970187	7,62623038	0,00078962	0,00609949
TRINITY_DN4391_c0_g1_i1	dmso	control	0,43949542	4,7005676	0,00512702	0,02747813
TRINITY_DN2761_c0_g1_i1	dmso	control	0,43903649	5,2460298	0,00276582	0,01692894
TRINITY_DN11376_c0_g1_i1	dmso	control	0,43786779	3,63494242	0,02348212	0,08926297
TRINITY_DN432_c0_g1_i1	dmso	control	0,43764116	8,04758544	0,00477807	0,02603514
TRINITY_DN1915_c0_g1_i1	dmso	control	0,43758572	5,35322828	0,00215891	0,01391588
TRINITY_DN10910_c0_g1_i1	dmso	control	0,43713296	5,82767062	0,00715215	0,03573601
TRINITY_DN5976_c0_g1_i1	dmso	control	0,43698086	3,90837414	0,01911794	0,07649826
TRINITY_DN43928_c0_g1_i1	dmso	control	0,43691387	7,53441896	0,00015331	0,0015034
TRINITY_DN11993_c0_g1_i1	dmso	control	0,43610816	6,29785141	0,00348327	0,02041212
TRINITY_DN1122_c0_g1_i1	dmso	control	0,43610037	6,40919562	0,00224832	0,01433863
TRINITY_DN29811_c0_g1_i1	dmso	control	0,43576146	7,51606185	0,00239414	0,01509528
TRINITY_DN12009_c0_g1_i1	dmso	control	0,4356351	6,20292587	0,00679363	0,03422878
TRINITY_DN21427_c0_g1_i1	dmso	control	0,43499126	3,54097882	0,0224798	0,08645388

TRINITY_DN9484_c0_g1_i1	dmso	control	0,43470233	5,24037751	0,00333472	0,01970678
TRINITY_DN33523_c0_g1_i1	dmso	control	0,43450721	3,76011464	0,02526967	0,09427179
TRINITY_DN6115_c0_g1_i1	dmso	control	0,43395284	7,11090057	0,00023626	0,00218064
TRINITY_DN4065_c0_g1_i1	dmso	control	0,43383324	3,77923624	0,0269726	0,09906411
TRINITY_DN1598_c0_g1_i1	dmso	control	0,43335982	4,33949597	0,02576388	0,09564617
TRINITY_DN21726_c0_g1_i1	dmso	control	0,43301553	4,96866905	0,01205504	0,05377858
TRINITY_DN1343_c0_g1_i1	dmso	control	0,43269315	4,49146938	0,0143721	0,06126776
TRINITY_DN1751_c0_g1_i1	dmso	control	0,4326168	4,91583824	0,00379049	0,02174487
TRINITY_DN6260_c0_g1_i1	dmso	control	0,4324449	7,01636744	0,00038149	0,00328601
TRINITY_DN8292_c0_g1_i1	dmso	control	0,43243443	4,32818857	0,01374009	0,05935277
TRINITY_DN5929_c0_g1_i1	dmso	control	0,43230544	6,98178595	0,00014256	0,00141632
TRINITY_DN5800_c0_g1_i1	dmso	control	0,43205939	6,01326614	0,0190412	0,07625453
TRINITY_DN23968_c0_g1_i1	dmso	control	0,43178143	3,84762315	0,01773736	0,07224456
TRINITY_DN6025_c0_g1_i1	dmso	control	0,431564	5,28993231	0,0020896	0,01353558
TRINITY_DN1504_c0_g1_i1	dmso	control	0,43089142	7,09352653	0,00015002	0,00147918
TRINITY_DN6186_c0_g1_i1	dmso	control	0,43061291	5,57931216	0,00831258	0,04021272
TRINITY_DN5505_c0_g1_i1	dmso	control	0,42997991	5,75987681	0,0022951	0,01455985
TRINITY_DN3825_c0_g1_i1	dmso	control	0,42995662	8,0932714	0,00055479	0,00452765
TRINITY_DN19282_c0_g1_i1	dmso	control	0,42951486	4,7196989	0,01378249	0,05948254
TRINITY_DN2127_c0_g1_i1	dmso	control	0,42935557	5,00675925	0,00310436	0,01862885
TRINITY_DN11767_c0_g1_i1	dmso	control	0,42932561	6,17215126	0,00038622	0,00331485
TRINITY_DN18701_c0_g1_i1	dmso	control	0,42839635	4,98396438	0,00380067	0,02177574
TRINITY_DN5180_c0_g1_i1	dmso	control	0,42833583	7,62732146	0,00319952	0,01910587
TRINITY_DN1411_c0_g1_i1	dmso	control	0,42832986	5,15483087	0,01776432	0,07232402
TRINITY_DN1260_c0_g1_i1	dmso	control	0,42765964	4,22194643	0,01381325	0,0595797
TRINITY_DN1046_c0_g1_i1	dmso	control	0,42725126	4,84275953	0,00848683	0,0408641
TRINITY_DN3657_c0_g1_i1	dmso	control	0,42721967	5,60876273	0,00219336	0,01408433
TRINITY_DN638_c0_g1_i1	dmso	control	0,42707012	7,65538376	9,64E-05	0,00101522
TRINITY_DN11945_c0_g1_i1	dmso	control	0,42702961	7,16132827	0,00020849	0,00195309
TRINITY_DN2996_c0_g1_i1	dmso	control	0,42632842	3,85820353	0,02615772	0,09673534
TRINITY_DN4828_c0_g1_i1	dmso	control	0,42604357	6,9140941	0,0001607	0,00156952

TRINITY_DN4298_c0_g1_i1	dmso	control	0,42589812	5,48066938	0,00129792	0,00924023
TRINITY_DN15853_c0_g1_i1	dmso	control	0,42572693	4,18483523	0,01622335	0,06745718
TRINITY_DN22130_c0_g1_i1	dmso	control	0,42527893	4,12632648	0,01385965	0,0596907
TRINITY_DN2920_c0_g1_i1	dmso	control	0,42524191	4,51290083	0,01403773	0,06024211
TRINITY_DN13444_c0_g1_i1	dmso	control	0,42508356	4,42981419	0,02242727	0,08627481
TRINITY_DN11155_c0_g1_i1	dmso	control	0,4250702	4,08763341	0,0158087	0,06605631
TRINITY_DN22902_c0_g1_i1	dmso	control	0,42506103	4,52635209	0,00800997	0,039076
TRINITY_DN532_c0_g1_i1	dmso	control	0,42437652	6,80508635	0,00425343	0,0237682
TRINITY_DN54685_c0_g1_i1	dmso	control	0,4243269	4,07667973	0,01670955	0,06896269
TRINITY_DN10366_c0_g1_i1	dmso	control	0,42408578	3,84728	0,02193572	0,0847951
TRINITY_DN759_c0_g1_i1	dmso	control	0,42396683	7,1119107	0,00086754	0,00658171
TRINITY_DN12714_c0_g1_i1	dmso	control	0,42389137	6,10208266	0,0039054	0,02227141
TRINITY_DN8223_c0_g1_i1	dmso	control	0,42386508	3,74012869	0,02071515	0,08129049
TRINITY_DN4622_c0_g1_i1	dmso	control	0,42345089	6,83526915	0,00032448	0,00287197
TRINITY_DN7773_c0_g1_i1	dmso	control	0,42340611	3,9169643	0,02480903	0,09296381
TRINITY_DN10805_c0_g1_i1	dmso	control	0,42315322	6,09547818	0,00053915	0,00441751
TRINITY_DN5965_c0_g1_i1	dmso	control	0,42311365	5,85067639	0,00073775	0,0057511
TRINITY_DN3164_c0_g1_i1	dmso	control	0,4226406	4,03582741	0,0242602	0,09135372
TRINITY_DN12277_c0_g1_i1	dmso	control	0,4224362	5,8353577	0,00908058	0,04299039
TRINITY_DN12269_c0_g1_i1	dmso	control	0,42216121	5,68942385	0,00100017	0,00739001
TRINITY_DN5788_c0_g1_i1	dmso	control	0,42135467	6,64581835	0,00258171	0,016033
TRINITY_DN10753_c0_g1_i1	dmso	control	0,42132269	6,46062445	0,00080006	0,00616037
TRINITY_DN5055_c0_g1_i1	dmso	control	0,42091434	7,49741733	0,00176877	0,01187259
TRINITY_DN10911_c0_g1_i1	dmso	control	0,42085224	6,62211598	0,00054313	0,00444254
TRINITY_DN5344_c0_g1_i1	dmso	control	0,42039764	4,41647715	0,01603571	0,06683087
TRINITY_DN19066_c0_g1_i1	dmso	control	0,41985872	5,23552565	0,00714899	0,03573259
TRINITY_DN12454_c0_g1_i1	dmso	control	0,41970873	6,06303044	0,00282484	0,01723178
TRINITY_DN9356_c0_g1_i1	dmso	control	0,4193654	6,93034761	0,00036395	0,00315184
TRINITY_DN19580_c0_g1_i1	dmso	control	0,41891978	5,569233	0,00172756	0,01164467
TRINITY_DN15024_c0_g1_i1	dmso	control	0,41891729	4,6408271	0,01725304	0,07074087
TRINITY_DN1430_c0_g1_i1	dmso	control	0,41701243	4,55856908	0,02443159	0,09183303

TRINITY_DN11919_c0_g1_i1	dmso	control	0,41697464	3,84541694	0,02386753	0,09046616
TRINITY_DN6881_c0_g1_i1	dmso	control	0,41668603	4,82969034	0,0062099	0,03197935
TRINITY_DN13016_c0_g1_i1	dmso	control	0,41660633	3,97618738	0,0173887	0,07113732
TRINITY_DN19201_c0_g1_i1	dmso	control	0,41652091	6,97347196	0,00030376	0,00271192
TRINITY_DN16322_c0_g1_i1	dmso	control	0,41623349	6,71753411	0,01356609	0,05890057
TRINITY_DN17202_c0_g1_i1	dmso	control	0,41579796	4,90537414	0,00853523	0,04101638
TRINITY_DN6771_c0_g1_i1	dmso	control	0,41562852	5,88669053	0,00185566	0,0123278
TRINITY_DN1085_c0_g1_i1	dmso	control	0,41532216	5,31857671	0,0033279	0,01967723
TRINITY_DN7030_c0_g1_i1	dmso	control	0,41529023	4,13944591	0,0185327	0,07471529
TRINITY_DN18875_c0_g1_i1	dmso	control	0,41458892	9,88630132	0,00156297	0,01073564
TRINITY_DN14175_c0_g1_i1	dmso	control	0,41446557	3,91805584	0,02662931	0,09812764
TRINITY_DN11538_c0_g1_i1	dmso	control	0,41417865	7,04065635	0,00050496	0,00416811
TRINITY_DN5735_c0_g1_i1	dmso	control	0,41409956	5,32514939	0,00434126	0,02417485
TRINITY_DN1437_c0_g1_i1	dmso	control	0,41409859	4,73116008	0,00729274	0,03628783
TRINITY_DN73_c0_g1_i1	dmso	control	0,41394544	5,36150023	0,00498088	0,02688669
TRINITY_DN7567_c0_g1_i1	dmso	control	0,41360483	4,3573687	0,01871851	0,07529628
TRINITY_DN5481_c0_g1_i1	dmso	control	0,4134763	5,71190083	0,00207776	0,01348693
TRINITY_DN6844_c0_g1_i1	dmso	control	0,41327093	6,03254322	0,01613434	0,06716443
TRINITY_DN2100_c0_g1_i1	dmso	control	0,41304448	5,30206848	0,00738463	0,03665668
TRINITY_DN5198_c0_g1_i1	dmso	control	0,41245777	5,76525347	0,00169706	0,01147121
TRINITY_DN6270_c0_g1_i1	dmso	control	0,41228248	4,42207434	0,01251392	0,05558535
TRINITY_DN4847_c0_g1_i1	dmso	control	0,41179949	9,31391348	0,00018574	0,00177101
TRINITY_DN4680_c0_g1_i1	dmso	control	0,41169103	7,83981147	0,00016992	0,00164178
TRINITY_DN1415_c0_g1_i1	dmso	control	0,41111988	6,71974035	0,00032413	0,00287071
TRINITY_DN18981_c0_g1_i1	dmso	control	0,41109995	7,21291396	0,00231843	0,01466917
TRINITY_DN13560_c0_g1_i1	dmso	control	0,41044846	5,838514	0,00104224	0,00767731
TRINITY_DN18998_c0_g1_i1	dmso	control	0,41003406	4,38295588	0,01249992	0,05554023
TRINITY_DN7414_c0_g1_i1	dmso	control	0,40983902	4,38233926	0,02186674	0,08465962
TRINITY_DN7020_c0_g1_i1	dmso	control	0,40912274	4,6853303	0,01981753	0,07877272
TRINITY_DN32457_c0_g1_i1	dmso	control	0,40894835	5,53563805	0,00933154	0,04394982
TRINITY_DN5685_c0_g1_i1	dmso	control	0,40881661	5,27734964	0,00526752	0,02810837

TRINITY_DN312_c0_g1_i1	dmso	control	0,40862	6,26971101	0,0008657	0,00657123
TRINITY_DN1268_c0_g1_i1	dmso	control	0,40833629	5,87012017	0,0011373	0,00825597
TRINITY_DN7580_c0_g1_i1	dmso	control	0,40799981	5,53586248	0,0061189	0,03159178
TRINITY_DN11662_c0_g1_i1	dmso	control	0,40767733	5,06117761	0,01278193	0,05642879
TRINITY_DN1245_c0_g1_i1	dmso	control	0,40688869	4,51392823	0,01387514	0,05970073
TRINITY_DN5255_c0_g1_i1	dmso	control	0,40645526	7,76066375	0,00071343	0,00558864
TRINITY_DN6081_c0_g1_i1	dmso	control	0,40624679	6,64743898	0,00052495	0,00432567
TRINITY_DN13640_c0_g1_i1	dmso	control	0,40609927	3,95793179	0,02601732	0,09633947
TRINITY_DN1515_c0_g1_i1	dmso	control	0,40461528	5,39818337	0,00698517	0,03498639
TRINITY_DN2082_c0_g1_i1	dmso	control	0,40450555	6,56064992	0,00630041	0,03230719
TRINITY_DN5241_c0_g1_i1	dmso	control	0,4033946	7,08324692	0,00559613	0,02946996
TRINITY_DN2809_c0_g1_i1	dmso	control	0,4033037	5,79989616	0,00468539	0,02568521
TRINITY_DN444_c0_g1_i1	dmso	control	0,40328516	8,92662203	0,00035735	0,00311222
TRINITY_DN10843_c0_g1_i1	dmso	control	0,40325852	5,79683531	0,00163827	0,01113116
TRINITY_DN12355_c0_g1_i1	dmso	control	0,40275254	6,29476933	0,00366094	0,02117835
TRINITY_DN11871_c0_g1_i1	dmso	control	0,40264959	5,53295572	0,0032888	0,01951795
TRINITY_DN6375_c0_g1_i1	dmso	control	0,40240967	4,28250346	0,01480527	0,06275299
TRINITY_DN19560_c0_g1_i1	dmso	control	0,40202641	6,4120042	0,00123135	0,00883598
TRINITY_DN5633_c0_g1_i1	dmso	control	0,40169389	5,33139087	0,01646696	0,06813671
TRINITY_DN9085_c0_g1_i1	dmso	control	0,40126822	4,03648071	0,02082635	0,08157175
TRINITY_DN4826_c0_g1_i1	dmso	control	0,40097726	8,31913172	0,00054695	0,00447126
TRINITY_DN19028_c0_g1_i1	dmso	control	0,40087731	4,83684167	0,00741571	0,03676042
TRINITY_DN2420_c0_g1_i1	dmso	control	0,40084771	6,58012611	0,00066861	0,00530368
TRINITY_DN15589_c0_g1_i1	dmso	control	0,40002418	5,31161562	0,0095	0,04464136
TRINITY_DN18741_c0_g1_i1	dmso	control	0,39979557	4,15069756	0,02455643	0,09218235
TRINITY_DN11372_c0_g1_i1	dmso	control	0,39912201	5,04228371	0,00901785	0,04275117
TRINITY_DN45590_c0_g1_i1	dmso	control	0,3980418	4,38939896	0,01866155	0,07510898
TRINITY_DN10738_c0_g1_i1	dmso	control	0,39787628	7,21971188	0,00034104	0,00299658
TRINITY_DN11446_c0_g1_i1	dmso	control	0,39759331	5,22305594	0,00573555	0,03007262
TRINITY_DN10944_c0_g1_i1	dmso	control	0,39757138	4,26707428	0,02317084	0,08837174
TRINITY_DN12174_c0_g1_i1	dmso	control	0,39698243	5,33346526	0,00458935	0,02530272

TRINITY_DN7666_c0_g1_i1	dmso	control	0,39674327	5,39661609	0,0151928	0,06400115
TRINITY_DN5663_c0_g1_i1	dmso	control	0,39566034	4,43997893	0,01787758	0,07270317
TRINITY_DN12012_c0_g1_i1	dmso	control	0,39478909	4,78237396	0,01041184	0,04793855
TRINITY_DN11107_c0_g1_i1	dmso	control	0,39405132	5,66961324	0,0026986	0,01658075
TRINITY_DN1761_c0_g1_i1	dmso	control	0,39387382	5,20455329	0,00555582	0,02931113
TRINITY_DN1907_c0_g1_i1	dmso	control	0,39351071	7,29236909	0,00039091	0,0033452
TRINITY_DN11657_c0_g1_i1	dmso	control	0,39307819	5,83993986	0,0060734	0,03144452
TRINITY_DN22816_c0_g1_i1	dmso	control	0,39268721	4,40051437	0,01713504	0,07035691
TRINITY_DN10816_c0_g1_i1	dmso	control	0,39173146	7,68694847	0,00087564	0,00662924
TRINITY_DN1135_c0_g1_i1	dmso	control	0,39139534	6,78484289	0,02106241	0,08234007
TRINITY_DN2181_c0_g1_i1	dmso	control	0,39108465	4,67998293	0,02579139	0,09572369
TRINITY_DN244_c0_g1_i1	dmso	control	0,39072948	8,2676153	0,00031446	0,0027963
TRINITY_DN5482_c0_g1_i1	dmso	control	0,39058752	6,12520441	0,00248001	0,015535
TRINITY_DN841_c0_g1_i1	dmso	control	0,39042676	5,70158279	0,00324496	0,01932129
TRINITY_DN12740_c0_g1_i1	dmso	control	0,39008429	5,37817085	0,0058396	0,03047374
TRINITY_DN5354_c0_g1_i1	dmso	control	0,38868971	8,58111304	0,00388208	0,02214718
TRINITY_DN2281_c0_g1_i1	dmso	control	0,38844399	4,78450614	0,01558426	0,06520098
TRINITY_DN6810_c0_g1_i1	dmso	control	0,38825763	5,51878852	0,0038019	0,02177574
TRINITY_DN1611_c0_g1_i1	dmso	control	0,38807743	5,97406337	0,01066323	0,04886752
TRINITY_DN18971_c0_g1_i1	dmso	control	0,38753853	8,93697216	0,00023911	0,00219855
TRINITY_DN40_c0_g1_i1	dmso	control	0,38681773	8,27841886	0,00098641	0,00729954
TRINITY_DN14180_c0_g1_i1	dmso	control	0,38637753	4,88921266	0,0226363	0,08684765
TRINITY_DN34556_c0_g1_i1	dmso	control	0,38624516	6,13899289	0,00660288	0,03350411
TRINITY_DN5114_c0_g1_i1	dmso	control	0,38623183	6,18734793	0,026811	0,09857086
TRINITY_DN5360_c0_g1_i1	dmso	control	0,38619564	5,68780522	0,00315657	0,01888843
TRINITY_DN1570_c0_g1_i1	dmso	control	0,3861862	5,08346875	0,01169897	0,05251447
TRINITY_DN6207_c0_g1_i1	dmso	control	0,38581352	5,79262738	0,00222625	0,0142293
TRINITY_DN2421_c0_g1_i1	dmso	control	0,38508039	5,94694705	0,00346387	0,02031491
TRINITY_DN5709_c0_g1_i1	dmso	control	0,38457922	6,28302807	0,01644937	0,0680834
TRINITY_DN4181_c0_g1_i1	dmso	control	0,38404492	6,28333804	0,00148471	0,01029604
TRINITY_DN19251_c0_g1_i1	dmso	control	0,38385027	4,67811748	0,01698964	0,06987909

TRINITY_DN2147_c0_g1_i1	dmso	control	0,38336188	6,74430376	0,01294542	0,05695908
TRINITY_DN2490_c0_g1_i1	dmso	control	0,38303551	6,25220062	0,01643885	0,06805937
TRINITY_DN436_c0_g1_i1	dmso	control	0,38227673	9,51882404	0,00026311	0,00238734
TRINITY_DN11840_c0_g1_i1	dmso	control	0,3820831	4,86028221	0,01834349	0,07412765
TRINITY_DN4815_c0_g1_i1	dmso	control	0,38177721	6,99668543	0,00063299	0,00506851
TRINITY_DN1483_c0_g1_i1	dmso	control	0,38163421	6,18443914	0,00137948	0,00968252
TRINITY_DN6034_c0_g1_i1	dmso	control	0,38149449	5,3468234	0,00554117	0,02926181
TRINITY_DN19678_c0_g1_i1	dmso	control	0,38142145	4,97812646	0,01920734	0,07670722
TRINITY_DN1000_c0_g1_i1	dmso	control	0,381055	7,75831161	0,00226942	0,01443494
TRINITY_DN11170_c0_g1_i1	dmso	control	0,38075114	6,08675799	0,02278243	0,08724606
TRINITY_DN5318_c0_g1_i1	dmso	control	0,38028637	6,18279499	0,00659302	0,03348668
TRINITY_DN6018_c0_g1_i1	dmso	control	0,38026334	5,0349646	0,01529332	0,06433082
TRINITY_DN10912_c0_g1_i1	dmso	control	0,38016103	5,50413466	0,00472282	0,02584132
TRINITY_DN3730_c0_g1_i1	dmso	control	0,38014443	5,59618896	0,01492391	0,06312618
TRINITY_DN28596_c0_g1_i1	dmso	control	0,37835603	6,36718649	0,00326115	0,01940168
TRINITY_DN7067_c0_g1_i1	dmso	control	0,37682934	5,85388666	0,00255598	0,01588689
TRINITY_DN20624_c0_g1_i1	dmso	control	0,3765444	6,32533752	0,00203143	0,01324188
TRINITY_DN6211_c0_g1_i1	dmso	control	0,37617972	6,56877674	0,00418278	0,02345505
TRINITY_DN3018_c0_g1_i1	dmso	control	0,37607776	8,28507514	0,00135621	0,00956095
TRINITY_DN30689_c0_g1_i1	dmso	control	0,37546203	8,37238013	0,0015038	0,01039471
TRINITY_DN8217_c0_g1_i1	dmso	control	0,37533288	5,24816153	0,01711269	0,07028512
TRINITY_DN14560_c0_g1_i1	dmso	control	0,37516435	5,58876933	0,00764866	0,03764851
TRINITY_DN311_c0_g1_i1	dmso	control	0,37482358	8,34619785	0,00076243	0,00591798
TRINITY_DN20456_c0_g1_i1	dmso	control	0,37447579	4,30318119	0,02353086	0,08940118
TRINITY_DN1601_c0_g1_i1	dmso	control	0,37440598	4,44020051	0,02409925	0,09100101
TRINITY_DN5543_c0_g1_i1	dmso	control	0,3742936	6,43238527	0,00245525	0,01540662
TRINITY_DN5387_c0_g1_i1	dmso	control	0,37424522	6,74410938	0,00989002	0,04611405
TRINITY_DN32418_c0_g1_i1	dmso	control	0,37417505	5,09455494	0,01720257	0,07055397
TRINITY_DN18668_c0_g1_i1	dmso	control	0,37384977	6,07131502	0,00757681	0,03736669
TRINITY_DN9975_c0_g1_i1	dmso	control	0,37380167	4,79510051	0,02718555	0,09974479
TRINITY_DN4967_c0_g1_i1	dmso	control	0,37348306	6,59113189	0,00161598	0,01104204

TRINITY_DN11900_c0_g1_i1	dmso	control	0,37325519	5,06474216	0,00985831	0,04598102
TRINITY_DN2136_c0_g1_i1	dmso	control	0,37325383	5,22054523	0,0082705	0,0400613
TRINITY_DN1329_c0_g1_i1	dmso	control	0,3732322	6,01392195	0,00677633	0,0341633
TRINITY_DN1738_c0_g1_i1	dmso	control	0,37287028	7,08373544	0,00132098	0,0093583
TRINITY_DN1259_c0_g1_i1	dmso	control	0,37268819	7,94147077	0,00548852	0,02904091
TRINITY_DN11205_c0_g1_i1	dmso	control	0,37264684	5,49182798	0,00515357	0,02760225
TRINITY_DN11960_c0_g1_i1	dmso	control	0,37257664	6,38542286	0,00404074	0,02285375
TRINITY_DN11121_c0_g1_i1	dmso	control	0,37170757	5,86058218	0,0150185	0,0634705
TRINITY_DN1712_c0_g1_i1	dmso	control	0,37170205	5,85024776	0,00298643	0,01802717
TRINITY_DN13653_c0_g1_i1	dmso	control	0,37150906	4,98884888	0,01302651	0,05715917
TRINITY_DN2958_c0_g1_i1	dmso	control	0,37002032	5,34535896	0,01014653	0,04699153
TRINITY_DN1452_c0_g1_i1	dmso	control	0,36991099	8,94990792	0,00111537	0,00812075
TRINITY_DN17132_c0_g1_i1	dmso	control	0,36989702	4,57949087	0,02030607	0,08012052
TRINITY_DN4988_c0_g1_i1	dmso	control	0,36920573	5,71429291	0,00447141	0,0247854
TRINITY_DN315_c0_g1_i1	dmso	control	0,36882246	6,80182184	0,00140846	0,00984765
TRINITY_DN6773_c0_g1_i1	dmso	control	0,36878333	4,4813784	0,02317426	0,08837174
TRINITY_DN935_c0_g1_i1	dmso	control	0,3683302	6,423002	0,00250239	0,01565485
TRINITY_DN18910_c0_g1_i1	dmso	control	0,36785669	4,35146321	0,02540433	0,09467637
TRINITY_DN234_c0_g1_i1	dmso	control	0,3670496	7,17825989	0,00095346	0,007086
TRINITY_DN5486_c0_g1_i1	dmso	control	0,36693298	6,71402606	0,00148727	0,0103039
TRINITY_DN24887_c0_g1_i1	dmso	control	0,36682969	6,65318027	0,00950974	0,04467258
TRINITY_DN18680_c0_g1_i1	dmso	control	0,36615248	6,36478658	0,00548837	0,02904091
TRINITY_DN1108_c0_g1_i1	dmso	control	0,36571076	6,34110729	0,00215673	0,01390802
TRINITY_DN5246_c0_g1_i1	dmso	control	0,36553312	7,70776029	0,00081322	0,00624837
TRINITY_DN3420_c0_g1_i1	dmso	control	0,36549237	9,1432278	0,00085959	0,00655235
TRINITY_DN2360_c0_g1_i1	dmso	control	0,36461213	5,26344828	0,02335575	0,08887613
TRINITY_DN6166_c0_g1_i1	dmso	control	0,36429376	5,25390202	0,01067906	0,04892453
TRINITY_DN144_c0_g1_i1	dmso	control	0,36327288	7,16520805	0,00217357	0,01397918
TRINITY_DN8466_c0_g1_i1	dmso	control	0,36297493	6,68965567	0,00319718	0,01909978
TRINITY_DN5514_c0_g1_i1	dmso	control	0,36287062	6,68986966	0,00162133	0,01105768
TRINITY_DN5406_c0_g1_i1	dmso	control	0,36218056	8,06537742	0,00140722	0,00984375

TRINITY_DN1796_c0_g1_i1	dmso	control	0,36168254	4,98333792	0,01633691	0,06775221
TRINITY_DN4775_c0_g1_i1	dmso	control	0,3612701	7,18111698	0,00297011	0,01794362
TRINITY_DN33661_c0_g1_i1	dmso	control	0,3611068	6,53393447	0,00621897	0,03201461
TRINITY_DN1308_c0_g1_i1	dmso	control	0,36098551	5,40215803	0,00897986	0,04263518
TRINITY_DN12284_c0_g1_i1	dmso	control	0,36076776	6,22322785	0,01275218	0,05635519
TRINITY_DN1803_c0_g1_i1	dmso	control	0,36027021	6,12655341	0,01281049	0,05652031
TRINITY_DN8_c0_g1_i1	dmso	control	0,3596183	7,23270823	0,00168673	0,01141209
TRINITY_DN5530_c0_g1_i1	dmso	control	0,35900154	5,83694148	0,00492375	0,02666242
TRINITY_DN2275_c0_g1_i1	dmso	control	0,35835647	7,92417998	0,00118291	0,00853278
TRINITY_DN1539_c0_g1_i1	dmso	control	0,35736203	5,46604918	0,00930454	0,04387989
TRINITY_DN2333_c0_g1_i1	dmso	control	0,35722416	7,35141776	0,00204481	0,01331107
TRINITY_DN32359_c0_g1_i1	dmso	control	0,35696888	5,57806181	0,01104935	0,05025435
TRINITY_DN13269_c0_g1_i1	dmso	control	0,35694648	6,22437145	0,00283771	0,01728838
TRINITY_DN215_c0_g1_i1	dmso	control	0,35686986	7,65766513	0,0011475	0,00831272
TRINITY_DN12247_c0_g1_i1	dmso	control	0,35493363	5,96694843	0,00482775	0,02625633
TRINITY_DN10725_c0_g1_i1	dmso	control	0,35445836	9,01621215	0,00141986	0,00992253
TRINITY_DN983_c0_g1_i1	dmso	control	0,35363224	8,29551714	0,00212403	0,01372782
TRINITY_DN5456_c0_g1_i1	dmso	control	0,35341641	5,49248077	0,0077751	0,03810944
TRINITY_DN1864_c0_g1_i1	dmso	control	0,3534002	4,77701619	0,02194191	0,0847951
TRINITY_DN14808_c0_g1_i1	dmso	control	0,35203382	4,83919601	0,0249758	0,09344707
TRINITY_DN20886_c0_g1_i1	dmso	control	0,35185095	5,53231825	0,02152877	0,08368758
TRINITY_DN136_c0_g1_i1	dmso	control	0,35139227	7,7897841	0,0046721	0,02563176
TRINITY_DN16697_c0_g1_i1	dmso	control	0,35136691	5,18661888	0,02617133	0,09676091
TRINITY_DN21852_c0_g1_i1	dmso	control	0,35121161	5,61694092	0,01937734	0,07730066
TRINITY_DN2210_c0_g1_i1	dmso	control	0,35110219	5,50867782	0,00836471	0,04043783
TRINITY_DN26616_c0_g1_i1	dmso	control	0,3503933	5,76761689	0,00771491	0,03786674
TRINITY_DN4890_c0_g1_i1	dmso	control	0,34937316	5,85086228	0,00577762	0,03021641
TRINITY_DN11090_c0_g1_i1	dmso	control	0,34898128	5,07704443	0,01449987	0,06160312
TRINITY_DN786_c0_g1_i1	dmso	control	0,3483214	8,35118787	0,0019395	0,01273456
TRINITY_DN249_c0_g1_i1	dmso	control	0,34822088	8,02053946	0,00353441	0,0206448
TRINITY_DN20372_c0_g1_i1	dmso	control	0,3474711	4,6069674	0,02542909	0,09470259

TRINITY_DN6444_c0_g1_i1	dmso	control	0,34638794	5,70795093	0,02069718	0,08124206
TRINITY_DN5492_c0_g1_i1	dmso	control	0,34607886	9,33749444	0,00201077	0,01313091
TRINITY_DN6288_c0_g1_i1	dmso	control	0,34468175	6,26010164	0,00815577	0,03966296
TRINITY_DN11269_c0_g1_i1	dmso	control	0,34434675	5,96114606	0,00681714	0,03431362
TRINITY_DN6193_c0_g1_i1	dmso	control	0,34381771	7,7859246	0,00177476	0,01190116
TRINITY_DN19280_c0_g1_i1	dmso	control	0,34355539	5,28007331	0,01421795	0,06076281
TRINITY_DN5870_c0_g1_i1	dmso	control	0,34338082	6,60694982	0,00643277	0,03284599
TRINITY_DN14061_c0_g1_i1	dmso	control	0,34232068	7,04407145	0,02020304	0,07986701
TRINITY_DN1307_c0_g1_i1	dmso	control	0,3419612	4,86668973	0,02600562	0,09633377
TRINITY_DN7370_c0_g1_i1	dmso	control	0,34184634	7,12393147	0,00303371	0,01828201
TRINITY_DN5793_c0_g1_i1	dmso	control	0,34104473	5,68849387	0,01259922	0,05582693
TRINITY_DN2019_c0_g1_i1	dmso	control	0,34080495	7,17501011	0,00608	0,03146741
TRINITY_DN23671_c0_g1_i1	dmso	control	0,33989726	5,27888884	0,01438649	0,06129318
TRINITY_DN881_c0_g1_i1	dmso	control	0,33980669	6,40030361	0,00329199	0,01952888
TRINITY_DN132_c0_g1_i1	dmso	control	0,33948865	7,79532887	0,00709011	0,03546284
TRINITY_DN6493_c0_g1_i1	dmso	control	0,33941472	5,53228062	0,01166406	0,05239036
TRINITY_DN236_c0_g1_i1	dmso	control	0,33912749	6,33672634	0,00454147	0,02511544
TRINITY_DN177_c0_g1_i1	dmso	control	0,33905484	9,08759223	0,00841312	0,04063106
TRINITY_DN12177_c0_g1_i1	dmso	control	0,33897056	6,31805074	0,00575584	0,03015709
TRINITY_DN12045_c0_g1_i1	dmso	control	0,33873949	5,90056	0,01839302	0,07427653
TRINITY_DN2623_c0_g1_i1	dmso	control	0,33828577	4,66657641	0,02717466	0,09973019
TRINITY_DN33040_c0_g1_i1	dmso	control	0,33817524	6,89966451	0,00760812	0,0374955
TRINITY_DN5704_c0_g1_i1	dmso	control	0,3372294	5,21999484	0,01543092	0,06477758
TRINITY_DN11397_c0_g1_i1	dmso	control	0,33719935	5,83947668	0,00847066	0,04082754
TRINITY_DN12197_c0_g1_i1	dmso	control	0,33680624	6,83552354	0,00428208	0,0238821
TRINITY_DN18679_c0_g1_i1	dmso	control	0,33638254	7,89959159	0,0029498	0,01785919
TRINITY_DN14770_c0_g1_i1	dmso	control	0,3358221	5,56668022	0,01024767	0,04736884
TRINITY_DN3110_c0_g1_i1	dmso	control	0,3336862	6,29674052	0,01049191	0,04822005
TRINITY_DN11066_c0_g1_i1	dmso	control	0,33365487	6,24621125	0,00737768	0,03663476
TRINITY_DN585_c0_g1_i1	dmso	control	0,33358144	7,98746862	0,00285526	0,01738795
TRINITY_DN12135_c0_g1_i1	dmso	control	0,33328324	6,71857373	0,00415791	0,02333375

TRINITY_DN11267_c0_g1_i1	dmso	control	0,33301608	5,27942114	0,0205785	0,08090811
TRINITY_DN12316_c0_g1_i1	dmso	control	0,33223565	6,71779969	0,01129151	0,05116242
TRINITY_DN4658_c0_g1_i1	dmso	control	0,33220917	9,55321812	0,02670476	0,09830538
TRINITY_DN19467_c0_g1_i1	dmso	control	0,3318925	5,66838021	0,01380286	0,05955265
TRINITY_DN19012_c0_g1_i1	dmso	control	0,33035826	6,56722559	0,0040939	0,02305514
TRINITY_DN12620_c0_g1_i1	dmso	control	0,32964838	5,47481677	0,01345995	0,05861592
TRINITY_DN2643_c0_g1_i1	dmso	control	0,32958572	7,25916362	0,00321938	0,01920858
TRINITY_DN20064_c0_g1_i1	dmso	control	0,32896714	8,79418123	0,00296967	0,01794362
TRINITY_DN8095_c0_g1_i1	dmso	control	0,32839711	5,15367299	0,02291131	0,08762347
TRINITY_DN2496_c0_g1_i1	dmso	control	0,32801191	5,53986773	0,02033758	0,08018403
TRINITY_DN1265_c0_g1_i1	dmso	control	0,3279548	6,30911721	0,00739679	0,03669184
TRINITY_DN6410_c0_g1_i1	dmso	control	0,32754006	5,35681409	0,01629405	0,06763435
TRINITY_DN633_c0_g1_i1	dmso	control	0,32740641	9,09770805	0,00362977	0,02105701
TRINITY_DN12097_c0_g1_i1	dmso	control	0,32581803	6,29043195	0,00866018	0,04149132
TRINITY_DN6129_c0_g1_i1	dmso	control	0,3256172	6,83242556	0,00670175	0,03386036
TRINITY_DN129_c0_g1_i1	dmso	control	0,32557002	8,19041515	0,00990473	0,04615282
TRINITY_DN5138_c0_g1_i1	dmso	control	0,32506191	6,41567106	0,00519691	0,02775914
TRINITY_DN5947_c0_g1_i1	dmso	control	0,32501881	5,87300902	0,01389459	0,05975212
TRINITY_DN5370_c0_g1_i1	dmso	control	0,3223503	7,57744398	0,00432543	0,02410525
TRINITY_DN3531_c0_g1_i1	dmso	control	0,32095148	6,22637525	0,01001857	0,04654815
TRINITY_DN21035_c0_g1_i1	dmso	control	0,32036643	6,28957181	0,02165871	0,08407531
TRINITY_DN457_c0_g1_i1	dmso	control	0,31948072	7,55734911	0,01228224	0,05464029
TRINITY_DN411_c0_g1_i1	dmso	control	0,31902594	8,24261642	0,00295789	0,01789228
TRINITY_DN4818_c0_g1_i1	dmso	control	0,31880615	7,35070025	0,00820932	0,03981988
TRINITY_DN2357_c0_g1_i1	dmso	control	0,31872768	6,44267751	0,00586868	0,03058193
TRINITY_DN10752_c0_g1_i1	dmso	control	0,31871145	8,94067548	0,00404934	0,02287799
TRINITY_DN4910_c0_g1_i1	dmso	control	0,31768854	5,85747628	0,01135386	0,05135873
TRINITY_DN1379_c0_g1_i1	dmso	control	0,31741523	5,59363689	0,01651519	0,06827759
TRINITY_DN1016_c0_g1_i1	dmso	control	0,31737743	6,80810608	0,00590078	0,03072705
TRINITY_DN6813_c0_g1_i1	dmso	control	0,31730036	5,28656424	0,024043	0,09091652
TRINITY_DN5039_c0_g1_i1	dmso	control	0,31668415	8,41631992	0,00809723	0,03940853

TRINITY_DN2185_c0_g1_i1	dmso	control	0,31636275	5,50957087	0,0175257	0,0716352
TRINITY_DN1861_c0_g1_i1	dmso	control	0,31553063	6,12309898	0,01309734	0,05740019
TRINITY_DN2597_c0_g1_i1	dmso	control	0,31428667	5,73756683	0,02390907	0,09057607
TRINITY_DN241_c0_g1_i1	dmso	control	0,31334868	8,18553242	0,02310819	0,08821294
TRINITY_DN12031_c0_g1_i1	dmso	control	0,31316009	6,04400277	0,01009343	0,04677563
TRINITY_DN390_c0_g1_i1	dmso	control	0,31295192	9,67443694	0,00387275	0,02210439
TRINITY_DN7997_c0_g1_i1	dmso	control	0,31254229	5,90544013	0,02010022	0,07954732
TRINITY_DN5552_c0_g1_i1	dmso	control	0,31224524	7,7108818	0,02001235	0,07928643
TRINITY_DN676_c0_g1_i1	dmso	control	0,31210652	5,33512416	0,02648877	0,09768454
TRINITY_DN941_c0_g1_i1	dmso	control	0,31050882	8,0240331	0,00407887	0,02297944
TRINITY_DN32449_c0_g1_i1	dmso	control	0,31034528	6,51847723	0,01202732	0,05368806
TRINITY_DN1757_c0_g1_i1	dmso	control	0,30972536	5,95381036	0,01631185	0,0676888
TRINITY_DN7332_c0_g1_i1	dmso	control	0,30890274	7,02157369	0,00666525	0,03374679
TRINITY_DN1460_c0_g1_i1	dmso	control	0,30730358	6,09155531	0,01829315	0,07397662
TRINITY_DN5711_c0_g1_i1	dmso	control	0,30723777	7,18478099	0,01057005	0,04854828
TRINITY_DN7099_c0_g1_i1	dmso	control	0,30581536	5,80954657	0,01557726	0,06520098
TRINITY_DN3129_c0_g1_i1	dmso	control	0,30432309	7,63706269	0,00569609	0,02993091
TRINITY_DN4954_c0_g1_i1	dmso	control	0,30415065	8,0755035	0,00782325	0,03830739
TRINITY_DN1202_c0_g1_i1	dmso	control	0,30405318	6,00538339	0,01432457	0,061099
TRINITY_DN19240_c0_g1_i1	dmso	control	0,30344639	7,12657942	0,01974593	0,07851087
TRINITY_DN12487_c0_g1_i1	dmso	control	0,30309623	5,98333898	0,02532516	0,09443001
TRINITY_DN1215_c0_g1_i1	dmso	control	0,30280828	6,14437405	0,01240774	0,05516463
TRINITY_DN11144_c0_g1_i1	dmso	control	0,3027193	6,31370308	0,01170705	0,05253442
TRINITY_DN1045_c0_g1_i1	dmso	control	0,30136016	5,90345182	0,01521914	0,06407477
TRINITY_DN5303_c0_g1_i1	dmso	control	0,30100172	6,07077606	0,01481762	0,06276849
TRINITY_DN6001_c0_g1_i1	dmso	control	0,30068917	6,39787684	0,02040951	0,0803969
TRINITY_DN11602_c0_g1_i1	dmso	control	0,29953567	5,62440813	0,02015621	0,07972521
TRINITY_DN1589_c0_g1_i1	dmso	control	0,29937767	6,57156573	0,02630606	0,09713472
TRINITY_DN5161_c0_g1_i1	dmso	control	0,29937378	6,10545079	0,01448354	0,06156534
TRINITY_DN541_c0_g1_i1	dmso	control	0,29932739	8,1604526	0,01215082	0,05414507
TRINITY_DN11204_c0_g1_i1	dmso	control	0,29758383	5,72130464	0,01964925	0,07816949

TRINITY_DN2047_c0_g1_i1	dmso	control	0,29676889	6,49286922	0,01463832	0,0621
TRINITY_DN4877_c0_g1_i1	dmso	control	0,29571556	7,69727492	0,00767506	0,0377505
TRINITY_DN10961_c0_g1_i1	dmso	control	0,29536635	8,01489387	0,01015465	0,04701407
TRINITY_DN837_c0_g1_i1	dmso	control	0,29501073	6,81579688	0,00965387	0,04521731
TRINITY_DN484_c0_g1_i1	dmso	control	0,29409739	7,87552229	0,0120459	0,05375441
TRINITY_DN5538_c0_g1_i1	dmso	control	0,29396771	6,66053423	0,01698383	0,06987507
TRINITY_DN18728_c0_g1_i1	dmso	control	0,29287292	8,26474326	0,0063612	0,03256112
TRINITY_DN580_c0_g1_i1	dmso	control	0,2928137	6,67861116	0,0193528	0,0772241
TRINITY_DN6185_c0_g1_i1	dmso	control	0,29272136	6,00368836	0,01960345	0,07803027
TRINITY_DN5267_c0_g1_i1	dmso	control	0,29264969	8,53377329	0,00625254	0,0321416
TRINITY_DN666_c0_g1_i1	dmso	control	0,29070736	7,48558244	0,00848213	0,04085857
TRINITY_DN7039_c0_g1_i1	dmso	control	0,29056717	6,13298681	0,01682964	0,06943847
TRINITY_DN6063_c0_g1_i1	dmso	control	0,28992496	7,05844856	0,01768386	0,07213876
TRINITY_DN1432_c0_g1_i1	dmso	control	0,28926306	5,81625522	0,02496769	0,09344707
TRINITY_DN1853_c0_g1_i1	dmso	control	0,288626	9,13510751	0,00787879	0,03855321
TRINITY_DN5251_c0_g1_i1	dmso	control	0,28848329	6,5065958	0,0165714	0,06849038
TRINITY_DN24_c0_g1_i1	dmso	control	0,28807189	7,5716966	0,01476831	0,06261468
TRINITY_DN923_c0_g1_i1	dmso	control	0,28805567	7,94432249	0,00802274	0,03909868
TRINITY_DN1053_c0_g1_i1	dmso	control	0,28750605	7,71561192	0,00898453	0,04263518
TRINITY_DN2985_c0_g1_i1	dmso	control	0,2861707	7,85626621	0,0133253	0,05815227
TRINITY_DN11044_c0_g1_i1	dmso	control	0,28605347	7,25246511	0,01280999	0,05652031
TRINITY_DN971_c0_g1_i1	dmso	control	0,28552504	5,85648094	0,02640187	0,09741379
TRINITY_DN1139_c0_g1_i1	dmso	control	0,28542584	7,70928534	0,00993326	0,04621125
TRINITY_DN6188_c0_g1_i1	dmso	control	0,285395	6,28314026	0,02516908	0,09396934
TRINITY_DN609_c0_g1_i1	dmso	control	0,28494797	7,39844729	0,01222991	0,05445776
TRINITY_DN6189_c0_g1_i1	dmso	control	0,28445006	5,89368723	0,0239911	0,09076778
TRINITY_DN5435_c0_g1_i1	dmso	control	0,28275152	7,12367035	0,01690457	0,06956881
TRINITY_DN2438_c0_g1_i1	dmso	control	0,28264741	9,21813729	0,00717268	0,03576435
TRINITY_DN44_c0_g1_i1	dmso	control	0,28251219	5,86989883	0,02321433	0,08846498
TRINITY_DN18622_c0_g1_i1	dmso	control	0,28217708	6,72086549	0,0241825	0,09115769
TRINITY_DN861_c0_g1_i1	dmso	control	0,28212089	11,0109809	0,01009279	0,04677563

TRINITY_DN11008_c0_g1_i1	dmso	control	0,28209935	7,31065078	0,01021308	0,04723919
TRINITY_DN2228_c0_g1_i1	dmso	control	0,28116561	5,78863275	0,02661509	0,09810027
TRINITY_DN6284_c0_g1_i1	dmso	control	0,27920855	6,16117376	0,01870289	0,07525439
TRINITY_DN5739_c0_g1_i1	dmso	control	0,27617212	5,96206443	0,02598186	0,09633163
TRINITY_DN306_c0_g1_i1	dmso	control	0,27449469	7,67373436	0,01758484	0,0718054
TRINITY_DN3673_c0_g1_i1	dmso	control	0,27426641	8,19569796	0,01060312	0,04865377
TRINITY_DN1848_c0_g1_i1	dmso	control	0,27399071	6,48667416	0,02612938	0,09665528
TRINITY_DN965_c0_g1_i1	dmso	control	0,27329866	7,49630779	0,02403333	0,09090375
TRINITY_DN921_c0_g1_i1	dmso	control	0,27071283	8,51784919	0,01146546	0,05172348
TRINITY_DN75_c0_g1_i1	dmso	control	0,27038793	7,92466188	0,01323652	0,05783472
TRINITY_DN279_c0_g1_i1	dmso	control	0,2677333	8,34557362	0,01431855	0,061099
TRINITY_DN369_c0_g1_i1	dmso	control	0,26728864	8,97911221	0,01140597	0,05154078
TRINITY_DN1372_c0_g1_i1	dmso	control	0,26696697	6,91001372	0,01752484	0,0716352
TRINITY_DN7063_c0_g1_i1	dmso	control	0,26666431	6,66524339	0,0235188	0,08937885
TRINITY_DN365_c0_g1_i1	dmso	control	0,26626432	7,99905339	0,01628998	0,06763435
TRINITY_DN2620_c0_g1_i1	dmso	control	0,26535762	7,14739205	0,01733333	0,07096939
TRINITY_DN19562_c0_g1_i1	dmso	control	0,26417783	8,98192719	0,01538223	0,06464835
TRINITY_DN4985_c0_g1_i1	dmso	control	0,26074806	6,96074658	0,01970416	0,07836635
TRINITY_DN77_c0_g1_i1	dmso	control	0,25886253	9,4157282	0,02439101	0,09172825
TRINITY_DN10688_c0_g1_i1	dmso	control	0,25813369	10,4219231	0,0127324	0,0563134
TRINITY_DN12912_c0_g1_i1	dmso	control	0,25796704	7,14579443	0,02628835	0,09709414
TRINITY_DN1031_c0_g1_i1	dmso	control	0,2571773	9,80954621	0,01852422	0,07470194
TRINITY_DN5125_c0_g1_i1	dmso	control	0,25458527	8,06095344	0,01818698	0,07369172
TRINITY_DN5713_c0_g1_i1	dmso	control	0,25451611	7,61476848	0,0199894	0,07921724
TRINITY_DN4987_c0_g1_i1	dmso	control	0,25257169	8,84323495	0,01908479	0,07640794
TRINITY_DN10749_c0_g1_i1	dmso	control	0,24847981	7,77207779	0,02599974	0,09633377
TRINITY_DN142_c0_g1_i1	dmso	control	0,24308572	9,8683303	0,02209903	0,08519362
TRINITY_DN2116_c0_g1_i1	dmso	control	0,24221709	8,57732943	0,02439813	0,09173112
TRINITY_DN814_c0_g1_i1	dmso	control	0,24178869	8,43809543	0,02381587	0,09031772
TRINITY_DN934_c0_g1_i1	dmso	control	0,23806331	10,8164713	0,02389258	0,09053735
TRINITY_DN18733_c0_g1_i1	dmso	control	-0,2440764	10,0508966	0,01928271	0,07698695

TRINITY_DN857_c0_g1_i1	dmso	control	-0,2452159	8,44374781	0,0244811	0,09194733
TRINITY_DN12851_c0_g1_i1	dmso	control	-0,2482612	7,67736712	0,02232458	0,08594843
TRINITY_DN10497_c0_g1_i1	dmso	control	-0,249637	7,23707394	0,02321545	0,08846498
TRINITY_DN18616_c0_g1_i1	dmso	control	-0,2497252	7,72829893	0,02316775	0,08837174
TRINITY_DN10693_c0_g1_i1	dmso	control	-0,2515492	8,48247758	0,01809533	0,07338209
TRINITY_DN5743_c0_g1_i1	dmso	control	-0,2515577	9,87156222	0,0164725	0,06814009
TRINITY_DN4840_c0_g1_i1	dmso	control	-0,2527918	7,92676547	0,02636836	0,09731502
TRINITY_DN18769_c0_g1_i1	dmso	control	-0,2535754	8,48294056	0,02628556	0,09709414
TRINITY_DN1466_c0_g1_i1	dmso	control	-0,2556951	8,45021196	0,01714342	0,07037131
TRINITY_DN274_c0_g1_i1	dmso	control	-0,255792	8,22045373	0,01727148	0,07077631
TRINITY_DN13534_c0_g1_i1	dmso	control	-0,2562141	8,64475983	0,02105378	0,08232873
TRINITY_DN4996_c0_g1_i1	dmso	control	-0,259277	9,87075007	0,01549176	0,0649386
TRINITY_DN5755_c0_g1_i1	dmso	control	-0,2600302	6,73881241	0,02313996	0,0883109
TRINITY_DN4770_c0_g1_i1	dmso	control	-0,2610746	6,51500432	0,02425661	0,09135372
TRINITY_DN11307_c0_g1_i1	dmso	control	-0,2613639	7,85133741	0,02713178	0,0995981
TRINITY_DN11188_c0_g1_i1	dmso	control	-0,261724	8,30892228	0,02154623	0,08371039
TRINITY_DN19135_c0_g1_i1	dmso	control	-0,2625725	7,02988929	0,02685031	0,09869027
TRINITY_DN412_c0_g1_i1	dmso	control	-0,2627249	6,57312995	0,02395725	0,09068722
TRINITY_DN10819_c0_g1_i1	dmso	control	-0,2629512	7,0290461	0,02208071	0,08514571
TRINITY_DN34151_c0_g1_i1	dmso	control	-0,2630556	9,68696219	0,0220499	0,08507234
TRINITY_DN32309_c0_g1_i1	dmso	control	-0,2635182	12,3337137	0,01917927	0,0766321
TRINITY_DN1868_c0_g1_i1	dmso	control	-0,2647501	8,68613697	0,01686838	0,06950809
TRINITY_DN5280_c0_g1_i1	dmso	control	-0,2662445	6,4807732	0,0215263	0,08368758
TRINITY_DN5673_c0_g1_i1	dmso	control	-0,2680302	7,48799987	0,02198913	0,0849059
TRINITY_DN18731_c0_g1_i1	dmso	control	-0,2698168	8,26193735	0,01342813	0,05851002
TRINITY_DN18_c0_g1_i1	dmso	control	-0,270655	8,4319754	0,01328465	0,05802745
TRINITY_DN743_c0_g1_i1	dmso	control	-0,2721281	8,3161998	0,02261926	0,08682839
TRINITY_DN20631_c0_g1_i1	dmso	control	-0,2725878	8,18874565	0,01407787	0,0603785
TRINITY_DN19091_c0_g1_i1	dmso	control	-0,2728898	6,26051231	0,02279022	0,08725278
TRINITY_DN6182_c0_g1_i1	dmso	control	-0,2728911	6,52862993	0,02557761	0,09512579
TRINITY_DN330_c0_g1_i1	dmso	control	-0,2736397	6,55132256	0,0182769	0,07393163

TRINITY_DN5634_c0_g1_i1	dmso	control	-0,2740943	6,98147911	0,01604452	0,06684831
TRINITY_DN11441_c0_g1_i1	dmso	control	-0,2745707	9,50246124	0,02285126	0,08741697
TRINITY_DN174_c0_g1_i1	dmso	control	-0,2752255	10,0463488	0,02376388	0,09016791
TRINITY_DN467_c0_g1_i1	dmso	control	-0,2761622	8,64027051	0,02166343	0,08407531
TRINITY_DN11094_c0_g1_i1	dmso	control	-0,2772285	6,98613891	0,02409258	0,09100101
TRINITY_DN18948_c0_g1_i1	dmso	control	-0,2787889	7,46860431	0,02129717	0,08298828
TRINITY_DN32509_c0_g1_i1	dmso	control	-0,2794529	9,9143461	0,01004599	0,04659897
TRINITY_DN10954_c0_g1_i1	dmso	control	-0,2795544	7,16902094	0,01308438	0,05736083
TRINITY_DN334_c0_g1_i1	dmso	control	-0,2813461	9,7029019	0,01030758	0,04756965
TRINITY_DN10914_c0_g1_i1	dmso	control	-0,281348	5,92208096	0,02398136	0,09075471
TRINITY_DN1253_c0_g1_i1	dmso	control	-0,2817424	6,92051398	0,01288993	0,05680141
TRINITY_DN10831_c0_g1_i1	dmso	control	-0,2819154	6,76595344	0,01289924	0,05682509
TRINITY_DN11907_c0_g1_i1	dmso	control	-0,2820751	6,6685154	0,01917981	0,0766321
TRINITY_DN19548_c0_g1_i1	dmso	control	-0,2839919	7,81345932	0,02181147	0,0845136
TRINITY_DN12952_c0_g1_i1	dmso	control	-0,2840908	6,60577922	0,01598417	0,06669306
TRINITY_DN997_c0_g1_i1	dmso	control	-0,2852453	7,41296049	0,01894522	0,07599657
TRINITY_DN18896_c0_g1_i1	dmso	control	-0,2858379	6,41398601	0,01624331	0,06752076
TRINITY_DN2039_c0_g1_i1	dmso	control	-0,2858584	6,42858826	0,02025198	0,07997263
TRINITY_DN5533_c0_g1_i1	dmso	control	-0,2866046	6,48826444	0,01384065	0,05964445
TRINITY_DN11416_c0_g1_i1	dmso	control	-0,2870462	7,35463041	0,01313598	0,05751718
TRINITY_DN11702_c0_g1_i1	dmso	control	-0,2870989	6,96931925	0,01292462	0,05690221
TRINITY_DN5040_c0_g1_i1	dmso	control	-0,2872383	7,41257497	0,00861324	0,04132124
TRINITY_DN10698_c0_g1_i1	dmso	control	-0,2884027	7,04442437	0,01406283	0,06033191
TRINITY_DN151_c0_g1_i1	dmso	control	-0,2885248	6,83161669	0,01438971	0,06129318
TRINITY_DN466_c0_g1_i1	dmso	control	-0,2890171	6,90984058	0,013031	0,05716148
TRINITY_DN6253_c0_g1_i1	dmso	control	-0,2892653	9,36599956	0,00946439	0,04451746
TRINITY_DN5497_c0_g1_i1	dmso	control	-0,2905031	6,21189114	0,01783865	0,0725653
TRINITY_DN1119_c0_g1_i1	dmso	control	-0,2907067	7,48657917	0,00968917	0,04533852
TRINITY_DN322_c0_g1_i1	dmso	control	-0,2914753	9,53152887	0,00817669	0,03972831
TRINITY_DN11798_c0_g1_i1	dmso	control	-0,2919088	5,84798037	0,01954303	0,07783266
TRINITY_DN10763_c0_g1_i1	dmso	control	-0,2919827	7,16099632	0,01930961	0,07707302

TRINITY_DN1188_c0_g1_i1	dmso	control	-0,2920277	6,74581203	0,02090683	0,08186477
TRINITY_DN4667_c0_g1_i1	dmso	control	-0,2938182	6,97547747	0,02329788	0,08869509
TRINITY_DN10701_c0_g1_i1	dmso	control	-0,2939526	7,57051133	0,0233675	0,08889744
TRINITY_DN5077_c0_g1_i1	dmso	control	-0,2944679	7,83120604	0,01039532	0,04789806
TRINITY_DN124_c0_g1_i1	dmso	control	-0,2954684	7,56665774	0,01103562	0,05022356
TRINITY_DN1973_c0_g1_i1	dmso	control	-0,2954852	7,19448912	0,00932598	0,0439437
TRINITY_DN5466_c0_g1_i1	dmso	control	-0,2958591	8,87230382	0,01532732	0,0644363
TRINITY_DN13612_c0_g1_i1	dmso	control	-0,2964299	6,80371857	0,0177285	0,07223928
TRINITY_DN2491_c0_g1_i1	dmso	control	-0,2966006	6,52291949	0,01002938	0,04658343
TRINITY_DN19939_c0_g1_i1	dmso	control	-0,2967185	7,23799588	0,01748957	0,071528
TRINITY_DN18682_c0_g1_i1	dmso	control	-0,2971445	5,56689821	0,02584458	0,09587184
TRINITY_DN2978_c0_g1_i1	dmso	control	-0,2973221	6,13924751	0,02263475	0,08684765
TRINITY_DN6133_c0_g1_i1	dmso	control	-0,2974656	8,06428878	0,01025929	0,04739987
TRINITY_DN12234_c0_g1_i1	dmso	control	-0,2978747	6,4134587	0,01131257	0,05122572
TRINITY_DN9262_c0_g1_i1	dmso	control	-0,2988455	6,69860273	0,01168726	0,05247825
TRINITY_DN9_c0_g1_i1	dmso	control	-0,2991191	11,1871802	0,00435096	0,02420085
TRINITY_DN5045_c0_g1_i1	dmso	control	-0,2997552	9,21148277	0,00707167	0,03538282
TRINITY_DN561_c0_g1_i1	dmso	control	-0,2999893	10,3387362	0,00640472	0,03273751
TRINITY_DN10884_c0_g1_i1	dmso	control	-0,3013998	6,16416326	0,01225201	0,0545394
TRINITY_DN3177_c0_g1_i1	dmso	control	-0,301543	7,66053826	0,01873075	0,07532453
TRINITY_DN32524_c0_g1_i1	dmso	control	-0,3017391	9,96545891	0,01029678	0,04753499
TRINITY_DN9171_c0_g1_i1	dmso	control	-0,3021216	6,76641021	0,01621903	0,06745718
TRINITY_DN5557_c0_g1_i1	dmso	control	-0,3025114	8,30102247	0,00907372	0,04297374
TRINITY_DN468_c0_g1_i1	dmso	control	-0,3025688	6,74824259	0,02446028	0,09189302
TRINITY_DN19644_c0_g1_i1	dmso	control	-0,3027294	6,39407036	0,01135616	0,05135873
TRINITY_DN21735_c0_g1_i1	dmso	control	-0,3031312	6,92082677	0,01154369	0,05196279
TRINITY_DN1364_c0_g1_i1	dmso	control	-0,3034888	5,52636981	0,02275363	0,08718201
TRINITY_DN6401_c0_g1_i1	dmso	control	-0,3035994	6,46528433	0,01366082	0,05913412
TRINITY_DN3741_c0_g1_i1	dmso	control	-0,3042657	5,49900175	0,02259764	0,08681377
TRINITY_DN18657_c0_g1_i1	dmso	control	-0,3044098	6,57907794	0,01252774	0,05559547
TRINITY_DN12252_c0_g1_i1	dmso	control	-0,3050457	6,30268134	0,02180028	0,08449291

TRINITY_DN12059_c0_g1_i1	dmso	control	-0,3051717	6,72125595	0,01165809	0,05237981
TRINITY_DN1005_c0_g1_i1	dmso	control	-0,3056174	6,64107652	0,00820768	0,03981988
TRINITY_DN42_c0_g1_i1	dmso	control	-0,3056939	8,77673038	0,00543344	0,02883398
TRINITY_DN18776_c0_g1_i1	dmso	control	-0,3064371	5,89420942	0,01686312	0,06950809
TRINITY_DN20071_c0_g1_i1	dmso	control	-0,3079206	7,37844098	0,00499498	0,02691252
TRINITY_DN3168_c0_g1_i1	dmso	control	-0,3079484	7,95593649	0,00874356	0,04179377
TRINITY_DN18807_c0_g1_i1	dmso	control	-0,3081854	8,17310458	0,01140712	0,05154078
TRINITY_DN18689_c0_g1_i1	dmso	control	-0,3088139	11,5138528	0,00377177	0,0216633
TRINITY_DN11626_c0_g1_i1	dmso	control	-0,3094463	7,17519289	0,00530059	0,0282431
TRINITY_DN5632_c0_g1_i1	dmso	control	-0,3094518	5,81214578	0,02072841	0,08129836
TRINITY_DN4975_c0_g1_i1	dmso	control	-0,3095027	6,49860195	0,00872513	0,04173327
TRINITY_DN19034_c0_g1_i1	dmso	control	-0,309539	6,11649889	0,01782002	0,07253034
TRINITY_DN5425_c0_g1_i1	dmso	control	-0,3097465	5,63917466	0,0166382	0,06872714
TRINITY_DN32712_c0_g1_i1	dmso	control	-0,3098654	7,08337612	0,00625902	0,0321635
TRINITY_DN11317_c0_g1_i1	dmso	control	-0,3112943	6,55912088	0,00921989	0,04352332
TRINITY_DN5212_c0_g1_i1	dmso	control	-0,3115997	6,68053073	0,01993655	0,07902951
TRINITY_DN19608_c0_g1_i1	dmso	control	-0,3118336	6,6079754	0,01114033	0,05058853
TRINITY_DN2068_c0_g1_i1	dmso	control	-0,3119832	6,04253555	0,01346924	0,0586213
TRINITY_DN729_c0_g1_i1	dmso	control	-0,312503	6,29387023	0,0131563	0,05758873
TRINITY_DN4907_c0_g1_i1	dmso	control	-0,3125281	6,1287278	0,02565757	0,0953005
TRINITY_DN1281_c0_g1_i1	dmso	control	-0,3126519	5,98721874	0,0154488	0,06479616
TRINITY_DN61314_c0_g1_i1	dmso	control	-0,313114	6,26112585	0,01517252	0,06393436
TRINITY_DN11727_c0_g1_i1	dmso	control	-0,313685	5,49511217	0,01771216	0,07219305
TRINITY_DN6767_c0_g1_i1	dmso	control	-0,3137139	6,89063255	0,01059288	0,04862221
TRINITY_DN116_c0_g1_i1	dmso	control	-0,3150806	8,3422909	0,00620457	0,03197469
TRINITY_DN12243_c0_g1_i1	dmso	control	-0,3165019	5,69273705	0,0134693	0,0586213
TRINITY_DN32338_c0_g1_i1	dmso	control	-0,3168638	5,51733871	0,02218467	0,08547816
TRINITY_DN11331_c0_g1_i1	dmso	control	-0,3184794	8,20129822	0,00367133	0,02122991
TRINITY_DN11695_c0_g1_i1	dmso	control	-0,3185772	5,91268232	0,02271035	0,08703928
TRINITY_DN1303_c0_g1_i1	dmso	control	-0,3187357	5,46931146	0,02406442	0,09094024
TRINITY_DN446_c0_g1_i1	dmso	control	-0,3188874	6,07969692	0,0136896	0,05920548

TRINITY_DN19085_c0_g1_i1	dmso	control	-0,3190118	8,04338507	0,01096194	0,04998269
TRINITY_DN19265_c0_g1_i1	dmso	control	-0,3191489	6,35942952	0,01396775	0,06000607
TRINITY_DN11785_c0_g1_i1	dmso	control	-0,3200105	6,44871452	0,00582799	0,03043578
TRINITY_DN10759_c0_g1_i1	dmso	control	-0,3206041	8,21671594	0,00278522	0,01704045
TRINITY_DN6899_c0_g1_i1	dmso	control	-0,3211435	5,70339019	0,01984118	0,07878116
TRINITY_DN5244_c0_g1_i1	dmso	control	-0,3215044	5,63030387	0,01322498	0,05780177
TRINITY_DN2133_c0_g1_i1	dmso	control	-0,3217296	6,81391235	0,0099208	0,04618306
TRINITY_DN20125_c0_g1_i1	dmso	control	-0,3219696	6,21232986	0,00645577	0,03294019
TRINITY_DN18618_c0_g1_i1	dmso	control	-0,3221296	6,53488672	0,00991436	0,04617222
TRINITY_DN10906_c0_g1_i1	dmso	control	-0,3228464	5,48262888	0,02330203	0,08869509
TRINITY_DN12512_c0_g1_i1	dmso	control	-0,3239407	5,60322341	0,01282123	0,05653315
TRINITY_DN12830_c0_g1_i1	dmso	control	-0,3240022	5,18055107	0,02127124	0,08292571
TRINITY_DN470_c0_g1_i1	dmso	control	-0,3244063	8,0859966	0,00342564	0,02011519
TRINITY_DN18744_c0_g1_i1	dmso	control	-0,3249485	6,65480008	0,00516233	0,02763896
TRINITY_DN12901_c0_g1_i1	dmso	control	-0,3250811	6,47276833	0,0066515	0,03368897
TRINITY_DN11018_c0_g1_i1	dmso	control	-0,3251398	9,32240723	0,00683623	0,03439545
TRINITY_DN5508_c0_g1_i1	dmso	control	-0,3255204	7,23145078	0,0036494	0,0211539
TRINITY_DN5676_c0_g1_i1	dmso	control	-0,325744	5,43459703	0,0214788	0,08358092
TRINITY_DN5059_c0_g1_i1	dmso	control	-0,3270239	6,85377476	0,00400664	0,02272315
TRINITY_DN696_c0_g1_i1	dmso	control	-0,3272667	10,5581823	0,00248619	0,01556698
TRINITY_DN18821_c0_g1_i1	dmso	control	-0,32747	6,53319413	0,00462031	0,02541521
TRINITY_DN10692_c0_g1_i1	dmso	control	-0,3275389	8,39415891	0,00254446	0,01584652
TRINITY_DN1716_c0_g1_i1	dmso	control	-0,3277334	7,43112652	0,00463691	0,02549226
TRINITY_DN2475_c0_g1_i1	dmso	control	-0,3283583	5,47678187	0,01361145	0,05899869
TRINITY_DN18764_c0_g1_i1	dmso	control	-0,3286435	7,96965134	0,00398281	0,02260579
TRINITY_DN74_c0_g1_i1	dmso	control	-0,3287168	6,16905002	0,01269362	0,05617627
TRINITY_DN616_c0_g1_i1	dmso	control	-0,3288003	6,15044145	0,00592515	0,03079844
TRINITY_DN19839_c0_g1_i1	dmso	control	-0,3293518	5,34823534	0,02003519	0,07935517
TRINITY_DN32666_c0_g1_i1	dmso	control	-0,3302566	5,86392893	0,02394684	0,09067161
TRINITY_DN5902_c0_g1_i1	dmso	control	-0,3304288	6,37445892	0,00772875	0,0379218
TRINITY_DN18670_c0_g1_i1	dmso	control	-0,3304644	7,00091901	0,01370421	0,0592509

TRINITY_DN35128_c0_g1_i1	dmso	control	-0,3308595	7,10489727	0,00684107	0,03440783
TRINITY_DN19989_c0_g1_i1	dmso	control	-0,3322392	5,04613597	0,02461065	0,09236186
TRINITY_DN4642_c0_g1_i1	dmso	control	-0,3324171	7,57802456	0,00379398	0,02175625
TRINITY_DN5569_c0_g1_i1	dmso	control	-0,3329874	5,46150618	0,01374712	0,05936536
TRINITY_DN287_c0_g1_i1	dmso	control	-0,3335054	9,60939176	0,00147044	0,01021671
TRINITY_DN32312_c0_g1_i1	dmso	control	-0,3342958	8,51776976	0,00883583	0,0421372
TRINITY_DN1667_c0_g1_i1	dmso	control	-0,3361619	5,64134328	0,00858611	0,04121847
TRINITY_DN18675_c0_g1_i1	dmso	control	-0,3362525	5,50594446	0,02325752	0,08857235
TRINITY_DN11689_c0_g1_i1	dmso	control	-0,3367445	5,34172908	0,02112303	0,08253236
TRINITY_DN18753_c0_g1_i1	dmso	control	-0,337457	10,8191746	0,00271438	0,0166635
TRINITY_DN8736_c0_g1_i1	dmso	control	-0,3385828	6,26876798	0,00439073	0,02440326
TRINITY_DN145_c0_g1_i1	dmso	control	-0,3386991	6,89971919	0,0026789	0,01650885
TRINITY_DN5348_c0_g1_i1	dmso	control	-0,338767	6,76378524	0,01862633	0,07501273
TRINITY_DN18813_c0_g1_i1	dmso	control	-0,3398256	7,48385372	0,00351409	0,02055934
TRINITY_DN18686_c0_g1_i1	dmso	control	-0,3399514	5,91204025	0,00624112	0,03210577
TRINITY_DN19375_c0_g1_i1	dmso	control	-0,3400086	6,17186675	0,01158941	0,05215236
TRINITY_DN7472_c0_g1_i1	dmso	control	-0,3404805	5,3926856	0,0158759	0,06629875
TRINITY_DN1581_c0_g1_i1	dmso	control	-0,340876	5,83456051	0,00805551	0,03922561
TRINITY_DN19203_c0_g1_i1	dmso	control	-0,3415729	5,6587547	0,00801807	0,03908913
TRINITY_DN37632_c0_g1_i1	dmso	control	-0,341646	6,61268633	0,00556482	0,02933717
TRINITY_DN7731_c0_g1_i1	dmso	control	-0,3419133	4,83269791	0,02561536	0,09521716
TRINITY_DN949_c0_g1_i1	dmso	control	-0,3421488	5,86359859	0,00630561	0,03231092
TRINITY_DN18697_c0_g1_i1	dmso	control	-0,3424998	5,81331027	0,00755746	0,03729675
TRINITY_DN14621_c0_g1_i1	dmso	control	-0,3425882	6,24763622	0,00384708	0,02199964
TRINITY_DN976_c0_g1_i1	dmso	control	-0,3426317	5,53798324	0,00892793	0,04246427
TRINITY_DN11361_c0_g1_i1	dmso	control	-0,3426469	6,20174367	0,00403508	0,02283066
TRINITY_DN11143_c0_g1_i1	dmso	control	-0,3428297	6,15705895	0,01390004	0,05975775
TRINITY_DN19195_c0_g1_i1	dmso	control	-0,3433052	6,25646314	0,00385024	0,02200896
TRINITY_DN6511_c0_g1_i1	dmso	control	-0,3433363	4,8846061	0,02675374	0,09846058
TRINITY_DN6313_c0_g1_i1	dmso	control	-0,3434415	5,64846307	0,00855726	0,04109913
TRINITY_DN647_c0_g1_i1	dmso	control	-0,3435102	10,7599873	0,00168574	0,01141077

TRINITY_DN12332_c0_g1_i1	dmso	control	-0,3447844	6,29413936	0,0051852	0,02772027
TRINITY_DN5698_c0_g1_i1	dmso	control	-0,3448968	5,79828376	0,00781015	0,03825621
TRINITY_DN8616_c0_g1_i1	dmso	control	-0,3452231	6,37543295	0,0069057	0,03466497
TRINITY_DN268_c0_g1_i1	dmso	control	-0,3456777	5,8698596	0,00850269	0,04092682
TRINITY_DN4700_c0_g1_i1	dmso	control	-0,3460988	10,2613805	0,0083836	0,04050206
TRINITY_DN6911_c0_g1_i1	dmso	control	-0,3474846	7,45619284	0,00215063	0,01387486
TRINITY_DN20670_c0_g1_i1	dmso	control	-0,3476405	5,73200068	0,00774701	0,03799848
TRINITY_DN1508_c0_g1_i1	dmso	control	-0,347905	7,32705322	0,00181512	0,01211058
TRINITY_DN4956_c0_g1_i1	dmso	control	-0,3483361	4,58792939	0,02467559	0,09255748
TRINITY_DN10733_c0_g1_i1	dmso	control	-0,3489052	7,13906714	0,00175618	0,01180457
TRINITY_DN2094_c0_g1_i1	dmso	control	-0,3489211	4,66442094	0,02543104	0,09470259
TRINITY_DN6267_c0_g1_i1	dmso	control	-0,3489988	6,25773661	0,00477315	0,02603514
TRINITY_DN1240_c0_g1_i1	dmso	control	-0,3499108	6,80454592	0,00726897	0,03619451
TRINITY_DN1353_c0_g1_i1	dmso	control	-0,3504045	5,62352229	0,01174459	0,05267016
TRINITY_DN10775_c0_g1_i1	dmso	control	-0,3504131	8,64091954	0,00189551	0,0125484
TRINITY_DN5243_c0_g1_i1	dmso	control	-0,3512806	6,00592374	0,00423905	0,0237062
TRINITY_DN4769_c0_g1_i1	dmso	control	-0,3513456	6,42188908	0,00382854	0,02191098
TRINITY_DN4605_c0_g1_i1	dmso	control	-0,3513997	5,66097731	0,00791835	0,0387219
TRINITY_DN33035_c0_g1_i1	dmso	control	-0,3517492	4,58903839	0,02321708	0,08846498
TRINITY_DN6530_c0_g1_i1	dmso	control	-0,3518741	6,24918487	0,0028098	0,017169
TRINITY_DN4881_c0_g1_i1	dmso	control	-0,3522085	6,83730775	0,00214022	0,01382622
TRINITY_DN18806_c0_g1_i1	dmso	control	-0,352297	5,739618	0,00613341	0,03165312
TRINITY_DN793_c0_g1_i1	dmso	control	-0,3532739	7,25886774	0,00183	0,01219298
TRINITY_DN11636_c0_g1_i1	dmso	control	-0,3533543	5,20701199	0,01363788	0,05907022
TRINITY_DN4728_c0_g1_i1	dmso	control	-0,353632	6,38682368	0,00416948	0,02338958
TRINITY_DN5209_c0_g1_i1	dmso	control	-0,3551804	5,51464213	0,0089933	0,04266278
TRINITY_DN2072_c0_g1_i1	dmso	control	-0,3565806	7,78023431	0,00407371	0,02295932
TRINITY_DN32794_c0_g1_i1	dmso	control	-0,3568827	5,02110661	0,01298667	0,05703633
TRINITY_DN819_c0_g1_i1	dmso	control	-0,356917	6,19084236	0,00362565	0,02105003
TRINITY_DN4707_c0_g1_i1	dmso	control	-0,3569554	7,75683227	0,00155355	0,01068485
TRINITY_DN10785_c0_g1_i1	dmso	control	-0,357448	9,60448686	0,00119513	0,00860174

TRINITY_DN5913_c0_g1_i1	dmso	control	-0,3591628	6,29206992	0,00302613	0,01824394
TRINITY_DN12380_c0_g1_i1	dmso	control	-0,359616	5,93251664	0,01161362	0,05220506
TRINITY_DN2259_c0_g1_i1	dmso	control	-0,3596758	6,08522503	0,00405085	0,02287799
TRINITY_DN10840_c0_g1_i1	dmso	control	-0,3601717	9,22607552	0,0008335	0,00637633
TRINITY_DN4888_c0_g1_i1	dmso	control	-0,3601825	9,14933719	0,00096585	0,00716571
TRINITY_DN1834_c0_g1_i1	dmso	control	-0,3602355	5,15208721	0,01523003	0,06410192
TRINITY_DN13379_c0_g1_i1	dmso	control	-0,3608193	5,20491864	0,01381866	0,05958523
TRINITY_DN6461_c0_g1_i1	dmso	control	-0,3611294	9,07130185	0,00110253	0,00805088
TRINITY_DN5893_c0_g1_i1	dmso	control	-0,3615032	5,09428118	0,01383278	0,05962833
TRINITY_DN453_c0_g1_i1	dmso	control	-0,3619675	4,91029861	0,02237931	0,08613619
TRINITY_DN32556_c0_g1_i1	dmso	control	-0,3628451	10,6145112	0,01016903	0,04706458
TRINITY_DN12005_c0_g1_i1	dmso	control	-0,3628514	5,20264528	0,01226247	0,05456914
TRINITY_DN5314_c0_g1_i1	dmso	control	-0,3635998	5,34808761	0,00910119	0,04303328
TRINITY_DN20089_c0_g1_i1	dmso	control	-0,3639081	5,1384576	0,01702895	0,06996108
TRINITY_DN32605_c0_g1_i1	dmso	control	-0,364035	6,37469581	0,00491394	0,02663484
TRINITY_DN19609_c0_g1_i1	dmso	control	-0,3642588	6,0640794	0,00461435	0,02539213
TRINITY_DN4105_c0_g1_i1	dmso	control	-0,3644765	5,53809747	0,00687723	0,03456562
TRINITY_DN11203_c0_g1_i1	dmso	control	-0,3650097	4,96617486	0,02489513	0,09321155
TRINITY_DN18872_c0_g1_i1	dmso	control	-0,3651621	7,2272029	0,00470555	0,02577192
TRINITY_DN11110_c0_g1_i1	dmso	control	-0,3658637	5,37588215	0,00815775	0,03966296
TRINITY_DN19175_c0_g1_i1	dmso	control	-0,3659842	9,79537487	0,00323522	0,01927124
TRINITY_DN18750_c0_g1_i1	dmso	control	-0,3661947	5,9877111	0,00397106	0,02257454
TRINITY_DN32409_c0_g1_i1	dmso	control	-0,3662041	6,62548534	0,00270403	0,01660701
TRINITY_DN5384_c0_g1_i1	dmso	control	-0,3666266	6,45636434	0,01687288	0,06950809
TRINITY_DN18876_c0_g1_i1	dmso	control	-0,3667141	7,08230406	0,00163389	0,01110726
TRINITY_DN10734_c0_g1_i1	dmso	control	-0,3671306	6,5759627	0,00403243	0,02283066
TRINITY_DN20570_c0_g1_i1	dmso	control	-0,3684931	5,91904666	0,00848286	0,04085857
TRINITY_DN1905_c0_g1_i1	dmso	control	-0,368603	4,36662378	0,02369385	0,08994946
TRINITY_DN4969_c0_g1_i1	dmso	control	-0,3694627	6,89926667	0,02723571	0,09990346
TRINITY_DN55_c0_g1_i1	dmso	control	-0,3696484	4,92435727	0,01458352	0,06190857
TRINITY_DN5542_c0_g1_i1	dmso	control	-0,3707799	6,76342627	0,00137661	0,00966944

TRINITY_DN7003_c0_g1_i1	dmso	control	-0,3708469	5,97743805	0,00415045	0,02330092
TRINITY_DN753_c0_g1_i1	dmso	control	-0,370965	6,84690924	0,00227086	0,01443773
TRINITY_DN11370_c0_g1_i1	dmso	control	-0,3711487	6,44547638	0,00338493	0,01990819
TRINITY_DN4921_c0_g1_i1	dmso	control	-0,3714748	9,38565269	0,005919	0,03077774
TRINITY_DN21356_c0_g1_i1	dmso	control	-0,3714794	5,18259414	0,0149181	0,06312618
TRINITY_DN25409_c0_g1_i1	dmso	control	-0,3715217	5,62125113	0,02380993	0,09031772
TRINITY_DN20744_c0_g1_i1	dmso	control	-0,372013	6,57268362	0,00173791	0,01169808
TRINITY_DN10736_c0_g1_i1	dmso	control	-0,3720713	8,83972959	0,00113737	0,00825597
TRINITY_DN11738_c0_g1_i1	dmso	control	-0,3722596	6,29258406	0,00492044	0,02666007
TRINITY_DN10899_c0_g1_i1	dmso	control	-0,3724367	10,2833556	0,00518355	0,02772027
TRINITY_DN11230_c0_g1_i1	dmso	control	-0,3724764	6,9401293	0,00209742	0,01356187
TRINITY_DN5231_c0_g1_i1	dmso	control	-0,372571	5,30862798	0,0135656	0,05890057
TRINITY_DN199_c0_g1_i1	dmso	control	-0,3727539	10,4536995	0,0042735	0,02384347
TRINITY_DN18992_c0_g1_i1	dmso	control	-0,3727746	6,23319195	0,00331381	0,01960997
TRINITY_DN11879_c0_g1_i1	dmso	control	-0,3728246	5,43185527	0,00576797	0,03019874
TRINITY_DN11391_c0_g1_i1	dmso	control	-0,3733931	5,09697318	0,01123762	0,05093422
TRINITY_DN18840_c0_g1_i1	dmso	control	-0,373775	6,95486373	0,00245738	0,0154133
TRINITY_DN4104_c0_g1_i1	dmso	control	-0,3739534	4,46631773	0,01914749	0,07659526
TRINITY_DN4453_c0_g1_i1	dmso	control	-0,3740856	5,56885233	0,00673714	0,03400347
TRINITY_DN11189_c0_g1_i1	dmso	control	-0,37416	8,06980702	0,00070698	0,00554412
TRINITY_DN20655_c0_g1_i1	dmso	control	-0,3742978	6,04047027	0,00289145	0,01755653
TRINITY_DN11065_c0_g1_i1	dmso	control	-0,3746861	4,62788205	0,01523577	0,0641074
TRINITY_DN4902_c0_g1_i1	dmso	control	-0,3754351	6,57701125	0,00260164	0,0161221
TRINITY_DN1551_c0_g1_i1	dmso	control	-0,3755395	4,33156508	0,02510789	0,09383176
TRINITY_DN5717_c0_g1_i1	dmso	control	-0,3758172	7,99691993	0,00133017	0,00941418
TRINITY_DN19572_c0_g1_i1	dmso	control	-0,376012	5,42915845	0,01397026	0,06000607
TRINITY_DN6073_c0_g1_i1	dmso	control	-0,3761603	5,33983248	0,0182227	0,07379992
TRINITY_DN25790_c0_g1_i1	dmso	control	-0,376319	6,8893406	0,00190396	0,01258131
TRINITY_DN10858_c0_g1_i1	dmso	control	-0,3769686	5,14070468	0,01080075	0,049435
TRINITY_DN4736_c0_g1_i1	dmso	control	-0,3771734	5,84454236	0,00275245	0,01685425
TRINITY_DN14135_c0_g1_i1	dmso	control	-0,3774932	4,74682552	0,01803097	0,07318273

TRINITY_DN21357_c0_g1_i1	dmso	control	-0,3783012	4,65213766	0,01427371	0,06096504
TRINITY_DN1417_c0_g1_i1	dmso	control	-0,3783443	6,57012777	0,00107575	0,00789597
TRINITY_DN19053_c0_g1_i1	dmso	control	-0,3783475	5,00276517	0,01084434	0,049556
TRINITY_DN11038_c0_g1_i1	dmso	control	-0,3787811	5,31359413	0,00670011	0,03386036
TRINITY_DN761_c0_g1_i1	dmso	control	-0,3790066	7,68167961	0,00057434	0,00466614
TRINITY_DN1261_c0_g1_i1	dmso	control	-0,3790701	7,5687891	0,00058565	0,00474024
TRINITY_DN14576_c0_g1_i1	dmso	control	-0,3797986	6,7768388	0,01355467	0,0588864
TRINITY_DN267_c0_g1_i1	dmso	control	-0,379826	8,17490959	0,00089767	0,00676063
TRINITY_DN4981_c0_g1_i1	dmso	control	-0,3815541	5,82948035	0,00321689	0,01920167
TRINITY_DN10855_c0_g1_i1	dmso	control	-0,3826714	7,55573396	0,00048836	0,00405424
TRINITY_DN12219_c0_g1_i1	dmso	control	-0,3836461	6,68463882	0,0030688	0,01845495
TRINITY_DN4651_c0_g1_i1	dmso	control	-0,3841173	8,58557233	0,00118326	0,00853278
TRINITY_DN33094_c0_g1_i1	dmso	control	-0,3845978	4,8766902	0,02445052	0,09188027
TRINITY_DN15114_c0_g1_i1	dmso	control	-0,3854579	5,26155021	0,0058045	0,03034598
TRINITY_DN11058_c0_g1_i1	dmso	control	-0,3857455	8,73367143	0,00067044	0,0053124
TRINITY_DN5866_c0_g1_i1	dmso	control	-0,3858016	7,21781585	0,00240681	0,01516196
TRINITY_DN5746_c0_g1_i1	dmso	control	-0,3858342	5,94199035	0,00731976	0,03638472
TRINITY_DN8610_c0_g1_i1	dmso	control	-0,3859166	8,46154702	0,00110847	0,00807867
TRINITY_DN227_c0_g1_i1	dmso	control	-0,3862357	7,53695662	0,00162759	0,01107945
TRINITY_DN2030_c0_g1_i1	dmso	control	-0,3869425	6,26382634	0,00131134	0,00931593
TRINITY_DN5473_c0_g1_i1	dmso	control	-0,3869733	5,20574348	0,0069655	0,03489999
TRINITY_DN5087_c0_g1_i1	dmso	control	-0,3871358	10,4521797	0,00019795	0,00186887
TRINITY_DN18356_c0_g1_i1	dmso	control	-0,3872392	4,57181217	0,01262857	0,05593981
TRINITY_DN33651_c0_g1_i1	dmso	control	-0,387319	5,75481298	0,00404563	0,02287247
TRINITY_DN7169_c0_g1_i1	dmso	control	-0,387403	4,37012567	0,01794385	0,07289057
TRINITY_DN10894_c0_g1_i1	dmso	control	-0,3881254	4,87572302	0,01091246	0,04982
TRINITY_DN1650_c0_g1_i1	dmso	control	-0,3882433	4,80430478	0,01387308	0,05970073
TRINITY_DN5627_c0_g1_i1	dmso	control	-0,3898364	4,85226684	0,01133035	0,05129015
TRINITY_DN15556_c0_g1_i1	dmso	control	-0,3900539	5,03258904	0,00744569	0,03683321
TRINITY_DN1194_c0_g1_i1	dmso	control	-0,3903718	7,4328377	0,00042466	0,00359781
TRINITY_DN6026_c0_g1_i1	dmso	control	-0,3907696	6,51270553	0,00177676	0,01190412

TRINITY_DN739_c0_g1_i1	dmso	control	-0,3910451	8,65674509	0,00031924	0,00283434
TRINITY_DN582_c0_g1_i1	dmso	control	-0,3911421	5,9936039	0,00477779	0,02603514
TRINITY_DN4511_c0_g1_i1	dmso	control	-0,3911883	4,96567623	0,01606637	0,06692004
TRINITY_DN2041_c0_g1_i1	dmso	control	-0,3912327	6,38727388	0,00093365	0,00696261
TRINITY_DN708_c0_g1_i1	dmso	control	-0,3914196	6,42130292	0,00156961	0,01077615
TRINITY_DN4637_c0_g1_i1	dmso	control	-0,3915124	6,62011373	0,00093456	0,00696578
TRINITY_DN4163_c0_g1_i1	dmso	control	-0,3916339	5,21670314	0,0104579	0,04808967
TRINITY_DN2113_c0_g1_i1	dmso	control	-0,3916341	7,18112319	0,00041579	0,00353713
TRINITY_DN103_c0_g1_i1	dmso	control	-0,3917785	10,6214797	0,00020949	0,00196119
TRINITY_DN18579_c0_g1_i1	dmso	control	-0,3917905	7,19447435	0,00072047	0,00564072
TRINITY_DN795_c0_g1_i1	dmso	control	-0,392459	7,96055541	0,00225816	0,0143825
TRINITY_DN573_c0_g1_i1	dmso	control	-0,3927861	5,85316346	0,00884494	0,04216673
TRINITY_DN4033_c0_g1_i1	dmso	control	-0,3928417	4,92565439	0,0093272	0,0439437
TRINITY_DN7155_c0_g1_i1	dmso	control	-0,3930229	5,28392844	0,00569115	0,0299158
TRINITY_DN6349_c0_g1_i1	dmso	control	-0,3935155	5,66873736	0,00193573	0,0127272
TRINITY_DN5847_c0_g1_i1	dmso	control	-0,3943848	4,67965772	0,02260883	0,08681377
TRINITY_DN11_c0_g1_i1	dmso	control	-0,3945757	8,55871884	0,00314693	0,01883851
TRINITY_DN11749_c0_g1_i1	dmso	control	-0,394912	4,04657136	0,0251437	0,09390624
TRINITY_DN4711_c0_g1_i1	dmso	control	-0,3953403	5,70923231	0,00220981	0,01416179
TRINITY_DN13204_c0_g1_i1	dmso	control	-0,3958027	5,44936375	0,00407077	0,02295169
TRINITY_DN32299_c0_g1_i1	dmso	control	-0,3959329	8,62002754	0,00281869	0,01720149
TRINITY_DN11648_c0_g1_i1	dmso	control	-0,3962542	6,14935563	0,00187021	0,01239226
TRINITY_DN12139_c0_g1_i1	dmso	control	-0,3965434	4,12539073	0,02523803	0,09417806
TRINITY_DN4723_c0_g1_i1	dmso	control	-0,3971068	9,86311204	0,00398013	0,02259947
TRINITY_DN4608_c0_g1_i1	dmso	control	-0,3978015	5,44867126	0,00328046	0,01947733
TRINITY_DN4771_c0_g1_i1	dmso	control	-0,3983295	8,75472283	0,00030402	0,00271256
TRINITY_DN19138_c0_g1_i1	dmso	control	-0,3984201	7,0387675	0,00038891	0,00333009
TRINITY_DN11644_c0_g1_i1	dmso	control	-0,3986504	6,57804394	0,00153245	0,01056119
TRINITY_DN2775_c0_g1_i1	dmso	control	-0,4000862	4,46964561	0,01836411	0,07418053
TRINITY_DN5589_c0_g1_i1	dmso	control	-0,4006321	5,36328995	0,00595889	0,03093647
TRINITY_DN18912_c0_g1_i1	dmso	control	-0,4014479	5,17573956	0,00471248	0,02579451

TRINITY_DN11284_c0_g1_i1	dmso	control	-0,4018016	6,25385292	0,00151952	0,01048334
TRINITY_DN440_c0_g1_i1	dmso	control	-0,4019708	5,75644996	0,00252346	0,01575257
TRINITY_DN37432_c0_g1_i1	dmso	control	-0,4022926	6,7187865	0,00139313	0,0097641
TRINITY_DN19064_c0_g1_i1	dmso	control	-0,4025025	6,72202571	0,00043876	0,00369853
TRINITY_DN11111_c0_g1_i1	dmso	control	-0,4032023	4,90094416	0,01508465	0,06360583
TRINITY_DN8567_c0_g1_i1	dmso	control	-0,4036232	4,20590568	0,01759538	0,07181839
TRINITY_DN2852_c0_g1_i1	dmso	control	-0,4037703	7,01693821	0,00091895	0,00688036
TRINITY_DN19815_c0_g1_i1	dmso	control	-0,4048265	3,89437983	0,02622561	0,09691197
TRINITY_DN5991_c0_g1_i1	dmso	control	-0,4051791	7,90673148	0,00036154	0,00313425
TRINITY_DN10702_c0_g1_i1	dmso	control	-0,4054982	8,48203933	0,00014437	0,00143034
TRINITY_DN20005_c0_g1_i1	dmso	control	-0,4056057	5,4910927	0,00399114	0,02264415
TRINITY_DN785_c0_g1_i1	dmso	control	-0,4056094	7,79260191	0,00038886	0,00333009
TRINITY_DN4795_c0_g1_i1	dmso	control	-0,4058463	5,36991256	0,00623775	0,03209988
TRINITY_DN4253_c0_g1_i1	dmso	control	-0,4060546	6,73140533	0,00059469	0,0047937
TRINITY_DN12157_c0_g1_i1	dmso	control	-0,4062184	5,00172724	0,00509014	0,02733347
TRINITY_DN11685_c0_g1_i1	dmso	control	-0,4065534	6,86349759	0,00129899	0,00924334
TRINITY_DN4123_c0_g1_i1	dmso	control	-0,4069997	8,63678423	0,00152106	0,01048767
TRINITY_DN13711_c0_g1_i1	dmso	control	-0,4076376	7,50818326	0,00065082	0,00518742
TRINITY_DN6981_c0_g1_i1	dmso	control	-0,4077482	6,62900537	0,00041841	0,00355317
TRINITY_DN5349_c0_g1_i1	dmso	control	-0,4078686	4,24791799	0,01592845	0,06649897
TRINITY_DN20721_c0_g1_i1	dmso	control	-0,4081661	5,15312367	0,00879901	0,04198935
TRINITY_DN19324_c0_g1_i1	dmso	control	-0,4082384	5,2363856	0,00827296	0,0400613
TRINITY_DN7746_c0_g1_i1	dmso	control	-0,4083076	5,39112421	0,00297811	0,01798445
TRINITY_DN10952_c0_g1_i1	dmso	control	-0,4084519	6,75861616	0,00047374	0,0039527
TRINITY_DN11308_c0_g1_i1	dmso	control	-0,4086139	7,18540736	0,0003467	0,0030297
TRINITY_DN19037_c0_g1_i1	dmso	control	-0,4086887	10,2056845	0,00868375	0,04154911
TRINITY_DN1765_c0_g1_i1	dmso	control	-0,4098047	5,47018299	0,00229669	0,01456067
TRINITY_DN11676_c0_g1_i1	dmso	control	-0,4106661	4,66301416	0,02310716	0,08821294
TRINITY_DN510_c0_g1_i1	dmso	control	-0,410741	5,95341344	0,00090006	0,00676678
TRINITY_DN8278_c0_g1_i1	dmso	control	-0,4107566	5,07664001	0,00644722	0,03290816
TRINITY_DN8943_c0_g1_i1	dmso	control	-0,410829	4,55886633	0,01917266	0,0766321

TRINITY_DN13314_c0_g1_i1	dmso	control	-0,4117839	5,63722638	0,0013184	0,00935062
TRINITY_DN852_c0_g1_i1	dmso	control	-0,4119578	6,70485852	0,00122924	0,00882525
TRINITY_DN10991_c0_g1_i1	dmso	control	-0,4123772	8,89054261	0,00108725	0,00795209
TRINITY_DN23616_c0_g1_i1	dmso	control	-0,412403	3,69767237	0,02551736	0,09495061
TRINITY_DN1456_c0_g1_i1	dmso	control	-0,4127563	7,08254709	0,00087836	0,00664636
TRINITY_DN18941_c0_g1_i1	dmso	control	-0,413324	4,81124287	0,00620897	0,03197935
TRINITY_DN21829_c0_g1_i1	dmso	control	-0,4134141	3,92782058	0,02067855	0,08119101
TRINITY_DN28306_c0_g1_i1	dmso	control	-0,4134527	4,08094815	0,01700727	0,06993167
TRINITY_DN20448_c0_g1_i1	dmso	control	-0,4158855	4,09128761	0,01962889	0,07811
TRINITY_DN10761_c0_g1_i1	dmso	control	-0,4168442	6,79124742	0,0024501	0,01538101
TRINITY_DN3101_c0_g1_i1	dmso	control	-0,4180464	4,06059324	0,02062168	0,08103379
TRINITY_DN11751_c0_g1_i1	dmso	control	-0,4182892	4,53126255	0,00692145	0,03471539
TRINITY_DN18956_c0_g1_i1	dmso	control	-0,4184879	6,23590754	0,00034659	0,0030297
TRINITY_DN19189_c0_g1_i1	dmso	control	-0,418866	6,48075901	0,00038799	0,00332608
TRINITY_DN11048_c0_g1_i1	dmso	control	-0,419016	5,70441371	0,00186189	0,01234847
TRINITY_DN18946_c0_g1_i1	dmso	control	-0,4193659	5,24003708	0,00955896	0,04484403
TRINITY_DN15881_c0_g1_i1	dmso	control	-0,4197651	4,3318642	0,01328987	0,05803269
TRINITY_DN5728_c0_g1_i1	dmso	control	-0,4214832	4,85697339	0,01192229	0,0533181
TRINITY_DN6687_c0_g1_i1	dmso	control	-0,4227937	5,16551471	0,00498756	0,02690267
TRINITY_DN17872_c0_g1_i1	dmso	control	-0,4228048	3,82796625	0,02600342	0,09633377
TRINITY_DN3679_c0_g1_i1	dmso	control	-0,4229967	4,29317663	0,01091613	0,04982101
TRINITY_DN2521_c0_g1_i1	dmso	control	-0,4232663	4,97085738	0,00365393	0,02116429
TRINITY_DN6809_c0_g1_i1	dmso	control	-0,4251526	4,78854404	0,01068706	0,04894564
TRINITY_DN20735_c0_g1_i1	dmso	control	-0,4255998	5,0926423	0,00370444	0,02137006
TRINITY_DN4798_c0_g1_i1	dmso	control	-0,4263128	10,0032748	0,00314597	0,01883851
TRINITY_DN19023_c0_g1_i1	dmso	control	-0,4263713	3,99954874	0,01649696	0,06822174
TRINITY_DN14396_c0_g1_i1	dmso	control	-0,4266049	5,14213315	0,00357873	0,02081946
TRINITY_DN10703_c0_g1_i1	dmso	control	-0,4285412	11,9257634	0,00067953	0,00537557
TRINITY_DN4905_c0_g1_i1	dmso	control	-0,4286074	6,8743441	0,00019547	0,00184786
TRINITY_DN10883_c0_g1_i1	dmso	control	-0,4286956	7,58468903	0,00025334	0,00231317
TRINITY_DN218_c0_g1_i1	dmso	control	-0,4289069	8,50108601	0,00021729	0,00202107

TRINITY_DN1579_c0_g1_i1	dmso	control	-0,4289521	5,97295583	0,00128556	0,00917488
TRINITY_DN34091_c0_g1_i1	dmso	control	-0,428967	3,99687437	0,01350233	0,05872965
TRINITY_DN14963_c0_g1_i1	dmso	control	-0,4293787	3,50589677	0,02517782	0,09397767
TRINITY_DN19497_c0_g1_i1	dmso	control	-0,4304639	4,22974569	0,01017207	0,04706458
TRINITY_DN14012_c0_g1_i1	dmso	control	-0,4305463	5,04569749	0,00288046	0,01750452
TRINITY_DN7714_c0_g1_i1	dmso	control	-0,4310484	4,7080969	0,00495522	0,02677823
TRINITY_DN13409_c0_g1_i1	dmso	control	-0,4318292	3,81097473	0,02022318	0,07992443
TRINITY_DN33577_c0_g1_i1	dmso	control	-0,4327081	4,16789658	0,0179025	0,07275289
TRINITY_DN19059_c0_g1_i1	dmso	control	-0,4328849	5,97080806	0,00081834	0,00627108
TRINITY_DN19119_c0_g1_i1	dmso	control	-0,4330534	3,93596425	0,0175764	0,07180179
TRINITY_DN1142_c0_g1_i1	dmso	control	-0,4336558	8,62095622	0,00011343	0,00116451
TRINITY_DN21116_c0_g1_i1	dmso	control	-0,4351251	7,00257513	0,00016294	0,00158174
TRINITY_DN5149_c0_g1_i1	dmso	control	-0,4355027	6,4502271	0,00023732	0,00218771
TRINITY_DN8124_c0_g1_i1	dmso	control	-0,4359931	3,7950285	0,01889585	0,07586173
TRINITY_DN13984_c0_g1_i1	dmso	control	-0,4360401	4,81696767	0,00421731	0,02361201
TRINITY_DN11577_c0_g1_i1	dmso	control	-0,4363093	4,98859005	0,00457928	0,02526649
TRINITY_DN13959_c0_g1_i1	dmso	control	-0,4363531	4,01984791	0,01554924	0,06512287
TRINITY_DN6294_c0_g1_i1	dmso	control	-0,4364739	4,27391055	0,01439297	0,06129318
TRINITY_DN5203_c0_g1_i1	dmso	control	-0,4367538	5,30817409	0,0103614	0,04778747
TRINITY_DN1104_c0_g1_i1	dmso	control	-0,436847	5,55445311	0,00148815	0,01030504
TRINITY_DN1002_c0_g1_i1	dmso	control	-0,4371638	8,44939385	5,79E-05	0,00065474
TRINITY_DN6271_c0_g1_i1	dmso	control	-0,4376587	3,90583589	0,01726697	0,07077631
TRINITY_DN1140_c0_g1_i1	dmso	control	-0,4379674	4,70069046	0,00674048	0,03400849
TRINITY_DN34050_c0_g1_i1	dmso	control	-0,4381688	6,21571983	0,00055376	0,00452437
TRINITY_DN678_c0_g1_i1	dmso	control	-0,4386097	7,16878534	0,00040822	0,00347891
TRINITY_DN6085_c0_g1_i1	dmso	control	-0,4387289	4,84634265	0,00501024	0,02697462
TRINITY_DN32288_c0_g1_i1	dmso	control	-0,4394875	8,28327101	0,00810693	0,03944246
TRINITY_DN6310_c0_g1_i1	dmso	control	-0,4399821	4,1146286	0,01274455	0,05634985
TRINITY_DN5322_c0_g1_i1	dmso	control	-0,4403021	9,07314025	0,0026983	0,01658075
TRINITY_DN6712_c0_g1_i1	dmso	control	-0,4403712	6,8633296	0,00035787	0,00311222
TRINITY_DN4880_c0_g1_i1	dmso	control	-0,4406068	4,96304994	0,00799685	0,03903834

TRINITY_DN6943_c0_g1_i1	dmso	control	-0,4410124	6,1425972	0,00224748	0,01433863
TRINITY_DN8083_c0_g1_i1	dmso	control	-0,441621	4,69979139	0,0037885	0,0217421
TRINITY_DN14710_c0_g1_i1	dmso	control	-0,4420156	3,34847455	0,02480966	0,09296381
TRINITY_DN11271_c0_g1_i1	dmso	control	-0,442253	5,21846395	0,00428652	0,02389765
TRINITY_DN10902_c0_g1_i1	dmso	control	-0,4436179	7,61160411	0,00028789	0,0025878
TRINITY_DN2937_c0_g1_i1	dmso	control	-0,4447326	6,77934232	0,00021041	0,00196723
TRINITY_DN3321_c0_g1_i1	dmso	control	-0,4454241	4,8058933	0,00719673	0,03585951
TRINITY_DN3756_c0_g1_i1	dmso	control	-0,445773	4,51439706	0,0069066	0,03466497
TRINITY_DN6132_c0_g1_i1	dmso	control	-0,446883	5,99367802	0,00125146	0,00896252
TRINITY_DN10919_c0_g1_i1	dmso	control	-0,4477436	4,99538595	0,00241896	0,01521861
TRINITY_DN24141_c0_g1_i1	dmso	control	-0,4478511	3,76279293	0,02141836	0,08338089
TRINITY_DN855_c0_g1_i1	dmso	control	-0,4481739	3,72267315	0,01458462	0,06190857
TRINITY_DN19751_c0_g1_i1	dmso	control	-0,4483568	3,57228832	0,02505191	0,09365292
TRINITY_DN675_c0_g1_i1	dmso	control	-0,4484281	7,47342217	0,00020485	0,00192157
TRINITY_DN32907_c0_g1_i1	dmso	control	-0,4485625	6,0702269	0,0014741	0,01023225
TRINITY_DN3232_c0_g1_i1	dmso	control	-0,4488961	4,21693548	0,00743604	0,03679806
TRINITY_DN5378_c0_g1_i1	dmso	control	-0,4489721	3,83223816	0,01864934	0,07508074
TRINITY_DN750_c0_g1_i1	dmso	control	-0,4497929	7,27161316	4,42E-05	0,00051366
TRINITY_DN9584_c0_g1_i1	dmso	control	-0,4500037	3,91767632	0,01418699	0,06068435
TRINITY_DN13777_c0_g1_i1	dmso	control	-0,4500577	3,55782133	0,02267055	0,08693285
TRINITY_DN11171_c0_g1_i1	dmso	control	-0,450177	6,00756015	0,00053278	0,00438022
TRINITY_DN18721_c0_g1_i1	dmso	control	-0,4508766	7,47192586	3,82E-05	0,00045268
TRINITY_DN2349_c0_g1_i1	dmso	control	-0,4514609	4,06882475	0,00972309	0,04546782
TRINITY_DN8891_c0_g1_i1	dmso	control	-0,4524159	3,56218047	0,01987106	0,07885644
TRINITY_DN3408_c0_g1_i1	dmso	control	-0,4527472	3,48473832	0,02179225	0,08448445
TRINITY_DN12612_c0_g1_i1	dmso	control	-0,4530589	5,92373534	0,00089899	0,00676349
TRINITY_DN1093_c0_g1_i1	dmso	control	-0,4536517	6,77538202	0,00010747	0,00111208
TRINITY_DN5327_c0_g1_i1	dmso	control	-0,4542091	6,24484546	0,00011299	0,00116087
TRINITY_DN12160_c0_g1_i1	dmso	control	-0,4543774	8,20355187	3,60E-05	0,00042971
TRINITY_DN14681_c0_g1_i1	dmso	control	-0,4549418	3,8305282	0,02583431	0,09585835
TRINITY_DN19868_c0_g1_i1	dmso	control	-0,4549584	4,25230646	0,00630311	0,03230956

TRINITY_DN5761_c0_g1_i1	dmso	control	-0,4560932	7,0695004	4,46E-05	0,0005174
TRINITY_DN20662_c0_g1_i1	dmso	control	-0,4573915	4,44392443	0,00464922	0,02551594
TRINITY_DN19081_c0_g1_i1	dmso	control	-0,4577493	6,07375237	0,01481489	0,06276849
TRINITY_DN9556_c0_g1_i1	dmso	control	-0,4586754	3,6344842	0,01807829	0,07333358
TRINITY_DN22586_c0_g1_i1	dmso	control	-0,459027	3,10374563	0,02669284	0,09828652
TRINITY_DN34975_c0_g1_i1	dmso	control	-0,4596244	4,07749189	0,01770407	0,07218042
TRINITY_DN5282_c0_g1_i1	dmso	control	-0,459734	6,60132439	0,00155198	0,01068382
TRINITY_DN12122_c0_g1_i1	dmso	control	-0,4597448	4,59409832	0,0047646	0,02600101
TRINITY_DN4749_c0_g1_i1	dmso	control	-0,4597506	5,99331196	0,00083429	0,00637633
TRINITY_DN10689_c0_g1_i1	dmso	control	-0,4598305	5,24387635	0,00179082	0,01198721
TRINITY_DN19645_c0_g1_i1	dmso	control	-0,4602566	3,69415747	0,01783059	0,07255292
TRINITY_DN363_c0_g1_i1	dmso	control	-0,4612857	7,13018402	0,00357197	0,02079959
TRINITY_DN1843_c0_g1_i1	dmso	control	-0,4617227	7,82356239	6,62E-05	0,00073422
TRINITY_DN19441_c0_g1_i1	dmso	control	-0,4618749	5,34137611	0,0015197	0,01048334
TRINITY_DN7443_c0_g1_i1	dmso	control	-0,4620323	4,18043026	0,00947734	0,04456387
TRINITY_DN243_c0_g1_i1	dmso	control	-0,4621133	5,0864513	0,00280302	0,01713484
TRINITY_DN13419_c0_g1_i1	dmso	control	-0,4622104	3,4531998	0,02054167	0,08080731
TRINITY_DN12995_c0_g1_i1	dmso	control	-0,4626704	3,85963948	0,00983814	0,04591662
TRINITY_DN7455_c0_g1_i1	dmso	control	-0,4631138	4,26941018	0,00546443	0,02895589
TRINITY_DN12700_c0_g1_i1	dmso	control	-0,4631589	3,33581266	0,02119691	0,08276571
TRINITY_DN2289_c0_g1_i1	dmso	control	-0,4642969	4,62537164	0,00390761	0,02227524
TRINITY_DN3289_c0_g1_i1	dmso	control	-0,4648912	5,89031714	0,00061785	0,00495276
TRINITY_DN18984_c0_g1_i1	dmso	control	-0,4655364	9,3027627	0,00083819	0,00640278
TRINITY_DN4027_c0_g1_i1	dmso	control	-0,4655849	6,57980361	0,00012058	0,00122746
TRINITY_DN614_c0_g1_i1	dmso	control	-0,466009	5,43705703	0,00111275	0,00810576
TRINITY_DN7473_c0_g1_i1	dmso	control	-0,4675	5,30136829	0,00096123	0,00713512
TRINITY_DN11215_c0_g1_i1	dmso	control	-0,4677475	5,35897989	0,00058955	0,00476023
TRINITY_DN11612_c0_g1_i1	dmso	control	-0,4685276	3,12400058	0,02152795	0,08368758
TRINITY_DN6381_c0_g1_i1	dmso	control	-0,4693001	5,44718614	0,00143755	0,01002194
TRINITY_DN13086_c0_g1_i1	dmso	control	-0,4698739	3,47666005	0,01580755	0,06605631
TRINITY_DN12943_c0_g1_i1	dmso	control	-0,4712001	4,9612551	0,00174155	0,01171712

TRINITY_DN34594_c0_g1_i1	dmso	control	-0,4713272	4,68253496	0,00489845	0,02657083
TRINITY_DN2487_c0_g1_i1	dmso	control	-0,4721951	4,33501163	0,00401747	0,0227578
TRINITY_DN2562_c0_g1_i1	dmso	control	-0,4722283	3,64570709	0,02199593	0,08490948
TRINITY_DN11163_c0_g1_i1	dmso	control	-0,4722435	7,49978631	0,000101	0,00105415
TRINITY_DN25969_c0_g1_i1	dmso	control	-0,4740444	4,46266397	0,00611545	0,03159178
TRINITY_DN6324_c0_g1_i1	dmso	control	-0,4743351	4,10051845	0,0090845	0,04299039
TRINITY_DN24614_c0_g1_i1	dmso	control	-0,4744942	3,9626836	0,01601403	0,06679836
TRINITY_DN10783_c0_g1_i1	dmso	control	-0,4746635	4,87617503	0,00537593	0,02857078
TRINITY_DN11898_c0_g1_i1	dmso	control	-0,4753016	4,11743118	0,00898387	0,04263518
TRINITY_DN20713_c0_g1_i1	dmso	control	-0,4753438	3,06880315	0,02164685	0,0840561
TRINITY_DN7428_c0_g1_i1	dmso	control	-0,4755494	6,25180109	0,00075574	0,0058818
TRINITY_DN24448_c0_g1_i1	dmso	control	-0,4756128	4,13329362	0,00620019	0,03196349
TRINITY_DN19581_c0_g1_i1	dmso	control	-0,4762295	4,69336168	0,0019015	0,01257799
TRINITY_DN2858_c0_g1_i1	dmso	control	-0,476271	3,73207105	0,0198227	0,07877272
TRINITY_DN13077_c0_g1_i1	dmso	control	-0,4771837	3,82193244	0,0085225	0,04100847
TRINITY_DN689_c0_g1_i1	dmso	control	-0,4773323	7,3203739	2,22E-05	0,00027925
TRINITY_DN1997_c0_g1_i1	dmso	control	-0,4774032	6,19002581	5,84E-05	0,00065891
TRINITY_DN21462_c0_g1_i1	dmso	control	-0,4782492	3,88692209	0,01147684	0,05175863
TRINITY_DN12206_c0_g1_i1	dmso	control	-0,478284	4,24331792	0,00532184	0,02832498
TRINITY_DN12859_c0_g1_i1	dmso	control	-0,478287	5,6938245	0,00096976	0,00719106
TRINITY_DN4214_c0_g1_i1	dmso	control	-0,4790396	6,73774521	2,63E-05	0,00032595
TRINITY_DN18626_c0_g1_i1	dmso	control	-0,4795655	5,5725373	0,00027694	0,00250185
TRINITY_DN19001_c0_g1_i1	dmso	control	-0,4796802	4,47127614	0,00236538	0,01494662
TRINITY_DN11429_c0_g1_i1	dmso	control	-0,4797319	8,98501436	0,00095432	0,00708747
TRINITY_DN32305_c0_g1_i1	dmso	control	-0,4798757	4,6060039	0,00760777	0,0374955
TRINITY_DN4712_c0_g1_i1	dmso	control	-0,4799082	7,7852248	1,22E-05	0,00016705
TRINITY_DN19649_c0_g1_i1	dmso	control	-0,4803251	3,54027262	0,0128177	0,05653315
TRINITY_DN13275_c0_g1_i1	dmso	control	-0,4804117	5,25856464	0,0010823	0,00792986
TRINITY_DN4666_c0_g1_i1	dmso	control	-0,4805778	5,40690618	0,00115075	0,00832791
TRINITY_DN1727_c0_g1_i1	dmso	control	-0,4814302	4,76712708	0,00314543	0,01883851
TRINITY_DN9155_c0_g1_i1	dmso	control	-0,4823042	3,12578043	0,02054815	0,08081078

TRINITY_DN11081_c0_g1_i1	dmso	control	-0,4823201	7,04737471	0,00016287	0,00158174
TRINITY_DN20504_c0_g1_i1	dmso	control	-0,4823932	3,21376576	0,0162656	0,06757452
TRINITY_DN416_c0_g1_i1	dmso	control	-0,4824811	7,85047426	6,83E-05	0,00075465
TRINITY_DN11156_c0_g1_i1	dmso	control	-0,4842097	3,76861145	0,01817108	0,07364795
TRINITY_DN20340_c0_g1_i1	dmso	control	-0,486738	4,22939928	0,00410495	0,02310833
TRINITY_DN3088_c0_g1_i1	dmso	control	-0,4875223	4,02029069	0,00591428	0,03077514
TRINITY_DN604_c0_g1_i1	dmso	control	-0,4877454	5,51696434	0,00028267	0,00254724
TRINITY_DN688_c0_g1_i1	dmso	control	-0,4883829	6,09818166	0,00014404	0,00142902
TRINITY_DN14091_c0_g1_i1	dmso	control	-0,4884071	2,78656879	0,02688654	0,09879829
TRINITY_DN1224_c0_g1_i1	dmso	control	-0,4889009	4,47805437	0,02171388	0,08420322
TRINITY_DN32650_c0_g1_i1	dmso	control	-0,489063	11,5921986	5,60E-05	0,00063497
TRINITY_DN16889_c0_g1_i1	dmso	control	-0,4892084	5,07480027	0,0012585	0,00900398
TRINITY_DN18886_c0_g1_i1	dmso	control	-0,4902123	4,82964986	0,00155409	0,01068485
TRINITY_DN14162_c0_g1_i1	dmso	control	-0,490417	3,89203933	0,00844718	0,04076828
TRINITY_DN32576_c0_g1_i1	dmso	control	-0,4908037	4,15945141	0,00996113	0,046326
TRINITY_DN11596_c0_g1_i1	dmso	control	-0,4914692	5,53888747	0,00254807	0,01585138
TRINITY_DN7088_c0_g1_i1	dmso	control	-0,4922392	4,0867137	0,00967578	0,04529264
TRINITY_DN9658_c0_g1_i1	dmso	control	-0,4923026	4,10617453	0,01199931	0,05361275
TRINITY_DN4842_c0_g1_i1	dmso	control	-0,4937768	6,58632054	0,000917	0,00687039
TRINITY_DN2285_c0_g1_i1	dmso	control	-0,4943471	7,18481547	7,85E-06	0,00011251
TRINITY_DN1489_c0_g1_i1	dmso	control	-0,4946358	3,69687388	0,00896829	0,0426001
TRINITY_DN14314_c0_g1_i1	dmso	control	-0,4948135	4,14597275	0,00387305	0,02210439
TRINITY_DN303_c0_g1_i1	dmso	control	-0,494816	4,79574933	0,00137695	0,00966944
TRINITY_DN18905_c0_g1_i1	dmso	control	-0,4951705	4,72770277	0,00201508	0,01314741
TRINITY_DN32837_c0_g1_i1	dmso	control	-0,4953778	6,45186478	8,52E-05	0,00090999
TRINITY_DN7715_c0_g1_i1	dmso	control	-0,4961864	3,392853	0,01357882	0,05892041
TRINITY_DN27420_c0_g1_i1	dmso	control	-0,4970653	4,5139475	0,00715469	0,03573632
TRINITY_DN5795_c0_g1_i1	dmso	control	-0,4973658	4,81747871	0,00112852	0,00821236
TRINITY_DN1502_c0_g1_i1	dmso	control	-0,4975061	6,1988091	4,02E-05	0,000473
TRINITY_DN32503_c0_g1_i1	dmso	control	-0,4978743	3,97931843	0,00559857	0,02947208
TRINITY_DN4716_c0_g1_i1	dmso	control	-0,4979895	5,84241991	0,00033714	0,0029695

TRINITY_DN11641_c0_g1_i1	dmso	control	-0,4994866	5,15197031	0,00193133	0,01270401
TRINITY_DN838_c0_g1_i1	dmso	control	-0,5004341	6,32503081	1,90E-05	0,00024568
TRINITY_DN19223_c0_g1_i1	dmso	control	-0,5004484	4,64538198	0,00137102	0,00964183
TRINITY_DN15394_c0_g1_i1	dmso	control	-0,5006465	3,09165032	0,02190684	0,08474674
TRINITY_DN20500_c0_g1_i1	dmso	control	-0,5034698	2,81200998	0,02130945	0,08301377
TRINITY_DN10974_c0_g1_i1	dmso	control	-0,5036924	5,23956279	0,00081533	0,00625487
TRINITY_DN13965_c0_g1_i1	dmso	control	-0,5038594	3,22785726	0,02065567	0,08114527
TRINITY_DN40316_c0_g1_i1	dmso	control	-0,5042237	2,80502067	0,02142101	0,08338089
TRINITY_DN42536_c0_g1_i1	dmso	control	-0,5045629	2,77368035	0,0267651	0,09847728
TRINITY_DN18957_c0_g1_i1	dmso	control	-0,5052373	3,62508216	0,01209939	0,05394311
TRINITY_DN13601_c0_g1_i1	dmso	control	-0,5054696	2,63535864	0,0266669	0,0982411
TRINITY_DN37772_c0_g1_i1	dmso	control	-0,5060521	4,84968134	0,00123221	0,0088378
TRINITY_DN8391_c0_g1_i1	dmso	control	-0,5062636	2,74214942	0,02301515	0,08797403
TRINITY_DN294_c0_g1_i1	dmso	control	-0,5063284	8,11447741	8,66E-06	0,00012238
TRINITY_DN4932_c0_g1_i1	dmso	control	-0,5065113	6,32097327	4,22E-05	0,00049273
TRINITY_DN46197_c0_g1_i1	dmso	control	-0,5065968	3,15099221	0,01822431	0,07379992
TRINITY_DN18377_c0_g1_i1	dmso	control	-0,5068795	5,1020856	0,00068544	0,0054075
TRINITY_DN2541_c0_g1_i1	dmso	control	-0,5069956	6,5069304	1,29E-05	0,00017583
TRINITY_DN18729_c0_g1_i1	dmso	control	-0,5077111	6,38606352	5,57E-05	0,00063256
TRINITY_DN13152_c0_g1_i1	dmso	control	-0,5095565	3,01173182	0,01627263	0,0675843
TRINITY_DN8568_c0_g1_i1	dmso	control	-0,5105493	3,24005029	0,01215219	0,05414507
TRINITY_DN11698_c0_g1_i1	dmso	control	-0,5109014	3,53250443	0,01090149	0,04978565
TRINITY_DN12953_c0_g1_i1	dmso	control	-0,5109571	4,43982916	0,00155751	0,01070325
TRINITY_DN11970_c0_g1_i1	dmso	control	-0,5111018	5,69220261	8,53E-05	0,00091079
TRINITY_DN163_c0_g1_i1	dmso	control	-0,5115737	9,00294322	1,68E-05	0,00021944
TRINITY_DN35954_c0_g1_i1	dmso	control	-0,5119734	4,77197431	0,00231708	0,01466703
TRINITY_DN1284_c0_g1_i1	dmso	control	-0,5129144	3,60533277	0,00677826	0,0341633
TRINITY_DN19505_c0_g1_i1	dmso	control	-0,5130632	7,24343598	2,83E-05	0,00034752
TRINITY_DN22717_c0_g1_i1	dmso	control	-0,5132124	3,96699449	0,00738974	0,03666945
TRINITY_DN4961_c0_g1_i1	dmso	control	-0,5137549	8,42837952	0,00015203	0,00149394
TRINITY_DN2429_c0_g1_i1	dmso	control	-0,513836	5,19718623	0,00034778	0,00303727

TRINITY_DN1399_c0_g1_i1	dmso	control	-0,5143451	5,29803736	0,00088361	0,00668053
TRINITY_DN19857_c0_g1_i1	dmso	control	-0,5145427	3,85087163	0,00961603	0,04505467
TRINITY_DN24861_c0_g1_i1	dmso	control	-0,514882	3,02990332	0,02023928	0,07996618
TRINITY_DN6663_c0_g1_i1	dmso	control	-0,5148982	5,6450114	9,85E-05	0,00103096
TRINITY_DN1666_c0_g1_i1	dmso	control	-0,5150987	4,07297153	0,00294801	0,01785919
TRINITY_DN47_c0_g1_i1	dmso	control	-0,5153316	10,217445	1,21E-06	2,09E-05
TRINITY_DN2049_c0_g1_i1	dmso	control	-0,516586	4,85777554	0,00090036	0,00676678
TRINITY_DN6454_c0_g1_i1	dmso	control	-0,5171027	2,59020369	0,02679614	0,09856641
TRINITY_DN21158_c0_g1_i1	dmso	control	-0,5184899	5,3644314	0,00016289	0,00158174
TRINITY_DN1755_c0_g1_i1	dmso	control	-0,5190306	3,70085572	0,00861998	0,04133147
TRINITY_DN8793_c0_g1_i1	dmso	control	-0,5195961	3,11359543	0,01276403	0,05636698
TRINITY_DN13020_c0_g1_i1	dmso	control	-0,5196607	3,32119086	0,00902842	0,04278724
TRINITY_DN11479_c0_g1_i1	dmso	control	-0,5206162	5,18203828	0,00021669	0,00201808
TRINITY_DN7271_c0_g1_i1	dmso	control	-0,5207921	5,70526207	0,00012433	0,00125939
TRINITY_DN10796_c0_g1_i1	dmso	control	-0,5216463	4,16183412	0,0033629	0,01981901
TRINITY_DN6480_c0_g1_i1	dmso	control	-0,5239413	5,27754522	0,00041375	0,00352184
TRINITY_DN22379_c0_g1_i1	dmso	control	-0,5242215	3,953422	0,00992539	0,0461895
TRINITY_DN11336_c0_g1_i1	dmso	control	-0,5244038	3,80518047	0,00796775	0,03893579
TRINITY_DN18828_c0_g1_i1	dmso	control	-0,5250365	7,7708464	0,00091929	0,00688036
TRINITY_DN6605_c0_g1_i1	dmso	control	-0,525147	3,59502979	0,00600536	0,03114809
TRINITY_DN23145_c0_g1_i1	dmso	control	-0,5253568	3,39149603	0,0085355	0,04101638
TRINITY_DN38319_c0_g1_i1	dmso	control	-0,5255289	3,13574272	0,0150428	0,06351747
TRINITY_DN18931_c0_g1_i1	dmso	control	-0,5261984	6,3368712	1,91E-05	0,00024651
TRINITY_DN5528_c0_g1_i1	dmso	control	-0,5274103	5,53839309	9,02E-05	0,00095496
TRINITY_DN2705_c0_g1_i1	dmso	control	-0,5278139	3,86074132	0,00472826	0,0258613
TRINITY_DN726_c0_g1_i1	dmso	control	-0,5280497	6,25451109	1,60E-05	0,0002112
TRINITY_DN14551_c0_g1_i1	dmso	control	-0,5281768	4,05310922	0,00333775	0,01970678
TRINITY_DN148_c0_g1_i1	dmso	control	-0,5284926	4,96673779	0,00048133	0,00400507
TRINITY_DN7193_c0_g1_i1	dmso	control	-0,5285177	3,99765611	0,00374219	0,02153625
TRINITY_DN33275_c0_g1_i1	dmso	control	-0,5286809	3,77856138	0,00380711	0,02179693
TRINITY_DN2091_c0_g1_i1	dmso	control	-0,5292613	4,97850487	0,00026175	0,00238099

TRINITY_DN10629_c0_g1_i1	dmso	control	-0,5304608	3,43582678	0,00663926	0,03364741
TRINITY_DN2080_c0_g1_i1	dmso	control	-0,5306328	4,27980367	0,00319311	0,01908335
TRINITY_DN17851_c0_g1_i1	dmso	control	-0,5309343	5,54042684	0,00326741	0,01943096
TRINITY_DN5880_c0_g1_i1	dmso	control	-0,5313792	3,89376536	0,0032806	0,01947733
TRINITY_DN11506_c0_g1_i1	dmso	control	-0,5318498	3,08761459	0,01082219	0,0494861
TRINITY_DN18869_c0_g1_i1	dmso	control	-0,5327191	6,13969774	2,32E-05	0,00029128
TRINITY_DN23416_c0_g1_i1	dmso	control	-0,5336547	7,06886613	2,70E-05	0,00033325
TRINITY_DN43873_c0_g1_i1	dmso	control	-0,5341018	3,21899926	0,01343158	0,05851002
TRINITY_DN12163_c0_g1_i1	dmso	control	-0,534181	4,99046537	0,00024384	0,00223635
TRINITY_DN32381_c0_g1_i1	dmso	control	-0,5349651	3,57010111	0,00921178	0,04349923
TRINITY_DN7776_c0_g1_i1	dmso	control	-0,5351149	3,34663126	0,00629902	0,03230719
TRINITY_DN28696_c0_g1_i1	dmso	control	-0,5357935	3,87450386	0,00314508	0,01883851
TRINITY_DN12349_c0_g1_i1	dmso	control	-0,5360301	4,73423962	0,00056941	0,00462863
TRINITY_DN19325_c0_g1_i1	dmso	control	-0,5366644	4,03208887	0,00442806	0,02459183
TRINITY_DN12436_c0_g1_i1	dmso	control	-0,5367938	4,38057981	0,0096972	0,0453614
TRINITY_DN12799_c0_g1_i1	dmso	control	-0,5368838	4,08967374	0,00268804	0,01653454
TRINITY_DN20507_c0_g1_i1	dmso	control	-0,5375229	4,15415509	0,00967623	0,04529264
TRINITY_DN33926_c0_g1_i1	dmso	control	-0,5376187	3,70057648	0,00401047	0,02273599
TRINITY_DN25550_c0_g1_i1	dmso	control	-0,538447	2,71727572	0,01896909	0,07603869
TRINITY_DN24099_c0_g1_i1	dmso	control	-0,5387393	2,81286985	0,01558594	0,06520098
TRINITY_DN6549_c0_g1_i1	dmso	control	-0,5387607	5,84634381	0,00122544	0,00880232
TRINITY_DN1412_c0_g1_i1	dmso	control	-0,5401007	3,65659645	0,00587435	0,03060042
TRINITY_DN13429_c0_g1_i1	dmso	control	-0,5407263	3,79123918	0,01144508	0,05169613
TRINITY_DN23217_c0_g1_i1	dmso	control	-0,541457	4,27623671	0,00178758	0,01197107
TRINITY_DN16094_c0_g1_i1	dmso	control	-0,5423552	4,30250723	0,0012585	0,00900398
TRINITY_DN1328_c0_g1_i1	dmso	control	-0,5424589	6,41110605	6,98E-06	0,00010107
TRINITY_DN4559_c0_g1_i1	dmso	control	-0,5432981	6,42525742	6,73E-05	0,00074422
TRINITY_DN20107_c0_g1_i1	dmso	control	-0,5433417	3,69439964	0,00363985	0,02110703
TRINITY_DN10842_c0_g1_i1	dmso	control	-0,5439012	4,60613169	0,00058763	0,0047474
TRINITY_DN5683_c0_g1_i1	dmso	control	-0,5449289	5,25060054	9,85E-05	0,00103096
TRINITY_DN1778_c0_g1_i1	dmso	control	-0,5462447	4,41627527	0,00068258	0,00539226

TRINITY_DN13603_c0_g1_i1	dmso	control	-0,5480946	3,41040896	0,00847076	0,04082754
TRINITY_DN5057_c0_g1_i1	dmso	control	-0,5487519	4,13566715	0,00171588	0,01158757
TRINITY_DN6840_c0_g1_i1	dmso	control	-0,5490058	5,12388316	0,00014648	0,00144826
TRINITY_DN23018_c0_g1_i1	dmso	control	-0,5494105	4,31637668	0,0012932	0,00922024
TRINITY_DN8044_c0_g1_i1	dmso	control	-0,5498468	4,1116804	0,00155246	0,01068382
TRINITY_DN10784_c0_g1_i1	dmso	control	-0,5501512	3,20377383	0,01900503	0,07615192
TRINITY_DN932_c0_g1_i1	dmso	control	-0,5502569	5,01070365	0,00016612	0,00160938
TRINITY_DN20904_c0_g1_i1	dmso	control	-0,551357	3,96250236	0,00217793	0,01400099
TRINITY_DN7941_c0_g1_i1	dmso	control	-0,5519512	4,10309723	0,00175876	0,01181639
TRINITY_DN8381_c0_g1_i1	dmso	control	-0,5523291	2,89216698	0,01171229	0,05254161
TRINITY_DN19628_c0_g1_i1	dmso	control	-0,5523355	4,42540525	0,00110642	0,00806775
TRINITY_DN26022_c0_g1_i1	dmso	control	-0,5523692	2,62479977	0,01862725	0,07501273
TRINITY_DN5945_c0_g1_i1	dmso	control	-0,5543673	4,85186129	0,00069097	0,00543924
TRINITY_DN15096_c0_g1_i1	dmso	control	-0,5544432	3,10352723	0,00845541	0,04078079
TRINITY_DN11153_c0_g1_i1	dmso	control	-0,5551814	3,74929115	0,00298863	0,01803289
TRINITY_DN7131_c0_g1_i1	dmso	control	-0,555376	3,37147355	0,00423021	0,0236751
TRINITY_DN4626_c0_g1_i1	dmso	control	-0,5554102	6,46621612	0,00032127	0,00284887
TRINITY_DN21136_c0_g1_i1	dmso	control	-0,5555947	2,57645061	0,02048821	0,08062309
TRINITY_DN34241_c0_g1_i1	dmso	control	-0,556088	8,53593565	6,74E-05	0,00074439
TRINITY_DN25973_c0_g1_i1	dmso	control	-0,5564783	2,87062006	0,02036897	0,08028099
TRINITY_DN5422_c0_g1_i1	dmso	control	-0,5576281	7,18138658	3,36E-06	5,17E-05
TRINITY_DN15550_c0_g1_i1	dmso	control	-0,5577208	3,53697281	0,00376803	0,02165044
TRINITY_DN5523_c0_g1_i1	dmso	control	-0,5582207	3,91847691	0,00709423	0,03547114
TRINITY_DN12134_c0_g1_i1	dmso	control	-0,558661	3,78213176	0,00229066	0,01455085
TRINITY_DN16367_c0_g1_i1	dmso	control	-0,5588195	5,15955455	0,00016185	0,00157688
TRINITY_DN9968_c0_g1_i1	dmso	control	-0,5589417	5,25268162	0,0030681	0,01845495
TRINITY_DN18736_c0_g1_i1	dmso	control	-0,5591727	4,01338087	0,00354696	0,02069302
TRINITY_DN13646_c0_g1_i1	dmso	control	-0,5592823	4,17188934	0,00833735	0,04031906
TRINITY_DN6326_c0_g1_i1	dmso	control	-0,5594908	2,89043041	0,01097482	0,05000985
TRINITY_DN2320_c0_g1_i1	dmso	control	-0,5601181	4,19872372	0,0010994	0,00803281
TRINITY_DN12091_c0_g1_i1	dmso	control	-0,5604328	3,46125383	0,00715986	0,03573747

TRINITY_DN20579_c0_g1_i1	dmso	control	-0,561301	2,92750501	0,01834589	0,07412765
TRINITY_DN1080_c0_g1_i1	dmso	control	-0,5619528	6,61822169	2,69E-06	4,27E-05
TRINITY_DN11987_c0_g1_i1	dmso	control	-0,5622628	2,75930102	0,02103557	0,08232427
TRINITY_DN9842_c0_g1_i1	dmso	control	-0,5623594	2,47650114	0,01855488	0,07478382
TRINITY_DN6711_c0_g1_i1	dmso	control	-0,5625121	3,04042162	0,0120208	0,05367558
TRINITY_DN33304_c0_g1_i1	dmso	control	-0,5632694	3,71395448	0,00359002	0,02086836
TRINITY_DN1752_c0_g1_i1	dmso	control	-0,5635024	5,33932303	4,64E-05	0,00053615
TRINITY_DN11590_c0_g1_i1	dmso	control	-0,5635684	8,19213129	2,38E-05	0,00029843
TRINITY_DN2155_c0_g1_i1	dmso	control	-0,5646278	6,07341209	1,57E-05	0,00020691
TRINITY_DN20131_c0_g1_i1	dmso	control	-0,5652849	3,51206237	0,00543769	0,02883533
TRINITY_DN8881_c0_g1_i1	dmso	control	-0,5656713	2,40125516	0,02169763	0,08416279
TRINITY_DN27371_c0_g1_i1	dmso	control	-0,5657559	5,03974305	0,00013114	0,00131676
TRINITY_DN22060_c0_g1_i1	dmso	control	-0,569318	5,19907461	0,00033194	0,00292904
TRINITY_DN25507_c0_g1_i1	dmso	control	-0,5695714	4,41500397	0,00791862	0,0387219
TRINITY_DN6990_c0_g1_i1	dmso	control	-0,569653	3,74499569	0,0023929	0,01509407
TRINITY_DN19680_c0_g1_i1	dmso	control	-0,5700063	5,14075595	7,66E-05	0,00083084
TRINITY_DN48566_c0_g1_i1	dmso	control	-0,5700303	3,00967286	0,01470933	0,06238292
TRINITY_DN18852_c0_g1_i1	dmso	control	-0,5702871	5,0191155	0,00022597	0,00209775
TRINITY_DN4708_c0_g1_i1	dmso	control	-0,5705324	5,63100482	0,0001914	0,00181531
TRINITY_DN14236_c0_g1_i1	dmso	control	-0,5722987	3,16733282	0,01757301	0,07180179
TRINITY_DN122_c0_g1_i1	dmso	control	-0,5725432	7,14179912	9,53E-07	1,67E-05
TRINITY_DN14642_c0_g1_i1	dmso	control	-0,5725872	5,30128853	5,79E-05	0,00065474
TRINITY_DN4713_c0_g1_i1	dmso	control	-0,5726217	5,07322213	0,00088548	0,00668973
TRINITY_DN18654_c0_g1_i1	dmso	control	-0,5731011	5,09985505	0,00333584	0,01970678
TRINITY_DN10614_c0_g1_i1	dmso	control	-0,5739898	2,54746292	0,01789155	0,07273948
TRINITY_DN637_c0_g1_i1	dmso	control	-0,5742993	6,96219713	7,82E-07	1,41E-05
TRINITY_DN11482_c0_g1_i1	dmso	control	-0,5743195	2,92347217	0,0106575	0,04885674
TRINITY_DN7130_c0_g1_i1	dmso	control	-0,5744266	2,62104037	0,01252668	0,05559547
TRINITY_DN2270_c0_g1_i1	dmso	control	-0,5748254	3,34289123	0,00329736	0,01953664
TRINITY_DN14675_c0_g1_i1	dmso	control	-0,5749008	2,1278509	0,02659319	0,09804458
TRINITY_DN12092_c0_g1_i1	dmso	control	-0,5749826	4,02940625	0,00463959	0,02549226

TRINITY_DN83_c0_g1_i1	dmso	control	-0,5759902	11,2944398	1,47E-07	3,15E-06
TRINITY_DN35351_c0_g1_i1	dmso	control	-0,5771837	6,00433191	2,03E-05	0,0002594
TRINITY_DN1543_c0_g1_i1	dmso	control	-0,5774391	3,75958786	0,00183731	0,01223036
TRINITY_DN24277_c0_g1_i1	dmso	control	-0,5778177	5,21732935	0,00014073	0,00140098
TRINITY_DN14451_c0_g1_i1	dmso	control	-0,577868	3,55029989	0,00480323	0,02615253
TRINITY_DN5647_c0_g1_i1	dmso	control	-0,5779905	4,89933315	0,00011224	0,00115557
TRINITY_DN11436_c0_g1_i1	dmso	control	-0,5782132	4,31746774	0,00054208	0,00443643
TRINITY_DN7072_c0_g1_i1	dmso	control	-0,5798553	2,6402818	0,01100132	0,05009902
TRINITY_DN20761_c0_g1_i1	dmso	control	-0,5801027	6,17331883	5,68E-06	8,36E-05
TRINITY_DN6945_c0_g1_i1	dmso	control	-0,5803961	3,87569204	0,00118378	0,00853278
TRINITY_DN18584_c0_g1_i1	dmso	control	-0,5817174	4,66767846	0,00029024	0,00260565
TRINITY_DN12978_c0_g1_i1	dmso	control	-0,5821775	4,03690259	0,00095306	0,007086
TRINITY_DN11666_c0_g1_i1	dmso	control	-0,5828093	5,82807759	5,37E-06	7,95E-05
TRINITY_DN5808_c0_g1_i1	dmso	control	-0,5830209	6,47973574	8,58E-06	0,00012147
TRINITY_DN16681_c0_g1_i1	dmso	control	-0,5834253	3,70121987	0,00157583	0,01081248
TRINITY_DN31431_c0_g1_i1	dmso	control	-0,5843118	3,84299995	0,00184749	0,01229246
TRINITY_DN1370_c0_g1_i1	dmso	control	-0,5853358	3,20480592	0,0077645	0,03807138
TRINITY_DN33201_c0_g1_i1	dmso	control	-0,585483	4,28059066	0,00079412	0,00612481
TRINITY_DN13725_c0_g1_i1	dmso	control	-0,5861391	2,65892329	0,01420766	0,06075479
TRINITY_DN26308_c0_g1_i1	dmso	control	-0,5870755	2,49693733	0,01602781	0,06681723
TRINITY_DN12049_c0_g1_i1	dmso	control	-0,5874889	2,95030379	0,00743347	0,03679798
TRINITY_DN19373_c0_g1_i1	dmso	control	-0,5889595	5,10479051	0,00012001	0,00122252
TRINITY_DN15940_c0_g1_i1	dmso	control	-0,5890498	3,40328419	0,01387102	0,05970073
TRINITY_DN18778_c0_g1_i1	dmso	control	-0,5902082	5,68604933	7,15E-06	0,00010336
TRINITY_DN25402_c0_g1_i1	dmso	control	-0,5903521	3,10684787	0,00764959	0,03764851
TRINITY_DN20668_c0_g1_i1	dmso	control	-0,590608	5,91871045	2,17E-05	0,00027486
TRINITY_DN7925_c0_g1_i1	dmso	control	-0,5906285	2,78572559	0,00866675	0,04150901
TRINITY_DN20495_c0_g1_i1	dmso	control	-0,5907471	3,64495503	0,00241063	0,0151728
TRINITY_DN23158_c0_g1_i1	dmso	control	-0,591023	2,63882785	0,02020312	0,07986701
TRINITY_DN78125_c0_g1_i1	dmso	control	-0,5920212	2,5438361	0,0163459	0,06775221
TRINITY_DN19156_c0_g1_i1	dmso	control	-0,5921963	4,11670642	0,00086489	0,00657118

TRINITY_DN20706_c0_g1_i1	dmso	control	-0,5923132	3,72382866	0,00142633	0,00995328
TRINITY_DN606_c0_g1_i1	dmso	control	-0,5925436	7,25532891	9,80E-07	1,71E-05
TRINITY_DN45971_c0_g1_i1	dmso	control	-0,5931398	3,56494717	0,00433291	0,02413764
TRINITY_DN18171_c0_g1_i1	dmso	control	-0,5939227	2,75774735	0,0091682	0,04332175
TRINITY_DN8088_c0_g1_i1	dmso	control	-0,5945852	3,37083392	0,00208632	0,01353025
TRINITY_DN11911_c0_g1_i1	dmso	control	-0,5950893	5,41572654	1,32E-05	0,00017926
TRINITY_DN12961_c0_g1_i1	dmso	control	-0,5954617	2,96832962	0,00744991	0,03684148
TRINITY_DN12507_c0_g1_i1	dmso	control	-0,5956544	8,42190424	0,0004641	0,00389311
TRINITY_DN22562_c0_g1_i1	dmso	control	-0,5957577	2,47722779	0,02194282	0,0847951
TRINITY_DN19281_c0_g1_i1	dmso	control	-0,5957835	3,81677382	0,00192994	0,01270065
TRINITY_DN18667_c0_g1_i1	dmso	control	-0,5964346	8,55915069	0,00017481	0,00168005
TRINITY_DN2121_c0_g1_i1	dmso	control	-0,5975008	4,42439306	0,00093169	0,00695161
TRINITY_DN14821_c0_g1_i1	dmso	control	-0,5976903	3,11010938	0,00392704	0,02236832
TRINITY_DN37519_c0_g1_i1	dmso	control	-0,5983802	5,77081667	2,06E-05	0,00026343
TRINITY_DN568_c0_g1_i1	dmso	control	-0,598551	7,82577569	1,65E-07	3,46E-06
TRINITY_DN20884_c0_g1_i1	dmso	control	-0,5996685	2,85924374	0,01120159	0,0508028
TRINITY_DN12746_c0_g1_i1	dmso	control	-0,6001014	5,25554402	0,00017915	0,00171831
TRINITY_DN11598_c0_g1_i1	dmso	control	-0,6002863	6,16570427	1,18E-05	0,00016214
TRINITY_DN19056_c0_g1_i1	dmso	control	-0,600846	5,05303605	5,00E-05	0,00057382
TRINITY_DN25082_c0_g1_i1	dmso	control	-0,6017603	4,3334742	0,00025408	0,00231849
TRINITY_DN4788_c0_g1_i1	dmso	control	-0,6017683	3,97119204	0,00070326	0,00552098
TRINITY_DN20217_c0_g1_i1	dmso	control	-0,6024083	5,24568291	2,34E-05	0,00029389
TRINITY_DN1213_c0_g1_i1	dmso	control	-0,6025074	3,14249067	0,00289652	0,01757991
TRINITY_DN8624_c0_g1_i1	dmso	control	-0,602659	3,85319333	0,00115196	0,00833255
TRINITY_DN1482_c0_g1_i1	dmso	control	-0,6037233	5,16108001	2,12E-05	0,00026899
TRINITY_DN49997_c0_g1_i1	dmso	control	-0,6039864	3,45670285	0,00660572	0,03350411
TRINITY_DN21555_c0_g1_i1	dmso	control	-0,6048039	2,83992051	0,01275356	0,05635519
TRINITY_DN4991_c0_g1_i1	dmso	control	-0,605489	8,13797576	1,03E-07	2,29E-06
TRINITY_DN33110_c0_g1_i1	dmso	control	-0,6073962	2,11240599	0,01891892	0,07593327
TRINITY_DN17673_c0_g1_i1	dmso	control	-0,6078905	6,54804507	1,38E-07	2,96E-06
TRINITY_DN12546_c0_g1_i1	dmso	control	-0,6080498	3,68080371	0,0022378	0,01429675

TRINITY_DN24544_c0_g1_i1	dmso	control	-0,6081191	2,49180194	0,01107763	0,05034699
TRINITY_DN10707_c0_g1_i1	dmso	control	-0,60893	8,61656773	3,10E-08	7,43E-07
TRINITY_DN20481_c0_g1_i1	dmso	control	-0,6098241	5,86489461	3,18E-05	0,00038435
TRINITY_DN11943_c0_g1_i1	dmso	control	-0,60994	3,84118923	0,00080983	0,00622568
TRINITY_DN648_c0_g1_i1	dmso	control	-0,6100362	6,95517007	3,43E-07	6,67E-06
TRINITY_DN12508_c0_g1_i1	dmso	control	-0,6114699	3,02177361	0,00332255	0,01965368
TRINITY_DN20142_c0_g1_i1	dmso	control	-0,6123867	1,98800753	0,02004234	0,07936174
TRINITY_DN4965_c0_g1_i1	dmso	control	-0,6124683	7,02556756	8,57E-08	1,91E-06
TRINITY_DN877_c0_g1_i1	dmso	control	-0,612919	5,38762729	1,34E-05	0,0001807
TRINITY_DN7456_c0_g1_i1	dmso	control	-0,6129592	4,11366146	0,00037227	0,00321642
TRINITY_DN19262_c0_g1_i1	dmso	control	-0,6142513	3,62229577	0,00140172	0,00981
TRINITY_DN40053_c0_g1_i1	dmso	control	-0,6144594	2,2667202	0,01903693	0,07625453
TRINITY_DN14885_c0_g1_i1	dmso	control	-0,6150317	5,13838132	2,05E-05	0,00026232
TRINITY_DN33468_c0_g1_i1	dmso	control	-0,6155032	3,97303741	0,00045658	0,00383668
TRINITY_DN33157_c0_g1_i1	dmso	control	-0,6155648	4,67720942	0,0006975	0,00548171
TRINITY_DN19805_c0_g1_i1	dmso	control	-0,6178023	2,77675689	0,00792147	0,03872271
TRINITY_DN11395_c0_g1_i1	dmso	control	-0,6181601	5,53360774	4,81E-06	7,20E-05
TRINITY_DN11179_c0_g1_i1	dmso	control	-0,6189181	4,74266594	4,61E-05	0,00053346
TRINITY_DN23318_c0_g1_i1	dmso	control	-0,6192864	4,46087833	0,0001358	0,00136034
TRINITY_DN198_c0_g1_i1	dmso	control	-0,6205591	6,08128304	1,84E-06	3,03E-05
TRINITY_DN35497_c0_g1_i1	dmso	control	-0,6213118	3,00535445	0,0098557	0,04598102
TRINITY_DN33912_c0_g1_i1	dmso	control	-0,6213182	2,72438456	0,02270928	0,08703928
TRINITY_DN47130_c0_g1_i1	dmso	control	-0,6213741	4,59638858	0,00049855	0,0041246
TRINITY_DN6278_c0_g1_i1	dmso	control	-0,6213889	3,87029235	0,00068954	0,00543098
TRINITY_DN11287_c0_g1_i1	dmso	control	-0,6220264	4,17403973	0,00059929	0,00482808
TRINITY_DN11889_c0_g1_i1	dmso	control	-0,6226537	5,46743893	2,76E-06	4,36E-05
TRINITY_DN18835_c0_g1_i1	dmso	control	-0,6228495	6,08717729	1,58E-06	2,67E-05
TRINITY_DN14989_c0_g1_i1	dmso	control	-0,6243166	3,84761597	0,0005386	0,00441751
TRINITY_DN78412_c0_g1_i1	dmso	control	-0,624327	4,28493806	0,00072651	0,00567979
TRINITY_DN744_c0_g1_i1	dmso	control	-0,6243624	5,04957639	6,46E-05	0,00072028
TRINITY_DN25215_c0_g1_i1	dmso	control	-0,6254556	2,22142135	0,02043806	0,08048741

TRINITY_DN32415_c0_g1_i1	dmso	control	-0,6256091	4,06337156	0,00975575	0,04559103
TRINITY_DN2188_c0_g1_i1	dmso	control	-0,6260153	4,52412056	0,00045914	0,00385378
TRINITY_DN30524_c0_g1_i1	dmso	control	-0,6260162	1,77268447	0,02197348	0,08486814
TRINITY_DN12231_c0_g1_i1	dmso	control	-0,6267559	6,40078787	3,52E-07	6,82E-06
TRINITY_DN8017_c0_g1_i1	dmso	control	-0,6293664	5,94308534	4,79E-07	9,01E-06
TRINITY_DN12981_c0_g1_i1	dmso	control	-0,6293743	3,19200681	0,0018746	0,01241566
TRINITY_DN7197_c0_g1_i1	dmso	control	-0,6308443	3,56951474	0,00357243	0,02079959
TRINITY_DN19902_c0_g1_i1	dmso	control	-0,6318327	6,0907801	5,26E-06	7,82E-05
TRINITY_DN33498_c0_g1_i1	dmso	control	-0,6332885	3,01306766	0,0025248	0,01575407
TRINITY_DN13222_c0_g1_i1	dmso	control	-0,6332899	3,25072393	0,00146158	0,01016488
TRINITY_DN23805_c0_g1_i1	dmso	control	-0,6334642	3,63051223	0,00097055	0,00719323
TRINITY_DN1205_c0_g1_i1	dmso	control	-0,6348812	5,70505166	3,87E-06	5,86E-05
TRINITY_DN19972_c0_g1_i1	dmso	control	-0,6348822	4,58670549	6,50E-05	0,00072342
TRINITY_DN6582_c0_g1_i1	dmso	control	-0,6363641	6,13960459	1,33E-07	2,89E-06
TRINITY_DN3161_c0_g1_i1	dmso	control	-0,6364856	2,80872981	0,00349912	0,02048834
TRINITY_DN6706_c0_g1_i1	dmso	control	-0,6365738	5,40245886	3,92E-05	0,00046341
TRINITY_DN12107_c0_g1_i1	dmso	control	-0,6370581	3,0972187	0,00206904	0,01344459
TRINITY_DN4691_c0_g1_i1	dmso	control	-0,6377345	6,68806522	1,07E-06	1,86E-05
TRINITY_DN20860_c0_g1_i1	dmso	control	-0,6379537	2,44513658	0,00816646	0,03969197
TRINITY_DN8170_c0_g1_i1	dmso	control	-0,6382137	2,68327172	0,00458403	0,02528305
TRINITY_DN2112_c0_g1_i1	dmso	control	-0,6383815	2,24724867	0,01004886	0,04659897
TRINITY_DN9221_c0_g1_i1	dmso	control	-0,6384868	3,80526191	0,001599	0,01094152
TRINITY_DN19613_c0_g1_i1	dmso	control	-0,6393729	2,21134688	0,01039078	0,04789806
TRINITY_DN9773_c0_g1_i1	dmso	control	-0,6399469	1,816099	0,02148397	0,08358092
TRINITY_DN1710_c0_g1_i1	dmso	control	-0,6409732	2,99622449	0,00434762	0,02419161
TRINITY_DN39418_c0_g1_i1	dmso	control	-0,64303	2,7779013	0,00443599	0,0246264
TRINITY_DN22193_c0_g1_i1	dmso	control	-0,6440618	3,17981485	0,0015779	0,01081248
TRINITY_DN19511_c0_g1_i1	dmso	control	-0,6442512	5,27763251	3,31E-06	5,11E-05
TRINITY_DN36161_c0_g1_i1	dmso	control	-0,6444118	2,72055047	0,0207208	0,08129058
TRINITY_DN5857_c0_g1_i1	dmso	control	-0,6463132	5,13247842	8,86E-06	0,000125
TRINITY_DN8150_c0_g1_i1	dmso	control	-0,6479032	2,48862806	0,00715959	0,03573747

TRINITY_DN14943_c0_g1_i1	dmso	control	-0,6483286	3,33754693	0,01075159	0,04922559
TRINITY_DN8651_c0_g1_i1	dmso	control	-0,6484111	5,84465507	2,21E-07	4,50E-06
TRINITY_DN4366_c0_g1_i1	dmso	control	-0,6486403	2,04113045	0,01530462	0,06435959
TRINITY_DN25372_c0_g1_i1	dmso	control	-0,6490298	1,79646386	0,01736442	0,07107654
TRINITY_DN2126_c0_g1_i1	dmso	control	-0,6533132	2,01971158	0,01795936	0,07291257
TRINITY_DN34586_c0_g1_i1	dmso	control	-0,6534062	1,99797923	0,01403499	0,06024211
TRINITY_DN7799_c0_g1_i1	dmso	control	-0,6535402	4,12774486	0,00018828	0,00179046
TRINITY_DN32562_c0_g1_i1	dmso	control	-0,6541004	2,31400264	0,00828131	0,04008832
TRINITY_DN11916_c0_g1_i1	dmso	control	-0,6543466	4,81118136	7,09E-05	0,00077955
TRINITY_DN542_c0_g1_i1	dmso	control	-0,6545727	8,16888447	0,0002766	0,00250031
TRINITY_DN28164_c0_g1_i1	dmso	control	-0,6555594	3,54308278	0,00360473	0,02093702
TRINITY_DN32607_c0_g1_i1	dmso	control	-0,6556874	4,50963105	3,50E-05	0,00041941
TRINITY_DN32096_c0_g1_i1	dmso	control	-0,6561867	3,67292495	0,00042329	0,00358831
TRINITY_DN7689_c0_g1_i1	dmso	control	-0,6576835	8,42662146	9,83E-05	0,00103082
TRINITY_DN12505_c0_g1_i1	dmso	control	-0,657868	2,58699483	0,004137	0,02326159
TRINITY_DN10452_c0_g1_i1	dmso	control	-0,6579987	3,81830114	0,00027081	0,00245438
TRINITY_DN2464_c0_g1_i1	dmso	control	-0,6581635	3,17522159	0,00158645	0,01086081
TRINITY_DN24885_c0_g1_i1	dmso	control	-0,6582083	2,61098054	0,00483281	0,02626408
TRINITY_DN67588_c0_g1_i1	dmso	control	-0,6583873	4,27882002	0,00216375	0,01394086
TRINITY_DN59986_c0_g1_i1	dmso	control	-0,6585211	3,10348435	0,00344267	0,02019877
TRINITY_DN1070_c0_g1_i1	dmso	control	-0,6588704	5,74310491	1,51E-06	2,56E-05
TRINITY_DN23847_c0_g1_i1	dmso	control	-0,659905	2,2841931	0,00956176	0,04484403
TRINITY_DN14855_c0_g1_i1	dmso	control	-0,6608787	4,38911239	0,00066609	0,00528953
TRINITY_DN34397_c0_g1_i1	dmso	control	-0,6628456	4,90163547	0,00010572	0,00109632
TRINITY_DN27938_c0_g1_i1	dmso	control	-0,6631901	2,00686639	0,01946235	0,07757549
TRINITY_DN14144_c0_g1_i1	dmso	control	-0,6634448	2,42950045	0,00930978	0,04389026
TRINITY_DN19976_c0_g1_i1	dmso	control	-0,6638643	2,26401637	0,00741876	0,03676296
TRINITY_DN12105_c0_g1_i1	dmso	control	-0,6642382	3,963277	0,0003391	0,00298494
TRINITY_DN14006_c0_g1_i1	dmso	control	-0,6644715	3,53667309	0,00079632	0,00613815
TRINITY_DN4669_c0_g1_i1	dmso	control	-0,6651976	6,04444593	7,53E-08	1,70E-06
TRINITY_DN32504_c0_g1_i1	dmso	control	-0,6676388	3,62872443	0,00069745	0,00548171

TRINITY_DN90_c0_g1_i1	dmso	control	-0,66806	11,4766834	1,04E-07	2,30E-06
TRINITY_DN13389_c0_g1_i1	dmso	control	-0,6684734	1,60200502	0,02451294	0,09204298
TRINITY_DN8375_c0_g1_i1	dmso	control	-0,6686252	5,12718647	0,00011276	0,00116005
TRINITY_DN9528_c0_g1_i1	dmso	control	-0,6686306	3,61320398	0,00240787	0,015162
TRINITY_DN35080_c0_g1_i1	dmso	control	-0,668638	1,62514495	0,01843887	0,07442008
TRINITY_DN21869_c0_g1_i1	dmso	control	-0,6689337	2,88092997	0,00204093	0,01329178
TRINITY_DN42486_c0_g1_i1	dmso	control	-0,6692923	2,15300184	0,01010161	0,04679851
TRINITY_DN21740_c0_g1_i1	dmso	control	-0,6700985	4,74806729	8,56E-05	0,00091368
TRINITY_DN23563_c0_g1_i1	dmso	control	-0,6702571	2,17818912	0,00867894	0,04154911
TRINITY_DN8890_c0_g1_i1	dmso	control	-0,6706949	2,05633635	0,01432084	0,061099
TRINITY_DN3803_c0_g1_i1	dmso	control	-0,6708933	3,85741316	0,00068755	0,00541823
TRINITY_DN20091_c0_g1_i1	dmso	control	-0,6728758	3,25497717	0,00108844	0,00795675
TRINITY_DN2828_c0_g1_i1	dmso	control	-0,6735671	3,68988208	0,00088637	0,00669296
TRINITY_DN32502_c0_g1_i1	dmso	control	-0,6740558	6,80371311	0,0001329	0,00133226
TRINITY_DN5174_c0_g1_i1	dmso	control	-0,674871	8,78898508	9,03E-08	2,01E-06
TRINITY_DN19017_c0_g1_i1	dmso	control	-0,6752395	6,06463427	3,69E-08	8,73E-07
TRINITY_DN11896_c0_g1_i1	dmso	control	-0,6761123	5,68650111	1,93E-07	4,00E-06
TRINITY_DN401_c0_g1_i1	dmso	control	-0,6762132	6,00669214	2,71E-06	4,30E-05
TRINITY_DN12802_c0_g1_i1	dmso	control	-0,6765019	5,47849607	1,12E-06	1,94E-05
TRINITY_DN32489_c0_g1_i1	dmso	control	-0,6765154	3,77189077	0,00025814	0,00234965
TRINITY_DN4083_c0_g1_i1	dmso	control	-0,6771323	4,97676412	4,07E-06	6,15E-05
TRINITY_DN20293_c0_g1_i1	dmso	control	-0,6771403	2,82672079	0,00173117	0,01166359
TRINITY_DN3750_c0_g1_i1	dmso	control	-0,6773308	1,98033993	0,01003498	0,04659445
TRINITY_DN3270_c0_g1_i1	dmso	control	-0,678828	1,80944679	0,0136132	0,05899869
TRINITY_DN40527_c0_g1_i1	dmso	control	-0,6801713	2,3793589	0,01602009	0,06680433
TRINITY_DN4477_c0_g1_i1	dmso	control	-0,6809941	7,71232411	1,05E-07	2,31E-06
TRINITY_DN18798_c0_g1_i1	dmso	control	-0,6826876	5,28115551	8,80E-07	1,56E-05
TRINITY_DN8865_c0_g1_i1	dmso	control	-0,6827489	3,58883904	0,00081381	0,00624959
TRINITY_DN14462_c0_g1_i1	dmso	control	-0,6829517	2,5557528	0,00395549	0,02249826
TRINITY_DN20160_c0_g1_i1	dmso	control	-0,6830114	1,96400676	0,01500163	0,06341776
TRINITY_DN5245_c0_g1_i1	dmso	control	-0,6831365	3,54945844	0,00043886	0,00369853

TRINITY_DN18975_c0_g1_i1	dmso	control	-0,6842578	5,00888378	2,19E-06	3,53E-05
TRINITY_DN1022_c0_g1_i1	dmso	control	-0,6844456	6,46500351	1,66E-08	4,19E-07
TRINITY_DN15143_c0_g1_i1	dmso	control	-0,684751	1,29004759	0,02541517	0,0946923
TRINITY_DN11452_c0_g1_i1	dmso	control	-0,6856226	5,0508629	3,64E-06	5,55E-05
TRINITY_DN15151_c0_g1_i1	dmso	control	-0,6860278	3,36456123	0,00087187	0,00660761
TRINITY_DN660_c0_g1_i1	dmso	control	-0,6861034	5,02287958	5,61E-06	8,27E-05
TRINITY_DN4721_c0_g1_i1	dmso	control	-0,6867809	11,746993	3,11E-09	8,86E-08
TRINITY_DN13013_c0_g1_i1	dmso	control	-0,6873695	4,29887427	0,00023227	0,00214799
TRINITY_DN33355_c0_g1_i1	dmso	control	-0,6879302	4,25024411	4,88E-05	0,00056199
TRINITY_DN24216_c0_g1_i1	dmso	control	-0,6880346	5,07071984	5,36E-06	7,93E-05
TRINITY_DN17054_c0_g1_i1	dmso	control	-0,6886713	2,68117312	0,00217177	0,01397385
TRINITY_DN14489_c0_g1_i1	dmso	control	-0,689926	3,06271378	0,01448376	0,06156534
TRINITY_DN649_c0_g1_i1	dmso	control	-0,6915116	6,7028216	1,46E-09	4,43E-08
TRINITY_DN7349_c0_g1_i1	dmso	control	-0,6928061	4,86529135	8,06E-05	0,00086866
TRINITY_DN14625_c0_g1_i1	dmso	control	-0,6929058	4,16096771	0,00017868	0,00171499
TRINITY_DN19188_c0_g1_i1	dmso	control	-0,6934204	2,9680505	0,00116003	0,00838669
TRINITY_DN12770_c0_g1_i1	dmso	control	-0,6938482	4,52986359	2,20E-05	0,00027712
TRINITY_DN12999_c0_g1_i1	dmso	control	-0,694307	5,3293104	1,07E-06	1,86E-05
TRINITY_DN66147_c0_g1_i1	dmso	control	-0,6948409	2,19154565	0,00853049	0,04101638
TRINITY_DN5008_c0_g1_i1	dmso	control	-0,6962066	5,32665113	8,93E-07	1,58E-05
TRINITY_DN11098_c0_g1_i1	dmso	control	-0,696339	5,79449179	1,63E-06	2,72E-05
TRINITY_DN14194_c0_g1_i1	dmso	control	-0,6963552	4,93286195	2,75E-06	4,36E-05
TRINITY_DN20319_c0_g1_i1	dmso	control	-0,696471	4,58007107	9,18E-06	0,00012899
TRINITY_DN2925_c0_g1_i1	dmso	control	-0,6974637	3,33838876	0,00090711	0,00680689
TRINITY_DN34098_c0_g1_i1	dmso	control	-0,697649	3,76480807	0,00020503	0,00192195
TRINITY_DN34294_c0_g1_i1	dmso	control	-0,6987557	3,84635883	0,0003105	0,00276519
TRINITY_DN11047_c0_g1_i1	dmso	control	-0,6995437	6,44979761	1,38E-08	3,56E-07
TRINITY_DN21966_c0_g1_i1	dmso	control	-0,700214	2,38771862	0,00577045	0,03020055
TRINITY_DN32698_c0_g1_i1	dmso	control	-0,7014956	3,63803446	0,00033393	0,00294482
TRINITY_DN11292_c0_g1_i1	dmso	control	-0,7026018	7,45702665	1,47E-05	0,00019693
TRINITY_DN12449_c0_g1_i1	dmso	control	-0,7030345	3,79192172	0,00086524	0,00657118

TRINITY_DN66338_c0_g1_i1	dmso	control	-0,7033389	4,25301041	0,00023209	0,00214771
TRINITY_DN26519_c0_g1_i1	dmso	control	-0,7033606	1,67019325	0,01831646	0,07405018
TRINITY_DN31378_c0_g1_i1	dmso	control	-0,7043555	4,39530122	0,00030803	0,0027449
TRINITY_DN7251_c0_g1_i1	dmso	control	-0,7047085	5,05589004	3,91E-06	5,92E-05
TRINITY_DN5534_c0_g1_i1	dmso	control	-0,705059	4,94623675	2,85E-06	4,48E-05
TRINITY_DN5895_c0_g1_i1	dmso	control	-0,7052323	6,11172566	3,06E-06	4,76E-05
TRINITY_DN19254_c0_g1_i1	dmso	control	-0,7085053	3,72322795	0,00011049	0,00114004
TRINITY_DN3638_c0_g1_i1	dmso	control	-0,7090751	3,97585753	6,87E-05	0,00075807
TRINITY_DN9235_c0_g1_i1	dmso	control	-0,7092936	4,32129666	5,03E-05	0,00057667
TRINITY_DN28973_c0_g1_i1	dmso	control	-0,7097318	1,12365613	0,02474969	0,09278726
TRINITY_DN12745_c0_g1_i1	dmso	control	-0,7100056	4,09622138	5,15E-05	0,00058839
TRINITY_DN32725_c0_g1_i1	dmso	control	-0,7133386	2,87887144	0,00136996	0,00963908
TRINITY_DN2368_c0_g1_i1	dmso	control	-0,7142592	2,26889411	0,00371661	0,02143172
TRINITY_DN12942_c0_g1_i1	dmso	control	-0,7148072	3,67608834	0,00087531	0,00662924
TRINITY_DN16405_c0_g1_i1	dmso	control	-0,7157358	1,11715954	0,02462638	0,09239691
TRINITY_DN874_c0_g1_i1	dmso	control	-0,7159795	4,14270068	8,65E-05	0,00092193
TRINITY_DN2715_c0_g1_i1	dmso	control	-0,7175626	8,18056029	1,78E-10	6,28E-09
TRINITY_DN3355_c0_g1_i1	dmso	control	-0,718046	3,92890871	0,00654731	0,0333131
TRINITY_DN19654_c0_g1_i1	dmso	control	-0,7180926	3,81714017	0,00014233	0,00141597
TRINITY_DN9628_c0_g1_i1	dmso	control	-0,7186875	1,21080086	0,02120674	0,08276989
TRINITY_DN20611_c0_g1_i1	dmso	control	-0,7196942	4,12152711	0,00010069	0,00105164
TRINITY_DN33136_c0_g1_i1	dmso	control	-0,7215455	3,11856276	0,00051482	0,00424705
TRINITY_DN19275_c0_g1_i1	dmso	control	-0,7261527	5,30212286	1,00E-05	0,00013998
TRINITY_DN11519_c0_g1_i1	dmso	control	-0,7274952	4,14301276	2,57E-05	0,00031975
TRINITY_DN33705_c0_g1_i1	dmso	control	-0,7275605	1,35439148	0,02668986	0,09828652
TRINITY_DN23396_c0_g1_i1	dmso	control	-0,7281792	5,49227681	2,67E-07	5,34E-06
TRINITY_DN12658_c0_g1_i1	dmso	control	-0,7293194	4,13698475	0,00353627	0,02064728
TRINITY_DN25395_c0_g1_i1	dmso	control	-0,7297102	2,02286955	0,01064804	0,04882885
TRINITY_DN50781_c0_g1_i1	dmso	control	-0,7299631	1,7012896	0,02256198	0,08670067
TRINITY_DN19129_c0_g1_i1	dmso	control	-0,7312643	6,05099629	1,53E-08	3,90E-07
TRINITY_DN26912_c0_g1_i1	dmso	control	-0,7318978	2,54436489	0,00267792	0,01650885

TRINITY_DN14793_c0_g1_i1	dmso	control	-0,7325797	3,49239438	0,00020376	0,00191376
TRINITY_DN6811_c0_g1_i1	dmso	control	-0,7342893	2,26568238	0,00638659	0,03266795
TRINITY_DN8423_c0_g1_i1	dmso	control	-0,7346867	2,88205696	0,00172051	0,01160804
TRINITY_DN14172_c0_g1_i1	dmso	control	-0,7357673	0,97255417	0,02249848	0,08650269
TRINITY_DN15719_c0_g1_i1	dmso	control	-0,7359681	2,47189311	0,00349388	0,02046599
TRINITY_DN35480_c0_g1_i1	dmso	control	-0,7361598	1,05724758	0,02514568	0,09390624
TRINITY_DN33841_c0_g1_i1	dmso	control	-0,7375263	3,6021696	0,00021117	0,00197308
TRINITY_DN15865_c0_g1_i1	dmso	control	-0,737712	4,51202281	3,53E-05	0,00042207
TRINITY_DN15871_c0_g1_i1	dmso	control	-0,738776	4,38518594	1,54E-05	0,00020475
TRINITY_DN20029_c0_g1_i1	dmso	control	-0,7389791	2,64418312	0,00299492	0,01806331
TRINITY_DN36448_c0_g1_i1	dmso	control	-0,7393051	2,34952396	0,0035086	0,02053554
TRINITY_DN187_c0_g1_i1	dmso	control	-0,7397367	6,48445306	1,03E-09	3,21E-08
TRINITY_DN23110_c0_g1_i1	dmso	control	-0,7413318	2,41923544	0,00207621	0,01348511
TRINITY_DN15727_c0_g1_i1	dmso	control	-0,7415187	1,80448819	0,01087267	0,04966976
TRINITY_DN19385_c0_g1_i1	dmso	control	-0,7418383	5,11108855	1,43E-05	0,00019221
TRINITY_DN13173_c0_g1_i1	dmso	control	-0,7433377	2,24725672	0,00370281	0,02137006
TRINITY_DN6473_c0_g1_i1	dmso	control	-0,7439745	2,76285259	0,00105733	0,00778048
TRINITY_DN22102_c0_g1_i1	dmso	control	-0,7452694	1,85778799	0,00646369	0,03295728
TRINITY_DN6878_c0_g1_i1	dmso	control	-0,7453374	3,13012221	0,00036129	0,00313425
TRINITY_DN19876_c0_g1_i1	dmso	control	-0,7459414	2,93211074	0,00086051	0,00655493
TRINITY_DN18969_c0_g1_i1	dmso	control	-0,7459872	5,26240019	2,47E-05	0,00030772
TRINITY_DN2982_c0_g1_i1	dmso	control	-0,7463121	3,99761849	6,62E-05	0,00073422
TRINITY_DN18911_c0_g1_i1	dmso	control	-0,7477633	7,485307	3,11E-09	8,86E-08
TRINITY_DN6447_c0_g1_i1	dmso	control	-0,748271	4,23258957	1,92E-05	0,0002482
TRINITY_DN48034_c0_g1_i1	dmso	control	-0,7487823	1,25410409	0,02124653	0,0828804
TRINITY_DN5910_c0_g1_i1	dmso	control	-0,7494635	4,64289291	1,42E-06	2,42E-05
TRINITY_DN18773_c0_g1_i1	dmso	control	-0,749581	5,88461403	1,77E-07	3,71E-06
TRINITY_DN14376_c0_g1_i1	dmso	control	-0,7496631	4,52244733	3,95E-05	0,0004658
TRINITY_DN25333_c0_g1_i1	dmso	control	-0,749836	0,89535329	0,02544086	0,09471473
TRINITY_DN40298_c0_g1_i1	dmso	control	-0,7499014	1,14147959	0,02260944	0,08681377
TRINITY_DN4109_c0_g1_i1	dmso	control	-0,7499134	1,14041879	0,02499038	0,09344707

TRINITY_DN27310_c0_g1_i1	dmso	control	-0,7499354	3,198103	0,00030009	0,00268409
TRINITY_DN14452_c0_g1_i1	dmso	control	-0,7499598	2,34527595	0,00536734	0,02853558
TRINITY_DN35638_c0_g1_i1	dmso	control	-0,7501977	2,74230174	0,00089648	0,00675514
TRINITY_DN19826_c0_g1_i1	dmso	control	-0,7502037	2,60442455	0,00290727	0,01762295
TRINITY_DN44882_c0_g1_i1	dmso	control	-0,7505496	1,7502595	0,00764677	0,03764851
TRINITY_DN39912_c0_g1_i1	dmso	control	-0,7514977	1,2697307	0,0163708	0,06783599
TRINITY_DN14623_c0_g1_i1	dmso	control	-0,7515885	2,88710225	0,00316794	0,01894858
TRINITY_DN12577_c0_g1_i1	dmso	control	-0,7517909	7,6444366	2,46E-08	6,03E-07
TRINITY_DN3024_c0_g1_i1	dmso	control	-0,7531904	2,23562698	0,00695542	0,03487365
TRINITY_DN7989_c0_g1_i1	dmso	control	-0,755567	2,84245736	0,00067467	0,00534301
TRINITY_DN11884_c0_g1_i1	dmso	control	-0,7557928	4,99953158	5,81E-07	1,07E-05
TRINITY_DN13556_c0_g1_i1	dmso	control	-0,7567249	4,43476539	2,84E-06	4,47E-05
TRINITY_DN1407_c0_g1_i1	dmso	control	-0,7572026	3,7950502	6,68E-05	0,00073958
TRINITY_DN29930_c0_g1_i1	dmso	control	-0,7575177	0,9490722	0,02104659	0,08232873
TRINITY_DN8572_c0_g1_i1	dmso	control	-0,759298	3,15039764	0,0035978	0,02090513
TRINITY_DN34049_c0_g1_i1	dmso	control	-0,7597492	0,70005299	0,02547748	0,09482663
TRINITY_DN10130_c0_g1_i1	dmso	control	-0,7606425	2,21244152	0,00289042	0,01755653
TRINITY_DN36495_c0_g1_i1	dmso	control	-0,7608015	1,09711429	0,01542589	0,06477527
TRINITY_DN57454_c0_g1_i1	dmso	control	-0,7610448	3,4129186	0,00039903	0,00340864
TRINITY_DN19021_c0_g1_i1	dmso	control	-0,7611323	5,28566129	5,93E-07	1,09E-05
TRINITY_DN545_c0_g1_i1	dmso	control	-0,7627068	4,3605934	8,54E-06	0,00012109
TRINITY_DN12910_c0_g1_i1	dmso	control	-0,7630929	1,96211024	0,00406799	0,02294499
TRINITY_DN3419_c0_g1_i1	dmso	control	-0,7635748	5,14331698	1,34E-07	2,90E-06
TRINITY_DN13465_c0_g1_i1	dmso	control	-0,7644016	0,90231437	0,02343454	0,08912901
TRINITY_DN21794_c0_g1_i1	dmso	control	-0,7665836	2,38475421	0,00194947	0,0127826
TRINITY_DN22561_c0_g1_i1	dmso	control	-0,768211	2,58789155	0,00376598	0,02164725
TRINITY_DN48093_c0_g1_i1	dmso	control	-0,768482	3,91955973	0,00012314	0,00124917
TRINITY_DN19118_c0_g1_i1	dmso	control	-0,7689597	8,20311435	1,71E-12	8,37E-11
TRINITY_DN12632_c0_g1_i1	dmso	control	-0,7691645	1,34376057	0,01297887	0,05703633
TRINITY_DN35012_c0_g1_i1	dmso	control	-0,7695473	1,87154201	0,00503456	0,02707528
TRINITY_DN8188_c0_g1_i1	dmso	control	-0,7702129	7,13868277	3,77E-10	1,27E-08

TRINITY_DN13619_c0_g1_i1	dmso	control	-0,7708604	3,69739303	3,93E-05	0,00046397
TRINITY_DN24437_c0_g1_i1	dmso	control	-0,77178	0,82242836	0,02105381	0,08232873
TRINITY_DN7951_c0_g1_i1	dmso	control	-0,7718838	1,48773768	0,01298186	0,05703633
TRINITY_DN13025_c0_g1_i1	dmso	control	-0,7721986	2,59660432	0,00108337	0,00792986
TRINITY_DN25966_c0_g1_i1	dmso	control	-0,7746784	1,46449534	0,00822582	0,03987314
TRINITY_DN21752_c0_g1_i1	dmso	control	-0,7754096	2,45401504	0,00220046	0,01410811
TRINITY_DN10049_c0_g1_i1	dmso	control	-0,7756215	2,56993899	0,00787505	0,038548
TRINITY_DN14308_c0_g1_i1	dmso	control	-0,7766622	3,49048862	8,94E-05	0,00094733
TRINITY_DN13321_c0_g1_i1	dmso	control	-0,777456	2,32745921	0,00201512	0,01314741
TRINITY_DN78086_c0_g1_i1	dmso	control	-0,7775582	1,98802302	0,00310397	0,01862885
TRINITY_DN39228_c0_g1_i1	dmso	control	-0,7777318	1,61835275	0,01910235	0,07645703
TRINITY_DN7127_c0_g1_i1	dmso	control	-0,7788939	4,11441365	0,00110462	0,0080587
TRINITY_DN3634_c0_g1_i1	dmso	control	-0,7792182	3,21749453	0,00034403	0,00301369
TRINITY_DN9494_c0_g1_i1	dmso	control	-0,7793698	7,0342302	2,49E-09	7,35E-08
TRINITY_DN11847_c0_g1_i1	dmso	control	-0,7824667	5,2240505	0,00023189	0,00214717
TRINITY_DN12457_c0_g1_i1	dmso	control	-0,7832015	3,14430832	0,00489375	0,02655532
TRINITY_DN2_c0_g1_i1	dmso	control	-0,783218	8,48975166	1,01E-11	4,42E-10
TRINITY_DN19744_c0_g1_i1	dmso	control	-0,7840652	2,02970388	0,00539813	0,02867817
TRINITY_DN41295_c0_g1_i1	dmso	control	-0,7841268	2,47437581	0,00168909	0,0114227
TRINITY_DN17073_c0_g1_i1	dmso	control	-0,7841846	3,6714079	8,66E-05	0,00092216
TRINITY_DN8388_c0_g1_i1	dmso	control	-0,7870902	3,93741282	4,43E-05	0,00051528
TRINITY_DN20344_c0_g1_i1	dmso	control	-0,7906991	1,85834492	0,01199607	0,05361275
TRINITY_DN65788_c0_g1_i1	dmso	control	-0,792192	3,61726949	6,62E-05	0,00073422
TRINITY_DN34345_c0_g1_i1	dmso	control	-0,7923873	1,83170181	0,00375994	0,02162976
TRINITY_DN10921_c0_g1_i1	dmso	control	-0,7934868	5,01616331	3,36E-07	6,56E-06
TRINITY_DN36497_c0_g1_i1	dmso	control	-0,7935226	3,94979913	7,90E-06	0,00011301
TRINITY_DN21596_c0_g1_i1	dmso	control	-0,7951231	1,90678929	0,00397507	0,02258311
TRINITY_DN17755_c0_g1_i1	dmso	control	-0,7951269	3,07329963	0,00018657	0,00177772
TRINITY_DN2315_c0_g1_i1	dmso	control	-0,795932	2,4731878	0,0007646	0,00592524
TRINITY_DN14219_c0_g1_i1	dmso	control	-0,7965565	4,24340749	2,44E-06	3,90E-05
TRINITY_DN39229_c0_g1_i1	dmso	control	-0,7971437	0,97223208	0,0194526	0,07755805

TRINITY_DN2577_c0_g1_i1	dmso	control	-0,7979653	2,15056191	0,00252746	0,01576389
TRINITY_DN3509_c0_g1_i1	dmso	control	-0,7996612	4,893824	3,44E-06	5,28E-05
TRINITY_DN20671_c0_g1_i1	dmso	control	-0,8008032	1,61504813	0,00606758	0,03142566
TRINITY_DN6457_c0_g1_i1	dmso	control	-0,8010668	2,48690014	0,00069495	0,00546764
TRINITY_DN8979_c0_g1_i1	dmso	control	-0,8029487	3,0641809	0,0001325	0,00132913
TRINITY_DN11923_c0_g1_i1	dmso	control	-0,8043405	4,18141337	5,97E-06	8,76E-05
TRINITY_DN23124_c0_g1_i1	dmso	control	-0,8043581	1,36206156	0,00855841	0,04109913
TRINITY_DN35302_c0_g1_i1	dmso	control	-0,8045378	1,13379146	0,0109908	0,05006688
TRINITY_DN23614_c0_g1_i1	dmso	control	-0,8067519	1,8664589	0,01217706	0,05423917
TRINITY_DN13636_c0_g1_i1	dmso	control	-0,8078746	1,82402426	0,00323145	0,01925674
TRINITY_DN1025_c0_g1_i1	dmso	control	-0,8078965	5,66165582	3,80E-09	1,06E-07
TRINITY_DN9298_c0_g1_i1	dmso	control	-0,8079271	3,48330598	3,02E-05	0,00036772
TRINITY_DN4444_c0_g1_i1	dmso	control	-0,8079534	4,93630126	8,41E-08	1,89E-06
TRINITY_DN9314_c0_g1_i1	dmso	control	-0,8092937	1,92918727	0,00342928	0,02012838
TRINITY_DN18390_c0_g1_i1	dmso	control	-0,8113951	2,30999611	0,00142492	0,00995307
TRINITY_DN6646_c0_g1_i1	dmso	control	-0,8122005	4,23779342	2,23E-06	3,59E-05
TRINITY_DN38495_c0_g1_i1	dmso	control	-0,8127814	3,07321296	0,0003315	0,00292696
TRINITY_DN15774_c0_g1_i1	dmso	control	-0,8136449	0,94358196	0,01849881	0,07462031
TRINITY_DN21913_c0_g1_i1	dmso	control	-0,814773	9,76480323	5,26E-11	2,02E-09
TRINITY_DN37082_c0_g1_i1	dmso	control	-0,8155254	2,18116406	0,00629839	0,03230719
TRINITY_DN19306_c0_g1_i1	dmso	control	-0,8155377	4,36538244	8,90E-06	0,0001254
TRINITY_DN38880_c0_g1_i1	dmso	control	-0,8160257	2,33056966	0,001398	0,00978873
TRINITY_DN13876_c0_g1_i1	dmso	control	-0,8188394	3,44694293	0,0004184	0,00355317
TRINITY_DN19571_c0_g1_i1	dmso	control	-0,8206124	3,21096298	0,00015518	0,00151975
TRINITY_DN36465_c0_g1_i1	dmso	control	-0,8208185	2,74289228	0,00034259	0,00300836
TRINITY_DN35722_c0_g1_i1	dmso	control	-0,820824	4,40422553	3,83E-07	7,38E-06
TRINITY_DN34966_c0_g1_i1	dmso	control	-0,8212886	0,54448994	0,02371676	0,09001275
TRINITY_DN1644_c0_g1_i1	dmso	control	-0,8221411	4,35689168	1,06E-06	1,84E-05
TRINITY_DN13884_c0_g1_i1	dmso	control	-0,8227007	2,70981492	0,00024501	0,00224562
TRINITY_DN8602_c0_g1_i1	dmso	control	-0,8227697	4,99508671	2,94E-08	7,09E-07
TRINITY_DN49515_c0_g1_i1	dmso	control	-0,8230539	2,02728975	0,00166839	0,01130922

TRINITY_DN2648_c0_g1_i1	dmso	control	-0,8233786	1,83222775	0,00263231	0,01627896
TRINITY_DN3366_c0_g1_i1	dmso	control	-0,8238637	1,45586497	0,02406816	0,09094024
TRINITY_DN22055_c0_g1_i1	dmso	control	-0,8242133	1,38326162	0,00905759	0,0429114
TRINITY_DN22191_c0_g1_i1	dmso	control	-0,8246308	2,38522748	0,00064906	0,00517705
TRINITY_DN13410_c0_g1_i1	dmso	control	-0,825191	1,71076402	0,00357147	0,02079959
TRINITY_DN1375_c0_g1_i1	dmso	control	-0,8257642	3,85252295	8,21E-06	0,00011712
TRINITY_DN33623_c0_g1_i1	dmso	control	-0,8276732	0,77927116	0,01804657	0,07322547
TRINITY_DN40383_c0_g1_i1	dmso	control	-0,8280748	3,71881064	7,42E-06	0,00010686
TRINITY_DN7930_c0_g1_i1	dmso	control	-0,828588	4,99260316	1,80E-08	4,51E-07
TRINITY_DN28644_c0_g1_i1	dmso	control	-0,8286131	2,25723006	0,01255282	0,05565716
TRINITY_DN13434_c0_g1_i1	dmso	control	-0,8287351	6,78646588	3,06E-12	1,44E-10
TRINITY_DN35778_c0_g1_i1	dmso	control	-0,8292236	1,66895735	0,00572753	0,03004146
TRINITY_DN31957_c0_g1_i1	dmso	control	-0,8300786	5,03063065	3,58E-07	6,93E-06
TRINITY_DN12562_c0_g1_i1	dmso	control	-0,8302781	3,72334236	9,61E-06	0,00013468
TRINITY_DN22596_c0_g1_i1	dmso	control	-0,8315649	3,33159907	7,29E-05	0,00079881
TRINITY_DN37565_c0_g1_i1	dmso	control	-0,8324223	1,55772216	0,00797273	0,03894693
TRINITY_DN27586_c0_g1_i1	dmso	control	-0,832841	2,65715796	0,00078926	0,00609949
TRINITY_DN20314_c0_g1_i1	dmso	control	-0,8332947	2,10406015	0,00142565	0,00995328
TRINITY_DN22668_c0_g1_i1	dmso	control	-0,8341047	2,67697077	0,00038701	0,00331966
TRINITY_DN2671_c0_g1_i1	dmso	control	-0,8355206	1,80818168	0,00202146	0,01318285
TRINITY_DN15390_c0_g1_i1	dmso	control	-0,8364327	3,86108699	8,93E-06	0,00012559
TRINITY_DN38068_c0_g1_i1	dmso	control	-0,8371352	1,18854061	0,00757153	0,03735339
TRINITY_DN20854_c0_g1_i1	dmso	control	-0,8371542	0,59357437	0,02163969	0,0840509
TRINITY_DN57087_c0_g1_i1	dmso	control	-0,8373157	1,65737717	0,01152528	0,05192847
TRINITY_DN6322_c0_g1_i1	dmso	control	-0,8381843	1,44726494	0,00624381	0,03210816
TRINITY_DN15124_c0_g1_i1	dmso	control	-0,8384783	4,89721082	1,46E-06	2,47E-05
TRINITY_DN43491_c0_g1_i1	dmso	control	-0,8386493	2,47338671	0,00591904	0,03077774
TRINITY_DN33723_c0_g1_i1	dmso	control	-0,8390284	0,63010683	0,02202402	0,08499518
TRINITY_DN16235_c0_g1_i1	dmso	control	-0,8404733	4,24098318	1,83E-06	3,03E-05
TRINITY_DN38603_c0_g1_i1	dmso	control	-0,8415302	1,60597875	0,00287876	0,01750154
TRINITY_DN2672_c0_g1_i1	dmso	control	-0,8426755	2,4806538	0,00124936	0,00895189

TRINITY_DN3079_c0_g1_i1	dmso	control	-0,8429764	1,79970601	0,00197476	0,0129308
TRINITY_DN1694_c0_g1_i1	dmso	control	-0,84456	2,66572768	0,00066595	0,00528953
TRINITY_DN14317_c0_g1_i1	dmso	control	-0,84567	2,23067913	0,00582533	0,03043287
TRINITY_DN18919_c0_g1_i1	dmso	control	-0,8473165	5,4057269	2,35E-07	4,76E-06
TRINITY_DN19443_c0_g1_i1	dmso	control	-0,8473644	2,3518265	0,00068693	0,00541633
TRINITY_DN12730_c0_g1_i1	dmso	control	-0,8492181	2,57494984	0,00727601	0,0362171
TRINITY_DN12566_c0_g1_i1	dmso	control	-0,8495493	4,15873018	1,84E-06	3,03E-05
TRINITY_DN11260_c0_g1_i1	dmso	control	-0,8500856	3,89721887	4,05E-06	6,12E-05
TRINITY_DN12617_c0_g1_i1	dmso	control	-0,8508292	5,39921867	1,52E-06	2,57E-05
TRINITY_DN15945_c0_g1_i1	dmso	control	-0,8514814	3,80848037	1,32E-05	0,00017884
TRINITY_DN5476_c0_g1_i1	dmso	control	-0,8518104	5,14831929	4,92E-07	9,21E-06
TRINITY_DN27614_c0_g1_i1	dmso	control	-0,8538083	1,88948441	0,0020557	0,01336992
TRINITY_DN32739_c0_g1_i1	dmso	control	-0,8548083	1,70207705	0,00455272	0,02515954
TRINITY_DN15891_c0_g1_i1	dmso	control	-0,8553168	2,5273527	0,00335188	0,0197624
TRINITY_DN8047_c0_g1_i1	dmso	control	-0,8566149	7,24298252	1,93E-11	8,05E-10
TRINITY_DN4178_c0_g1_i1	dmso	control	-0,8571072	0,32284563	0,0224046	0,08621055
TRINITY_DN21642_c0_g1_i1	dmso	control	-0,8585589	1,6906238	0,00273254	0,01676074
TRINITY_DN7944_c0_g1_i1	dmso	control	-0,8586157	3,84272963	1,26E-05	0,00017228
TRINITY_DN26352_c0_g1_i1	dmso	control	-0,858801	0,5541122	0,02048926	0,08062309
TRINITY_DN25661_c0_g1_i1	dmso	control	-0,8590135	1,75792739	0,00257802	0,01601696
TRINITY_DN2960_c0_g1_i1	dmso	control	-0,8613309	3,25164887	0,00012504	0,00126571
TRINITY_DN17460_c0_g1_i1	dmso	control	-0,8629452	4,47554365	6,91E-07	1,25E-05
TRINITY_DN21131_c0_g1_i1	dmso	control	-0,8634154	1,83228395	0,00498566	0,02690248
TRINITY_DN6000_c0_g1_i1	dmso	control	-0,8640384	3,76580436	5,63E-06	8,30E-05
TRINITY_DN4493_c0_g1_i1	dmso	control	-0,8641834	3,20756906	4,62E-05	0,00053452
TRINITY_DN20046_c0_g1_i1	dmso	control	-0,8658535	0,51736652	0,02622259	0,09691197
TRINITY_DN7939_c0_g1_i1	dmso	control	-0,8664524	1,13177993	0,00610039	0,03153906
TRINITY_DN664_c0_g1_i1	dmso	control	-0,8672143	7,83510847	8,26E-14	4,89E-12
TRINITY_DN44043_c0_g1_i1	dmso	control	-0,8682216	0,67731877	0,02307937	0,08814953
TRINITY_DN8766_c0_g1_i1	dmso	control	-0,8687062	2,52009064	0,00076293	0,00591867
TRINITY_DN4879_c0_g1_i1	dmso	control	-0,8694128	4,41465616	1,30E-07	2,82E-06

TRINITY_DN23015_c0_g1_i1	dmso	control	-0,8711402	1,45477135	0,00519597	0,02775914
TRINITY_DN32733_c0_g1_i1	dmso	control	-0,8740132	3,19768314	8,33E-05	0,0008909
TRINITY_DN10520_c0_g1_i1	dmso	control	-0,8754205	2,58013024	0,00186305	0,01235051
TRINITY_DN32782_c0_g1_i1	dmso	control	-0,8762484	1,13154002	0,01508574	0,06360583
TRINITY_DN3412_c0_g1_i1	dmso	control	-0,8762488	2,68704406	0,00474486	0,02591283
TRINITY_DN15106_c0_g1_i1	dmso	control	-0,8763741	1,14763125	0,00733926	0,03645656
TRINITY_DN14017_c0_g1_i1	dmso	control	-0,8791247	4,78157849	3,01E-08	7,22E-07
TRINITY_DN25298_c0_g1_i1	dmso	control	-0,8806695	0,77118678	0,01822906	0,07379992
TRINITY_DN29726_c0_g1_i1	dmso	control	-0,8808099	0,62520138	0,01689189	0,06953642
TRINITY_DN20900_c0_g1_i1	dmso	control	-0,8808269	0,75515536	0,0111474	0,05058883
TRINITY_DN23138_c0_g1_i1	dmso	control	-0,8809263	3,43826879	7,57E-06	0,00010897
TRINITY_DN18521_c0_g1_i1	dmso	control	-0,8810284	1,04118718	0,00691581	0,03469918
TRINITY_DN10312_c0_g1_i1	dmso	control	-0,8813496	1,76341548	0,00651758	0,03318523
TRINITY_DN27672_c0_g1_i1	dmso	control	-0,8823457	1,69000119	0,0035313	0,02063499
TRINITY_DN24234_c0_g1_i1	dmso	control	-0,8832318	3,24690557	1,03E-05	0,00014356
TRINITY_DN3821_c0_g1_i1	dmso	control	-0,8837932	0,16938149	0,02303058	0,08800972
TRINITY_DN35436_c0_g1_i1	dmso	control	-0,8866771	0,91379824	0,01883379	0,07563362
TRINITY_DN15987_c0_g1_i1	dmso	control	-0,8878792	0,25232204	0,02498162	0,09344707
TRINITY_DN18635_c0_g1_i1	dmso	control	-0,8895768	10,2929787	1,05E-14	7,08E-13
TRINITY_DN2222_c0_g1_i1	dmso	control	-0,890123	5,26234581	6,01E-10	1,96E-08
TRINITY_DN18222_c0_g1_i1	dmso	control	-0,8925985	0,34597468	0,02386145	0,09046616
TRINITY_DN15409_c0_g1_i1	dmso	control	-0,8930417	2,10339938	0,00066724	0,00529576
TRINITY_DN12641_c0_g1_i1	dmso	control	-0,8941588	4,94980847	1,54E-08	3,93E-07
TRINITY_DN16881_c0_g1_i1	dmso	control	-0,8942725	2,36459069	0,0002973	0,00266412
TRINITY_DN7672_c0_g1_i1	dmso	control	-0,8949301	3,78239585	1,94E-05	0,00024972
TRINITY_DN40204_c0_g1_i1	dmso	control	-0,896656	0,47985026	0,02031333	0,08012725
TRINITY_DN42588_c0_g1_i1	dmso	control	-0,8975891	1,76884549	0,00107409	0,00788908
TRINITY_DN14997_c0_g1_i1	dmso	control	-0,8976223	2,437588	0,00058077	0,00470512
TRINITY_DN37016_c0_g1_i1	dmso	control	-0,899193	4,23763728	8,48E-07	1,51E-05
TRINITY_DN8071_c0_g1_i1	dmso	control	-0,8999596	2,02743786	0,00258894	0,01607099
TRINITY_DN23609_c0_g1_i1	dmso	control	-0,9004166	8,67436616	4,56E-09	1,26E-07

TRINITY_DN32792_c0_g1_i1	dmso	control	-0,9014617	2,48075102	0,00047858	0,00398448
TRINITY_DN15593_c0_g1_i1	dmso	control	-0,9030048	2,72017063	7,53E-05	0,00082012
TRINITY_DN62957_c0_g1_i1	dmso	control	-0,9030155	2,03897386	0,00089839	0,00676251
TRINITY_DN33737_c0_g1_i1	dmso	control	-0,9036522	1,71438536	0,00485548	0,02636742
TRINITY_DN9571_c0_g1_i1	dmso	control	-0,904126	1,52311898	0,0050101	0,02697462
TRINITY_DN14486_c0_g1_i1	dmso	control	-0,9055227	4,83125011	2,56E-09	7,54E-08
TRINITY_DN44971_c0_g1_i1	dmso	control	-0,9055988	2,80678919	6,31E-05	0,00070447
TRINITY_DN22848_c0_g1_i1	dmso	control	-0,906368	3,76987727	1,77E-05	0,00023071
TRINITY_DN36475_c0_g1_i1	dmso	control	-0,9076823	5,82166522	4,33E-11	1,69E-09
TRINITY_DN8847_c0_g1_i1	dmso	control	-0,9085	0,77943008	0,01354918	0,0588864
TRINITY_DN5390_c0_g1_i1	dmso	control	-0,9087042	5,46510745	5,16E-06	7,68E-05
TRINITY_DN20793_c0_g1_i1	dmso	control	-0,9092468	6,58137582	6,42E-13	3,45E-11
TRINITY_DN10502_c0_g1_i1	dmso	control	-0,9106523	6,19546968	8,22E-13	4,38E-11
TRINITY_DN12317_c0_g1_i1	dmso	control	-0,9124504	5,04588229	6,95E-08	1,59E-06
TRINITY_DN3215_c0_g1_i1	dmso	control	-0,9125652	4,33626378	0,01387851	0,05970073
TRINITY_DN34948_c0_g1_i1	dmso	control	-0,9138423	0,78704441	0,00859608	0,04125262
TRINITY_DN21166_c0_g1_i1	dmso	control	-0,9141285	5,79610806	9,41E-10	2,97E-08
TRINITY_DN34008_c0_g1_i1	dmso	control	-0,9148596	2,02196954	0,00074616	0,00581352
TRINITY_DN37732_c0_g1_i1	dmso	control	-0,9155855	2,00939539	0,00044351	0,00372907
TRINITY_DN33431_c0_g1_i1	dmso	control	-0,9156137	1,62509705	0,00181781	0,01212293
TRINITY_DN28790_c0_g1_i1	dmso	control	-0,9161123	1,64310346	0,00654039	0,03328961
TRINITY_DN1521_c0_g1_i1	dmso	control	-0,9169423	4,23723012	8,93E-06	0,00012559
TRINITY_DN9332_c0_g1_i1	dmso	control	-0,9169817	3,76451682	7,10E-07	1,28E-05
TRINITY_DN17568_c0_g1_i1	dmso	control	-0,9188364	2,48661525	0,00088728	0,00669635
TRINITY_DN29477_c0_g1_i1	dmso	control	-0,9208263	0,62516913	0,01045768	0,04808967
TRINITY_DN39257_c0_g1_i1	dmso	control	-0,92202	0,49812174	0,01892803	0,07594874
TRINITY_DN29822_c0_g1_i1	dmso	control	-0,9220208	0,5357507	0,01049806	0,04823297
TRINITY_DN33765_c0_g1_i1	dmso	control	-0,9229075	3,14809038	6,43E-06	9,37E-05
TRINITY_DN11165_c0_g1_i1	dmso	control	-0,9231638	4,46403294	6,61E-08	1,51E-06
TRINITY_DN9705_c0_g1_i1	dmso	control	-0,9234909	3,22033191	5,05E-05	0,00057851
TRINITY_DN35667_c0_g1_i1	dmso	control	-0,9264844	-0,0007957	0,02511274	0,09383176

TRINITY_DN11600_c0_g1_i1	dmso	control	-0,9289096	5,92697759	0,00023091	0,0021395
TRINITY_DN12133_c0_g1_i1	dmso	control	-0,9292337	3,79185813	2,49E-06	3,98E-05
TRINITY_DN30037_c0_g1_i1	dmso	control	-0,9301781	0,31714599	0,01861125	0,07499008
TRINITY_DN20837_c0_g1_i1	dmso	control	-0,9306435	3,59049596	1,03E-06	1,80E-05
TRINITY_DN14117_c0_g1_i1	dmso	control	-0,9310725	1,26244609	0,00279219	0,01707586
TRINITY_DN33789_c0_g1_i1	dmso	control	-0,9338081	7,7605221	1,42E-14	9,39E-13
TRINITY_DN22260_c0_g1_i1	dmso	control	-0,9346906	3,51318221	2,25E-06	3,63E-05
TRINITY_DN9702_c0_g1_i1	dmso	control	-0,9356374	3,03327688	9,07E-05	0,00095964
TRINITY_DN44188_c0_g1_i1	dmso	control	-0,9365155	1,89708674	0,00053888	0,00441751
TRINITY_DN22919_c0_g1_i1	dmso	control	-0,9368954	1,57871853	0,00290602	0,01762278
TRINITY_DN4365_c0_g1_i1	dmso	control	-0,9371019	3,03146121	0,00011792	0,00120547
TRINITY_DN20168_c0_g1_i1	dmso	control	-0,9372788	2,23554584	0,00032365	0,00286818
TRINITY_DN30211_c0_g1_i1	dmso	control	-0,9386588	0,31791114	0,0131323	0,05751718
TRINITY_DN13240_c0_g1_i1	dmso	control	-0,9407045	1,38415384	0,00229785	0,01456067
TRINITY_DN17195_c0_g1_i1	dmso	control	-0,9410624	2,24505306	0,00034282	0,00300853
TRINITY_DN15770_c0_g1_i1	dmso	control	-0,9437714	1,09513367	0,00307628	0,01849227
TRINITY_DN15684_c0_g1_i1	dmso	control	-0,9462165	2,62325336	4,94E-05	0,00056854
TRINITY_DN6345_c0_g1_i1	dmso	control	-0,9471662	0,6165385	0,01024739	0,04736884
TRINITY_DN2280_c0_g1_i1	dmso	control	-0,9511106	4,20484966	0,00557347	0,02937203
TRINITY_DN6120_c0_g1_i1	dmso	control	-0,9516035	5,10227768	2,88E-09	8,32E-08
TRINITY_DN24178_c0_g1_i1	dmso	control	-0,9532662	2,6896564	2,78E-05	0,00034203
TRINITY_DN22493_c0_g1_i1	dmso	control	-0,9539245	1,70914076	0,0019504	0,01278288
TRINITY_DN17454_c0_g1_i1	dmso	control	-0,9550941	2,48684864	0,0001711	0,0016499
TRINITY_DN22842_c0_g1_i1	dmso	control	-0,9562923	2,14010258	0,00021574	0,00201184
TRINITY_DN15158_c0_g1_i1	dmso	control	-0,9563765	3,45794623	2,90E-05	0,0003536
TRINITY_DN24335_c0_g1_i1	dmso	control	-0,9573634	2,59578727	0,00015372	0,00150646
TRINITY_DN17990_c0_g1_i1	dmso	control	-0,9583914	2,23141385	0,00024224	0,00222451
TRINITY_DN22613_c0_g1_i1	dmso	control	-0,9590933	0,77342065	0,01133657	0,05130226
TRINITY_DN37935_c0_g1_i1	dmso	control	-0,9595218	-0,0155229	0,02434415	0,09157584
TRINITY_DN37166_c0_g1_i1	dmso	control	-0,9597981	0,78165819	0,00590962	0,03076198
TRINITY_DN12364_c0_g1_i1	dmso	control	-0,9603703	3,83459315	1,29E-07	2,81E-06

TRINITY_DN34422_c0_g1_i1	dmso	control	-0,9623095	1,56064203	0,02489438	0,09321155
TRINITY_DN24198_c0_g1_i1	dmso	control	-0,9625411	0,39319917	0,01717954	0,07047953
TRINITY_DN2934_c0_g1_i1	dmso	control	-0,963299	3,80756686	1,62E-07	3,41E-06
TRINITY_DN33441_c0_g1_i1	dmso	control	-0,9641239	4,64314138	1,47E-08	3,76E-07
TRINITY_DN14193_c0_g1_i1	dmso	control	-0,9642792	4,32697568	2,15E-06	3,47E-05
TRINITY_DN45310_c0_g1_i1	dmso	control	-0,9646831	3,47019007	6,91E-07	1,25E-05
TRINITY_DN23578_c0_g1_i1	dmso	control	-0,9650565	1,25172181	0,00192072	0,01266574
TRINITY_DN11363_c0_g1_i1	dmso	control	-0,9653275	6,29757344	1,34E-09	4,09E-08
TRINITY_DN32260_c0_g1_i1	dmso	control	-0,9680789	5,18382016	3,51E-11	1,39E-09
TRINITY_DN6086_c0_g1_i1	dmso	control	-0,9689962	4,25514794	1,61E-06	2,70E-05
TRINITY_DN29116_c0_g1_i1	dmso	control	-0,9690494	4,27318146	7,81E-09	2,09E-07
TRINITY_DN37127_c0_g1_i1	dmso	control	-0,9691815	1,79550853	0,00251214	0,01570224
TRINITY_DN55145_c0_g1_i1	dmso	control	-0,970625	-0,010516	0,01548977	0,0649386
TRINITY_DN27845_c0_g1_i1	dmso	control	-0,9710512	4,89279691	5,92E-10	1,94E-08
TRINITY_DN24316_c0_g1_i1	dmso	control	-0,9712647	2,06335192	0,00064626	0,00515756
TRINITY_DN49195_c0_g1_i1	dmso	control	-0,971945	0,4683545	0,00769601	0,03782539
TRINITY_DN23284_c0_g1_i1	dmso	control	-0,9740551	1,09787244	0,00262538	0,01624835
TRINITY_DN21945_c0_g1_i1	dmso	control	-0,9750416	0,75683037	0,00733104	0,03642821
TRINITY_DN3367_c0_g1_i1	dmso	control	-0,9771757	3,65873998	1,84E-06	3,03E-05
TRINITY_DN14023_c0_g1_i1	dmso	control	-0,9782978	2,64745179	3,97E-05	0,00046751
TRINITY_DN5541_c0_g1_i1	dmso	control	-0,9798715	3,95579241	2,86E-07	5,67E-06
TRINITY_DN36356_c0_g1_i1	dmso	control	-0,9801478	1,66390577	0,00133333	0,00942728
TRINITY_DN50735_c0_g1_i1	dmso	control	-0,980625	1,02692246	0,0065985	0,03350277
TRINITY_DN36888_c0_g1_i1	dmso	control	-0,9806929	0,25896452	0,01031643	0,04759528
TRINITY_DN11973_c0_g1_i1	dmso	control	-0,981486	3,3611247	2,74E-05	0,00033801
TRINITY_DN34074_c0_g1_i1	dmso	control	-0,9818187	3,35927666	1,67E-05	0,00021924
TRINITY_DN5408_c0_g1_i1	dmso	control	-0,9818897	5,47266204	8,67E-12	3,79E-10
TRINITY_DN612_c0_g1_i1	dmso	control	-0,9819838	3,95748096	0,00955959	0,04484403
TRINITY_DN3446_c0_g1_i1	dmso	control	-0,9821326	4,38980906	3,54E-09	9,95E-08
TRINITY_DN4399_c0_g1_i1	dmso	control	-0,9845935	0,62512546	0,00754285	0,03723732
TRINITY_DN24454_c0_g1_i1	dmso	control	-0,9875298	1,47569749	0,00090891	0,00681685

TRINITY_DN23374_c0_g1_i1	dmso	control	-0,9885678	0,50535801	0,0061471	0,03171244
TRINITY_DN36484_c0_g1_i1	dmso	control	-0,990085	1,3917176	0,00411863	0,0231763
TRINITY_DN24378_c0_g1_i1	dmso	control	-0,9959238	2,31360759	0,0001719	0,00165543
TRINITY_DN15652_c0_g1_i1	dmso	control	-0,9962568	3,84217032	1,92E-07	4,00E-06
TRINITY_DN26378_c0_g1_i1	dmso	control	-1,0047836	0,81207338	0,00804281	0,03918327
TRINITY_DN11151_c0_g1_i1	dmso	control	-1,0051837	6,49787012	5,03E-17	4,30E-15
TRINITY_DN21303_c0_g1_i1	dmso	control	-1,0070044	3,32855826	8,53E-07	1,52E-05
TRINITY_DN34518_c0_g1_i1	dmso	control	-1,0085608	5,96425041	4,12E-14	2,53E-12
TRINITY_DN8099_c0_g1_i1	dmso	control	-1,0100188	3,526958	1,46E-06	2,47E-05
TRINITY_DN12410_c0_g1_i1	dmso	control	-1,0102959	3,96306642	7,89E-07	1,42E-05
TRINITY_DN5013_c0_g1_i1	dmso	control	-1,0132093	4,13020507	1,69E-08	4,25E-07
TRINITY_DN40700_c0_g1_i1	dmso	control	-1,0146236	-0,1322421	0,01437428	0,06126776
TRINITY_DN2246_c0_g1_i1	dmso	control	-1,0157067	4,49193345	1,63E-10	5,83E-09
TRINITY_DN16142_c0_g1_i1	dmso	control	-1,0159426	1,66615089	0,00049983	0,00413282
TRINITY_DN21472_c0_g1_i1	dmso	control	-1,0187332	2,40668928	0,00084856	0,00647172
TRINITY_DN63104_c0_g1_i1	dmso	control	-1,0195115	2,66872387	1,78E-05	0,00023094
TRINITY_DN19366_c0_g1_i1	dmso	control	-1,0205581	3,93993618	2,48E-08	6,06E-07
TRINITY_DN2379_c0_g1_i1	dmso	control	-1,0213029	5,74844612	3,14E-14	1,97E-12
TRINITY_DN24525_c0_g1_i1	dmso	control	-1,0213523	1,63995425	0,00028572	0,00256986
TRINITY_DN55878_c0_g1_i1	dmso	control	-1,0233155	2,39580589	6,52E-05	0,00072486
TRINITY_DN33984_c0_g1_i1	dmso	control	-1,0243454	0,26125283	0,01063543	0,04878652
TRINITY_DN39253_c0_g1_i1	dmso	control	-1,0252889	0,36982739	0,00887849	0,04225694
TRINITY_DN1113_c0_g1_i1	dmso	control	-1,0259972	3,77228409	2,64E-08	6,41E-07
TRINITY_DN31982_c0_g1_i1	dmso	control	-1,0272198	0,07865731	0,02712698	0,0995981
TRINITY_DN13375_c0_g1_i1	dmso	control	-1,0279032	4,09112559	2,42E-07	4,87E-06
TRINITY_DN67133_c0_g1_i1	dmso	control	-1,0298852	0,15208566	0,01416866	0,06064185
TRINITY_DN34668_c0_g1_i1	dmso	control	-1,030549	0,43440108	0,00767554	0,0377505
TRINITY_DN24941_c0_g1_i1	dmso	control	-1,0326411	1,10222259	0,00180087	0,01203775
TRINITY_DN25567_c0_g1_i1	dmso	control	-1,0362832	1,16959255	0,00309772	0,01861335
TRINITY_DN27452_c0_g1_i1	dmso	control	-1,0373533	1,10586858	0,00121072	0,00870525
TRINITY_DN26239_c0_g1_i1	dmso	control	-1,0374142	1,16728736	0,00246253	0,01543224

TRINITY_DN24795_c0_g1_i1	dmso	control	-1,0411832	2,7510019	1,48E-05	0,00019769
TRINITY_DN22464_c0_g1_i1	dmso	control	-1,0414487	1,15640313	0,00099327	0,0073465
TRINITY_DN24418_c0_g1_i1	dmso	control	-1,0416544	-0,0035556	0,02197344	0,08486814
TRINITY_DN13194_c0_g1_i1	dmso	control	-1,0426486	1,26238774	0,00185624	0,0123278
TRINITY_DN13091_c0_g1_i1	dmso	control	-1,0438962	2,45515098	1,49E-05	0,00019853
TRINITY_DN15595_c0_g1_i1	dmso	control	-1,0439666	2,18554327	6,11E-05	0,00068545
TRINITY_DN59569_c0_g1_i1	dmso	control	-1,0445656	0,89947176	0,00184929	0,0122988
TRINITY_DN14387_c0_g1_i1	dmso	control	-1,0446678	1,93922108	7,76E-05	0,00084058
TRINITY_DN6561_c0_g1_i1	dmso	control	-1,0451755	3,43792182	8,18E-05	0,0008792
TRINITY_DN2674_c0_g1_i1	dmso	control	-1,0452201	4,50140925	3,45E-08	8,19E-07
TRINITY_DN13880_c0_g1_i1	dmso	control	-1,0487405	2,63962307	1,28E-05	0,00017455
TRINITY_DN25069_c0_g1_i1	dmso	control	-1,0487645	0,63563748	0,00378204	0,02171368
TRINITY_DN2419_c0_g1_i1	dmso	control	-1,0498659	4,57273903	2,25E-11	9,27E-10
TRINITY_DN37611_c0_g1_i1	dmso	control	-1,0502622	0,87866603	0,00254304	0,01584652
TRINITY_DN30443_c0_g1_i1	dmso	control	-1,0506439	1,47929935	0,00177549	0,01190116
TRINITY_DN12522_c0_g1_i1	dmso	control	-1,0508502	2,78660429	5,13E-06	7,65E-05
TRINITY_DN12227_c0_g1_i1	dmso	control	-1,051528	3,99006594	2,17E-05	0,00027455
TRINITY_DN20547_c0_g1_i1	dmso	control	-1,0529086	0,13095554	0,01318592	0,05766594
TRINITY_DN14346_c0_g1_i1	dmso	control	-1,0532543	1,1496968	0,00142905	0,00996745
TRINITY_DN19350_c0_g1_i1	dmso	control	-1,0548401	4,30104082	1,78E-08	4,48E-07
TRINITY_DN11200_c0_g1_i1	dmso	control	-1,0550491	1,45970968	0,00337484	0,01985728
TRINITY_DN13423_c0_g1_i1	dmso	control	-1,0554801	4,67824643	4,32E-11	1,69E-09
TRINITY_DN22929_c0_g1_i1	dmso	control	-1,0555282	1,63041371	0,00087078	0,00660282
TRINITY_DN32751_c0_g1_i1	dmso	control	-1,0574992	1,36688868	0,00052585	0,0043306
TRINITY_DN2367_c0_g1_i1	dmso	control	-1,0628127	3,36749368	6,17E-08	1,42E-06
TRINITY_DN23877_c0_g1_i1	dmso	control	-1,0629396	1,69373666	0,00040325	0,0034406
TRINITY_DN71500_c0_g1_i1	dmso	control	-1,0650919	0,10999101	0,01918322	0,0766321
TRINITY_DN18431_c0_g1_i1	dmso	control	-1,0657153	-0,0632107	0,00853445	0,04101638
TRINITY_DN20244_c0_g1_i1	dmso	control	-1,0658645	2,76211048	2,30E-05	0,00028885
TRINITY_DN5969_c0_g1_i1	dmso	control	-1,0677968	4,51386359	1,75E-11	7,39E-10
TRINITY_DN20021_c0_g1_i1	dmso	control	-1,0692337	2,53519387	1,52E-05	0,00020268

TRINITY_DN36861_c0_g1_i1	dmso	control	-1,0699961	1,67947392	0,00056184	0,00457224
TRINITY_DN32437_c0_g1_i1	dmso	control	-1,0712821	4,89672014	1,47E-12	7,36E-11
TRINITY_DN23070_c0_g1_i1	dmso	control	-1,0745322	1,22151873	0,00190572	0,01258714
TRINITY_DN34003_c0_g1_i1	dmso	control	-1,0746543	1,27462714	0,00414708	0,02329107
TRINITY_DN49114_c0_g1_i1	dmso	control	-1,077199	0,487715	0,00353007	0,02063499
TRINITY_DN17518_c0_g1_i1	dmso	control	-1,0778508	3,48169836	5,10E-08	1,19E-06
TRINITY_DN7902_c0_g1_i1	dmso	control	-1,0804819	6,88737838	1,55E-18	1,53E-16
TRINITY_DN8323_c0_g1_i1	dmso	control	-1,0808741	5,1297958	1,11E-10	4,11E-09
TRINITY_DN25385_c0_g1_i1	dmso	control	-1,0812005	3,44097409	1,12E-07	2,45E-06
TRINITY_DN39092_c0_g1_i1	dmso	control	-1,0816292	5,44022493	8,17E-15	5,57E-13
TRINITY_DN20444_c0_g1_i1	dmso	control	-1,0825302	0,4573587	0,00424102	0,02370804
TRINITY_DN7641_c0_g1_i1	dmso	control	-1,0856437	1,88480674	0,00028393	0,00255537
TRINITY_DN27363_c0_g1_i1	dmso	control	-1,0906194	1,37952699	0,00055876	0,00454978
TRINITY_DN6362_c0_g1_i1	dmso	control	-1,0930603	5,13194953	2,58E-08	6,28E-07
TRINITY_DN10665_c0_g1_i1	dmso	control	-1,0938987	0,98141445	0,00191847	0,01265983
TRINITY_DN66918_c0_g1_i1	dmso	control	-1,0976869	2,61203471	2,40E-05	0,00029947
TRINITY_DN11732_c0_g1_i1	dmso	control	-1,0989716	2,32216278	7,35E-06	0,00010589
TRINITY_DN25261_c0_g1_i1	dmso	control	-1,1035662	0,27407117	0,00929939	0,04386993
TRINITY_DN36602_c0_g1_i1	dmso	control	-1,1062609	1,22446613	0,00162445	0,01106329
TRINITY_DN23708_c0_g1_i1	dmso	control	-1,1086365	2,47493711	5,94E-06	8,73E-05
TRINITY_DN42298_c0_g1_i1	dmso	control	-1,1092567	0,58510661	0,0019248	0,01267739
TRINITY_DN6472_c0_g1_i1	dmso	control	-1,1130483	3,85663994	1,12E-06	1,94E-05
TRINITY_DN18380_c0_g1_i1	dmso	control	-1,1133396	2,33503633	2,42E-05	0,0003025
TRINITY_DN8800_c0_g1_i1	dmso	control	-1,118451	5,57969098	1,58E-16	1,28E-14
TRINITY_DN34358_c0_g1_i1	dmso	control	-1,119048	2,10010678	0,00216969	0,01396695
TRINITY_DN7794_c0_g1_i1	dmso	control	-1,121024	2,45658072	3,95E-05	0,00046566
TRINITY_DN23868_c0_g1_i1	dmso	control	-1,1214443	3,1153617	2,97E-06	4,64E-05
TRINITY_DN14622_c0_g1_i1	dmso	control	-1,1254819	1,32272945	0,00214826	0,01387195
TRINITY_DN44762_c0_g1_i1	dmso	control	-1,1255328	1,423768	0,00020168	0,00189793
TRINITY_DN34662_c0_g1_i1	dmso	control	-1,1293876	1,16477864	0,00035768	0,00311222
TRINITY_DN63105_c0_g1_i1	dmso	control	-1,129647	-0,1444072	0,00799499	0,03903834

TRINITY_DN17904_c0_g1_i1	dmso	control	-1,1322186	5,98323778	1,88E-17	1,68E-15
TRINITY_DN34228_c0_g1_i1	dmso	control	-1,1355309	1,52158165	0,00207836	0,01348693
TRINITY_DN47169_c0_g1_i1	dmso	control	-1,1377741	0,94697977	0,00190259	0,01257799
TRINITY_DN14668_c0_g1_i1	dmso	control	-1,1384535	2,29601881	6,59E-06	9,55E-05
TRINITY_DN40157_c0_g1_i1	dmso	control	-1,1406318	2,91300088	3,73E-07	7,18E-06
TRINITY_DN5844_c0_g1_i1	dmso	control	-1,1410038	5,24596564	7,90E-14	4,70E-12
TRINITY_DN17184_c0_g1_i1	dmso	control	-1,1410806	1,72913544	0,00596426	0,030946
TRINITY_DN34181_c0_g1_i1	dmso	control	-1,1436812	1,55868697	9,39E-05	0,00099181
TRINITY_DN21855_c0_g1_i1	dmso	control	-1,1437164	2,07111134	1,54E-05	0,00020484
TRINITY_DN38891_c0_g1_i1	dmso	control	-1,1456419	1,91117305	0,00013715	0,00137296
TRINITY_DN38075_c0_g1_i1	dmso	control	-1,1509918	3,74128385	1,86E-08	4,66E-07
TRINITY_DN8499_c0_g1_i1	dmso	control	-1,1539206	0,90886625	0,00268612	0,01653454
TRINITY_DN14449_c0_g1_i1	dmso	control	-1,1564322	4,48383561	4,11E-13	2,25E-11
TRINITY_DN3725_c0_g1_i1	dmso	control	-1,1589099	2,15759193	0,00026266	0,00238623
TRINITY_DN5213_c0_g1_i1	dmso	control	-1,1598993	4,30900141	2,11E-09	6,30E-08
TRINITY_DN9362_c0_g1_i1	dmso	control	-1,1614901	2,43903018	2,02E-05	0,00025854
TRINITY_DN27861_c0_g1_i1	dmso	control	-1,16163	1,14147327	0,00219581	0,01409086
TRINITY_DN24365_c0_g1_i1	dmso	control	-1,1640821	2,49862434	6,58E-06	9,55E-05
TRINITY_DN40253_c0_g1_i1	dmso	control	-1,1657389	2,72907731	3,54E-05	0,00042276
TRINITY_DN35931_c0_g1_i1	dmso	control	-1,1658353	2,25654634	8,91E-05	0,00094564
TRINITY_DN21097_c0_g1_i1	dmso	control	-1,1696523	1,0430773	0,00131665	0,00934598
TRINITY_DN15217_c0_g1_i1	dmso	control	-1,1700195	2,96549027	1,54E-07	3,28E-06
TRINITY_DN10129_c0_g1_i1	dmso	control	-1,1703434	1,07254775	0,00038201	0,0032867
TRINITY_DN30879_c0_g1_i1	dmso	control	-1,1723583	1,64743769	4,05E-05	0,00047506
TRINITY_DN20068_c0_g1_i1	dmso	control	-1,1723816	3,49578767	2,71E-09	7,91E-08
TRINITY_DN19378_c0_g1_i1	dmso	control	-1,1726292	6,25534446	8,56E-14	5,05E-12
TRINITY_DN1703_c0_g1_i1	dmso	control	-1,172696	8,5741493	2,20E-26	4,41E-24
TRINITY_DN22419_c0_g1_i1	dmso	control	-1,1741311	2,56190039	4,41E-05	0,00051366
TRINITY_DN632_c0_g1_i1	dmso	control	-1,1776586	5,23301667	3,39E-12	1,58E-10
TRINITY_DN9220_c0_g1_i1	dmso	control	-1,1796808	0,64064748	0,00268186	0,01652
TRINITY_DN78653_c0_g1_i1	dmso	control	-1,1800931	0,04973895	0,00425758	0,02378218

TRINITY_DN20204_c0_g1_i1	dmso	control	-1,1811813	3,08628327	2,23E-08	5,50E-07
TRINITY_DN44194_c0_g1_i1	dmso	control	-1,185106	2,19873028	1,51E-05	0,00020069
TRINITY_DN12933_c0_g1_i1	dmso	control	-1,1865603	4,49080229	4,87E-07	9,15E-06
TRINITY_DN52438_c0_g1_i1	dmso	control	-1,1869726	0,38448472	0,00160381	0,01096404
TRINITY_DN38327_c0_g1_i1	dmso	control	-1,1872274	0,50556641	0,00136663	0,00962037
TRINITY_DN14875_c0_g1_i1	dmso	control	-1,1889041	2,61834444	5,31E-07	9,89E-06
TRINITY_DN36681_c0_g1_i1	dmso	control	-1,189895	3,94686205	2,81E-09	8,12E-08
TRINITY_DN44208_c0_g1_i1	dmso	control	-1,1899813	1,05768845	0,00164799	0,0111878
TRINITY_DN26502_c0_g1_i1	dmso	control	-1,1908015	0,60867126	0,00108366	0,00792986
TRINITY_DN32394_c0_g1_i1	dmso	control	-1,1941607	2,85190991	0,00043384	0,00366478
TRINITY_DN20116_c0_g1_i1	dmso	control	-1,2009943	2,08482499	2,78E-05	0,00034181
TRINITY_DN21669_c0_g1_i1	dmso	control	-1,2065253	2,65993728	1,57E-07	3,31E-06
TRINITY_DN10053_c0_g1_i1	dmso	control	-1,2075176	0,18144855	0,00249672	0,01562611
TRINITY_DN40827_c0_g1_i1	dmso	control	-1,2088982	1,66413788	0,00557616	0,02937548
TRINITY_DN21117_c0_g1_i1	dmso	control	-1,2096748	2,53124364	1,64E-06	2,75E-05
TRINITY_DN15609_c0_g1_i1	dmso	control	-1,2118196	2,91592062	2,09E-05	0,00026631
TRINITY_DN23232_c0_g1_i1	dmso	control	-1,2152779	1,53375424	0,00015074	0,00148432
TRINITY_DN11929_c0_g1_i1	dmso	control	-1,2172484	4,4941863	1,54E-12	7,66E-11
TRINITY_DN6305_c0_g1_i1	dmso	control	-1,2237134	2,37816248	9,77E-05	0,00102683
TRINITY_DN34555_c0_g1_i1	dmso	control	-1,2256007	2,98531899	1,28E-08	3,32E-07
TRINITY_DN20332_c0_g1_i1	dmso	control	-1,2268685	3,32162898	9,18E-07	1,62E-05
TRINITY_DN13764_c0_g1_i1	dmso	control	-1,2315851	3,53134792	8,82E-10	2,79E-08
TRINITY_DN14186_c0_g1_i1	dmso	control	-1,2334088	1,05258753	0,00053495	0,00439552
TRINITY_DN51228_c0_g1_i1	dmso	control	-1,2337011	0,47978911	0,00092377	0,0069089
TRINITY_DN1633_c0_g1_i1	dmso	control	-1,2368213	3,68862248	2,40E-08	5,91E-07
TRINITY_DN18895_c0_g1_i1	dmso	control	-1,2381545	2,67697933	5,63E-07	1,04E-05
TRINITY_DN29450_c0_g1_i1	dmso	control	-1,2400856	-0,3411239	0,0057164	0,02999395
TRINITY_DN2009_c0_g1_i1	dmso	control	-1,2433458	3,4559613	1,15E-08	3,02E-07
TRINITY_DN11530_c0_g1_i1	dmso	control	-1,2434727	4,64578042	5,82E-08	1,35E-06
TRINITY_DN24467_c0_g1_i1	dmso	control	-1,2447073	0,90183338	0,00016208	0,00157764
TRINITY_DN64354_c0_g1_i1	dmso	control	-1,2450065	2,60621686	1,43E-07	3,06E-06

TRINITY_DN26375_c0_g1_i1	dmso	control	-1,2450301	0,89728029	0,00068276	0,00539226
TRINITY_DN26018_c0_g1_i1	dmso	control	-1,2459618	1,94612146	0,00068124	0,00538618
TRINITY_DN13407_c0_g1_i1	dmso	control	-1,2523096	3,06908571	1,23E-06	2,11E-05
TRINITY_DN24637_c0_g1_i1	dmso	control	-1,2565733	0,41493663	0,0029511	0,01785919
TRINITY_DN42338_c0_g1_i1	dmso	control	-1,2567841	0,889608	0,00018504	0,00176661
TRINITY_DN920_c0_g1_i1	dmso	control	-1,2609613	4,86138767	1,70E-15	1,21E-13
TRINITY_DN49379_c0_g1_i1	dmso	control	-1,263401	-0,1009832	0,00265195	0,01637771
TRINITY_DN20693_c0_g1_i1	dmso	control	-1,2634479	4,74239377	2,47E-11	1,01E-09
TRINITY_DN3962_c0_g1_i1	dmso	control	-1,2639695	0,867899	0,00030038	0,00268501
TRINITY_DN11553_c0_g1_i1	dmso	control	-1,2647849	2,54789888	3,17E-07	6,21E-06
TRINITY_DN24356_c0_g1_i1	dmso	control	-1,2650525	1,85626355	7,66E-06	0,00011016
TRINITY_DN15384_c0_g1_i1	dmso	control	-1,2693985	0,50610273	0,00076347	0,00591969
TRINITY_DN18306_c0_g1_i1	dmso	control	-1,2700985	1,041679	0,00015062	0,00148407
TRINITY_DN42389_c0_g1_i1	dmso	control	-1,2718955	0,18048605	0,00161871	0,01104624
TRINITY_DN44929_c0_g1_i1	dmso	control	-1,2721275	0,29170771	0,00106438	0,0078284
TRINITY_DN1909_c0_g1_i1	dmso	control	-1,2727149	3,62476764	1,41E-10	5,10E-09
TRINITY_DN13284_c0_g1_i1	dmso	control	-1,2727632	1,20785033	5,11E-05	0,00058379
TRINITY_DN5194_c0_g1_i1	dmso	control	-1,274906	4,51984302	1,75E-14	1,13E-12
TRINITY_DN4226_c0_g1_i1	dmso	control	-1,2795159	-0,0436912	0,00334134	0,01971636
TRINITY_DN38723_c0_g1_i1	dmso	control	-1,2835596	0,82287537	0,01197612	0,05354228
TRINITY_DN28712_c0_g1_i1	dmso	control	-1,2843845	0,01246673	0,00395608	0,02249826
TRINITY_DN9757_c0_g1_i1	dmso	control	-1,285365	0,93471349	0,02154299	0,08371039
TRINITY_DN28023_c0_g1_i1	dmso	control	-1,2873858	4,06432605	8,11E-10	2,59E-08
TRINITY_DN16732_c0_g1_i1	dmso	control	-1,2901793	3,90407938	2,02E-06	3,29E-05
TRINITY_DN31015_c0_g1_i1	dmso	control	-1,2904175	0,12581046	0,00157654	0,01081248
TRINITY_DN29221_c0_g1_i1	dmso	control	-1,2931045	1,89611023	3,74E-06	5,70E-05
TRINITY_DN3251_c0_g1_i1	dmso	control	-1,2931305	4,16591617	3,86E-12	1,78E-10
TRINITY_DN2732_c0_g1_i1	dmso	control	-1,2941534	3,61514114	1,21E-11	5,18E-10
TRINITY_DN21343_c0_g1_i1	dmso	control	-1,2949192	0,19557271	0,0008838	0,00668053
TRINITY_DN33614_c0_g1_i1	dmso	control	-1,2970665	1,84000279	2,75E-06	4,36E-05
TRINITY_DN17391_c0_g1_i1	dmso	control	-1,2978582	-0,2604978	0,00322325	0,01922371

TRINITY_DN34088_c0_g1_i1	dmso	control	-1,3002558	0,77821142	0,00138458	0,00971357
TRINITY_DN18205_c0_g1_i1	dmso	control	-1,3046564	0,25373023	0,00394171	0,02244302
TRINITY_DN42498_c0_g1_i1	dmso	control	-1,3047769	0,30739098	0,0018217	0,01214325
TRINITY_DN2467_c0_g1_i1	dmso	control	-1,3049103	2,25724639	4,09E-07	7,86E-06
TRINITY_DN19892_c0_g1_i1	dmso	control	-1,307004	2,14144301	6,12E-07	1,12E-05
TRINITY_DN40496_c0_g1_i1	dmso	control	-1,309849	1,89347327	2,00E-06	3,25E-05
TRINITY_DN40775_c0_g1_i1	dmso	control	-1,3105916	0,28575983	0,00194723	0,01277374
TRINITY_DN25425_c0_g1_i1	dmso	control	-1,3165539	3,09203649	2,69E-10	9,31E-09
TRINITY_DN44303_c0_g1_i1	dmso	control	-1,3326325	2,45129955	2,98E-07	5,88E-06
TRINITY_DN42653_c0_g1_i1	dmso	control	-1,3331527	2,49283174	8,11E-08	1,82E-06
TRINITY_DN54273_c0_g1_i1	dmso	control	-1,3338435	-0,2463506	0,00348062	0,02040486
TRINITY_DN22918_c0_g1_i1	dmso	control	-1,338901	1,29178987	1,66E-05	0,00021823
TRINITY_DN16161_c0_g1_i1	dmso	control	-1,3404676	3,19297021	1,70E-10	6,06E-09
TRINITY_DN53219_c0_g1_i1	dmso	control	-1,3439087	1,62135431	3,48E-06	5,33E-05
TRINITY_DN26190_c0_g1_i1	dmso	control	-1,346317	0,35997104	0,00048973	0,00406092
TRINITY_DN18044_c0_g1_i1	dmso	control	-1,3539846	0,17535562	0,00283153	0,01726526
TRINITY_DN33111_c0_g1_i1	dmso	control	-1,3607207	2,01785313	2,89E-06	4,54E-05
TRINITY_DN47270_c0_g1_i1	dmso	control	-1,3625273	1,32160452	2,86E-05	0,00034997
TRINITY_DN26654_c0_g1_i1	dmso	control	-1,3625771	-0,1708037	0,00137482	0,00966385
TRINITY_DN9320_c0_g1_i1	dmso	control	-1,3653259	3,97811699	1,42E-12	7,18E-11
TRINITY_DN12614_c0_g1_i1	dmso	control	-1,3656687	2,91041916	8,54E-08	1,91E-06
TRINITY_DN10168_c0_g1_i1	dmso	control	-1,3695618	0,47570787	0,00023723	0,00218771
TRINITY_DN27973_c0_g1_i1	dmso	control	-1,3697852	1,63060811	3,82E-06	5,80E-05
TRINITY_DN21296_c0_g1_i1	dmso	control	-1,3699636	4,01288447	2,57E-13	1,44E-11
TRINITY_DN31436_c0_g1_i1	dmso	control	-1,3700628	0,88469425	0,00018728	0,00178332
TRINITY_DN24957_c0_g1_i1	dmso	control	-1,3750406	0,56367876	0,00018114	0,00173169
TRINITY_DN2657_c0_g1_i1	dmso	control	-1,3758724	4,57932416	1,03E-12	5,38E-11
TRINITY_DN3235_c0_g1_i1	dmso	control	-1,3772084	1,97093098	2,17E-07	4,44E-06
TRINITY_DN33067_c0_g1_i1	dmso	control	-1,3866591	5,54365599	8,55E-25	1,54E-22
TRINITY_DN41855_c0_g1_i1	dmso	control	-1,3875131	0,90482994	0,00042711	0,00361642
TRINITY_DN7513_c0_g1_i1	dmso	control	-1,3912662	2,45891325	4,37E-08	1,03E-06

TRINITY_DN26892_c0_g1_i1	dmso	control	-1,3933533	0,96311321	6,62E-05	0,00073422
TRINITY_DN31039_c0_g1_i1	dmso	control	-1,3955954	-0,3401752	0,00511533	0,02742797
TRINITY_DN23492_c0_g1_i1	dmso	control	-1,4038463	2,46117096	2,29E-08	5,63E-07
TRINITY_DN32042_c0_g1_i1	dmso	control	-1,4062302	1,75791667	2,97E-06	4,65E-05
TRINITY_DN7177_c0_g1_i1	dmso	control	-1,4185538	2,62403579	1,07E-09	3,32E-08
TRINITY_DN23325_c0_g1_i1	dmso	control	-1,4188112	2,3851957	9,74E-09	2,58E-07
TRINITY_DN24275_c0_g1_i1	dmso	control	-1,4230994	0,5780886	0,00146604	0,01019101
TRINITY_DN5247_c0_g1_i1	dmso	control	-1,4250667	6,52089629	5,95E-32	1,95E-29
TRINITY_DN34197_c0_g1_i1	dmso	control	-1,427558	0,09246115	0,00717266	0,03576435
TRINITY_DN20578_c0_g1_i1	dmso	control	-1,4300559	2,5807925	1,74E-09	5,22E-08
TRINITY_DN29953_c0_g1_i1	dmso	control	-1,4349292	2,19517334	2,99E-08	7,20E-07
TRINITY_DN3082_c0_g1_i1	dmso	control	-1,4482842	4,07989838	3,93E-16	3,10E-14
TRINITY_DN36460_c0_g1_i1	dmso	control	-1,4521235	1,29871376	0,00012731	0,00128315
TRINITY_DN21580_c0_g1_i1	dmso	control	-1,4551705	1,76109527	3,50E-06	5,36E-05
TRINITY_DN19412_c0_g1_i1	dmso	control	-1,4602072	3,80454384	1,16E-13	6,73E-12
TRINITY_DN5157_c0_g1_i1	dmso	control	-1,4607631	4,54443597	1,10E-14	7,35E-13
TRINITY_DN32750_c0_g1_i1	dmso	control	-1,4621853	3,59664134	4,40E-14	2,69E-12
TRINITY_DN17374_c0_g1_i1	dmso	control	-1,4625003	0,5109203	7,24E-05	0,00079391
TRINITY_DN8787_c0_g1_i1	dmso	control	-1,4627936	4,45294029	3,83E-10	1,29E-08
TRINITY_DN38031_c0_g1_i1	dmso	control	-1,4632321	1,65775208	5,12E-07	9,56E-06
TRINITY_DN17049_c0_g1_i1	dmso	control	-1,4632818	1,31245132	0,00055589	0,00453148
TRINITY_DN14507_c0_g1_i1	dmso	control	-1,4677434	1,35611357	1,32E-05	0,00017926
TRINITY_DN25884_c0_g1_i1	dmso	control	-1,4695278	2,31078468	1,29E-08	3,34E-07
TRINITY_DN4576_c0_g1_i1	dmso	control	-1,4733505	2,92167341	2,31E-11	9,48E-10
TRINITY_DN33631_c0_g1_i1	dmso	control	-1,4783021	1,03119973	4,20E-05	0,00049164
TRINITY_DN24163_c0_g1_i1	dmso	control	-1,4839827	3,166919	3,13E-09	8,89E-08
TRINITY_DN24644_c0_g1_i1	dmso	control	-1,4927291	0,10120051	0,00034347	0,00301057
TRINITY_DN52045_c0_g1_i1	dmso	control	-1,4928878	4,53262464	1,43E-12	7,19E-11
TRINITY_DN38330_c0_g1_i1	dmso	control	-1,5007974	0,44294274	6,30E-05	0,00070443
TRINITY_DN51212_c0_g1_i1	dmso	control	-1,5186437	4,22822755	1,16E-09	3,58E-08
TRINITY_DN66751_c0_g1_i1	dmso	control	-1,5193439	0,02199912	0,00027864	0,00251559

TRINITY_DN21941_c0_g1_i1	dmso	control	-1,5207826	4,66111213	1,46E-14	9,51E-13
TRINITY_DN56695_c0_g1_i1	dmso	control	-1,5273175	2,50716906	1,46E-07	3,13E-06
TRINITY_DN8667_c0_g1_i1	dmso	control	-1,5303457	0,64458616	2,63E-05	0,0003257
TRINITY_DN21817_c0_g1_i1	dmso	control	-1,5305839	1,42276746	2,31E-06	3,71E-05
TRINITY_DN69507_c0_g1_i1	dmso	control	-1,5417776	-0,3095677	0,00049483	0,00409614
TRINITY_DN24007_c0_g1_i1	dmso	control	-1,5431307	1,44221009	5,05E-06	7,55E-05
TRINITY_DN26898_c0_g1_i1	dmso	control	-1,5617081	-0,2467122	0,00053732	0,00441003
TRINITY_DN6790_c0_g1_i1	dmso	control	-1,563797	5,51726997	2,22E-29	5,93E-27
TRINITY_DN28277_c0_g1_i1	dmso	control	-1,5888189	-0,0579581	0,00039732	0,00339804
TRINITY_DN7720_c0_g1_i1	dmso	control	-1,5897035	3,36555946	9,46E-15	6,42E-13
TRINITY_DN47972_c0_g1_i1	dmso	control	-1,5935952	2,87165436	7,25E-11	2,73E-09
TRINITY_DN777_c0_g1_i1	dmso	control	-1,5943813	7,0430169	2,66E-08	6,45E-07
TRINITY_DN25283_c0_g1_i1	dmso	control	-1,6039566	0,64585088	1,39E-05	0,0001876
TRINITY_DN9185_c0_g1_i1	dmso	control	-1,6059661	1,4616139	2,07E-05	0,00026406
TRINITY_DN36106_c0_g1_i1	dmso	control	-1,6171761	-0,0987704	0,00013727	0,00137315
TRINITY_DN49374_c0_g1_i1	dmso	control	-1,6190368	3,35140889	1,11E-11	4,78E-10
TRINITY_DN43949_c0_g1_i1	dmso	control	-1,622843	0,05356366	0,0001022	0,00106364
TRINITY_DN10325_c0_g1_i1	dmso	control	-1,6347514	-0,0919707	0,00037491	0,00323705
TRINITY_DN14828_c0_g1_i1	dmso	control	-1,6431584	2,39675378	4,30E-11	1,69E-09
TRINITY_DN34207_c0_g1_i1	dmso	control	-1,6469036	1,46721684	8,12E-07	1,45E-05
TRINITY_DN13639_c0_g1_i1	dmso	control	-1,6486355	4,29830083	6,13E-19	6,47E-17
TRINITY_DN38211_c0_g1_i1	dmso	control	-1,6556323	1,25317292	2,04E-07	4,21E-06
TRINITY_DN21370_c0_g1_i1	dmso	control	-1,6583692	1,30893496	3,07E-07	6,03E-06
TRINITY_DN26720_c0_g1_i1	dmso	control	-1,6645518	-0,3400056	0,00048178	0,00400652
TRINITY_DN13126_c0_g1_i1	dmso	control	-1,6677675	3,1874759	1,14E-15	8,37E-14
TRINITY_DN34341_c0_g1_i1	dmso	control	-1,6792378	1,36508697	0,00015648	0,00153034
TRINITY_DN23139_c0_g1_i1	dmso	control	-1,6836103	3,53583517	2,63E-14	1,67E-12
TRINITY_DN22105_c0_g1_i1	dmso	control	-1,7094557	0,88952118	5,89E-07	1,09E-05
TRINITY_DN47302_c0_g1_i1	dmso	control	-1,722513	-0,3758747	0,00028882	0,00259453
TRINITY_DN6104_c0_g1_i1	dmso	control	-1,7275231	5,045287	3,09E-31	9,70E-29
TRINITY_DN17585_c0_g1_i1	dmso	control	-1,7356953	3,76808628	8,03E-09	2,14E-07

TRINITY_DN27987_c0_g1_i1	dmso	control	-1,7599448	0,95595183	3,98E-07	7,65E-06
TRINITY_DN10002_c0_g1_i1	dmso	control	-1,7615785	3,13145063	3,33E-14	2,08E-12
TRINITY_DN14758_c0_g1_i1	dmso	control	-1,7652247	1,53702245	2,19E-07	4,46E-06
TRINITY_DN23601_c0_g1_i1	dmso	control	-1,7770613	5,86211198	1,78E-36	7,78E-34
TRINITY_DN3171_c0_g1_i1	dmso	control	-1,778011	2,20806683	1,08E-11	4,69E-10
TRINITY_DN67948_c0_g1_i1	dmso	control	-1,7877453	0,25723549	7,96E-06	0,00011376
TRINITY_DN14158_c0_g1_i1	dmso	control	-1,8003706	0,83736957	7,38E-07	1,33E-05
TRINITY_DN21721_c0_g1_i1	dmso	control	-1,8160521	2,0771376	2,57E-11	1,04E-09
TRINITY_DN1421_c0_g1_i1	dmso	control	-1,82457	6,66278009	1,55E-52	1,50E-49
TRINITY_DN17393_c0_g1_i1	dmso	control	-1,8252759	-0,3727598	8,69E-05	0,00092314
TRINITY_DN10090_c0_g1_i1	dmso	control	-1,8292768	2,99032469	2,96E-13	1,65E-11
TRINITY_DN38048_c0_g1_i1	dmso	control	-1,8306881	-0,2887908	0,00021172	0,00197694
TRINITY_DN9375_c0_g1_i1	dmso	control	-1,8382497	1,36634084	2,08E-08	5,15E-07
TRINITY_DN24117_c0_g1_i1	dmso	control	-1,8454749	2,69158098	1,64E-12	8,04E-11
TRINITY_DN23039_c0_g1_i1	dmso	control	-1,8535922	1,52078583	7,65E-09	2,05E-07
TRINITY_DN44637_c0_g1_i1	dmso	control	-1,8549128	-0,2766318	3,34E-05	0,00040049
TRINITY_DN28350_c0_g1_i1	dmso	control	-1,8580744	-0,0311456	1,36E-05	0,00018423
TRINITY_DN8795_c0_g1_i1	dmso	control	-1,8691283	4,68504762	1,42E-15	1,02E-13
TRINITY_DN14858_c0_g1_i1	dmso	control	-1,8735625	4,62708281	2,10E-23	3,28E-21
TRINITY_DN64516_c0_g1_i1	dmso	control	-1,8785093	0,83985123	1,30E-07	2,83E-06
TRINITY_DN74971_c0_g1_i1	dmso	control	-1,886623	0,79797118	2,02E-06	3,29E-05
TRINITY_DN5696_c0_g1_i1	dmso	control	-1,9031183	5,9054057	1,70E-43	1,02E-40
TRINITY_DN12456_c0_g1_i1	dmso	control	-1,9046605	4,60007946	3,25E-13	1,80E-11
TRINITY_DN9421_c0_g1_i1	dmso	control	-1,9075127	-0,3062754	4,73E-05	0,00054658
TRINITY_DN50846_c0_g1_i1	dmso	control	-1,910513	3,6768515	1,03E-21	1,40E-19
TRINITY_DN21153_c0_g1_i1	dmso	control	-1,9114101	2,59613469	1,06E-14	7,14E-13
TRINITY_DN59235_c0_g1_i1	dmso	control	-1,9136525	0,59764242	3,34E-07	6,52E-06
TRINITY_DN2635_c0_g1_i1	dmso	control	-1,9256854	4,56902315	3,77E-26	7,35E-24
TRINITY_DN21437_c0_g1_i1	dmso	control	-1,9283837	5,53637092	4,64E-44	3,05E-41
TRINITY_DN12754_c0_g1_i1	dmso	control	-1,9342018	2,14354786	6,59E-13	3,53E-11
TRINITY_DN19800_c0_g1_i1	dmso	control	-1,9375036	2,70332073	7,84E-16	5,96E-14

TRINITY_DN25756_c0_g1_i1	dmso	control	-1,9418953	0,37241726	7,99E-07	1,43E-05
TRINITY_DN3237_c0_g1_i1	dmso	control	-1,9498514	0,33295662	1,01E-06	1,76E-05
TRINITY_DN7407_c0_g1_i1	dmso	control	-1,9811126	1,00609724	1,28E-08	3,32E-07
TRINITY_DN31400_c0_g1_i1	dmso	control	-1,9811489	0,00784221	1,46E-05	0,00019615
TRINITY_DN78228_c0_g1_i1	dmso	control	-1,9972774	2,90527806	2,80E-09	8,12E-08
TRINITY_DN24021_c0_g1_i1	dmso	control	-2,0018185	1,41879119	2,19E-09	6,51E-08
TRINITY_DN10365_c0_g1_i1	dmso	control	-2,0190255	3,3804062	1,53E-17	1,39E-15
TRINITY_DN18557_c0_g1_i1	dmso	control	-2,0556947	1,26340794	5,84E-08	1,35E-06
TRINITY_DN25169_c0_g1_i1	dmso	control	-2,060068	1,52387416	1,48E-08	3,79E-07
TRINITY_DN35520_c0_g1_i1	dmso	control	-2,0767919	2,38155429	9,37E-16	7,01E-14
TRINITY_DN39799_c0_g1_i1	dmso	control	-2,0849527	2,9115725	4,95E-19	5,30E-17
TRINITY_DN37691_c0_g1_i1	dmso	control	-2,1368113	3,99412952	2,95E-29	7,75E-27
TRINITY_DN15325_c0_g1_i1	dmso	control	-2,142626	3,100655	2,44E-21	3,15E-19
TRINITY_DN25125_c0_g1_i1	dmso	control	-2,1528755	1,87102527	2,12E-11	8,80E-10
TRINITY_DN33085_c0_g1_i1	dmso	control	-2,1593497	1,12070016	1,30E-10	4,72E-09
TRINITY_DN39169_c0_g1_i1	dmso	control	-2,1601665	1,5489538	2,80E-12	1,33E-10
TRINITY_DN8748_c0_g1_i1	dmso	control	-2,2105711	7,90730979	2,25E-80	1,63E-76
TRINITY_DN19749_c0_g1_i1	dmso	control	-2,2210251	2,93646122	7,76E-21	9,75E-19
TRINITY_DN59079_c0_g1_i1	dmso	control	-2,221818	0,18867934	2,53E-07	5,09E-06
TRINITY_DN23661_c0_g1_i1	dmso	control	-2,2391755	2,02125672	1,24E-09	3,79E-08
TRINITY_DN24388_c0_g1_i1	dmso	control	-2,2411245	2,34614062	2,58E-12	1,23E-10
TRINITY_DN44378_c0_g1_i1	dmso	control	-2,2524207	2,24098605	1,93E-12	9,40E-11
TRINITY_DN56549_c0_g1_i1	dmso	control	-2,264294	-0,2627484	3,37E-06	5,18E-05
TRINITY_DN32283_c0_g1_i1	dmso	control	-2,2784105	1,41388213	5,53E-12	2,47E-10
TRINITY_DN17761_c0_g1_i1	dmso	control	-2,3141121	-0,3073422	3,41E-06	5,24E-05
TRINITY_DN13053_c0_g1_i1	dmso	control	-2,3218171	4,17578213	5,35E-36	2,21E-33
TRINITY_DN26294_c0_g1_i1	dmso	control	-2,3408386	-0,2215213	1,11E-06	1,91E-05
TRINITY_DN31312_c0_g1_i1	dmso	control	-2,3616133	1,29542455	3,94E-10	1,31E-08
TRINITY_DN40328_c0_g1_i1	dmso	control	-2,4044053	3,4342078	1,91E-29	5,32E-27
TRINITY_DN14469_c0_g1_i1	dmso	control	-2,4064193	1,26500885	1,50E-11	6,35E-10
TRINITY_DN29229_c0_g1_i1	dmso	control	-2,4200009	-0,1646376	1,85E-07	3,87E-06

TRINITY_DN68066_c0_g1_i1	dmso	control	-2,422584	2,46063226	1,26E-17	1,15E-15
TRINITY_DN27144_c0_g1_i1	dmso	control	-2,4244344	1,97281394	4,70E-11	1,82E-09
TRINITY_DN21706_c0_g1_i1	dmso	control	-2,4639484	1,51851374	2,84E-10	9,82E-09
TRINITY_DN38691_c0_g1_i1	dmso	control	-2,4899862	0,13298165	5,61E-09	1,52E-07
TRINITY_DN52089_c0_g1_i1	dmso	control	-2,4924515	-0,0038601	2,07E-07	4,25E-06
TRINITY_DN27375_c0_g1_i1	dmso	control	-2,5171415	1,97329167	4,17E-18	3,94E-16
TRINITY_DN40376_c0_g1_i1	dmso	control	-2,5824961	2,13976886	3,61E-16	2,88E-14
TRINITY_DN17506_c0_g1_i1	dmso	control	-2,6107616	3,18390383	2,10E-25	3,84E-23
TRINITY_DN23443_c0_g1_i1	dmso	control	-2,6147309	-0,091664	2,57E-08	6,26E-07
TRINITY_DN28742_c0_g1_i1	dmso	control	-2,6179808	2,45230232	9,51E-16	7,08E-14
TRINITY_DN24108_c0_g1_i1	dmso	control	-2,6531884	3,82719332	1,29E-18	1,30E-16
TRINITY_DN3680_c0_g1_i1	dmso	control	-2,6706315	0,97329238	4,11E-10	1,37E-08
TRINITY_DN7826_c0_g1_i1	dmso	control	-2,7418949	1,94799193	2,05E-18	1,99E-16
TRINITY_DN13164_c0_g1_i1	dmso	control	-2,7631684	0,67144443	2,04E-12	9,87E-11
TRINITY_DN24154_c0_g1_i1	dmso	control	-2,8172172	0,17428097	1,52E-09	4,60E-08
TRINITY_DN28283_c0_g1_i1	dmso	control	-2,8553857	-0,1935742	5,06E-09	1,38E-07
TRINITY_DN49166_c0_g1_i1	dmso	control	-2,8723541	-0,5479592	6,13E-08	1,41E-06
TRINITY_DN9863_c0_g1_i1	dmso	control	-2,8879807	1,40032645	3,73E-13	2,06E-11
TRINITY_DN45567_c0_g1_i1	dmso	control	-2,9724324	-0,0931608	6,63E-10	2,14E-08
TRINITY_DN25391_c0_g1_i1	dmso	control	-3,033361	0,72028446	2,73E-14	1,73E-12
TRINITY_DN13907_c0_g1_i1	dmso	control	-3,1108884	3,91883705	1,17E-16	9,64E-15
TRINITY_DN42487_c0_g1_i1	dmso	control	-3,1471458	-0,1923808	3,44E-10	1,17E-08
TRINITY_DN57390_c0_g1_i1	dmso	control	-3,2151252	-0,1308907	1,18E-09	3,63E-08
TRINITY_DN45766_c0_g1_i1	dmso	control	-3,4214995	2,15508335	3,73E-18	3,54E-16
TRINITY_DN4159_c0_g1_i1	dmso	control	-3,5921986	1,44576841	1,30E-19	1,48E-17
TRINITY_DN43485_c0_g1_i1	dmso	control	-3,6287686	-0,452026	1,09E-10	4,05E-09
TRINITY_DN22995_c0_g1_i1	dmso	control	-3,6495434	3,88202601	1,10E-60	1,95E-57
TRINITY_DN48321_c0_g1_i1	dmso	control	-3,8729054	1,20475886	1,61E-10	5,76E-09
TRINITY_DN11536_c0_g1_i1	dmso	control	-3,9853454	5,42369558	5,93E-125	8,57E-121
TRINITY_DN40182_c0_g1_i1	dmso	control	-4,0558585	1,69690558	1,24E-29	3,51E-27
TRINITY_DN47797_c0_g1_i1	dmso	control	-4,0749421	-0,5782928	2,57E-11	1,04E-09

TRINITY_DN24257_c0_g1_i1	dmso	control	-4,1163393	1,40514278	5,89E-24	9,78E-22
TRINITY_DN44170_c0_g1_i1	dmso	control	-4,3733345	0,37012289	1,10E-12	5,70E-11
