# Supplementary material for: Sublethal Exposure Effects of the Neonicotinoid Clothianidin Strongly Modify the Brain Transcriptome and Proteome in the Male Moth Agrotis ipsilon
Source: Insects. 2021 Feb 11;12(2):152. doi: 10.3390/insects12020152 (PMC7916958; doi:10.3390/insects12020152)
Supplement: Supplementary file 1 [file insects-12-00152-s001.tgz › TableS3.pdf]

TRINITY CODE	logFC_clothia_DMSO	logFC_clothia_control	annotation
TRINITY_DN5830_c0_g1_i1	-0,513641902	-0,725715713	Full=Acetyl-CoA acetyltransferase B, mitochondrial;
TRINITY_DN10709_c0_g1_i1	-0,520581149	-0,442197187	Full=Catalase;
TRINITY_DN4884_c0_g1_i1	-0,542534545	-0,705716908	Full=UPF0061 protein xcc-b100_1894 {ECO:0000255|HAMAP-Rule:MF_00692};^Bacteria; Proteobacteria; Gammaproteobacteria; Xanthomonadales; Xanthomonadaceae; Xanthomonas
TRINITY_DN19_c0_g1_i1	-0,569901458	-0,650555009	Full=Adenosylhomocysteinase;
TRINITY_DN18786_c0_g1_i1	-0,571570247		Full=Probable 39S ribosomal protein L45, mitochondrial;
TRINITY_DN18696_c0_g1_i1	-0,609625906	-0,442676401	0
TRINITY_DN1941_c0_g1_i1	-0,623485171	-0,886745415	Full=Perilipin-4;
TRINITY_DN5963_c0_g1_i1	-0,63532932	-0,536822159	Full=Alkaline phosphatase, tissue-nonspecific isozyme;
TRINITY_DN15717_c0_g1_i1	-0,636780777		Full=2-oxoglutarate dehydrogenase-like, mitochondrial;
TRINITY_DN224_c0_g1_i1	-0,63976921	-0,529861129	0
TRINITY_DN6720_c0_g1_i1	-0,691291574	-0,902046228	Full=D-arabinitol dehydrogenase 1;
TRINITY_DN11543_c0_g1_i1	-0,733684853	-0,538758739	0
TRINITY_DN4730_c0_g1_i1	-0,741326982	-0,531266701	Full=Mevalonate kinase;
TRINITY_DN2762_c0_g1_i1	-0,87487484	-0,64799718	0
TRINITY_DN19669_c0_g1_i1	-0,875346814	-1,228200397	Full=BolA-like protein DDB_G0274169;
TRINITY_DN14430_c0_g1_i1	-0,90842733	-0,942056234	Full=Copia protein;
TRINITY_DN9205_c0_g1_i1	-0,991980293		Full=Fumarate hydratase, mitochondrial;
TRINITY_DN22277_c0_g1_i1	-1,033765049		Full=Mannose-6-phosphate isomerase;
TRINITY_DN7069_c0_g1_i1	-1,063494271	-1,265905906	Full=Solute carrier family 46 member 3;
TRINITY_DN19532_c0_g1_i1	-1,103151648	-1,337305347	Full=Protein takeout;
TRINITY_DN35019_c0_g1_i1	-1,140092084	-0,724121858	Full=Zonadhesin;
TRINITY_DN34496_c0_g1_i1	-1,261541342	-1,454106987	Full=Esterase B1;
TRINITY_DN10489_c0_g1_i1	-1,281329447	-1,407962294	0
TRINITY_DN3287_c0_g1_i1	-1,365197223	-1,264300341	0
TRINITY_DN10545_c0_g1_i1	-1,385574484	-1,351259084	Full=Probable RNA-directed DNA polymerase from transposon BS;
TRINITY_DN35801_c0_g1_i1	-1,448523719	-1,081196088	Full=Condensin complex subunit 1;
TRINITY_DN9370_c0_g1_i1	-1,673941476	-1,721808867	0
TRINITY_DN18812_c0_g1_i1	-1,701359832	-1,448433673	Full=Farnesoate epoxidase {ECO:0000305};

TRINITY_DN12841_c0_g1_i1	-1,735143456	-1,847724461	Full=40S ribosomal protein S12, mitochondrial;	
TRINITY_DN32801_c0_g1_i1	-1,778031857	-2,309450409	Full=Elongation of very long chain fatty acids protein 7 {ECO:0000255|HAMAP-Rule:MF_03207, ECO:0000305};	
TRINITY_DN37927_c0_g1_i1	-1,783490336	-2,312550939	Full=Endocuticle structural glycoprotein ABD-5;	
TRINITY_DN32791_c0_g1_i1	-2,073754051		Full=Ejaculatory bulb-specific protein 3;	
TRINITY_DN44850_c0_g1_i1	-2,082048891	-1,640420101	Full=CCR4-NOT transcription complex subunit 1;	
TRINITY_DN62406_c0_g1_i1	-2,113532993	-1,826508688		0
TRINITY_DN33988_c0_g1_i1	-2,147000881	-2,486090066	Full=Farnesoate epoxidase {ECO:0000305};	
TRINITY_DN9591_c0_g1_i1		-2,020068887		0
TRINITY_DN57731_c0_g1_i1		-1,969575731		0
TRINITY_DN48162_c0_g1_i1		-1,825079603	Full=RutC family protein C23G10.2;	
TRINITY_DN36009_c0_g1_i1		-1,777345642	Full=Protein-tyrosine sulfotransferase;	
TRINITY_DN52557_c0_g1_i1		-1,663113615		0
TRINITY_DN73860_c0_g1_i1		-1,595329072		0
TRINITY_DN63597_c0_g1_i1		-1,552194434	Full=Retrovirus-related Pol polyprotein from transposon TNT 1-94;	
TRINITY_DN45251_c0_g1_i1		-1,542749215		0
TRINITY_DN37602_c0_g1_i1		-1,528539656	Full=D-amino-acid oxidase;	
TRINITY_DN52570_c0_g1_i1		-1,515313295	Full=Protein Skeletor, isoforms B/C;	
TRINITY_DN2872_c0_g1_i1		-1,514107452	Full=mRNA export factor;	
TRINITY_DN27963_c0_g1_i1		-1,48614603	Full=Probable cytochrome P450 6a13;	
TRINITY_DN39050_c0_g1_i1		-1,471120839	Full=Copia protein;	
TRINITY_DN26418_c0_g1_i1		-1,443791536	Full=Zinc finger protein 554;	
TRINITY_DN20937_c0_g1_i1		-1,420795449	Full=Cytochrome P450 4c3;	
TRINITY_DN41552_c0_g1_i1		-1,416548266		0
TRINITY_DN47964_c0_g1_i1		-1,393462361		0
TRINITY_DN29880_c0_g1_i1		-1,371808831	Full=Facilitated trehalose transporter Tret1 {ECO:0000303|PubMed:20035867};	
TRINITY_DN33195_c0_g1_i1		-1,32554914	Full=Glia maturation factor beta;	
TRINITY_DN54768_c0_g1_i1		-1,310088827	Full=Serine incorporator 1;	
TRINITY_DN31189_c0_g1_i1		-1,31008179	Full=Splicing factor 1;	
TRINITY_DN17813_c0_g1_i1		-1,290288121		0
TRINITY_DN46000_c0_g1_i1		-1,281406117		0

TRINITY_DN67295_c0_g1_i1	-1,27866098		0
TRINITY_DN37510_c0_g1_i1	-1,235128636		0
TRINITY_DN34640_c0_g1_i1	-1,228664758	Full=Oocyte zinc finger protein XlCOF26;	
TRINITY_DN36002_c0_g1_i1	-1,221247347	Full=Lysosomal acid phosphatase;	
TRINITY_DN50955_c0_g1_i1	-1,20755057	Full=Ubiquitin conjugation factor E4 B {ECO:0000305};	
TRINITY_DN44849_c0_g1_i1	-1,196824664	Full=Cystathionine beta-synthase {ECO:0000305};	
TRINITY_DN22655_c0_g1_i1	-1,196069464	Full=Very long-chain specific acyl-CoA dehydrogenase, mitochondrial;	
TRINITY_DN4543_c0_g1_i1	-1,189910587	Full=DNA replication licensing factor mcm7;	
TRINITY_DN27817_c0_g1_i1	-1,189334492		0
TRINITY_DN7348_c0_g1_i1	-1,175124346	Full=Retrovirus-related Pol polyprotein from transposon TNT 1-94;	
TRINITY_DN34041_c0_g1_i1	-1,164139921		0
TRINITY_DN37703_c0_g1_i1	-1,158359891	Full=Cytochrome P450 6B7;	
TRINITY_DN56752_c0_g1_i1	-1,152762601		0
TRINITY_DN47838_c0_g1_i1	-1,1280387		0
TRINITY_DN20711_c0_g1_i1	-1,125122033	Full=Protein lethal(2)essential for life;	
TRINITY_DN35001_c0_g1_i1	-1,114169546		0
TRINITY_DN49009_c0_g1_i1	-1,113141069		0
TRINITY_DN45184_c0_g1_i1	-1,110875489	Full=E3 ubiquitin-protein ligase HECTD1;	
TRINITY_DN53086_c0_g1_i1	-1,100797479		0
TRINITY_DN30346_c0_g1_i1	-1,099975516		0
TRINITY_DN33202_c0_g1_i1	-1,098505524		0
TRINITY_DN33981_c0_g1_i1	-1,095843792		0
TRINITY_DN20276_c0_g1_i1	-1,074992801	Full=Fructose-1,6-bisphosphatase 1;	
TRINITY_DN32771_c0_g1_i1	-1,074310511		0
TRINITY_DN16217_c0_g1_i1	-1,073856776	Full=Membrane-associated progesterone receptor component 2;	
TRINITY_DN3733_c0_g1_i1	-1,073610856	Full=Protein msta, isoform A;	
TRINITY_DN21720_c0_g1_i1	-1,062186842		0
TRINITY_DN23451_c0_g1_i1	-1,058121874	Full=Ras-related protein Rab-32;	
TRINITY_DN22829_c0_g1_i1	-1,05724383	Full=AT-rich interactive domain-containing protein 1A;	
TRINITY_DN21151_c0_g1_i1	-1,057019494		0
TRINITY_DN32473_c0_g1_i1	-1,05513924	Full=Dual specificity protein phosphatase 23;	

TRINITY_DN55965_c0_g1_i1	-1,054773644	Full=F-box only protein 11;	
TRINITY_DN24380_c0_g1_i1	-1,041734693	Full=Brahma-associated protein of 60 kDa;	
TRINITY_DN15632_c0_g1_i1	-1,033722463	Full=Protein pigeon;	
TRINITY_DN33400_c0_g1_i1	-1,02914242	Full=5-hydroxytryptamine receptor;	
TRINITY_DN14750_c0_g1_i1	-1,027065665	Full=Poly(U)-binding-splicing factor half pint;	
TRINITY_DN41250_c0_g1_i1	-1,026338677	Full=Macrophage erythroblast attacher;	
TRINITY_DN21996_c0_g1_i1	-1,021689143	Full=Eclosion hormone;	
TRINITY_DN20175_c0_g1_i1	-1,011301077	Full=ATP-binding cassette sub-family A member 3;	
TRINITY_DN34530_c0_g1_i1	-1,007517224		0
TRINITY_DN21522_c0_g1_i1	-1,004374832	Full=(E3-independent) E2 ubiquitin-conjugating enzyme;	
TRINITY_DN3720_c0_g1_i1	-1,004286319	Full=Monocarboxylate transporter 12;	
TRINITY_DN21134_c0_g1_i1	-1,001265033	Full=Lipopolysaccharide-induced tumor necrosis factor-alpha factor homolog;	
TRINITY_DN21971_c0_g1_i1	-0,989135572		0
TRINITY_DN19718_c0_g1_i1	-0,985647264	Full=Hemolymph lipopolysaccharide-binding protein;	
TRINITY_DN34105_c0_g1_i1	-0,971997957	Full=OCIA domain-containing protein 1;	
TRINITY_DN25820_c0_g1_i1	-0,969939556		0
TRINITY_DN34383_c0_g1_i1	-0,964500741	Full=Cytochrome P450 4C1;	
TRINITY_DN46386_c0_g1_i1	-0,960304355		0
TRINITY_DN27671_c0_g1_i1	-0,956631868		0
TRINITY_DN12496_c0_g1_i1	-0,951682777	Full=Annexin B9;	
TRINITY_DN13662_c0_g1_i1	-0,950073107		0
TRINITY_DN26208_c0_g1_i1	-0,947155393	Full=Retrovirus-related Pol polyprotein from type-2 retrotransposable element R2DM;	
TRINITY_DN15290_c0_g1_i1	-0,936031547	Full=Cystathionine beta-synthase {ECO:0000305};	
TRINITY_DN16883_c0_g1_i1	-0,924392323		0
TRINITY_DN8091_c0_g1_i1	-0,917999507		0
TRINITY_DN7376_c0_g1_i1	-0,913216932	Full=DDB1- and CUL4-associated factor 13;	
TRINITY_DN20516_c0_g1_i1	-0,910908533	Full=Kynurenine 3-monooxygenase {ECO:0000255|HAMAP-Rule:MF_03018};	
TRINITY_DN3529_c0_g1_i1	-0,910865386	Full=Very long-chain specific acyl-CoA dehydrogenase, mitochondrial;	
TRINITY_DN43024_c0_g1_i1	-0,910219291	Full=Transmembrane protein 184C;	
TRINITY_DN32762_c0_g1_i1	-0,9089199	Full=Zinc finger protein 2;	

TRINITY_DN24346_c0_g1_i1	-0,908119335	Full=Protein stoned-B;	
TRINITY_DN9464_c0_g1_i1	-0,90693156		0
TRINITY_DN17472_c0_g1_i1	-0,904950526		0
TRINITY_DN1185_c0_g1_i1	-0,902828678	Full=Coiled-coil domain-containing protein 12;	
TRINITY_DN78118_c0_g1_i1	-0,902652632	Full=SH3 domain-binding glutamic acid-rich protein homolog;	
TRINITY_DN24001_c0_g1_i1	-0,895184095		0
TRINITY_DN8817_c0_g1_i1	-0,892239133		0
TRINITY_DN19719_c0_g1_i1	-0,888800601		0
TRINITY_DN22064_c0_g1_i1	-0,884049698	Full=Ribonuclease P protein subunit p40;	
TRINITY_DN19380_c0_g1_i1	-0,868888348	Full=Ecdysteroid UDP-glucosyltransferase;^Viruses; dsDNA viruses, no RNA stage; Baculoviridae; Alphabaculovirus	
TRINITY_DN21182_c0_g1_i1	-0,864171025	Full=Leucine-rich repeat-containing protein C10orf11 homolog;	
TRINITY_DN32910_c0_g1_i1	-0,84304084		0
TRINITY_DN19775_c0_g1_i1	-0,841366524	Full=28S ribosomal protein S2, mitochondrial;	
TRINITY_DN3399_c0_g1_i1	-0,833315505	Full=Potassium channel subfamily K member 18;	
TRINITY_DN6891_c0_g1_i1	-0,831575023	Full=Glucose dehydrogenase [FAD, quinone];	
TRINITY_DN20079_c0_g1_i1	-0,82525307		0
TRINITY_DN25896_c0_g1_i1	-0,82297246	Full=Probable RNA-directed DNA polymerase from transposon BS;	
TRINITY_DN4348_c0_g1_i1	-0,814305212	Full=KAT8 regulatory NSL complex subunit 1;	
TRINITY_DN4809_c0_g1_i1	-0,814165589	Full=Protein lethal(2)essential for life;	
TRINITY_DN18994_c0_g1_i1	-0,8136373	Full=Fork head domain transcription factor slp2;	
TRINITY_DN15415_c0_g1_i1	-0,812983731	Full=Kelch-like protein diablo {ECO:0000250|UniProtKB:Q9VUU5};	
TRINITY_DN53395_c0_g1_i1	-0,811465141		0
TRINITY_DN18890_c0_g1_i1	-0,806442829		0
TRINITY_DN19221_c0_g1_i1	-0,806038348	Full=Putative OPA3-like protein CG13603;	
TRINITY_DN21928_c0_g1_i1	-0,794791029	Full=Golgi SNAP receptor complex member 1;	
TRINITY_DN24711_c0_g1_i1	-0,794732346		0
TRINITY_DN8335_c0_g1_i1	-0,774411922	Full=Transcription factor SOX-2;	
TRINITY_DN3669_c0_g1_i1	-0,75749847	Full=Reticulon-3-B;	
TRINITY_DN22300_c0_g1_i1	-0,757011027		0
TRINITY_DN16597_c0_g1_i1	-0,755868879		0

TRINITY_DN19305_c0_g1_i1	-0,755515714	Full=Protein HGH1 homolog;	
TRINITY_DN14880_c0_g1_i1	-0,754711385	Full=E3 ubiquitin-protein ligase RNF31;	
TRINITY_DN19747_c0_g1_i1	-0,753008947	Full=17-beta-hydroxysteroid dehydrogenase 14;	
TRINITY_DN2514_c0_g1_i1	-0,745349969	Full=Transposable element Tcb2 transposase;	
TRINITY_DN12873_c0_g1_i1	-0,742620116		0
TRINITY_DN20149_c0_g1_i1	-0,735517231	Full=THAP domain-containing protein 4;	
TRINITY_DN11696_c0_g1_i1	-0,733666074	Full=Set1/Ash2 histone methyltransferase complex subunit ASH2 {ECO:0000303|PubMed:21694722};	
TRINITY_DN36247_c0_g1_i1	-0,729064216	Full=Heat shock protein beta-6;	
TRINITY_DN1008_c0_g1_i1	-0,726796764	Full=Uncharacterized kinase-like protein D1044.1;	
TRINITY_DN7747_c0_g1_i1	-0,726407963	Full=Phosphatidylcholine:ceramide cholinephosphotransferase 1;	
TRINITY_DN32999_c0_g1_i1	-0,722523468	Full=15 kDa selenoprotein;	
TRINITY_DN21216_c0_g1_i1	-0,716905716		0
TRINITY_DN12072_c0_g1_i1	-0,712590178		0
TRINITY_DN1903_c0_g1_i1	-0,70549103	Full=Probable tRNA N6-adenosine threonylcarbamoyltransferase, mitochondrial {ECO:0000255|HAMAP-Rule:MF_03179};	
TRINITY_DN18714_c0_g1_i1	-0,703271313	Full=Guanine nucleotide-binding protein subunit beta-like protein;	
TRINITY_DN14109_c0_g1_i1	-0,700207402		0
TRINITY_DN11579_c0_g1_i1	-0,69574151	Full=ATP-binding cassette sub-family F member 3;	
TRINITY_DN11305_c0_g1_i1	-0,694500408		0
TRINITY_DN20657_c0_g1_i1	-0,693480244	Full=Aldo-keto reductase AKR2E4;	
TRINITY_DN24011_c0_g1_i1	-0,689068691		0
TRINITY_DN178_c0_g1_i1	-0,688509183	Full=Sodium/potassium-transporting ATPase subunit beta-2;	
TRINITY_DN1472_c0_g1_i1	-0,68836351	Full=Putative deoxyribonuclease TATDN1;	
TRINITY_DN17209_c0_g1_i1	-0,688025898	Full=Multifunctional methyltransferase subunit TRM112-like protein;	
TRINITY_DN14169_c0_g1_i1	-0,68463568		0
TRINITY_DN6746_c0_g1_i1	-0,683815524	Full=DNA polymerase alpha subunit B;	
TRINITY_DN17579_c0_g1_i1	-0,679274504	Full=Armadillo segment polarity protein;	
TRINITY_DN11463_c0_g1_i1	-0,672049538	Full=Transposon Ty3-I Gag-Pol polyprotein;	
TRINITY_DN26843_c0_g1_i1	-0,671625358	Full=Nascent polypeptide-associated complex subunit alpha;	
TRINITY_DN21452_c0_g1_i1	-0,669386926	Full=Acyl-CoA Delta(11) desaturase;	
TRINITY_DN5783_c0_g1_i1	-0,667098348	Full=Importin-5;	

TRINITY_DN4737_c0_g1_i1	-0,661726229	Full=ER membrane protein complex subunit 7;	
TRINITY_DN8834_c0_g1_i1	-0,661492791		0
TRINITY_DN1358_c0_g1_i1	-0,660900681	Full=28S ribosomal protein S34, mitochondrial;	
TRINITY_DN11096_c0_g1_i1	-0,660209971	Full=Cathepsin L;	
TRINITY_DN15408_c0_g1_i1	-0,658208791	Full=Arginine/serine-rich coiled-coil protein 2;	
TRINITY_DN10895_c0_g1_i1	-0,657339856	Full=Mitochondrial import inner membrane translocase subunit Tim22;	
TRINITY_DN34289_c0_g1_i1	-0,656839314		0
TRINITY_DN10835_c0_g1_i1	-0,654615719	Full=40S ribosomal protein S6;	
TRINITY_DN12215_c0_g1_i1	-0,652396748	Full=Probable U3 small nucleolar RNA-associated protein 11;	
TRINITY_DN5297_c0_g1_i1	-0,651288093	Full=Electron transfer flavoprotein subunit alpha, mitochondrial;	
TRINITY_DN60962_c0_g1_i1	-0,650891639	Full=40S ribosomal protein S8;	
TRINITY_DN6021_c0_g1_i1	-0,649199318		0
TRINITY_DN13521_c0_g1_i1	-0,646971015	Full=Interleukin enhancer-binding factor 2 homolog;	
TRINITY_DN19975_c0_g1_i1	-0,64501874		0
TRINITY_DN32690_c0_g1_i1	-0,644962668		0
TRINITY_DN13143_c0_g1_i1	-0,644242954		0
TRINITY_DN2973_c0_g1_i1	-0,640371807	Full=Fragile X mental retardation syndrome-related protein 1;	
TRINITY_DN3920_c0_g1_i1	-0,637034873	Full=Putative 115 kDa protein in type-1 retrotransposable element R1DM;	
TRINITY_DN12604_c0_g1_i1	-0,636473501	Full=Heterogeneous nuclear ribonucleoprotein K;	
TRINITY_DN2660_c0_g1_i1	-0,635832639	Full=Replication factor C subunit 1;	
TRINITY_DN179_c0_g1_i1	-0,634066001	Full=Venom carboxylesterase-6;	
TRINITY_DN5682_c0_g1_i1	-0,633589312	Full=Nuclear fragile X mental retardation-interacting protein 1;	
TRINITY_DN5813_c0_g1_i1	-0,633156573	Full=RNA-binding protein 45;	
TRINITY_DN17106_c0_g1_i1	-0,632832101	Full=Xaa-Pro dipeptidase;	
TRINITY_DN6248_c0_g1_i1	-0,632055851		0
TRINITY_DN33125_c0_g1_i1	-0,62961523	Full=COX assembly mitochondrial protein homolog;	
TRINITY_DN5740_c0_g1_i1	-0,628411057	Full=Glutathione S-transferase 1;	
TRINITY_DN20194_c0_g1_i1	-0,626992504		0
TRINITY_DN7191_c0_g1_i1	-0,626147963	Full=Deoxyhypusine hydroxylase {ECO:0000255|HAMAP-Rule:MF_03101};	
TRINITY_DN1684_c0_g1_i1	-0,614647461	Full=Putative inorganic phosphate cotransporter;	
TRINITY_DN18560_c0_g1_i1	-0,614438281		0

TRINITY_DN5545_c0_g1_i1	-0,611475274	Full=Peptidoglycan-recognition protein LB;	
TRINITY_DN19563_c0_g1_i1	-0,610114157	Full=Probable 28S ribosomal protein S26, mitochondrial;	
TRINITY_DN11314_c0_g1_i1	-0,610077123	Full=Protein SMG9;	
TRINITY_DN18661_c0_g1_i1	-0,609170134		0
TRINITY_DN19231_c0_g1_i1	-0,609063638		0
TRINITY_DN7458_c0_g1_i1	-0,607697153	Full=Ecdysone-inducible protein E75;	
TRINITY_DN12361_c0_g1_i1	-0,607322947		0
TRINITY_DN6082_c0_g1_i1	-0,60724397	Full=Cystinosin homolog;	
TRINITY_DN19423_c0_g1_i1	-0,600317954	Full=Probable proline--tRNA ligase, mitochondrial;	
TRINITY_DN34012_c0_g1_i1	-0,599535674	Full=Regulator of G-protein signaling 9;	
TRINITY_DN16691_c0_g1_i1	-0,598584912	Full=Echinoderm microtubule-associated protein-like 2;	
TRINITY_DN4822_c0_g1_i1	-0,59847525	Full=Cell differentiation protein RCD1 homolog;	
TRINITY_DN19178_c0_g1_i1	-0,593794606	Full=RNA-binding protein 39;	
TRINITY_DN11306_c0_g1_i1	-0,592979727	Full=60S ribosomal protein L4;	
TRINITY_DN11421_c0_g1_i1	-0,582253468	Full=U8-agatoxin-Ao1a;	
TRINITY_DN11728_c0_g1_i1	-0,577973809	Full=Leucine zipper putative tumor suppressor 2 homolog {ECO:0000255|HAMAP-Rule:MF_03026};	
TRINITY_DN3075_c0_g1_i1	-0,57671918	Full=E3 ubiquitin-protein ligase hyd;	
TRINITY_DN10748_c0_g1_i1	-0,576633642	Full=NEDD8-activating enzyme E1 regulatory subunit;	
TRINITY_DN32399_c0_g1_i1	-0,575873213	Full=60S ribosomal protein L10a;	
TRINITY_DN13715_c0_g1_i1	-0,574592497	Full=Protein Tob2;	
TRINITY_DN22013_c0_g1_i1	-0,569813892	Full=RAD50-interacting protein 1;	
TRINITY_DN11932_c0_g1_i1	-0,565210866	Full=Zinc transporter 10;	
TRINITY_DN263_c0_g1_i1	-0,564570504	Full=Mitochondrial carrier homolog 2;	
TRINITY_DN32818_c0_g1_i1	-0,563049604	Full=PBAN-type neuropeptides;	
TRINITY_DN12392_c0_g1_i1	-0,561928909	Full=SOSS complex subunit C homolog;	
TRINITY_DN13306_c0_g1_i1	-0,560970925	Full=Venom acid phosphatase Acph-1;	
TRINITY_DN11386_c0_g1_i1	-0,560935136	Full=Protein CREG1;	
TRINITY_DN12559_c0_g1_i1	-0,560056642	Full=Phospholipid phosphatase 3 {ECO:0000250|UniProtKB:O14495};	
TRINITY_DN20560_c0_g1_i1	-0,5591471	Full=Acyl carrier protein, mitochondrial;	
TRINITY_DN415_c0_g1_i1	-0,557335261	Full=Cytoglobin-1;	

TRINITY_DN11400_c0_g1_i1	-0,555260656	Full=Retrovirus-related Pol polyprotein from transposon TNT 1-94;	
TRINITY_DN11843_c0_g1_i1	-0,552598378	Full=Organic solute transporter alpha-like protein;	
TRINITY_DN81_c0_g1_i1	-0,550832753	Full=Zinc-type alcohol dehydrogenase-like protein SERP1785;^Bacteria; Firmicutes; Bacilli; Bacillales; Staphylococcus	
TRINITY_DN11813_c0_g1_i1	-0,547501505	Full=DNA repair protein XRCC4;	
TRINITY_DN37010_c0_g1_i1	-0,547451597	Full=Heat shock 70 kDa protein cognate 4;	
TRINITY_DN2395_c0_g1_i1	-0,547118737	Full=Presenilin homolog;	
TRINITY_DN19316_c0_g1_i1	-0,54521564	Full=Long-chain-fatty-acid--CoA ligase 4;	
TRINITY_DN4990_c0_g1_i1	-0,54299736	Full=Proteasome subunit beta type-6;	
TRINITY_DN13499_c0_g1_i1	-0,542988735		0
TRINITY_DN11286_c0_g1_i1	-0,54030911	Full=Restin homolog;	
TRINITY_DN10953_c0_g1_i1	-0,535839652	Full=Synaptic vesicle glycoprotein 2B;	
TRINITY_DN18973_c0_g1_i1	-0,532658367	Full=Lysine-specific demethylase 3B;	
TRINITY_DN32372_c0_g1_i1	-0,531451492		0
TRINITY_DN33601_c0_g1_i1	-0,531334804	Full=60S ribosomal protein L34;	
TRINITY_DN7609_c0_g1_i1	-0,530327214		0
TRINITY_DN20722_c0_g1_i1	-0,53013434	Full=Histone-lysine N-methyltransferase ash1;	
TRINITY_DN21531_c0_g1_i1	-0,529895712	Full=Serine palmitoyltransferase 2;	
TRINITY_DN10916_c0_g1_i1	-0,527958346	Full=Non-specific lipid-transfer protein;	
TRINITY_DN5792_c0_g1_i1	-0,526313209		0
TRINITY_DN4845_c0_g1_i1	-0,525146359	Full=Nuclear transcription factor Y subunit alpha;	
TRINITY_DN18631_c0_g1_i1	-0,524633821	Full=40S ribosomal protein S2;	
TRINITY_DN4029_c0_g1_i1	-0,523702652	Full=Zinc-type alcohol dehydrogenase-like protein SERP1785;^Bacteria; Firmicutes; Bacilli; Bacillales; Staphylococcus	
TRINITY_DN18717_c0_g1_i1	-0,523387183	Full=CCHC-type zinc finger protein CG3800;	
TRINITY_DN6150_c0_g1_i1	-0,522870199	Full=Adenylate kinase {ECO:0000255|HAMAP-Rule:MF_03168};	
TRINITY_DN5986_c0_g1_i1	-0,522055569		0
TRINITY_DN14050_c0_g1_i1	-0,52108517		0
TRINITY_DN2440_c0_g1_i1	-0,519702086	Full=Zinc finger CCCH domain-containing protein 18;	
TRINITY_DN10994_c0_g1_i1	-0,519253361	Full=60S ribosomal protein L6;	
TRINITY_DN5128_c0_g1_i1	-0,518751041	Full=GTPase Era, mitochondrial;	
TRINITY_DN5070_c0_g1_i1	-0,518702361	Full=Double-stranded RNA-binding protein Staufen homolog 1;	

TRINITY_DN16_c0_g1_i1	-0,518115012	Full=Growth hormone-inducible transmembrane protein;	
TRINITY_DN32661_c0_g1_i1	-0,517067713	Full=60S ribosomal protein L28;	
TRINITY_DN11326_c0_g1_i1	-0,514483974	Full=Glutamine synthetase 2 cytoplasmic;	
TRINITY_DN610_c0_g1_i1	-0,513229103	Full=Adenylosuccinate lyase;	
TRINITY_DN18703_c0_g1_i1	-0,51225499		0
TRINITY_DN18809_c0_g1_i1	-0,51112287	Full=Probable enoyl-CoA hydratase, mitochondrial;	
TRINITY_DN11645_c0_g1_i1	-0,510247315	Full=Rhombotin-1;	
TRINITY_DN12016_c0_g1_i1	-0,509652184	Full=Host cell factor;	
TRINITY_DN19311_c0_g1_i1	-0,50821359	Full=Vacuolar protein sorting-associated protein 35;	
TRINITY_DN32455_c0_g1_i1	-0,508031094	Full=Heat shock 70 kDa protein cognate 4;	
TRINITY_DN19196_c0_g1_i1	-0,506565279	Full=60S ribosomal protein L18a;	
TRINITY_DN18648_c0_g1_i1	-0,505654089	Full=V-set and transmembrane domain-containing protein 2B;	
TRINITY_DN906_c0_g1_i1	-0,504336363		0
TRINITY_DN12550_c0_g1_i1	-0,502289526	Full=Ethanolamine-phosphate phospho-lyase;	
TRINITY_DN5449_c0_g1_i1	-0,501874144	Full=Serine protease snake;	
TRINITY_DN11812_c0_g1_i1	-0,501188592	Full=RNA 3'-terminal phosphate cyclase;	
TRINITY_DN10742_c0_g1_i1	-0,501004947	Full=60S ribosomal protein L3;	
TRINITY_DN12925_c0_g1_i1	-0,500695828	Full=Bcl-2-related ovarian killer protein;	
TRINITY_DN4767_c0_g1_i1	-0,499124677	Full=Venom carboxylesterase-6;	
TRINITY_DN12725_c0_g1_i1	-0,498412646		0
TRINITY_DN19062_c0_g1_i1	-0,494670295	Full=60S ribosomal protein L7a;	
TRINITY_DN4432_c0_g1_i1	-0,492404542	Full=Membrane-associated progesterone receptor component 2;	
TRINITY_DN18937_c0_g1_i1	-0,492312932	Full=Lysosomal Pro-X carboxypeptidase;	
TRINITY_DN19077_c0_g1_i1	-0,49174552	Full=Activating transcription factor of chaperone {ECO:0000303|PubMed:15530439};	
TRINITY_DN1204_c0_g1_i1	-0,489404015	Full=Tyrosine-protein kinase PR2;	
TRINITY_DN18850_c0_g1_i1	-0,489376967	Full=Ubiquitin carboxyl-terminal hydrolase 20;	
TRINITY_DN11667_c0_g1_i1	-0,488245531	Full=Testin;	
TRINITY_DN355_c0_g1_i1	-0,487331307	Full=Leucine-rich repeat-containing protein 20;	
TRINITY_DN238_c0_g1_i1	-0,485767349	Full=Carboxypeptidase N subunit 2;	
TRINITY_DN11501_c0_g1_i1	-0,485558515	Full=Conserved oligomeric Golgi complex subunit 8;	

TRINITY_DN11514_c0_g1_i1	-0,485066594	Full=Cytochrome c oxidase subunit 5A, mitochondrial;	
TRINITY_DN4762_c0_g1_i1	-0,484735231	Full=40S ribosomal protein S3;	
TRINITY_DN526_c0_g1_i1	-0,484719641		0
TRINITY_DN20206_c0_g1_i1	-0,484662453	Full=Glutamate receptor ionotropic, NMDA 2D;	
TRINITY_DN4688_c0_g1_i1	-0,48365764	Full=BAG domain-containing protein Samui;	
TRINITY_DN12570_c0_g1_i1	-0,482547359	Full=Glycogen-binding subunit 76A;	
TRINITY_DN746_c0_g1_i1	-0,48127912	Full=COP9 signalosome complex subunit 3;	
TRINITY_DN18857_c0_g1_i1	-0,481120284	Full=Mitogen-activated protein kinase kinase kinase 7;	
TRINITY_DN32289_c0_g1_i1	-0,480892517	Full=60S ribosomal protein L10;	
TRINITY_DN1400_c0_g1_i1	-0,480642385	Full=Stress-induced-phosphoprotein 1;	
TRINITY_DN491_c0_g1_i1	-0,480149374	Full=Probable medium-chain specific acyl-CoA dehydrogenase, mitochondrial;	
TRINITY_DN5023_c0_g1_i1	-0,479876199	Full=Thioredoxin reductase 1, mitochondrial;	
TRINITY_DN11500_c0_g1_i1	-0,478612479	Full=Protein c-ets-1-B;	
TRINITY_DN32336_c0_g1_i1	-0,478158291	Full=Cytochrome c oxidase subunit 7A1, mitochondrial;	
TRINITY_DN7124_c0_g1_i1	-0,472718135		0
TRINITY_DN10754_c0_g1_i1	-0,470576609	Full=NAD(P)H-hydrate epimerase {ECO:0000255|HAMAP-Rule:MF_03159};	
TRINITY_DN530_c0_g1_i1	-0,469658361	Full=60S acidic ribosomal protein P0;	
TRINITY_DN19689_c0_g1_i1	-0,469572267		0
TRINITY_DN21252_c0_g1_i1	-0,466463901		0
TRINITY_DN5363_c0_g1_i1	-0,465627185	Full=Alcohol dehydrogenase class-3;	
TRINITY_DN4797_c0_g1_i1	-0,463930471	Full=Ubiquitin-like protein 4A;	
TRINITY_DN765_c0_g1_i1	-0,462740185	Full=NADH dehydrogenase [ubiquinone] flavoprotein 2, mitochondrial;	
TRINITY_DN6237_c0_g1_i1	-0,462681852	Full=Lipoma-preferred partner homolog;	
TRINITY_DN11036_c0_g1_i1	-0,460893015	Full=28S ribosomal protein S7, mitochondrial;	
TRINITY_DN18718_c0_g1_i1	-0,456443313		0
TRINITY_DN570_c0_g1_i1	-0,455804816	Full=Facilitated trehalose transporter Tret1 {ECO:0000250|UniProtKB:Q7PIR5};	
TRINITY_DN10714_c0_g1_i1	-0,455440271	Full=Pyruvate dehydrogenase E1 component subunit beta, mitochondrial;	
TRINITY_DN6293_c0_g1_i1	-0,455439841		0
TRINITY_DN11607_c0_g1_i1	-0,455143961	Full=Peptide chain release factor 1-like, mitochondrial;	
TRINITY_DN18789_c0_g1_i1	-0,454929382	Full=Elongation factor 1-alpha;	

TRINITY_DN33016_c0_g1_i1	-0,454589303	Full=60S ribosomal protein L23a;	
TRINITY_DN160_c0_g1_i1	-0,454386557	Full=Hydroxymethylglutaryl-CoA lyase, mitochondrial;	
TRINITY_DN3739_c0_g1_i1	-0,45433336	Full=Peptidyl-prolyl cis-trans isomerase 5;	
TRINITY_DN7095_c0_g1_i1	-0,45338117	Full=Eukaryotic translation initiation factor 3 subunit H {ECO:0000255|HAMAP-Rule:MF_03007};	
TRINITY_DN19022_c0_g1_i1	-0,452437409	Full=Zinc finger protein 32;	
TRINITY_DN5826_c0_g1_i1	-0,451659613		0
TRINITY_DN19110_c0_g1_i1	-0,451337952	Full=28S ribosomal protein S18a, mitochondrial;	
TRINITY_DN10845_c0_g1_i1	-0,449392474	Full=ATP-dependent RNA helicase p62;	
TRINITY_DN5428_c0_g1_i1	-0,449191979	Full=Kinesin heavy chain;	
TRINITY_DN19886_c0_g1_i1	-0,448882472	Full=40S ribosomal protein S7;	
TRINITY_DN5026_c0_g1_i1	-0,446094891	Full=Short-chain specific acyl-CoA dehydrogenase, mitochondrial;	
TRINITY_DN18742_c0_g1_i1	-0,444655257	Full=Krueppel homolog 1;	
TRINITY_DN250_c0_g1_i1	-0,444623922	Full=Lipoyltransferase 1, mitochondrial;	
TRINITY_DN5235_c0_g1_i1	-0,444184685	Full=Protein lifeguard 4;	
TRINITY_DN32363_c0_g1_i1	-0,44405143		0
TRINITY_DN11575_c0_g1_i1	-0,443539024	Full=Myosin heavy chain, muscle;	
TRINITY_DN4041_c0_g1_i1	-0,443441946	Full=Pyrroline-5-carboxylate reductase;	
TRINITY_DN10074_c0_g1_i1	-0,442590366	Full=Elongation of very long chain fatty acids protein AAEL008004;	
TRINITY_DN10879_c0_g1_i1	-0,44213988	Full=Bestrophin-4;	
TRINITY_DN19131_c0_g1_i1	-0,441049804	Full=3-ketoacyl-CoA thiolase, mitochondrial;	
TRINITY_DN23992_c0_g1_i1	-0,440071193	Full=Protein FAM151B;	
TRINITY_DN398_c0_g1_i1	-0,440046354	Full=Abl interactor 2;	
TRINITY_DN11615_c0_g1_i1	-0,438811733	Full=Ribosome biogenesis protein NSA2 homolog;	
TRINITY_DN4764_c0_g1_i1	-0,438381503	Full=Protein extra-macrochaetae;	
TRINITY_DN1966_c0_g1_i1	-0,436785446	Full=Tyrosine-protein kinase transforming protein ros;^Viruses; Retro-transcribing viruses; Retroviridae; Orthoretrovirinae; Alpharetrovirus	
TRINITY_DN5324_c0_g1_i1	-0,43609038	Full=60S ribosomal protein L12;	
TRINITY_DN18887_c0_g1_i1	-0,435608521		0
TRINITY_DN32616_c0_g1_i1	-0,435547636	Full=Aminopeptidase N;	
TRINITY_DN10727_c0_g1_i1	-0,435244052	Full=Uncharacterized protein CG1785;	
TRINITY_DN18628_c0_g1_i1	-0,434727603	Full=ATP synthase subunit d, mitochondrial;	

TRINITY_DN11177_c0_g1_i1	-0,432503885	Full=S-phase kinase-associated protein 1;	
TRINITY_DN22569_c0_g1_i1	-0,432304874	Full=Transmembrane protein 147;	
TRINITY_DN5397_c0_g1_i1	-0,431763188	Full=3-hydroxy-3-methylglutaryl-coenzyme A reductase;	
TRINITY_DN11541_c0_g1_i1	-0,430938072		0
TRINITY_DN10730_c0_g1_i1	-0,430555727	Full=Transcription factor AP-1;	
TRINITY_DN32304_c0_g1_i1	-0,429186146	Full=60S ribosomal protein L18;	
TRINITY_DN5577_c0_g1_i1	-0,428680369	Full=Protein tipE;	
TRINITY_DN7551_c0_g1_i1	-0,426716444		0
TRINITY_DN21473_c0_g1_i1	-0,426659862	Full=40S ribosomal protein S23;	
TRINITY_DN10786_c0_g1_i1	-0,425873968	Full=Eukaryotic translation initiation factor 2 subunit 3;	
TRINITY_DN10744_c0_g1_i1	-0,424784661	Full=Selenide, water dikinase;	
TRINITY_DN19374_c0_g1_i1	-0,424755745	Full=MAP kinase-interacting serine/threonine-protein kinase 1;	
TRINITY_DN8740_c0_g1_i1	-0,423014691	Full=60S ribosomal protein L17;	
TRINITY_DN712_c0_g1_i1	-0,422023024	Full=Mitochondrial-processing peptidase subunit beta;	
TRINITY_DN4722_c0_g1_i1	-0,421786359	Full=Dynein heavy chain, cytoplasmic;	
TRINITY_DN11173_c0_g1_i1	-0,421094771	Full=Diuretic hormone 45;	
TRINITY_DN10699_c0_g1_i1	-0,420281673	Full=Annexin B11;	
TRINITY_DN11697_c0_g1_i1	-0,420055368		0
TRINITY_DN1200_c0_g1_i1	-0,41950649	Full=COP9 signalosome complex subunit 1;	
TRINITY_DN12297_c0_g1_i1	-0,419453489		0
TRINITY_DN5383_c0_g1_i1	-0,417809864	Full=Ubiquitin-conjugating enzyme E2 N;	
TRINITY_DN5088_c0_g1_i1	-0,416569111	Full=Dystrophin, isoform D {ECO:0000303|PubMed:10731132, ECO:0000303|PubMed:17543506};	
TRINITY_DN5006_c0_g1_i1	-0,415876872	Full=Suppressor of lurcher protein 1;	
TRINITY_DN1291_c0_g1_i1	-0,415652214	Full=Protein Dr1;	
TRINITY_DN228_c0_g1_i1	-0,415222957	Full=Stomatin-like protein 2, mitochondrial;	
TRINITY_DN773_c0_g1_i1	-0,413314698	Full=DNA polymerase iota;	
TRINITY_DN10908_c0_g1_i1	-0,412739157	Full=Protein canopy homolog 3;	
TRINITY_DN11062_c0_g1_i1	-0,41203697	Full=Polymerase delta-interacting protein 2;	
TRINITY_DN5117_c0_g1_i1	-0,411484634	Full=Secretory carrier-associated membrane protein 1;	
TRINITY_DN5073_c0_g1_i1	-0,410917473	Full=CCR4-NOT transcription complex subunit 7;	

TRINITY_DN10940_c0_g1_i1	-0,41052848	Full=Proteasome subunit beta type-7;	
TRINITY_DN6277_c0_g1_i1	-0,40900877	Full=Sodium-coupled monocarboxylate transporter 2;	
TRINITY_DN8206_c0_g1_i1	-0,407685261	Full=Nuclear factor interleukin-3-regulated protein;	
TRINITY_DN583_c0_g1_i1	-0,407388627	Full=ATP synthase subunit beta, mitochondrial;	
TRINITY_DN6527_c0_g1_i1	-0,406416114		0
TRINITY_DN18851_c0_g1_i1	-0,404902859	Full=Transaldolase;	
TRINITY_DN4871_c0_g1_i1	-0,404633928	Full=Collagen alpha-1(XI) chain;	
TRINITY_DN5903_c0_g1_i1	-0,40365751	Full=Thioredoxin, mitochondrial;	
TRINITY_DN11364_c0_g1_i1	-0,403503037	Full=Transcription initiation factor TFIID subunit 5;	
TRINITY_DN551_c0_g1_i1	-0,402387583	Full=Sodium leak channel non-selective protein;	
TRINITY_DN419_c0_g1_i1	-0,401529504	Full=Metalloproteinase inhibitor 3;	
TRINITY_DN6273_c0_g1_i1	-0,400245269	Full=60S acidic ribosomal protein P2;	
TRINITY_DN5206_c0_g1_i1	-0,400023748	Full=60S ribosomal protein L21;	
TRINITY_DN4670_c0_g1_i1	-0,399830397	Full=DnaJ protein homolog 1;	
TRINITY_DN33055_c0_g1_i1	-0,397831278	Full=Putative ATP synthase subunit f, mitochondrial;	
TRINITY_DN780_c0_g1_i1	-0,397551659	Full=S-adenosylmethionine synthase;	
TRINITY_DN1323_c0_g1_i1	-0,397162867	Full=Neprilysin-11;	
TRINITY_DN11339_c0_g1_i1	-0,39685869	Full=Enoyl-CoA hydratase domain-containing protein 2, mitochondrial;	
TRINITY_DN691_c0_g1_i1	-0,396160436	Full=26S proteasome non-ATPase regulatory subunit 14;	
TRINITY_DN1393_c0_g1_i1	-0,395604968	Full=Facilitated trehalose transporter Tret1 {ECO:0000250|UniProtKB:Q7PIR5};	
TRINITY_DN5565_c0_g1_i1	-0,39488434	Full=Mitochondrial import receptor subunit TOM20 homolog;	
TRINITY_DN32525_c0_g1_i1	-0,393080873	Full=60S ribosomal protein L13;	
TRINITY_DN5119_c0_g1_i1	-0,39281959	Full=ATP-dependent RNA helicase p62;	
TRINITY_DN18811_c0_g1_i1	-0,392245205	Full=Stathmin-4;	
TRINITY_DN5379_c0_g1_i1	-0,392146325	Full=ATP synthase mitochondrial F1 complex assembly factor 2;	
TRINITY_DN5730_c0_g1_i1	-0,391161417		0
TRINITY_DN4915_c0_g1_i1	-0,38996348	Full=Protein slit;	
TRINITY_DN18800_c0_g1_i1	-0,389314162	Full=Probable aconitate hydratase, mitochondrial;	
TRINITY_DN1639_c0_g1_i1	-0,388274822		0
TRINITY_DN108_c0_g1_i1	-0,387984226	Full=Interferon-inducible double-stranded RNA-dependent protein kinase activator A;	

TRINITY_DN5034_c0_g1_i1	-0,387325876	Full=ATP-binding cassette sub-family E member 1;	
TRINITY_DN9224_c0_g1_i1	-0,387249887	Full=Cathepsin L;	
TRINITY_DN18844_c0_g1_i1	-0,386774969	Full=Ubiquitin-conjugating enzyme E2 Q1;	
TRINITY_DN342_c0_g1_i1	-0,3859353	Full=HIG1 domain family member 1A, mitochondrial;	
TRINITY_DN11493_c0_g1_i1	-0,385930451	Full=Electron transfer flavoprotein subunit beta {ECO:0000305};	
TRINITY_DN18961_c0_g1_i1	-0,385502645	Full=60S ribosomal protein L5;	
TRINITY_DN18632_c0_g1_i1	-0,383475169	Full=ATP synthase subunit gamma, mitochondrial;	
TRINITY_DN18649_c0_g1_i1	-0,382825748	Full=Arginine--tRNA ligase, cytoplasmic;	
TRINITY_DN12067_c0_g1_i1	-0,382525085	Full=Leucine-rich repeat and immunoglobulin-like domain-containing nogo receptor-interacting protein 2;	
TRINITY_DN18711_c0_g1_i1	-0,382103606	Full=Serine/threonine-protein kinase mig-15;	
TRINITY_DN5076_c0_g1_i1	-0,381273393	Full=2-oxoglutarate and iron-dependent oxygenase domain-containing protein 3;	
TRINITY_DN5183_c0_g1_i1	-0,381174185	Full=Superoxide dismutase [Mn], mitochondrial;	
TRINITY_DN11280_c0_g1_i1	-0,381148037	Full=Ets DNA-binding protein pokkuri;	
TRINITY_DN333_c0_g1_i1	-0,38099708	Full=Hepatocyte growth factor-regulated tyrosine kinase substrate;	
TRINITY_DN6765_c0_g1_i1	-0,380649268	Full=Diacylglycerol kinase epsilon;	
TRINITY_DN11022_c0_g1_i1	-0,379655347	Full=Alpha-endosulfine;	
TRINITY_DN1198_c0_g1_i1	-0,37944265	Full=Sodium/potassium/calcium exchanger 4;	
TRINITY_DN14888_c0_g1_i1	-0,379277796		0
TRINITY_DN18951_c0_g1_i1	-0,378911768	Full=Ubiquitin domain-containing protein UBFD1;	
TRINITY_DN11450_c0_g1_i1	-0,376428438	Full=Trifunctional enzyme subunit beta, mitochondrial;	
TRINITY_DN487_c0_g1_i1	-0,376368372		0
TRINITY_DN10724_c0_g1_i1	-0,375682782	Full=Alanine--tRNA ligase, cytoplasmic {ECO:0000255|HAMAP-Rule:MF_03133};	
TRINITY_DN18724_c0_g1_i1	-0,375442274	Full=Cytochrome c1, heme protein, mitochondrial;	
TRINITY_DN32297_c0_g1_i1	-0,375225573	Full=Uncharacterized protein CG16817;	
TRINITY_DN4892_c0_g1_i1	-0,374213066	Full=Transient receptor potential channel pyrexia;	
TRINITY_DN2569_c0_g1_i1	-0,373749291		0
TRINITY_DN5277_c0_g1_i1	-0,373472779	Full=Ubiquitin thioesterase otubain-like;	
TRINITY_DN256_c0_g1_i1	-0,373373429	Full=Protein NDRG3;	
TRINITY_DN964_c0_g1_i1	-0,370910069	Full=Calcium-transporting ATPase sarcoplasmic/endoplasmic reticulum type;	

TRINITY_DN19363_c0_g1_i1	-0,37065719	Full=40S ribosomal protein S13;	
TRINITY_DN5475_c0_g1_i1	-0,369545228	Full=Sodium-dependent nutrient amino acid transporter 1 {ECO:0000250|UniProtKB:Q9W4C5};	
TRINITY_DN5399_c0_g1_i1	-0,368526541	Full=Phospholipid hydroperoxide glutathione peroxidase, mitochondrial;	
TRINITY_DN19074_c0_g1_i1	-0,368151477	Full=ATP synthase subunit O, mitochondrial;	
TRINITY_DN5249_c0_g1_i1	-0,367932073	Full=Cytochrome c oxidase subunit 5B, mitochondrial {ECO:0000250|UniProtKB:P00428};	
TRINITY_DN6062_c0_g1_i1	-0,367917846	Full=28S ribosomal protein S9, mitochondrial;	
TRINITY_DN18659_c0_g1_i1	-0,367336524	Full=Peroxiredoxin 1;	
TRINITY_DN501_c0_g1_i1	-0,365800496	Full=Armadillo segment polarity protein;	
TRINITY_DN18745_c0_g1_i1	-0,365441424	Full=NADH dehydrogenase [ubiquinone] 1 alpha subcomplex subunit 11;	
TRINITY_DN5573_c0_g1_i1	-0,365235531		0
TRINITY_DN11851_c0_g1_i1	-0,364921058	Full=Riboflavin kinase;	
TRINITY_DN7380_c0_g1_i1	-0,364699932	Full=Myosin-2 essential light chain;	
TRINITY_DN19322_c0_g1_i1	-0,36310522	Full=V-type proton ATPase 16 kDa proteolipid subunit;	
TRINITY_DN11599_c0_g1_i1	-0,36291118	Full=Eukaryotic translation initiation factor 4E;	
TRINITY_DN5238_c0_g1_i1	-0,362572889	Full=Cysteine synthase;	
TRINITY_DN18640_c0_g1_i1	-0,361075166		0
TRINITY_DN13345_c0_g1_i1	-0,359852593	Full=60S ribosomal protein L19;	
TRINITY_DN19014_c0_g1_i1	-0,357928804	Full=60S ribosomal protein L13a;	
TRINITY_DN10706_c0_g1_i1	-0,357659262	Full=Cytochrome b-c1 complex subunit 2, mitochondrial;	
TRINITY_DN617_c0_g1_i1	-0,355885684	Full=Sperm-associated antigen 6;	
TRINITY_DN19500_c0_g1_i1	-0,355420447		0
TRINITY_DN12102_c0_g1_i1	-0,35526847	Full=Phosphate carrier protein, mitochondrial;	
TRINITY_DN19958_c0_g1_i1	-0,354756377	Full=40S ribosomal protein S24;	
TRINITY_DN13247_c0_g1_i1	-0,352688305	Full=60S ribosomal protein L11;	
TRINITY_DN2383_c0_g1_i1	-0,352163576		0
TRINITY_DN5211_c0_g1_i1	-0,352012089	Full=Histone H3.3;	
TRINITY_DN5499_c0_g1_i1	-0,351348658		0
TRINITY_DN284_c0_g1_i1	-0,351190151	Full=Enolase;	
TRINITY_DN32291_c0_g1_i1	-0,35107417	Full=27 kDa hemolymph protein;	
TRINITY_DN4625_c0_g1_i1	-0,35093626	Full=Cytochrome P450 CYP12A2;	

TRINITY_DN21547_c0_g1_i1	-0,34931163	Full=Actin, cytoplasmic A3a;	
TRINITY_DN9151_c0_g1_i1	-0,349067301		0
TRINITY_DN5668_c0_g1_i1	-0,34752191	Full=Nucleoplasmin-like protein;	
TRINITY_DN10827_c0_g1_i1	-0,346416112	Full=Glutamate dehydrogenase, mitochondrial;	
TRINITY_DN5287_c0_g1_i1	-0,344953031	Full=Beta-ureidopropionase;	
TRINITY_DN11026_c0_g1_i1	-0,344437554		0
TRINITY_DN5812_c0_g1_i1	-0,343826774	Full=40S ribosomal protein S5;	
TRINITY_DN5062_c0_g1_i1	-0,343458248	Full=Proteasome subunit beta type-1;	
TRINITY_DN18687_c0_g1_i1	-0,342796286	Full=Probable malate dehydrogenase, mitochondrial;	
TRINITY_DN486_c0_g1_i1	-0,341372303	Full=Probable isocitrate dehydrogenase [NAD] subunit alpha, mitochondrial;	
TRINITY_DN19750_c0_g1_i1	-0,341169504	Full=40S ribosomal protein S10;	
TRINITY_DN395_c0_g1_i1	-0,340901983	Full=Sorting nexin lst-4 {ECO:0000250|UniProtKB:Q8WV41};	
TRINITY_DN908_c0_g1_i1	-0,340370135	Full=Protein lev-9;	
TRINITY_DN11252_c0_g1_i1	-0,339152738	Full=S-adenosylmethionine decarboxylase proenzyme;	
TRINITY_DN32821_c0_g1_i1	-0,338791043	Full=60S ribosomal protein L37a;	
TRINITY_DN18685_c0_g1_i1	-0,337841481	Full=Pyruvate kinase;	
TRINITY_DN11490_c0_g1_i1	-0,336281412	Full=Mobility group protein 1A;	
TRINITY_DN10930_c0_g1_i1	-0,335452322	Full=Isocitrate dehydrogenase [NADP], mitochondrial;	
TRINITY_DN10700_c0_g1_i1	-0,334567972	Full=Maltase A2;	
TRINITY_DN131_c0_g1_i1	-0,334348094	Full=Protein disulfide-isomerase;	
TRINITY_DN18889_c0_g1_i1	-0,332426424	Full=Immunoglobulin domain-containing protein oig-1 {ECO:0000305};	
TRINITY_DN147_c0_g1_i1	-0,331629067	Full=Trifunctional enzyme subunit alpha, mitochondrial;	
TRINITY_DN18763_c0_g1_i1	-0,331397397	Full=NADH dehydrogenase [ubiquinone] iron-sulfur protein 7, mitochondrial;	
TRINITY_DN5332_c0_g1_i1	-0,330414391	Full=Serine/threonine-protein phosphatase 2A 56 kDa regulatory subunit delta isoform;	
TRINITY_DN6190_c0_g1_i1	-0,330205768	Full=Ras-related protein Rap-1b;	
TRINITY_DN4631_c0_g1_i1	-0,32919844	Full=26S proteasome non-ATPase regulatory subunit 11;	
TRINITY_DN10721_c0_g1_i1	-0,327725965	Full=Transmembrane emp24 domain-containing protein bai {ECO:0000250|UniProtKB:Q9I7K5};	
TRINITY_DN16948_c0_g1_i1	-0,326138515	Full=RNA-binding protein Rsf1;	
TRINITY_DN20_c0_g1_i1	-0,325719402		0

TRINITY_DN32940_c0_g1_i1	-0,325152225	Full=60S ribosomal protein L9;	
TRINITY_DN482_c0_g1_i1	-0,323971699	Full=Guanine nucleotide-binding protein subunit beta-like protein;	
TRINITY_DN19960_c0_g1_i1	-0,323298762	Full=NADH dehydrogenase [ubiquinone] 1 beta subcomplex subunit 9;	
TRINITY_DN11056_c0_g1_i1	-0,321641873	Full=Putative neuropeptide precursor protein {ECO:0000303|PubMed:19540422};	
TRINITY_DN4634_c0_g1_i1	-0,320549057		0
TRINITY_DN32446_c0_g1_i1	-0,319529305	Full=60S ribosomal protein L44;	
TRINITY_DN5519_c0_g1_i1	-0,316133109	Full=2-hydroxyacyl-CoA lyase 1;	
TRINITY_DN285_c0_g1_i1	-0,315226663	Full=Ras-related protein Rab-1A;	
TRINITY_DN665_c0_g1_i1	-0,314911289	Full=Microtubule-associated protein RP/EB family member 3;	
TRINITY_DN11320_c0_g1_i1	-0,313715815	Full=Protein lethal(2)essential for life;	
TRINITY_DN392_c0_g1_i1	-0,306464752	Full=Sodium- and chloride-dependent GABA transporter ine;	
TRINITY_DN807_c0_g1_i1	-0,306115214	Full=BTB/POZ domain-containing protein KCTD16;	
TRINITY_DN38750_c0_g1_i1	-0,305934884	Full=60S ribosomal protein L35;	
TRINITY_DN4729_c0_g1_i1	-0,304511457	Full=Eukaryotic initiation factor 4A;	
