# Supplementary figures and images for: Sublethal Exposure Effects of the Neonicotinoid Clothianidin Strongly Modify the Brain Transcriptome and Proteome in the Male Moth Agrotis ipsilon
Source: Insects. 2021 Feb 11;12(2):152. doi: 10.3390/insects12020152 (PMC7916958; doi:10.3390/insects12020152)

A

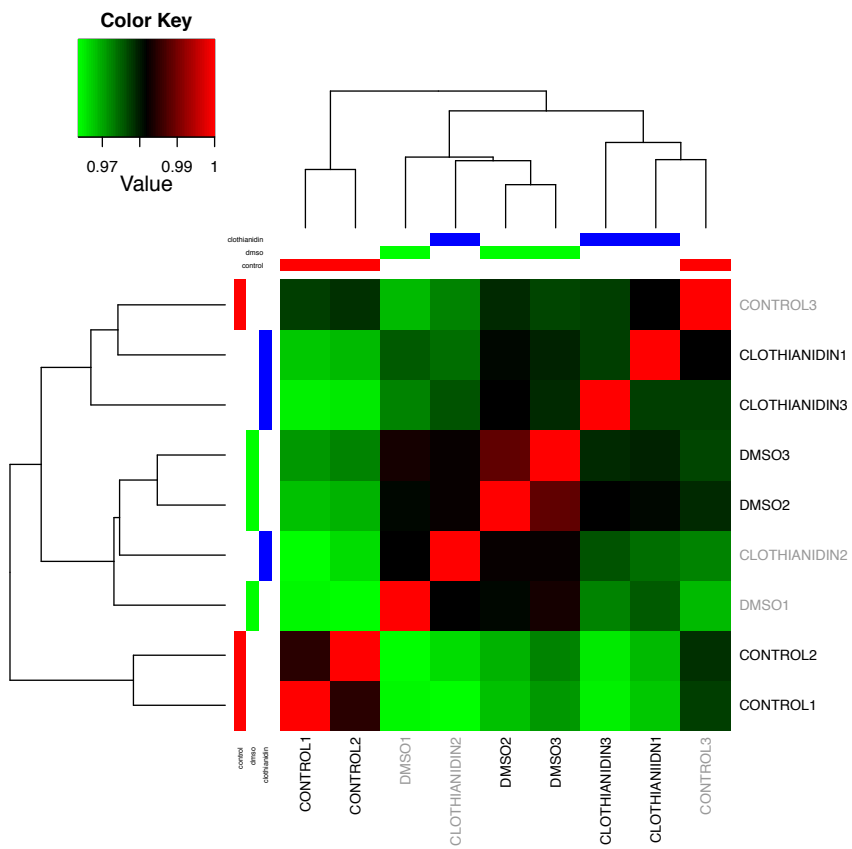

B

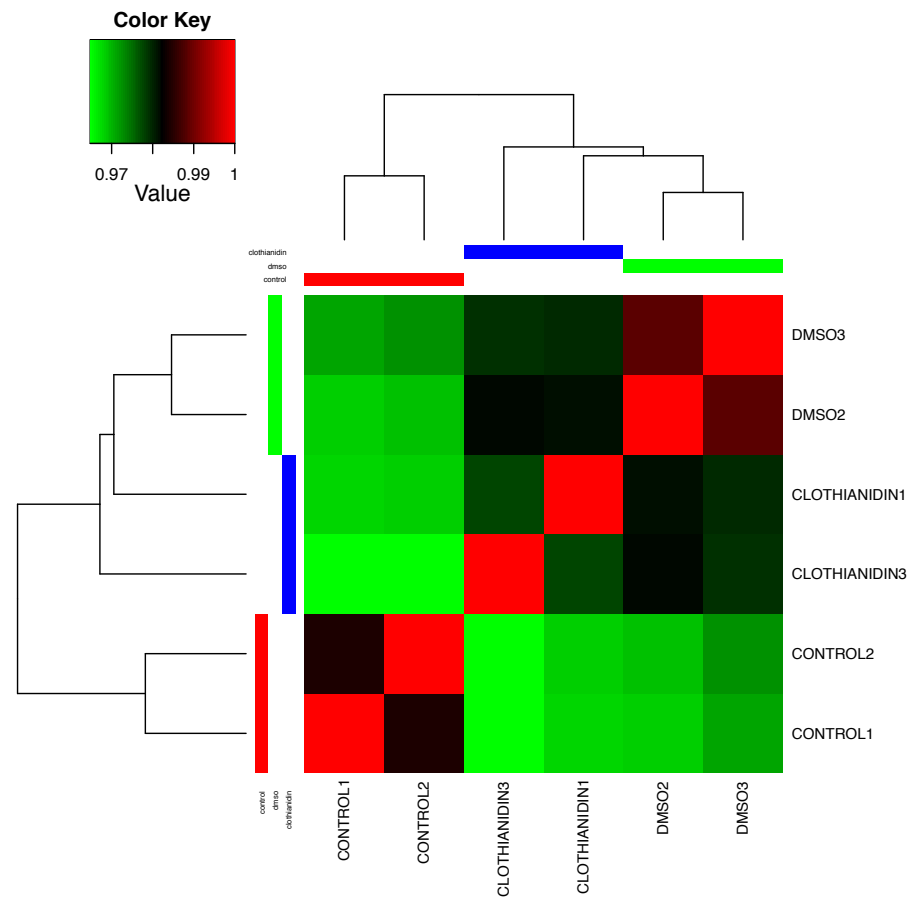

Supplement: Supplementary file 1 [file insects-12-00152-s001.tgz › SuppData1.pdf]
